# Practical, metal-free remote heteroarylation of amides *via* unactivated C(sp^3^)–H bond functionalization†
†Electronic supplementary information (ESI) available. See DOI: 10.1039/c9sc02564b


**DOI:** 10.1039/c9sc02564b

**Published:** 2019-06-11

**Authors:** Nana Tang, Xinxin Wu, Chen Zhu

**Affiliations:** a Key Laboratory of Organic Synthesis of Jiangsu Province , College of Chemistry , Chemical Engineering and Materials Science , Soochow University , 199 Ren-Ai Road , Suzhou , Jiangsu 215123 , China . Email: chzhu@suda.edu.cn; b Key Laboratory of Synthesis Chemistry of Natural Substances , Shanghai Institute of Organic Chemistry , Chinese Academy of Science , 345 Lingling Road , Shanghai 200032 , China

## Abstract

Development of practical methods for the production of multi-functionalized amides is one of the most important topics in both synthetic chemistry and drug discovery. Disclosed herein is a new, efficient, site-selective heteroarylation of amides *via* C(sp^3^)–H bond functionalization. Amidyl radicals are directly generated from the amide N–H bonds under mild conditions, which trigger the subsequent 1,5-HAT process. A wide scope of aliphatic amides including carboxamides, sulfonamides, and phosphoramides are readily modified at remote C(sp^3^)–H bonds by installing diverse heteroaryl groups. Borne out of pragmatic consideration, this protocol can be used for the late-stage functionalization of amides.

## Abstract 2

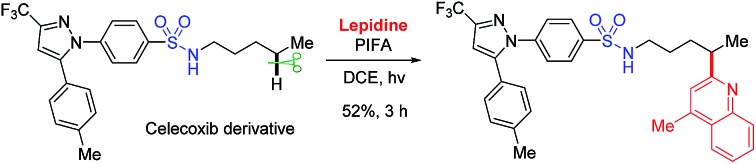
A simple and practical approach for the regioselective heteroarylation of amides *via* unactivated C(sp^3^)–H bond functionalization is described.

## Introduction

**Scheme 1:**
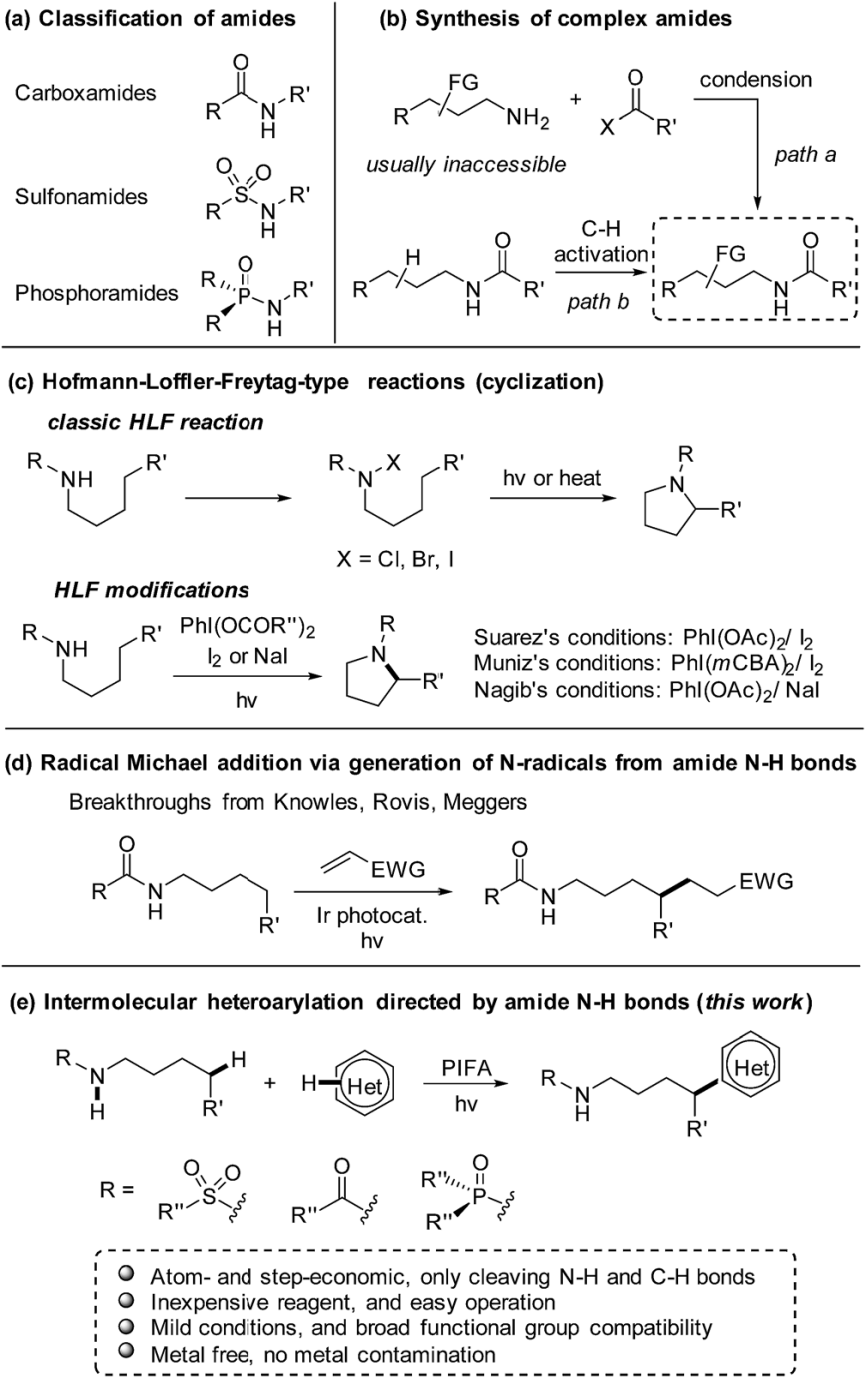
Amide N–H bond-directed functionalization of C(sp^3^)–H bonds. (a) Classification of amides. (b) Synthesis of complex amides. (c) Hofmann–Loffler–Freytag-type reactions. *m*CBA = 3-chlorobenzoate. (d) Intermolecular alkylation *via* generation of N-radicals from amide N–H bonds. (e) Intermolecular heteroarylation directed by amide N–H bonds.

As a class of paramount chemical structures, amides consisting of carboxamides, sulfonamides, and phosphoramides are ubiquitous in natural and synthetic products (Scheme 1a). They constitute the basic framework of proteins in the form of peptides in life systems, and are engaged as key candidates for structure design in drug development. An analysis reveals that amide formation is one of the most frequently occurring reactions used in current medicinal chemistry.^1^ Generally, amides are readily obtained from the condensation of amines with carboxylic/sulfonic/phosphoric acids and derivatives (Scheme 1b, path a). Alternatively, direct installation of the target functional group at the aliphatic chain of amides *via* C(sp^3^)–H bond functionalization provides an ingenious and powerful access to the multi-functionalized amides (Scheme 1b, path b),^2^ in particular when the complex alkylamine precursors are inaccessible for the condensation approach.

Inspired by the classic Hofmann–Löffler–Freytag (HLF) reaction,^3^ recently the regioselective functionalization of amides *via* C(sp^3^)–H functionalization enabled by hydrogen atom transfer (HAT) has gained intense interest.^4^ Due to the high bond-dissociation free energy (BDFE) of N–H bonds (107–110 kcal mol^–1^),^5^ it is a formidable challenge to directly generate amidyl radicals from N–H bonds. Therefore, amides are usually elaborated to other surrogates that deliver amidyl radicals under mild conditions.^6^,^7^ Elegant modifications of HLF reaction from Suárez^8^ and others^9^,^10^ convert the amide N–H bonds to amidyl radicals by homolysis of the *in situ* formed N–I species. But those reactions are limited to the cyclization to afford the pyrrolidine derivatives (Scheme 1c). The recent breakthroughs in the radical Michael addition achieved by Knowles,^11^ Rovis,^12^ and Meggers^13^ significantly increase the atom- and step-economy of the amide-directed C(sp^3^)–H functionalization (Scheme 1d). Despite the notable progress, the production of amidyl radicals direct from amide N–H bonds and applications in the intermolecular transformation of C(sp^3^)–H bonds still remains scarce.

Heteroarenes are extensively found in biologically active compounds, including almost half of the top 200 pharmaceuticals by prescriptions.^14^ It is of great synthetic value to introduce various heteroaryl groups to amides by C–H functionalization. To the best of our knowledge, only two examples from Zhu7*g* and Nagib,7*l* respectively, reported the remote C–H heteroarylation of amides. The preformed N–F derivative of amides were employed as starting materials. Both reports focused on the C–H arylation, while only a few examples on heteroarylation were involved. Inspired by our recent achievements in the alcohol-directed C(sp^3^)–H heteroarylation,^15^ we herein report a novel, metal-free regioselective heteroarylation of amides *via* C(sp^3^)–H bond functionalization (Scheme 1e). A broad range of heteroarenes as well as amides including carboxamides, sulfonamides, and phosphoramides are tolerated in the reaction. Amidyl radicals are readily generated from the amide N–H bonds under mild conditions, manifesting the step-economy of the protocol. The metal-free conditions avoid the metal contamination of products, boosting the potential use in medicinal chemistry. Furthermore, the method features the use of inexpensive reagent and easy operation, offering a practical approach for the late-stage functionalization of amides.

## Results and discussion

**Scheme 2:**
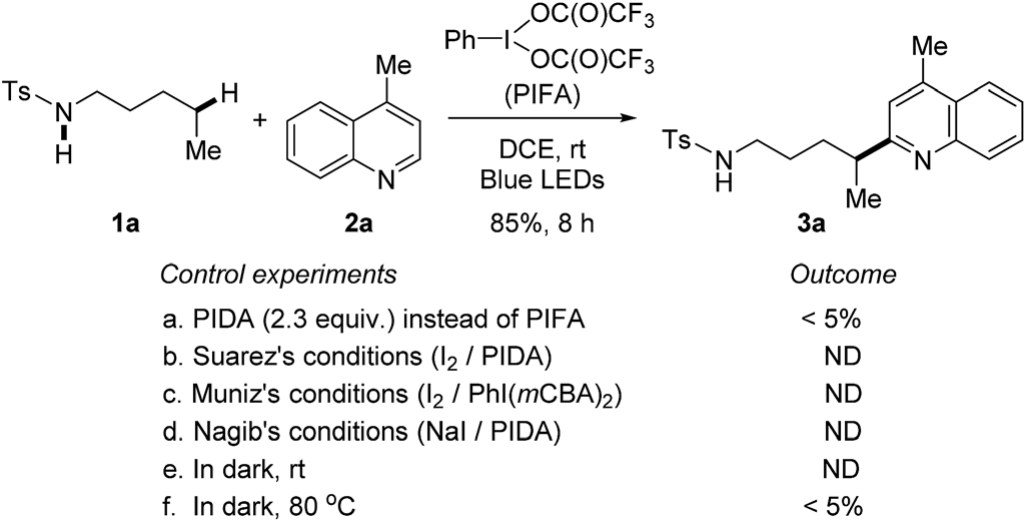
Control experiments. Standard reaction conditions: tosylamide **1a** (0.6 mmol), lepidine **2a** (0.2 mmol), and PIFA (0.46 mmol) in DCE (2 mL), irradiated by 2 × 50 W blue LEDs at rt.

At the outset, a systematic survey of reaction parameters was carried out to define the optimized conditions (for details, see ESI†). In the presence of visible-light irradiation and phenyliodine bis(trifluoroacetate) (PIFA), the reaction of tosylamide **1a** with lepidine **2a** proceeded smoothly to furnish the desired Minisci-type product **3a** in 85% yield (Scheme 2). It should be noted that the use of excess amides significantly improved the yield, but the rest of amides could be recovered during the purification. The dual-role PIFA that acted as both the initiator of amidyl radical and oxidant was crucial to the reaction outcome. Variation of PIFA to either PIDA or the conditions valid for the HLF reactions involving hypervalent iodine reagents (Scheme 1c) did not efficiently afford the desired product, suggesting that the current reaction pathway should be distinct to the previous reports. The reaction did not take place without the photo-irradiation or while heating the reaction in dark.

**Scheme 3:**
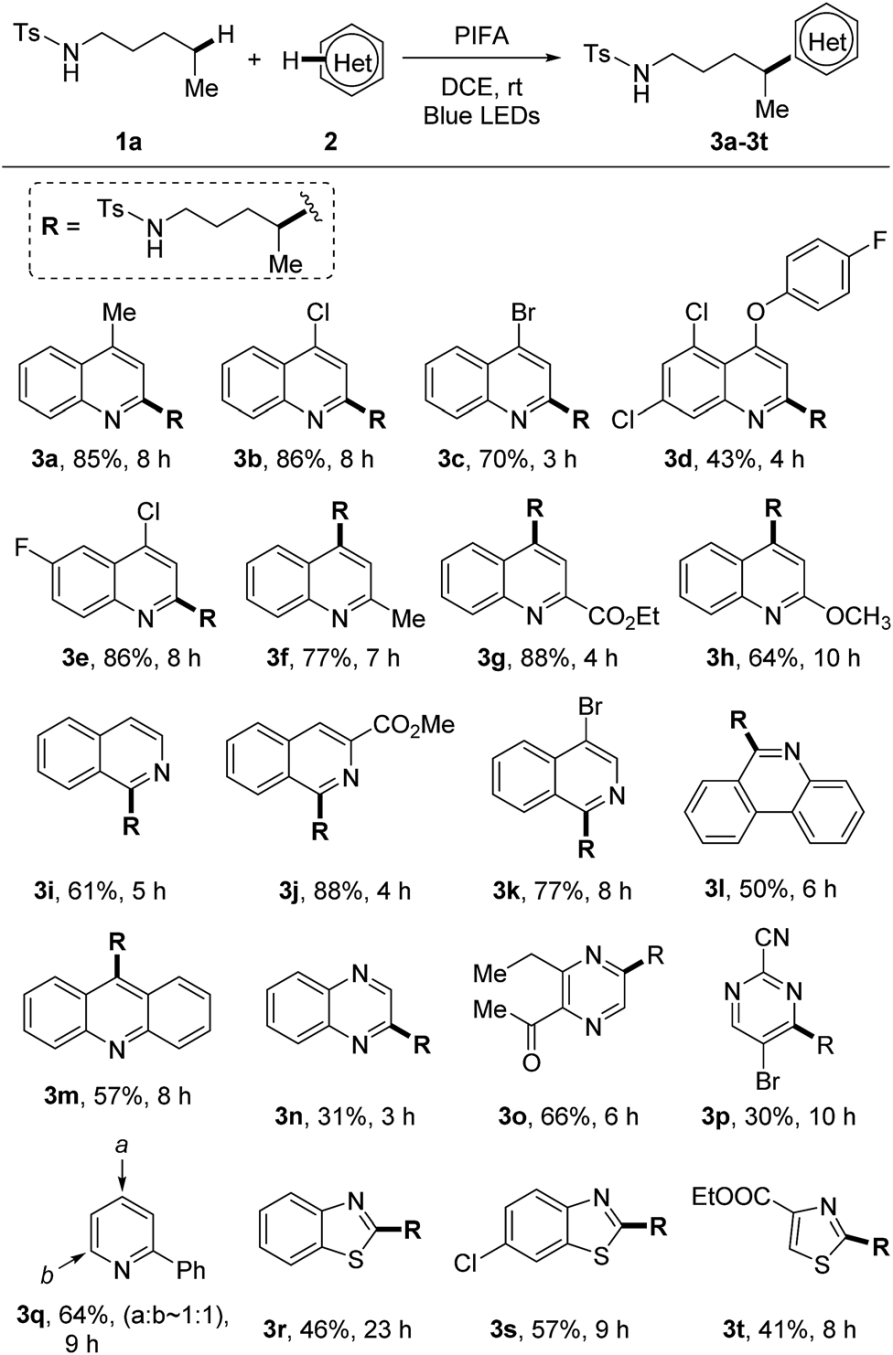
Scope of heteroarenes. Reaction conditions: tosylamide **1a** (0.6 mmol), heteroarene **2** (0.2 mmol), and PIFA (0.46 mmol) in DCE (2 mL), irradiated by 2 × 50 W blue LEDs at rt. Yields of isolated products are given.

With the optimized reaction conditions in hand, we firstly examined the scope of heteroarenes (Scheme 3). A variety of six-membered nitrogen-containing heteroarenes were proved to be suitable reaction partners. Lots of functional groups were well tolerated under the mild conditions. Various quinolines were readily alkylated at either *ortho*- or *para*-position regardless of the electronic characteristics, leading to the corresponding products **3a–3h**. The alkylation of isoquinolines only occurred at the 1-position (**3i–3k**). Aryl bromide remained intact during the reaction, reserving a platform for product processing by cross couplings (**3c** and **3k**). While other heteroarenes such as phenanthridine (**3l**), acridine (**3m**), quinoxaline (**3n**), pyrazine (**3o**), and pyrimidine (**3p**) resulted in single alkylated products, the conversion of pyridine led to a mixture of regio-isomers (**3q**). The five-membered heteroarenes are generally challenging substrates for the Minisci reaction due to the disfavored high electron-density. In this reaction, the reaction of benzothiazoles and thiazoles afforded the desired products **3r–3t** in synthetically useful yields.

**Scheme 4:**
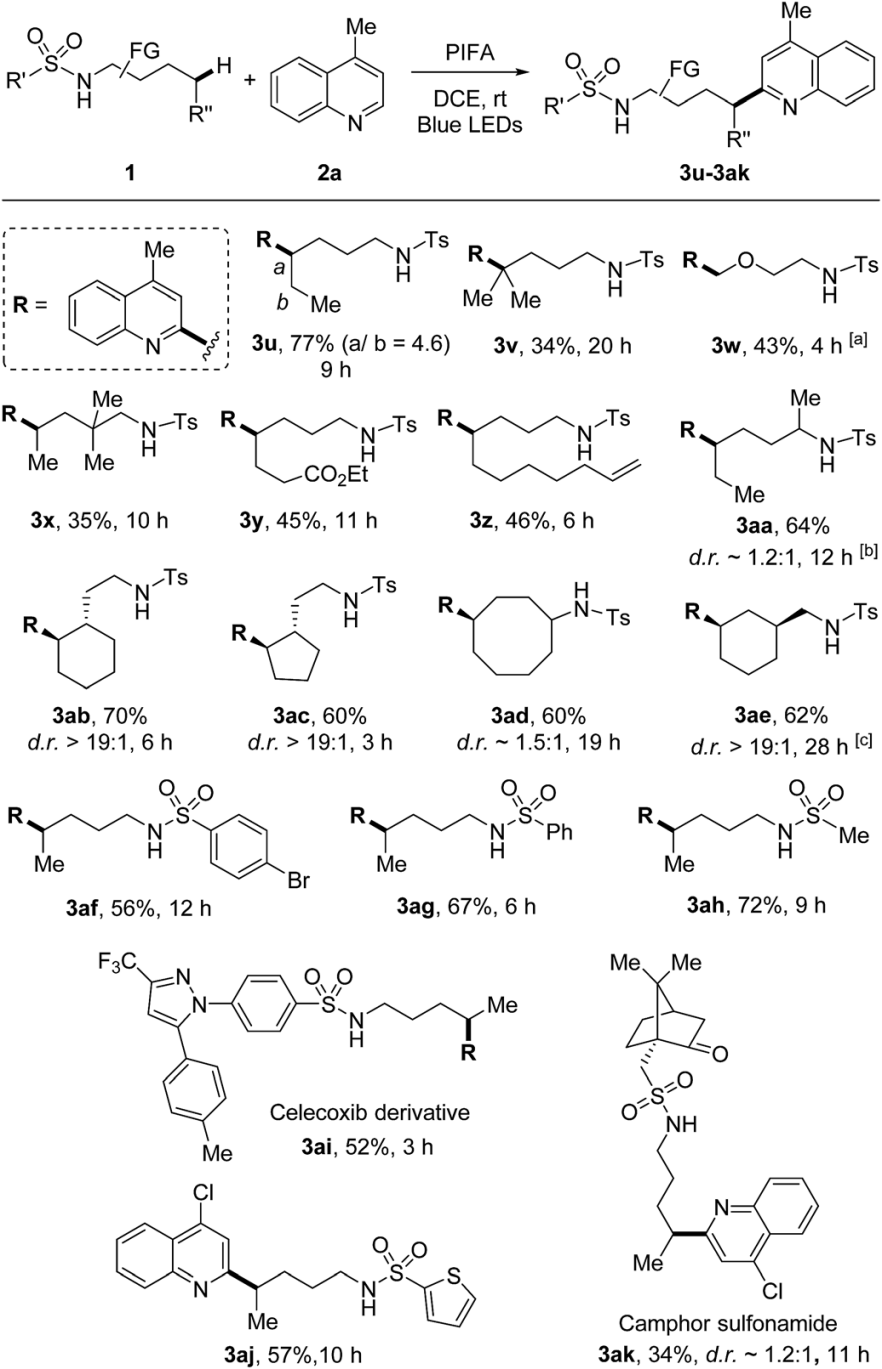
Variation of sulfonamides. Reaction conditions: sulfonamides **1** (0.6 mmol), quinoline **2a** (0.2 mmol), and PIFA (0.46 mmol) in DCE (2 mL), irradiated by 2 × 50 W blue LEDs at rt. Yields of isolated products are given. ^a^5.0 equiv. amide was used. ^b^PIFA (0.92 mmol), added in four portions (every 3 h), ^c^PIFA (0.92 mmol), added in two portions (the second portion was added after 12 h).

The scope of sulfonamides was next evaluated (Scheme 4). A number of sulfonamides including both linear and cyclic amides were suitable substrates. While the adduct **3u** was obtained in the form of regio-isomers *via* 1,5- and 1,6-HAT, other products **3v–3ak** were generated in unique regioselectivity. In addition to secondary C–H bond, tertiary C–H bond was also readily reacted (**3v**), albeit resulting in lower yield. Additionally, primary C(sp^3^)–H bond adjacent to oxygen was also smoothly functionalized (**3w**). Benzylic C–H bonds were not suitable under the current conditions, the HLF-type byproduct was identified as pyrrolidine obtained from the over-oxidation of benzylic radical. Terminal alkene which is normally sensitive to radical conditions was compatible in this transformation (**3z**). The reaction directed by secondary amide offered the outcome comparable to that of primary amide (**3aa**). Site-selective abstraction of the C–H bonds on cycloalkanes provided an efficient approach for the decoration of cycloalkyl skeletons (**3ab–3ae**). Moreover, tosylamide could be altered to other sulfonamides, such as the aryl (**3af** and **3ag**), heteroaryl (**3aj**), and alkyl (**3ah** and **3ak**) substituted sulfonamides. Notably, this method could be applied to the modification of complex natural products and drug derivatives (**3ai** and **3ak**). It should be mentioned that while determining the relative configuration of the products with low d.r. values (*e.g.*, **3aa**, **3ad**, **3ak**) was not necessary, for other stereospecific cases (d.r. >19 : 1, *e.g.*, **3ab**, **3ac**, **3ae**) the thermodynamically preferred products could be assigned as the major isomers in theory.

**Scheme 5:**
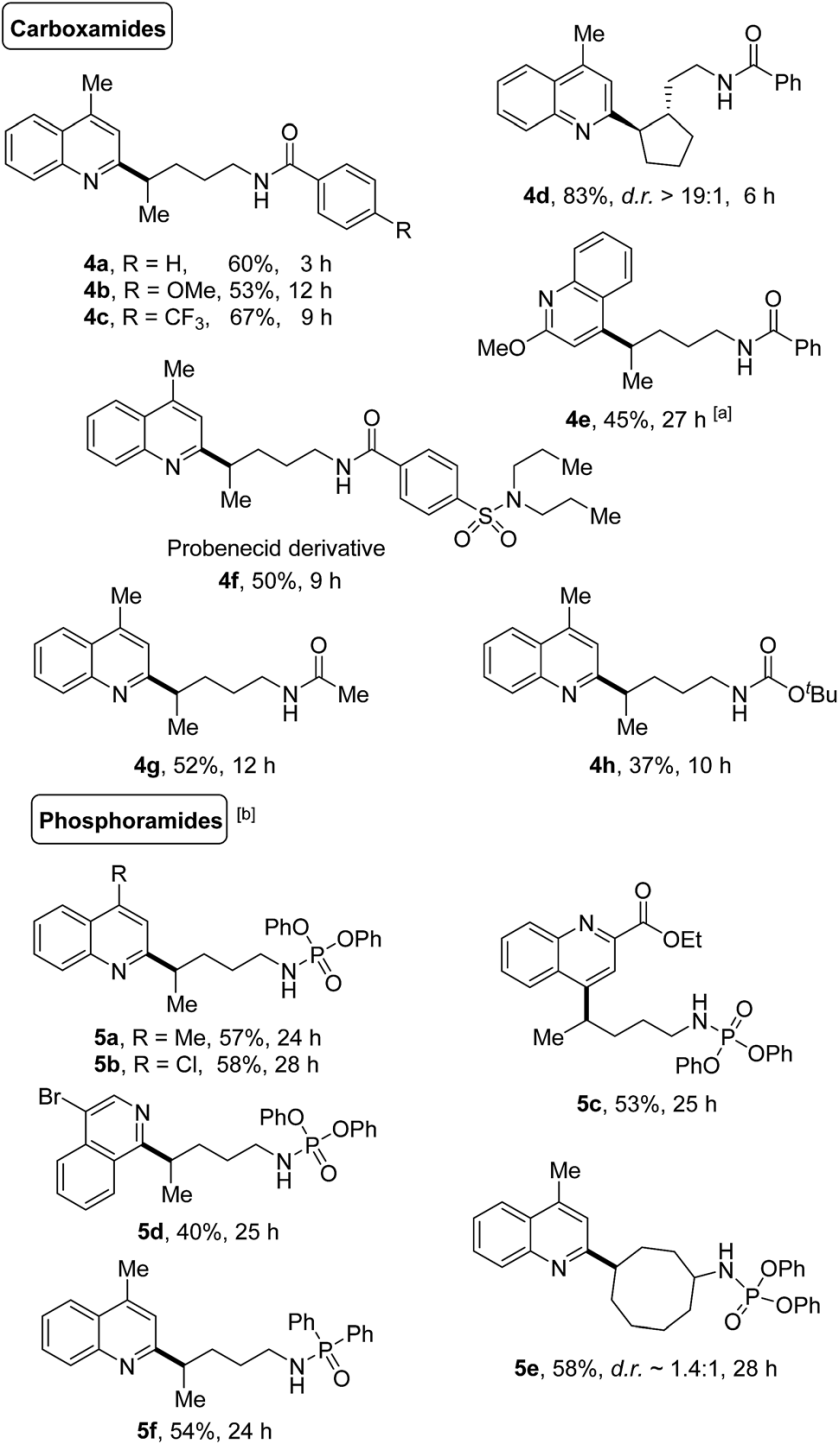
Transformation of carboxamides and phosphoramides. Reaction conditions: amides (0.6 mmol), quinoline (0.2 mmol), and PIFA (0.46 mmol) in DCE (2 mL), irradiated by 2 × 50 W blue LEDs at rt. Yields of isolated products are given. ^a^PIFA (0.46 mmol), added in two portions (the second portion was added after 12 h). ^b^PIFA (0.92 mmol), added in two portions (the second portion was added after 12 h).

The generality of this protocol was further illustrated by the heteroarylation of carboxamides and phosphoramides (Scheme 5). A portfolio of electron-rich or deficient benzamides (**4a–4f**), acetamide (**4g**), and even the Boc-protected amide (**4h**) served as competent precursors for the transformation. Only the *δ*-functionalized products were obtained, no matter whether linear or cyclic amides were used. Likewise, various phosphoramide derivatives such as phosphoramidates (**5a–5e**) and phosphinamide (**5f**) also provided the site-specific C–H heteroarylation exclusively at the *δ*-position, furnishing the desired products in synthetically useful yields.

**Scheme 6:**
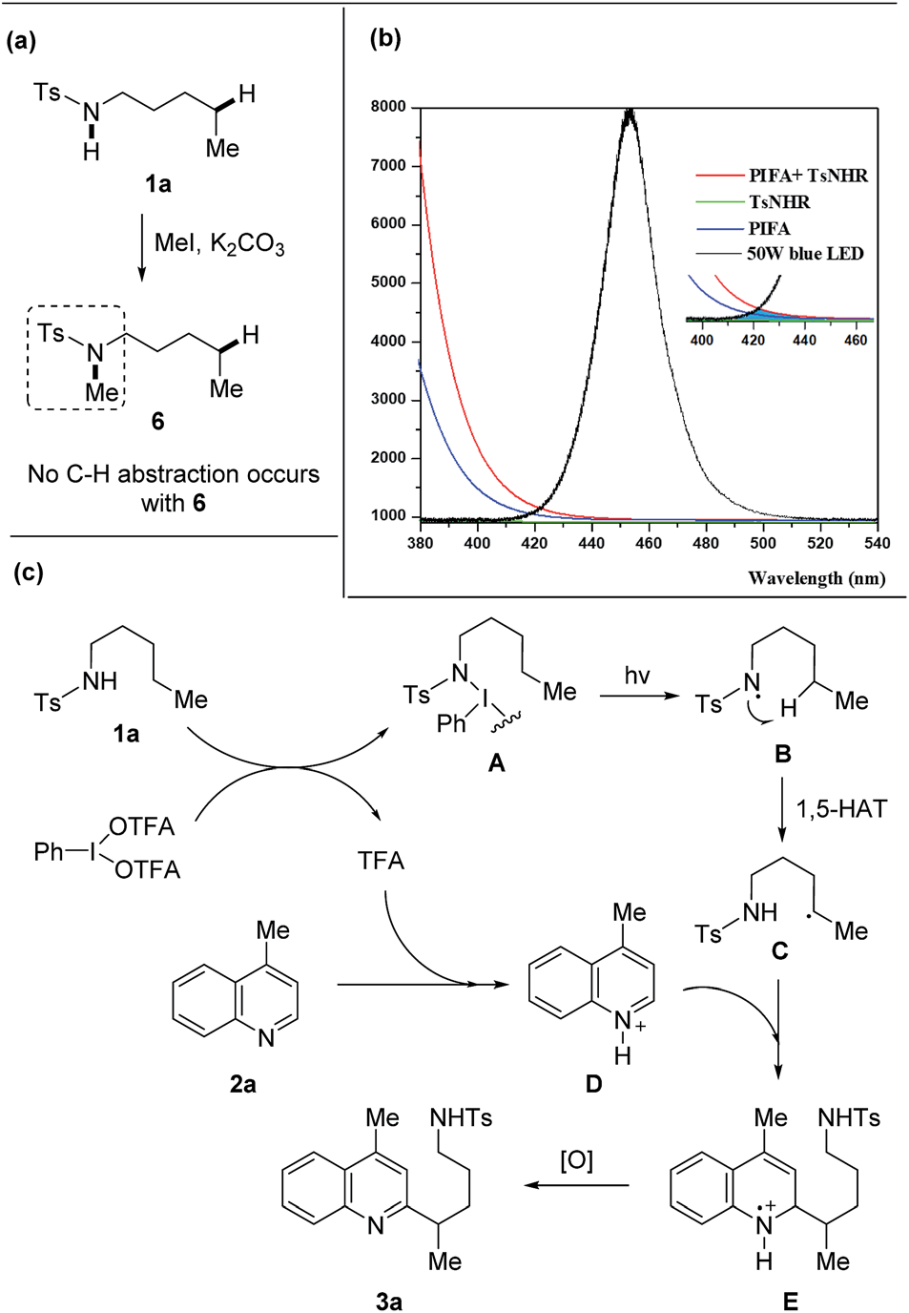
Mechanistic investigation and proposed mechanism. (a) Reaction of tosylamide without N–H bond. (b) Absorption spectra (**1a** + PIFA) and emission spectra (blue LEDs). (c) Proposed mechanism.

A set of experiments were performed to shine light on the mechanism. The free N–H bond of amides was requisite to the transformation as the heteroarylation did not proceed with the methylated tosylamide **6** (Scheme 6a), supporting the hypothesis that HAT process was enabled by amidyl radical generated from N–H bond. The mixture of **1a** and PIFA showed a weak absorption in the field ranging from 410 to 460 nm that overlapped with the emission of blue LEDs (Scheme 6b), indicating that homolysis of the N–I intermediate might be triggered from light absorption.

A proposed mechanism is depicted (Scheme 6c). Initially, the interaction of **1a** with PIFA generates the N–I(iii) complex **A** and TFA.^16^ According to the DFT calculations,16*b* the N–I(iii) complex **A** is probably generated from amide and PIFA *via* the ligand exchange on hypervalent iodine. The light-induced homolysis of N–I bond of **A** affords the amidyl radical **B** that undergoes 1,5-HAT to form the alkyl radical **C**. Meanwhile, heteroarene **2a** is pre-activated through the protonation with *in situ* generated TFA and then reacts with the nucleophilic alkyl radical **C**, leading to the intermediate **E**. Further single-electron oxidation of **E** furnishes the final product **3a**. The quantum yield (4.8%) of this reaction also suggested a photochemical process (for details, see ESI†).

## Conclusions

In summary, we have described a simple and practical approach for the regioselective heteroarylation of amides *via* unactivated C(sp^3^)–H bond functionalization. The transformation is promoted by visible-light irradiation which leads to the mild generation of amidyl radicals directly from the amide N–H bonds. A vast array of heteroarenes as well as amides including carboxamides, sulfonamides, and phosphoramides are readily functionalized. It is noteworthy that while the classic Minisci reaction usually requires the addition of extrinsic acid, this protocol takes place under neutral conditions. This metal-free process may find the potential use in medicinal chemistry in near future.

## Experimental section

### General procedure for heteroarylation of remote C(sp^3^)–H bonds

Heteroarene **2** (0.2 mmol) and amide **1** (0.6 mmol) were loaded in a reaction vial without N_2_ atmosphere. Then DCE (2.0 mL) followed by PIFA (0.46 mmol) was added to the mixture. The reaction was irradiated with 2 × 50 W blue LEDs from 5 cm away and kept at 25 °C under fan cooling. After the reaction completion monitored by TLC, the mixture was neutralized by aq. KOH until pH > 8 and then extracted with ethyl acetate (3 × 10 mL). The combined organic extracts were washed by brine, dried over Na_2_SO_4_, filtered, concentrated, and purified by flash column chromatography on silica gel (eluent: ethyl acetate/petroleum ether) to give the desired products **3–5**.

## Conflicts of interest

There are no conflicts to declare.

## Supplementary Material

Supplementary information
