# Supplementary material for: Practical, metal-free remote heteroarylation of amides via unactivated C(sp3)–H bond functionalization
Source: Chem Sci. 2019 Jun 11;10(28):6915–9. doi: 10.1039/c9sc02564b (PMC6640195; doi:10.1039/c9sc02564b)
Supplement: Supplementary file 1 [file SC-010-C9SC02564B-s001.pdf]

Electronic Supplementary Material (ESI) for Chemical Science.
This journal is © The Royal Society of Chemistry 2019

Supplementary Information

Practical, Metal-Free Remote Heteroarylation of Amides via Unactivated C(sp^3)-H Bond Functionalization

Nana Tang,[a] Xinxin Wu,[a] and Chen Zhu[a,b,]*

[a] Key Laboratory of Organic Synthesis of Jiangsu Province, College of Chemistry, Chemical Engineering and Materials Science, Soochow University, 199 Ren-Ai Road, Suzhou, Jiangsu 215123, China

[b] Key Laboratory of Synthesis Chemistry of Natural Substances, Shanghai Institute of Organic Chemistry, Chinese Academy of Science, 345 Lingling Road, Shanghai 200032, China.

Email: chzhu@suda.edu.cn

Table of Contents

1. General experimental details S2
2. Reaction conditions optimization S2
3. General procedure for the C(sp^3)-H heteroarylation of amides S3
4. Synthesis of starting materials S4
5. Characterization of new starting materials S7
6. Characterization of products S9
7. ^{1}H, ^{13}C, ^{19}F, ^{31}P NMR spectra S23
8. Absorption and emission studies S94
9. Determination of quantum yield S95

1. General experimental details

Commercially available reagents were used without further purification. Infrared (FT-IR) spectra were recorded on a BRUKER VERTEX 70, ν_{max} in cm^{-1}. ^{1}H-NMR spectra were recorded on a BRUKER AVANCE III HD (400 MHz) spectrometer. Chemical shifts are reported in ppm from tetramethylsilane with the solvent resonance as internal standard ($CDCl_3$: δ 7.26). Data are reported as follows: chemical shift, multiplicity (s = singlet, d = doublet, t = triplet, q = quadruplet, br = broad, m = multiplet), coupling constants (Hz) and integration. ^{13}C-NMR spectra were recorded on a BRUKER AVANCE III HD (100 MHz) spectrometer with complete proton decoupling. Chemical shifts are reported in ppm from tetramethylsilane with the solvent resonance as the internal standard ($CDCl_3$: δ 77.16). ^{19}F-NMR spectra were recorded on a BRUKER AVANCE III HD (376 MHz) spectrometer. Mass spectra were measured with an Agilent Technologies 6120 Quadrupole LC/MS. High resolution mass spectrometry (HRMS) were measured with a GCT Premier^{TM} and BRUKER micrOTF-Q III. Melting points were measured using INESA WRR and values are uncorrected.

2. Reaction conditions optimization

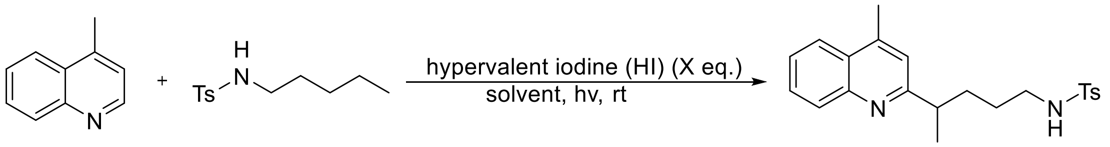

entry	HI(X equiv.)	*hv*	Amide(equiv.)	Solvent	Yield(%)
1	PIFA(2.3)	2 X 50 W blue LEDs	3.0	DCM	84%
**2**	**PIFA(2.3)**	**2 X 50 W blue LEDs**	**3.0**	**DCE**	**85%**
3	PIFA(2.3)	2 X 50 W blue LEDs	3.0	CH_3CN	57%
4	PIFA(2.3)	2 X 50 W blue LEDs	3.0	$PhCF_3$	75%
5	PIFA(2.3)	2 X 50 W blue LEDs	3.0	MeOH	51%
6	PIFA(2.3)	2 X 50 W blue LEDs	3.0	$CHCl_3$	41%
7	PIFA(2.3)	2 X 50 W blue LEDs	3.0	DMF	34%
8	PIFA(2.3)	2 X 50 W blue LEDs	3.0	DMSO	<5%
9	PIDA(2.3)	2 X 50 W blue LEDs	3.0	DCE	<5%
10	BI-OH(2.3)	2 X 50 W blue LEDs	3.0	DCE	ND
11	BI-OAc(2.3)	2 X 50 W blue LEDs	3.0	DCE	ND

12	PIFA(2.3)	In dark	3.0	DCE	ND
13	PIFA(2.3)	30 W white LEDs	3.0	DCE	79%
14	PIFA(2.3)	30 W green LEDs	3.0	DCE	ND
15	PIFA(2.3)	30 W blue LEDs	3.0	DCE	77%
16	PIFA(2.3)	In dark, 80 °C	3.0	DCE	<10%
17	PIFA(1.9)	2 X 50 W blue LEDs	3.0	DCE	68%
18	PIFA(2.1)	2 X 50 W blue LEDs	3.0	DCE	72%
19	PIFA(2.5)	2 X 50 W blue LEDs	3.0	DCE	84%
20	PIFA(2.3)	2 X 50 W blue LEDs	2.0	DCE	77%
21	PIFA(2.3)	2 X 50 W blue LEDs	2.5	DCE	80%
22	PIFA(2.3)	2 X 50 W blue LEDs	3.5	DCE	84%

3. General procedure for the C(sp^3)-H heteroarylation of amides

Heteroarene **2** (0.2 mmol) and amide **1** (0.6 mmol) were loaded in a reaction vial without N_2 atmosphere. Then DCE (2.0 mL) followed by PIFA (0.46 mmol) was added to the mixture. The reaction was irradiated with 2 x 50 W blue LEDs from 5 cm away and kept at 25 °C under fan cooling. After the reaction completion monitored by TLC, the mixture was neutralized by aq. KOH until pH > 8 and then extracted with ethyl acetate (3 x 10 mL). The combined organic extracts were washed by brine, dried over Na_2SO_4, filtered, concentrated, and purified by flash column chromatography on silica gel (eluent: ethyl acetate/ petroleum ether) to give the desired products **3-5**.

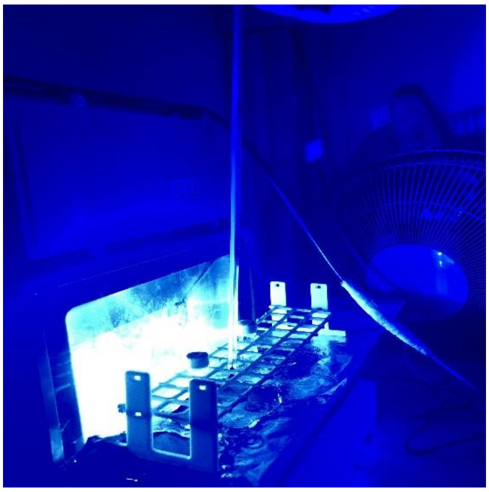

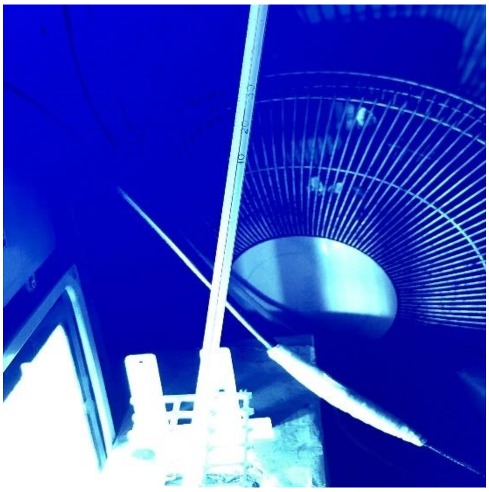

Lamp parameters: blue light, rated voltage: AC220V, rated frequency: 50/60Hz, executive standard: GB700.1-2007, GB700.202-2008, quality inspection number: QC-H02, production date: 2017.13.

4. Synthesis of starting materials

Starting materials and the references for known compounds:

	Structure		Structure
**1a**[6]	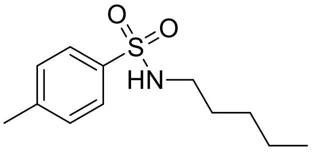	**1ah**[6]	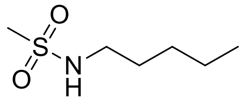
**1u**[7]	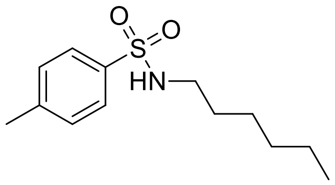	**1ai**	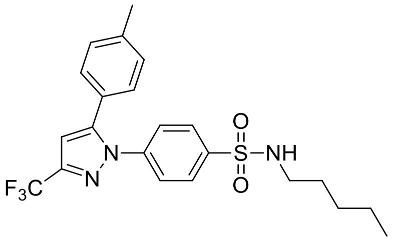
**1v**[19]	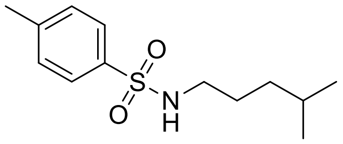	**1aj**	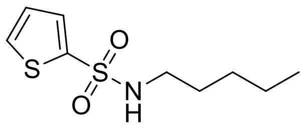
**1w**[20]	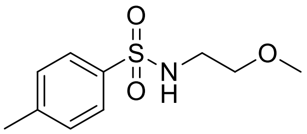	**1ak**[6]	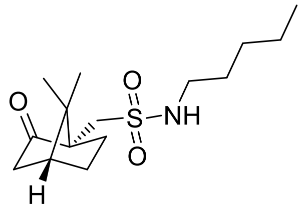
**1x**[8]	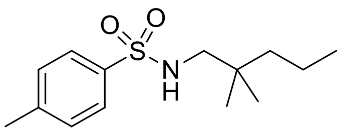	**7a**[14]	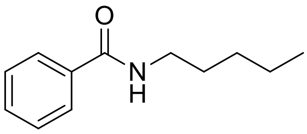
**1y**	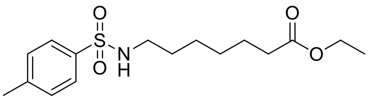	**7b**[15]	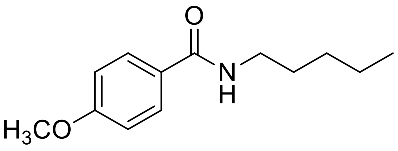
**1z**	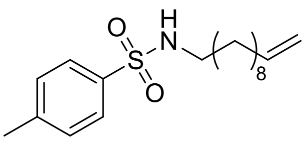	**7c**	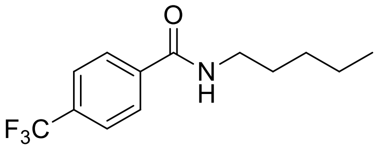

**1aa**	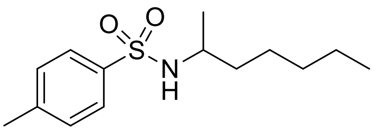	**7d**	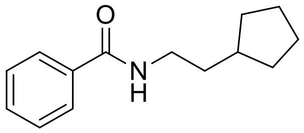
**1ab**[9]	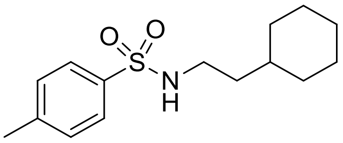	**7f**	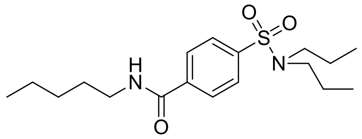
**1ac**[10]	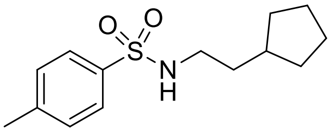	**7g**[16]	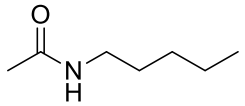
**1ad**[11]	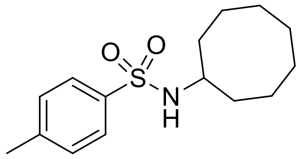	**7h**[17]	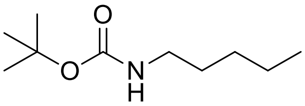
**1ae**[12]	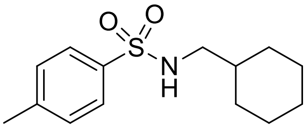	**8a**	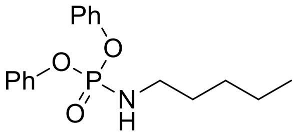
**1af**	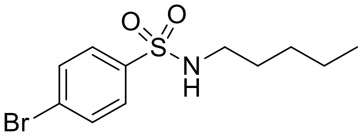	**8e**[18]	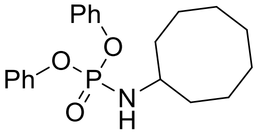
**1ag**[13]	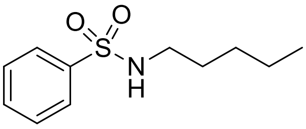	**8f**	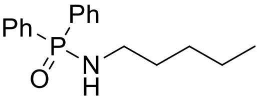

4.1 General procedure for carboxamides synthesis

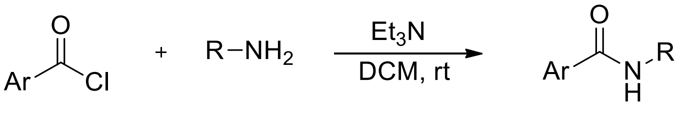

A flame-dried round-bottomed flask was charged with aroyl chloride (11 mmol, 1.1 equiv.), DCM (20 mL), Et_3N (2.9 mL, 21 mmol, 2.1 equiv.) and amine/ amine hydrochloride (10 mmol, 1 equiv.). The reaction mixture was stirred at RT for 3 h. The reaction mixture was then diluted with DCM (20 mL), washed with 1 M HCl (20 mL), water (20 mL), and brine (20 mL), and then dried over Na_2SO_4 and concentrated. The crude product was purified by flash column chromatography on silica gel (gradient 100% petroleum ether to 25% ethyl acetate/ petroleum ether) to afford the desired product **7c**, **7d**, **7f**.[1]

4.2 General procedure for sulfonamides synthesis

Method A:

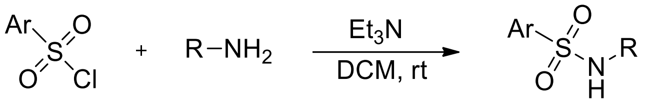

A flame-dried round-bottomed flask was charged with arylsulfony chloride (11 mmol, 1.1 equiv.), DCM (20 mL), Et_3N (2.9 mL, 21 mmol, 2.1 equiv.) and amine/ amine hydrochloride (10 mmol, 1equiv.). The reaction mixture was stirred at RT for 3 h. The reaction mixture was then diluted with DCM (20 mL), washed with 1 M HCl (20 mL), water (20 mL), and brine (20 mL), and then dried over Na_2SO_4 and concentrated. The crude product was purified by flash column chromatography on silica gel (gradient 100% petroleum ether to 25% ethyl acetate/ petroleum ether) to afford the desired product **1af, 1aj**.[2]

Method B:

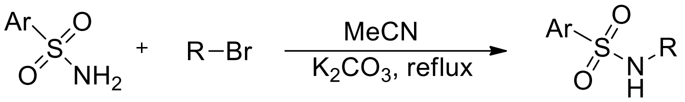

To a solution of alkyl bromide (5 mmol, 1 equiv.) in MeCN (20 mL) were added K_2CO_3 (1.38 g, 10 mmol, 2 equiv.) and $TsNH_2$ (1.171 g, 10 mmol, 2 equiv.), and the reaction was heated to reflux. After 5 h, the reaction mixture was filtrated through Celite® and concentrated in vacuo. The desired product was afforded after purification by flash column chromatography on silica gel (petroleum ether/ ethyl acetate = 90/ 10).

1y, 1z, **1ai** are new compounds which were prepared according to the known procedures.[3]

4.3 General procedure for phosphoramides synthesis

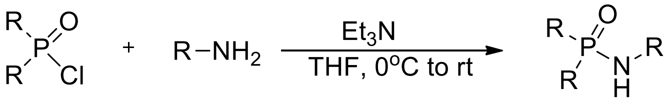

To a stirred solution of amines (10 mmol) in THF (25 mL) was added triethylamine (21 mmol) at RT. Diphenylphosphinic chloride (12 mmol) or diphenyl chlorophosphate (12 mmol) in 25 mL of THF was added to the solution at 0 °C. After being stirred for 15 min at 0 °C, the reaction solution was allowed back to RT and stirred for overnight. The resulting mixture was cooled in ice bath, and diluted with $CHCl_3$ and water. The product was extracted with $CHCl_3$ and combined organic layer was washed by brine, 1 N HCl, sat. $NaHCO_3$ and brine. The combined organic phases were dried over Na_2SO_4, filtered, and concentrated in vacuo. The crude product was purified by flash column chromatography on silica gel (CH_2Cl_2/ MeOH = 95:5) to give the corresponding phosphinic amides or phosphoramidates.

8a, **8f** are new compounds which were prepared according to the known procedures.[4]

References:

[1] T. Aubineau, J. Cossy, *Chem. Commun.*, **2013**, 49, 3303-330.

[2] T. Cochet, V. Bellosta, D. Roche, J.-Y. Ortholand, A. Greiner, J. Cossy, *Chem. Commun.*, **2012**, 48, 10745-10747.

[3] C. Bakkali-Hassani, E. Rieger, J. Vignolle, F. R. Wurm, S. Carlotti, D. Taton, *Chem. Commun.*, **2016**,52, 9719-9722.

[4] B.-J. Li, R. D. Simard, A. M. Beauchemin, *Chem. Commun.*, **2017**,53, 8667-8670.

[5] X. Xiao, C. Hou, Z. Zhang, Z. Ke, J. Lan, H. Jiang, W. Zeng, *Angew. Chem. Int. Ed.*, **2016**, *19*, 11897-11901.
[6] D. Chang, R. Zhao,C. Wei, Y. Yao,Y. Liu, L. Shi, *J. Org. Chem.*, **2018**, *83*, 3305-3315.
[7] C. Bakkali-Hassani, E. Rieger, J. Vignolle, F. R. Wurm, S. Carlotti, D. Taton, *Chem. Commun.*, **2016**, *52*, 9719-9722.
[8] D. Meng, Y. Tang, J. Wei, X. Shi, M. Yang, *Chem. Commun.*, **2017**, *53*, 5744-5747.
[9] M. Lecomte, G. Evano, *Angew. Chem. Int. Ed.*, **2016**, *24*, 4547-4551.
[10] A. Martínez-Asencio, D. J. Ramón, M. Yus, *Tetrahedron Lett.*, **2010**, *51*, 325-327.
[11] D. C. Rosenfeld, S. Shekhar, A. Takemiya, M. Utsonomiya, J. F. Hartwig, *Org. Lett.*, **2006**, *8*, 4179-4182.
[12] R. Cano, D. J. Ramón, M. Yus, *J. Org. Chem.*, **2011**, *76*, 5547-5557.
[13] W. Wei, C. Liu, D. Yang, J. Wen, J. You, H. Wang, *Adv.Synth.Cata.*, **2015**, *23*, 987-992.
[14] T. Aubineau, J. Cossy, *Chem. Commun.*, **2013**, *49*, 3303-3305.
[15] G. J. Choi, Q. Zhu, D. C. Miller, C. Gu, R. R. Knowles, *Nature*, **2016**, *539*, 268-271.
[16] M. Milan, G. Carboni, M. Salamone, M. Costas, M. Bietti, *ACS Cat*. **2017**, *7*, 5903-5911.
[17] S. Chowdhury, R. F. Standaert, *J. Org. Chem.* **201**6, *81*, 9957-9963.
[18] A. K. Gupta, D. K .Dubey, M. Sharma, M. P. Kaushik, *Organic Preparations and Procedures International*, **2007**, *39*, 297-305.
[19] M. Zhu, K. Fujita, R. Yamaguchi, *Org. Lett.*, **2010**, *12*, 1336-1339.
[20] M. Zhu, W. Wei, D. Yang, H. Cui, L. Wang, G. Menga and H. Wang. *Org. Biomol. Chem.*, **2017**, *15*, 4789-4793.

5. Characterization of new starting materials

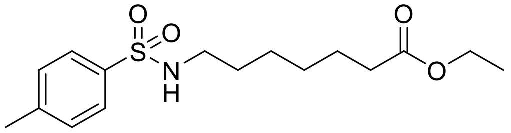

1y: colorless oil. ^{1}H NMR (400 MHz, $CDCl_3$) δ 7.74-7.70 (m, 2H), 7.29-7.24 (m, 2H), 5.03 (t, *J* = 6.0 Hz, 1H), 4.07 (q, *J* = 7.2 Hz, 2H), 2.90-2.83 (m, 2H), 2.39 (s, 3H), 2.20 (t, *J* = 7.6 Hz, 2H), 1.56-1.46 (m, 2H), 1.46-1.37 (m, 2H), 1.28-1.14 (m, 7H); ^{13}C NMR (100 MHz, $CDCl_3$) δ 173.7, 143.2, 137.0, 129.6, 127.0, 60.2, 43.0, 34.1, 29.2, 28.5, 26.1, 24.7, 21.4, 14.2. FT-IR: ν (cm^{-1}) 2936, 2862, 1732, 1652, 1599, 1420, 1398, 1327. HRMS [ESI] calcd for $C_{16}H_{26}NO_4S$ $[M+H]^+$ 328.1577, found 328.1585.

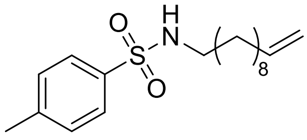

1z: white solid, m.p. 53-54 °C. ^{1}H NMR (400 MHz, $CDCl_3$) δ 7.77-7.74 (m, 2H), 7.31-7 .27(m, 2H), 5.84-5.73 (m, 1H), 5.00-4.87 (m, 3H), 2.89 (q, *J* = 6.8 Hz, 2H), 2.41 (s, 3H), 2.04-1.97 (m, 2H), 1.47-1.29 (m, 4H), 1.26-1.15 (m, 10H). ^{13}C NMR (100 MHz, $CDCl_3$) δ 143.2, 139.1, 137.0, 129.6, 127.1, 114.1, 43.2, 33.8, 29.5, 29.4, 29.3, 29.1, 29.0, 28.9, 26.5, 21.5. FT-IR: ν (cm^{-1}) 3293, 2917, 2850, 2340, 1643, 1596, 1467, 1384, 1290. HRMS [ESI] calcd for $C_{18}H_{29}NNaO_2S$ $[M+Na]^+$ 346.1811, found 346.1803.

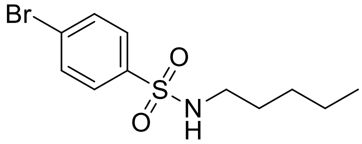

1af: yellow solid, m.p. 59-60 °C.. ^{1}H NMR (400 MHz, $CDCl_3$) δ 7.75-7.71 (m, 2H), 7.67-7.62 (m, 2H), 4.85 (t, *J* = 6.0 Hz, 1H), 2.92 (q, *J* = 6.8 Hz, 2H), 1.49-1.40 (m, 2H), 1.28-1.18 (m, 4H),

0.83 (t, J = 6.4 Hz, 3H); ^{13}C NMR (100 MHz, $CDCl_3$) δ 139.1, 132.4, 128.6, 127.5, 43.3, 29.2, 28.6, 22.1, 13.9. FT-IR: ν (cm-1) 3247, 3083, 2954, 2860, 1574, 1437, 1391, 1298. HRMS [ESI] calcd for $C_{11}H_{16}BrNNaO_2S$ $[M+Na]^+$ 327.9977, found 327.9963.

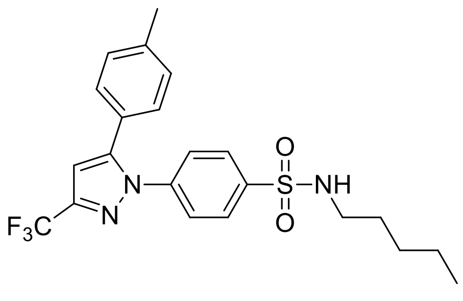

1ai: white solid, m.p. 167-168 °C. ^{1}H NMR (400 MHz, $CDCl_3$) δ 7.88-7.82 (m, 2H), 7.49-7.43 (m, 2H), 7.19-7.17 (m, 4H), 6.74 (s, 1H), 5.10 (br, 1H), 2.96-2.88 (m, 2H), 2.36 (s, 3H), 1.50-1.40 (m, 2H), 1.28-1.20 (m, 4H), 0.84 (t, J = 6.0 Hz, 3H), ^{13}C NMR (100 MHz, $CDCl_3$) δ 151.3, 144.0 (q, $J_{C\text{-}F}$ = 38.2 Hz), 142.4, 139.8, 139.6, 129.7, 128.7, 128.1, 125.7, 125.6, 121.1 (q, $J_{C\text{-}F}$ = 267.5 Hz), 106.2, 43.3, 29.2, 28.6, 22.1, 21.3, 13.9. ^{19}F NMR (376 MHz, $CDCl_3$) δ -62.4 (s). FT-IR: ν (cm-1) 3290, 3066, 2962, 2918, 2849, 1601, 1561, 1471, 1374, 1269. HRMS [ESI] calcd for $C_{22}H_{24}F_3N_3NaO_2S$ $[M+Na]^+$ 474.1434, found 474.1429.

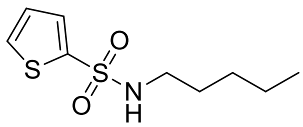

1aj: yellow oil. ^{1}H NMR (400 MHz, $CDCl_3$) δ 7.55-7.53 (m, 1H), 7.52-7.50 (m, 1H), 6.99 (dd, J = 4.8, 3.6 Hz, 1H), 5.45 (t, J = 6.0 Hz, 1H), 2.91 (q, J = 6.8 Hz, 2H), 1.45-1.35 (m, 2H), 1.21-1.09 (m, 4H), 0.77-0.71 (m, 3H); ^{13}C NMR (100 MHz, $CDCl_3$) δ 140.8, 131.9, 131.8, 127.5, 43.4, 28.9, 28.6, 22.1, 13.9. FT-IR: ν (cm^{-1}) 3283, 3107, 2957, 2870, 1508, 1465, 1379, 1226. HRMS [ESI] calcd for $C_9H_{16}NO_2S_2$ $[M+Na]^+$ 234.0617, found 234.0617.

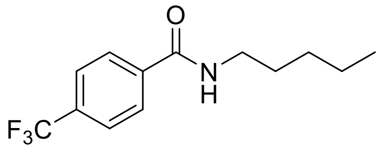

7c: white solid, m.p. 84-85 °C. ^{1}H NMR (400 MHz, $CDCl_3$) δ 7.88-7.83 (m, 2H), 7.67-7.62 (m, 2H), 6.52 (br, 1H), 3.46-3.39 (m, 2H), 1.65-1.56 (m, 2H), 1.38-1.28 (m, 4H), 0.89 (t, J = 6.8 Hz, 3H); ^{13}C NMR (100 MHz, $CDCl_3$) δ 166.5, 138.1, 133.0 (q, $J_{C\text{-}F}$ = 32.6 Hz), 127.4, 125.4 (q, $J_{C\text{-}F}$ = 3.1 Hz), 123.7 (q, $J_{C\text{-}F}$ = 270.9 Hz), 40.3, 29.2, 29.1, 22.3, 13.9; ^{19}F NMR (376 MHz, $CDCl_3$) δ -63.0 (s). FT-IR: ν (cm^{-1}) 3306, 2953, 2934, 2872, 1631, 1579, 1477, 1374, 1289. HRMS [ESI] calcd for $C_{13}H_{16}F_3NNaO$ $[M+Na]^+$ 282.1076, found 282.1082.

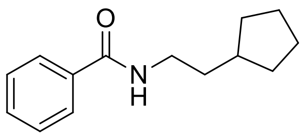

7d: white solid, m.p. 57-58 °C. ^{1}H NMR (400 MHz, $CDCl_3$) δ 7.80-7.75 (m, 2H), 7.50-7.43 (m, 1H), 7.42-7.35 (m, 2H), 6.56 (br, 1H), 3.48-3.40 (m, 2H), 1.88-1.74 (m, 3H), 1.66-1.45 (m, 6H), 1.18-1.06 (m, 2H); ^{13}C NMR (100 MHz, $CDCl_3$) δ 167.6, 134.8, 131.2, 128.5, 126.9, 39.6, 37.9, 35.9, 32.6, 25.1. FT-IR: ν (cm^{-1}) 3301, 3062, 2944, 2854, 1702, 1633, 1577, 1488, 1359, 1293. HRMS [ESI] calcd for $C_{14}H_{19}NNaO$ $[M+H]^+$ 240.1359, found 240.1354.

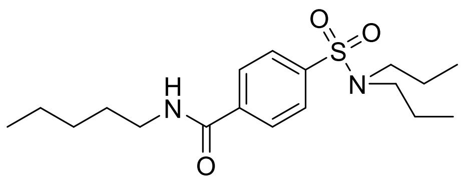

7f: yellow solid, m.p. 100-101 °C. ^{1}H NMR (400 MHz, $CDCl_3$) δ 7.87-7.82 (m, 2H), 7.80-7.74 (m, 2H), 6.58-6.45 (br, 1H), 3.47-3.39 (m, 2H), 3.08-3.02 (m, 4H), 1.67-1.57 (m, 2H), 1.57-1.46 (m, 4H), 1.38-1.31

(m, 4H), 0.93-0.87 (m, 3H), 0.85 (t, J = 7.2 Hz, 6H); ^{13}C NMR (100 MHz, $CDCl_3$) δ 166.3, 142.2, 138.6, 127.8, 126.9, 50.0, 40.3, 29.1, 22.3, 21.9, 13.9, 11.1. FT-IR: ν (cm^{-1}) 3306, 2964, 2928, 2874, 1633, 1552, 1467, 1372, 1296. HRMS [ESI] calcd for $C_{18}H_{31}N_2O_3P$ $[M+Na]^+$ 355.2050, found 355.2053.

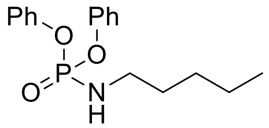

8a: colourless oil. ^{1}H NMR (400 MHz, $CDCl_3$) δ 7.32-7.25 (m, 8H), 7.16-7.09 (m, 2H), 4.50-4.35 (m, 1H), 3.04-2.94 (m, 2H), 1.45-1.36 (m, 2H), 1.26-1.15 (m, 4H), 0.82 (t, J = 6.8 Hz, 3H); ^{13}C NMR (100 MHz, $CDCl_3$) δ 151.0 (d, J_{C-P} = 6.5 Hz), 129.6, 124.7, 120.2 (d, J_{C-P} = 5.0Hz), 41.7, 31.0 (d, J_{C-P} = 6.2 Hz), 28.7, 22.3, 14.0; ^{31}P NMR (161 MHz, $CDCl_3$) δ 0.11 (s). FT-IR: ν (cm^{-1}) 3223, 3069, 2956, 2871, 1590, 1455, 1378, 1220. HRMS [ESI] calcd for $C_{17}H_{22}NNaO_3P$ $[M+Na]^+$ 342.1230, found 342.1224.

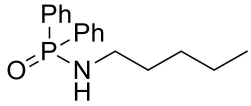

8f: colourless oil. ^{1}H NMR (400 MHz, $CDCl_3$) δ 7.89-7.82 (m, 4H), 7.46-7.34 (m, 6H), 2.98-2.85 (m, 3H), 1.57-1.48 (m, 2H), 1.28-1.18 (m, 4H), 0.82 (t, J = 7.2 Hz, 3H); ^{13}C NMR (100 MHz, $CDCl_3$) δ 132.6 (d, J_{C-P} = 128.2 Hz), 132.1 (d, J_{C-P} = 9.3 Hz), 131.7 (d, J_{C-P} = 2.7 Hz), 128.5 (d, J_{C-P} = 12.5 Hz), 40.8 (d, J_{C-P} = 1.7 Hz), 31.8 (d, J_{C-P} = 7.1 Hz), 28.9, 22.3, 14.0; ^{31}P NMR (161 MHz, $CDCl_3$) δ 23.5 (s). FT-IR: ν (cm^{-1}) 3224, 3123, 3026, 2954, 2867, 1456, 1438, 1377, 1186. HRMS [ESI] calcd for $C_{17}H_{23}NOP$ $[M+Na]^+$ 288.1512, found 288.1520.

6. Characterization of products

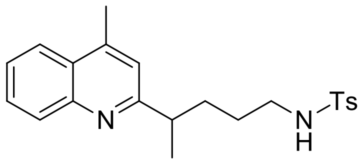

3a: yellow oil. ^{1}H NMR (400 MHz, $CDCl_3$) δ 8.12-8.08 (m, 1H), 7.94 (dd, J = 8.4, 0.8 Hz, 1H), 7.71-7.65 (m, 3H), 7.52 (ddd, J = 8.0, 6.8, 0.8 Hz, 1H), 7.20-7.17 (m, 2H), 7.08 (s, 1H), 5.63-5.55 (m, 1H), 3.02-2.82 (m, 3H), 2.67 (s, 3H), 2.36 (s, 3H), 1.88-1.77 (m, 1H), 1.68-1.57 (m, 1H), 1.50-1.32 (m, 2H), 1.28 (d, J = 7.2 Hz, 3H); ^{13}C NMR (100 MHz, $CDCl_3$) δ 165.2, 146.8, 144.4, 142.5, 136.6, 129.1, 128.8, 128.7, 126.6, 125.2, 123.1, 119.8, 42.6, 41.3, 33.2, 26.7, 21.0, 20.5, 18.4. FT-IR: ν (cm^{-1}) 3069, 2961, 2928, 2869, 2855, 1604, 1509, 1455, 1381, 1264. HRMS [ESI] calcd for $C_{22}H_{27}N_2O_2S$ $[M+H]^+$ 383.1788, found 383.1803.

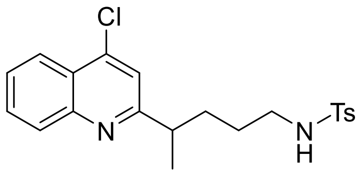

3b: yellow oil. ^{1}H NMR (400 MHz, $CDCl_3$) δ 8.18-8.13(m, 1H), 8.10-8.05 (m, 1H), 7.76-7.70 (m, 1H), 7.70-7.65 (m, 2H), 7.61-7.55 (m, 1H), 7.32 (s, 1H), 7.21 - 7.17 (m, 2H), 5.51-5.40 (m, 1H), 3.03-2.83 (m, 3H), 2.34 (s, 3H), 1.86-1.74 (m, 1H), 1.68-1.57 (m, 1H), 1.51-1.31 (m, 2H), 1.27 (d, J = 7.2 Hz, 3H); ^{13}C NMR (100 MHz, $CDCl_3$) δ 166.0, 148.3, 143.2, 143.1, 137.0, 130.5, 129.6, 129.2, 127.0, 127.0, 125.1, 123.9, 119.8, 43.1, 41.8, 33.5, 27.2, 21.5, 20.9. FT-IR: ν (cm^{-1})3282, 3067, 2964, 2927, 2870, 1615, 1553, 1455, 1378, 1219. HRMS [ESI] calcd for $C_{21}H_{24}ClN_2O_2S$ $[M+H]^+$ 403.1242, found 403.1247.

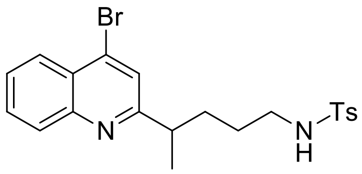

3c: yellow oil, ^{1}H NMR (400 MHz, $CDCl_3$) δ 8.11-8.07 (m, 1H), 8.04-8.00 (m, 1H), 7.72-7.65 (m, 3H), 7.55 (ddd, *J* = 8.0, 6.8, 1.2 Hz, 1H), 7.51 (s, 1H), 7.19-7.15 (m, 2H), 5.54 (t, *J* = 5.6 Hz, 1H), 2.99-2.83 (m, 3H), 2.32 (s, 3H), 1.84-1.73 (m, 1H), 1.66-1.56 (m, 1H), 1.51-1.28 (m, 2H), 1.26 (d, *J* = 6.8 Hz, 3H); ^{13}C NMR (100 MHz, $CDCl_3$) δ 166.0, 148.2, 143.1, 137.0, 134.5, 130.4, 129.6, 129.3, 127.1, 127.0, 126.6, 126.5, 123.7, 43.1, 41.7, 33.5, 27.2, 21.5, 20.8. FT-IR: ν (cm^{-1}) 3279, 3064, 2962, 2850, 1614, 1551, 1454, 1378, 1214. HRMS [ESI] calcd for $C_{21}H_{24}BrN_2O_2S$ $[M+H]^+$ 447.0736, found 447.0744.

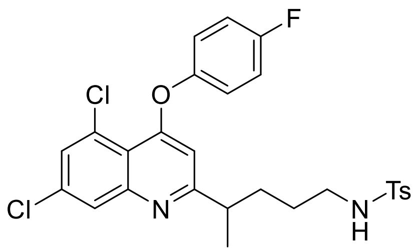

3d: white solid, m.p. 122-123 °C. ^{1}H NMR (400 MHz, $CDCl_3$) δ 7.98-7.95 (m, 1H), 7.69-7.64 (m, 2H), 7.53-7.51 (m, 1H), 7.25-7.21 (m, 2H), 7.19-7.12 (m, 2H), 7.12-7.07 (m, 2H), 6.44 (s, 1H), 5.07-4.99 (m, 1H), 2.93-2.74 (m, 3H), 2.39 (s, 3H), 1.76-1.64 (m, 1H), 1.58-1.46 (m, 1H), 1.46-1.27 (m, 2H), 1.15 (d, *J* = 6.8 Hz, 3H). ^{13}C NMR (100 MHz, $CDCl_3$) δ 168.8, 162.7, 160.0 (d, J_{C-F} = 243.1 Hz), 151.2, 150.1 (d, J_{C-F} = 2.7 Hz), 143.3, 137.0, 135.0, 130.0, 129.6, 128.9, 127.4, 127.0, 122.0 (d, J_{C-F} = 8.3 Hz), 117.1 (d, J_{C-F} = 23.3 Hz), 117.0, 105.6, 43.0, 41.8, 33.1, 27.3, 21.5, 20.7. ^{19}F NMR (376 MHz, $CDCl_3$) δ -117.0 (s). FT-IR: ν (cm^{-1}) 3282, 3074, 2961, 1598, 1551, 1455, 1364, 1211. HRMS [ESI] calcd for $C_{27}H_{26}Cl_2FN_2O_3S$ $[M+Na]^+$ 547.1020, found 547.1015.

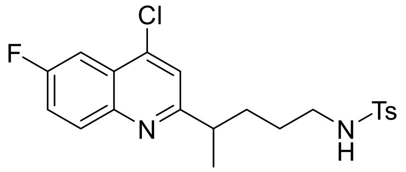

3e: yellow oil. ^{1}H NMR (400 MHz, $CDCl_3$) δ 8.08-8.03 (m, 1H), 7.78-7.73 (m, 1H), 7.71-7.65 (m, 2H), 7.51-7.45 (m, 1H), 7.33 (s, 1H), 7.24-7.19 (m, 2H), 5.36 (t, *J* = 6.0 Hz, 1H), 3.00-2.84 (m, 3H), 2.36 (s, 3H), 1.85-1.74 (m, 1H), 1.68-1.56 (m, 1H), 1.51-1.30 (m, 2H), 1.27 (d, *J* = 7.2 Hz, 3H). ^{13}C NMR (100 MHz, $CDCl_3$) δ 165.3 (d, J_{C-F} = 2.8 Hz), 160.9 (d, J_{C-F} = 247.2 Hz), 145.4, 143.2, 142.2 (d, J_{C-F} = 5.5 Hz), 137.0, 131.9 (d, J_{C-F} = 9.1 Hz), 129.6, 127.1, 126.0 (d, J_{C-F} = 10.1 Hz), 120.6, 120.6 (d, J_{C-F} = 25.4 Hz), 107.7 (d, J_{C-F} = 24.4 Hz), 43.1, 41.7, 33.5, 27.2, 21.5, 20.8. ^{19}F NMR (376 MHz, $CDCl_3$) δ - 112.0 (s). FT-IR: ν (cm^{-1}) 3281, 2931, 2870, 1626, 1559, 1456, 1373, 1228. HRMS [ESI] calcd for $C_{21}H_{23}ClFN_2O_2S$ $[M+Na]^+$ 421.1147, found 421.11150.

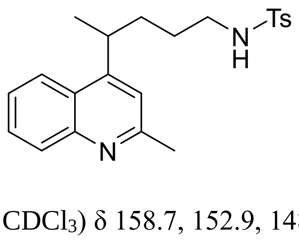

3f: yellow oil. ^{1}H NMR (400 MHz, $CDCl_3$) δ 8.04-7.99 (m, 1H), 7.98-7.93 (m, 1H), 7.70-7.61(m, 3H), 7.50-7.44 (m, 1H), 7.25-7.20 (m, 2H), 7.09 (s, 1H), 4.80-4.60 (m, 1H), 3.54-3.43 (m, 1H), 2.95-2.86 (m, 2H), 2.69 (s, 3H), 2.38 (s, 3H), 1.81-1.62 (m, 2H), 1.54-1.35 (m, 2H), 1.30 (d, *J* = 6.8 Hz, 3H); ^{13}C NMR (100 MHz, $CDCl_3$) δ 158.7, 152.9, 148.0, 143.3, 136.9, 129.6, 129.3, 128.9, 127.0, 125.5, 125.3, 122.7, 118.4, 43.1, 33.9, 32.8, 27.5, 25.3, 21.5, 21.2. FT-IR: ν (cm^{-1}) 3281, 3062, 2965, 2870, 1598, 1510, 1455, 1379, 1184. HRMS [ESI] calcd for $C_{22}H_{27}N_2O_2S$ $[M+H]^+$ 383.1788, found 383.1795.

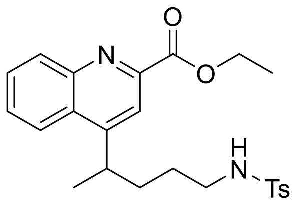

3g: yellow oil. ^{1}H NMR (400 MHz, $CDCl_3$) δ 8.30-8.25 (m, 1H), 8.06-8.01 (m, 1H), 7.90-7.96 (m, 1H), 7.75-7.68 (m, 1H), 7.68-7.63 (m, 2H), 7.63-7.57 (m, 1H), 7.22-7.15 (m, 2H), 5.08-4.92 (m, 1H), 4.56-4.47 (m, 2H), 3.58-3.50 (m, 1H), 2.93-2.84 (m, 2H), 2.34 (s, 3H), 1.83-1.64 (m, 2H), 1.54-1.35 (m, 5H), 1.34-1.28 (m, 3H); ^{13}C NMR (100 MHz, $CDCl_3$) δ 165.3, 154.1, 147.5, 147.4, 142.9, 136.3, 131.2, 129.2, 129.1, 127.9, 127.6, 126.5, 122.3, 116.9, 76.9, 42.6, 33.4, 32.7, 27.0, 21.0, 20.6, 13.9. FT-IR: ν (cm^{-1}) 3283, 2965, 2929, 2869, 1718, 1590, 1459, 1395, 1262. HRMS [ESI] calcd for $C_{24}H_{29}N_2O_4S$ [M+H]$^+$ 441.1843, found 441.1852.

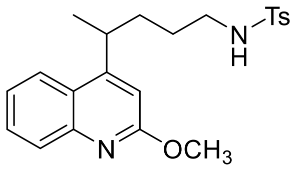

3h: yellow oil. ^{1}H NMR (400 MHz, $CDCl_3$) δ 7.89-7.84 (m, 2H), 7.71-7.66 (m, 2H), 7.62-7.56 (m, 1H), 7.39-7.33 (m, 1H), 7.24-7.22 (m, 2H), 6.70 (s, 1H), 5.00-4.74 (m, 1H), 4.05 (s, 3H), 3.45-3.34 (m 1H), 2.93-2.85 (m, 2H), 2.38 (s, 3H), 1.76-1.55 (m, 2H), 1.52-1.34 (m, 2H), 1.28-1.24 (m, 3H). ^{13}C NMR (100 MHz, $CDCl_3$) δ 162.5, 155.5, 147.2, 143.4, 136.9, 129.7, 129.1, 128.2, 127.0, 124.1, 123.9, 122.9, 109.1, 53.2, 43.2, 33.7, 32.9, 27.4, 21.5, 21.0. FT-IR: ν (cm^{-1}) 3283, 2968, 2934, 2874, 1697, 1552, 1420, 1388, 1249. HRMS [ESI] calcd for $C_{22}H_{26}N_2NaO_3S$ [M+Na]$^+$ 421.1556, found 421.1556.

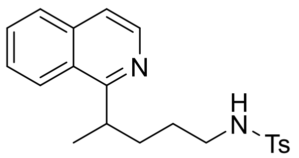

3i: yellow oil. ^{1}H NMR (400 MHz, $CDCl_3$) δ 8.48-8.45 (m, 1H), 8.17-8.13 (m, 1H), 7.84-7.80 (m, 1H), 7.71 - 7.64 (m, 3H), 7.59 (ddd, *J* = 8.4, 7.2, 1.2 Hz, 1H), 7.52-7.48 (m, 1H), 7.25-7.21 (m, 2H), 5.08 (t, *J* = 5.2 Hz, 1H), 3.78-3.68 (m, 1H), 2.93-2.85(m, 2H), 2.38 (s, 3H), 2.10-1.99 (m, 1H), 1.76-1.65 (m, 1H), 1.58-1.45 (m, 1H),1.39-1.27(m, 1H), 1.32 (d, *J* = 6.8 Hz, 3H); ^{13}C NMR (100 MHz, $CDCl_3$) δ 164.3, 142.7, 140.9, 136.5, 136.0, 129.5, 129.1, 127.2, 126.8, 126.6, 126.2, 124.2, 118.9, 42.7, 35.3, 31.9, 27.0, 21.0, 20.9. FT-IR: ν (cm^{-1}) 3280, 3053, 2958, 2919, 2850, 1622, 1560, 1455, 1378, 1260. HRMS [ESI] calcd for $C_{21}H_{25}N_2O_2S$ [M+H]$^+$ 369.1631, found 369.1656.

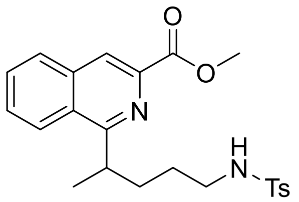

3j: yellow solid, m.p. 134-135 °C. ^{1}H NMR (400 MHz, $CDCl_3$) δ 8.35 (s, 1H), 8.20-8.14 (m, 1H), 7.93-7.87(m, 1H), 7.73-7.64 (m, 4H), 7.20-7.14 (m, 2H), 5.43 (s, 1H), 4.02 (s, 3H), 3.73-3.62 (m, 1H), 2.98-2.88 (m, 1H), 2.83-2.73 (m, 1H), 2.33 (s, 3H), 2.29-2.19 (m, 1H), 1.72-1.51 (m, 2H), 1.41-1.32 (m, 1H), 1.29 (d, *J* = 7.2 Hz, 3H); ^{13}C NMR (100 MHz, $CDCl_3$) δ 166.7, 165.3, 142.9, 140.2, 137.3, 136.0, 130.4, 129.5, 129.4, 129.1, 128.0, 127.0, 124.8, 122.6, 52.8, 43.0, 36.5, 31.3, 27.7, 21.6, 21.5. FT-IR: ν (cm^{-1}) 3242, 3080, 2975, 2868, 2849, 1711, 1500, 1424, 1318, 1264. HRMS [ESI] calcd for $C_{23}H_{26}N_2NaO_4S$ [M+Na]$^+$ 449.1505, found 449.1499.

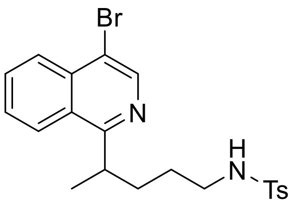

3k: yellow solid, m.p. 126-127 °C. ^{1}H NMR (400 MHz, $CDCl_3$) δ 8.63 (s, 1H), 8.17 (dd, *J* = 15.2, 8.4 Hz, 2H), 7.78 (t, *J* = 7.7 Hz, 1H), 7.70-7.62 (m, 3H), 7.25-7.20 (m, 2H), 4.92-4.82 (m, 1H), 3.74-3.64 (m, 1H), 2.89 (q, *J* = 6.6 Hz, 2H), 2.38 (s, 3H), 2.07-1.96 (m, 1H), 1.74-1 .63 (m, 1H), 1.55-1.42 (m, 1H),

1.40-1.32 (m, 1H) 1.30 (d, *J* = 6.8Hz, 3H); ^{13}C NMR (100 MHz, $CDCl_3$) δ 164.5, 143.3, 143.2, 136.9, 134.9, 131.1, 129.6, 128.1, 128.0, 127.0, 126.9, 125.0, 117.8, 43.2, 35.7, 32.5, 27.5, 21.5, 21.2. FT-IR: ν (cm^{-1}) 3275, 3069, 3046, 2961, 2924, 2852, 1615, 1566, 1450, 1388, 1241. HRMS [ESI] calcd for $C_{21}H_{24}Br_3N_2O_2S$ $[M+H]^+$ 447.0736, found 447.0760.

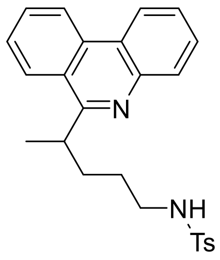

3l: yellow solid, m.p. 47-48 ^oC. ^{1}H NMR (400 MHz, $CDCl_3$) δ 8.65-8.61 (m, 1H), 8.55-8.10 (m, 1H), 8.25-8.17 (m, 2H), 7.83-7.77 (m, 1H), 7.76-7.70 (m, 1H), 7.70-7.59 (m, 4H), 7.15-7.09 (m, 2H), 5.30 (t, *J* = 5.6 Hz, 1H), 3.80-3.69 (m, 1H), 3.00-2.82 (m, 2H), 2.30 (s, 3H), 2.29-2.19 (m, 1H), 1.77-1.66 (m, 1H), 1.64-1.51 (m, 1H), 1.47-1.38 (m, 1H), 1.36 (d, *J* = 6.8 Hz, 3H); ^{13}C NMR (100 MHz, $CDCl_3$) δ 164.1, 143.1, 142.6, 136.5, 132.6, 129.8, 129.3, 129.0, 128.2, 126.9, 126.5, 126.0, 125.1, 124.5, 122.9, 122.1, 121.4, 42.7, 35.8, 31.1, 27.0, 21.0, 20.9. FT-IR: ν (cm^{-1}) 3268, 3069, 2966, 2866, 2225, 1911, 1610, 1573, 1486, 1380, 1265. HRMS [ESI] calcd for $C_{25}H_{27}N_2O_2S$ $[M+H]^+$ 419.1788, found 419.1792.

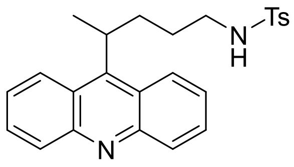

3m: yellow oil. ^{1}H NMR (400 MHz, $CDCl_3$) δ 8.40-8.22 (m, 2H), 8.21-8.15 (m, 2H), 7.72-7.64 (m, 2H), 7.62-7.58 (m, 2H), 7.50-7.40 (m, 2H), 7.15-7.11 (m, 2H), 5.18-5.08 (m, 1H), 4.26-4.15 (m, 1H), 2.88-2.78 (m, 2H), 2.33 (s, 3H), 2.22-2.06 (m, 2H), 1.62 (d, *J* = 7.2 Hz, 3H), 1.53-1.41 (m, 1H), 1.22-1.09 (m, 1H); ^{13}C NMR (100 MHz, $CDCl_3$) δ 150.2, 148.6, 143.3, 136.9, 130.9, 130.4, 129.6, 126.9, 125.9, 124.7, 123.5, 43.0, 34.0, 33.7, 28.7, 21.5, 21.2. FT-IR: ν (cm^{-1}) 3283, 3066, 3048, 2960, 2960, 2851, 1599, 1457, 1379, 1289. HRMS [ESI] calcd for $C_{25}H_{27}N_2O_2S$ $[M+H]^+$ 419.1788, found 419.1777.

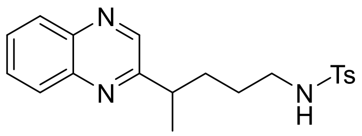

3n: yellow oil. ^{1}H NMR (400 MHz, $CDCl_3$) δ 8.67 (s, 1H), 8.08-8.03 (m, 2H), 7.77-7.65 (m, 4H), 7.23-7.18 (m, 2H), 5.09-4.99 (m, 1H), 3.12-3.02 (m, 1H), 2.98-2.88 (m, 2H), 2.36 (s, 3H), 1.94-1.83 (m, 1H), 1.75-1.64 (m, 1H), 1.57-1.44 (m, 1H), 1.41-1.30 (m, 4H), 1.34 (d, *J* = 7.2 Hz, 3H); ^{13}C NMR (100 MHz, $CDCl_3$) δ 160.4, 145.0, 143.3, 142.0, 141.4, 136.9, 130.1, 129.6, 129.2, 129.1, 129.0, 127.0, 43.1, 39.7, 32.9, 27.4, 21.5, 20.6. FT-IR: ν (cm^{-1}) 3358, 3287, 3182, 3066, 2958, 2919, 2850, 1658, 1598, 1469, 1368, 1323, 1156. HRMS [ESI] calcd for $C_{20}H_{24}N_3O_2S$ $[M+H]^+$ 370.1584, found 370.1593.

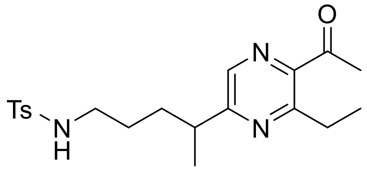

3o: yellow oil. ^{1}H NMR (400 MHz, $CDCl_3$) δ 8.22 (s, 1H), 7.73-7.69 (m, 2H), 7.29-7.24 (m, 2H), 4.94-4.79 (br, 1H), 3.10 (q, *J* = 7.6 Hz, 2H), 2.94-2.83 (m, 3H), 2.66 (s, 3H), 2.40 (s, 3H), 1.81-1.70 (m, 1H), 1.66-1.55 (m, 1H), 1.51-1.38 (m, 1H), 1.38-1.27 (m, 1H), 1.26-1.20 (m, 6H). ^{13}C NMR (100 MHz, $CDCl_3$) δ 201.3, 162.5, 158.0, 144.5, 143.4, 139.1, 136.9, 129.7, 127.1, 43.1, 39.0, 33.1, 28.8, 28.1, 27.4, 21.5, 20.3, 13.1. FT-IR: ν (cm^{-1}) 3280, 3182, 3064, 2850, 2850, 1647, 1573, 1494, 1378, 1243. HRMS [ESI] calcd for $C_{20}H_{28}$N3O3S $[M+H]^+$390.1846, found 390.1857.

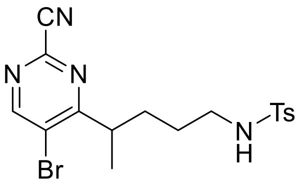

3p: white solid, m.p. 84-85 °C. ^{1}H NMR (400 MHz, $CDCl_3$) δ 8.78 (s, 1H), 7.73-7.69 (m, 2H), 7.31-7.27 (m, 2H), 4.88-4.80 (m, 1H), 3.37-3.25 (m, 1H), 2.90 (q, *J* = 6.8 Hz, 2H), 2.42 (s, 3H), 1.84-1.72 (m, 1H), 1.63-1.52 (m, 1H), 1.52-1.39 (m, 1H), 1.38-1.27 (m, 1H), 1.19 (d, *J* = 6.8 Hz, 3H); ^{13}C NMR (100 MHz, $CDCl_3$) δ 174.2, 159.6, 143.5, 143.1, 136.9, 129.8, 127.1, 124.4, 115.3, 43.0, 38.8, 31.7, 27.4, 21.5, 18.9. FT-IR: ν (cm^{-1}) 3282, 2933, 2870, 2358, 2254, 1598, 1495, 1378, 1257. HRMS [ESI] calcd for $C_{17}H_{20}BrN_4O_2S$ $[M+H]^+$ 423.0485, found 423.0478.

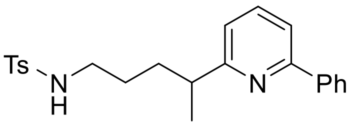

3q-1: yellow oil. ^{1}H NMR (400 MHz, $CDCl_3$) δ 8.03-7.98 (m, 2H), 7.69-7.65 (m, 2H), 7.65-7.61 (m, 1H), 7.55-7.51 (m, 1H), 7.49-7.43 (m, 2H), 7.43-7.37 (m, 1H), 7.25-7.21 (m, 2H), 7.03-6.99 (m, 1H), 4.83-4.77 (m, 1H), 2.96-2.87 (m, 3H), 2.38 (s, 3H), 1.86-1.75 (m, 1H), 1.66-1.55 (m, 1H), 1.52-1.32 (m, 2H), 1.27 (d, *J* = 6.8 Hz, 3H); ^{13}C NMR (10 MHz, $CDCl_3$) δ 165.5, 156.4, 143.2, 139.6, 137.1, 137.0, 129.6, 128.8, 128.7, 127.1, 127.0, 119.9, 117.9, 43.3, 41.3, 33.9, 27.5, 21.5, 21.0. FT-IR: ν (cm^{-1}) 3277, 3062, 2962, 2868, 1597, 1509, 1435, 1379, 1261. HRMS [ESI] calcd for $C_{23}H_{27}N_2O_2S$ $[M+H]^+$ 395.1786, found 395.1788.

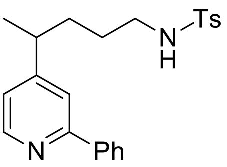

3q-2: yellow oil. ^{1}H NMR (400 MHz, $CDCl_3$) δ 8.57-8.34 (m, 1H), 7.96-7.10 (m, 2H), 7.72-7.66 (m, 2H), 7.48-7.36 (m, 4H), 7.25-7.20 (m, 2H), 6.99-6.95 (m, 1H), 4.96-4.92 (m, 1H), 2.92-2.85 (m, 1H), 2.71-2.61 (m, 1H), 2.37 (s, 3H), 1.62-1.54 (m, 2H), 1.47-1.27 (m, 2H), 1.22 (d, *J* = 7.2 Hz, 3H); ^{13}C NMR (100 MHz, $CDCl_3$) δ 157.5, 156.9, 149.5, 143.4, 139.2, 136.9, 129.7, 129.0, 128.7, 127.0, 127.0, 120.9, 119.6, 43.1, 39.3, 34.3, 27.6, 21.5. FT-IR: ν (cm^{-1}) 3356, 3181, 3061, 2958, 2850, 1658, 1598, 1470, 1378, 1156. HRMS [ESI] calcd for $C_{23}H_{27}N_2O_2S$ $[M+H]^+$ 395.1788, found 395.1787.

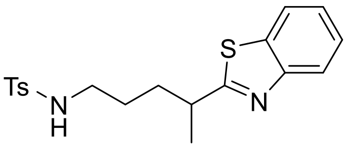

3r: yellow oil. ^{1}H NMR (400 MHz, $CDCl_3$) δ 7.97-7.93 (m, 1H), 7.84-7.81 (m, 1H), 7.72-7.67 (m, 2H), 7.47-7.41 (m, 1H), 7.37-7.31 (m, 1H), 7.24-7.19 (m, 2H), 5.20-5.14 (m, 1H), 3.26-3.16 (m, 1H), 2.98-2.89 (m, 2H), 2.36 (s, 3H), 1.89-1.78 (m, 1H), 1.77-1.67 (m, 1H), 1.60-1.40 (m, 2H), 1.38 (d, *J* = 6.8 Hz, 3H); ^{13}C NMR (100 MHz, $CDCl_3$) δ 177.2, 152.7, 143.3, 136.9, 134.5, 129.6, 127.0, 126.0, 124.8, 122.6, 121.6, 43.0, 38.8, 34.0, 27.1, 21.5, 21.4. FT-IR: ν (cm^{-1}) 3276, 3063, 2964, 2927, 2869, 1597, 1495, 1739, 1242. HRMS [ESI] calcd for $C_{19}H_{22}N_2NaO_2S_2$ $[M+Na]^+$ 397.1015, found 397.1020.

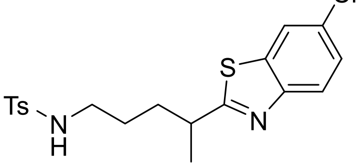

3s: yellow oil. ^{1}H NMR (400 MHz, $CDCl_3$) δ 7.91 (s, 1H), 7.75-7.67 (m, 3H), 7.34-7.30 (m, 1H), 7.25-7.21 (m, 2H), 5.04-4.92 (m, 1H), 3.25-3.15 (m, 1H), 2.99-2.89 (m, 2H), 2.38 (s, 3H), 1.89-1.78 (m, 1H), 1.78-1.67 (m, 1H), 1.60-1.41 (m, 2H), 1.38 (d, *J* = 6.8 Hz, 3H); ^{13}C NMR (100 MHz, $CDCl_3$) δ 179.2, 153.7, 143.3, 136.9, 132.8, 132.0, 129.7, 127.0, 125.3, 122.5, 122.3, 42.9, 38.9, 34.0, 27.1, 21.5, 21.2. FT-IR: ν (cm^{-1}) 3280, 3061, 2959, 2852, 1590,

1495, 1378, 1323. HRMS [ESI] calcd for $C_{19}H_{21}ClN_2NaO_2S_2$ $[M+Na]^+$ 431.0625, found 431.0632.

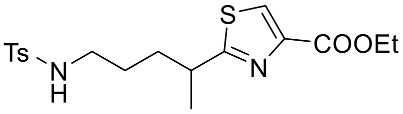

3t: yellow oil. ^1H NMR (400 MHz, $CDCl_3$) δ 8.04 (s, 1H), 7.74-7.70 (m, 2H), 7.30-7.26 (m, 2H), 4.80-4.69 (m, 1H), 4.40 (q, *J* = 7.2 Hz, 2H), 3.27-3.17 (m, 1H), 2.97-2.87 (m, 2H), 2.41 (s, 3H), 1.83-1.61 (m, 2H), 1.59-1.41 (m, 2H), 1.39 (t, *J* = 7.2 Hz, 3H), 1.34 (d, *J* = 6.8 Hz, 3H); ^{13}C NMR (100 MHz, $CDCl_3$) δ 177.4, 161.4, 146.5, 143.3, 137.0, 129.7, 127.1, 126.6, 61.4, 42.9, 38.2, 34.4, 27.2, 21.5, 21.4, 14.4. FT-IR: ν (cm^{-1}) 3280, 3121, 2963, 2871, 2854, 1717, 1598, 1453, 1369, 1212. HRMS [ESI] calcd for $C_{18}H_{24}N_2NaO_4S_2$ $[M+Na]^+$ 419.1070, found 419.1064.

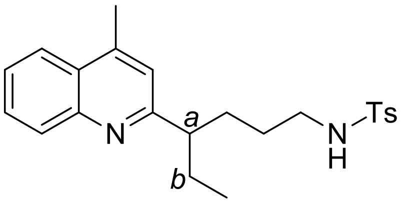

3u (4.6:1): yellow oil. ^1H NMR (400 MHz, $CDCl_3$) δ 8.08-8.02(m, 1H, two isomers), 7.93-7.89 (m, 1H, two isomers), 7.71-7.59 (m, 3H, two isomers), 7.51-7.42 (m, 1H, two isomers), 7.22-7.17 (m, 0.36H, one isomer), 7.17-7.12 (m, 1.64H, one isomer), 7.07 (s, 0.18H, one isomer), 7.03 (s, 0.82H, one isomer), 6.00-5.91 (m, 0.82H, one isomer), 5.40-5.25 (m, 0.18H, one isomer), 2.97-2.78 (m, 2H, two isomers), 2.74-2.65 (m, 1H, two isomers), 2.64 (s, 0.54H, one isomer), 2.63 (s, 2.36H, one isomer), 2.33 (s, 0.54H, one isomer), 2.32 (s, 2.36H, one isomer), 1.79-1.58 (m, 3H, two isomers), 1.47-1.19 (m, 3.54H, two isomers), 0.74 (t, *J* = 7.6 Hz, 2.46H, one isomer); ^{13}C NMR (100 MHz, $CDCl_3$) δ 166.3 & 164.7 (two isomers), 147.4 & 147.3 (two isomers), 144.7 (overlap, two isomers), 143.1 & 143.0 (two isomers), 137.1 (overlap, two isomers), 129.6 & 129.5 (two isomers), 129.4 (overlap, two isomers), 129.2 & 129.0 (two isomers), 127.1 (overlap, two isomers), 125.7 & 125.5 (two isomers), 123.7 (overlap, two isomers), 120.7 & 120.2 (two isomers), 49.2 & 43.0 (two isomers), 43.2 & 42.52 (two isomers), 36.2 & 32.3 (two isomers), 29.5 & 28.7 (two isomers), 27.0 & 24.5 (two isomers), 21.5 & 20.8 (two isomers), 18.9 (overlap, two isomers), 12.1 (overlap, two isomers). FT-IR: ν (cm^{-1}) 3065, 2964, 2927, 2850, 1635, 1556, 1456, 1379, 1246. HRMS [ESI] calcd for $C_{23}H_{29}N_2O_2S$ $[M+H]^+$ 397.1944, found 397.1966.

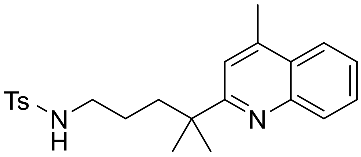

3v: yellow oil. ^1H NMR (400 MHz, $CDCl_3$) δ 8.23-8.19 (m, 1H), 7.97-7.93 (m, 1H), 7.71 (ddd, *J* = 8.4, 6.8, 1.6 Hz, 1H), 7.69-7.65 (m, 2H), 7.53 (ddd, *J* = 8.0, 6.8, 1.2 Hz, 1H), 7.26 (s, 1H), 7.22-7.17 (m, 2H), 5.49-5.44 (m, 1H), 2.91-2.85 (m, 2H), 2.37 (s, 3H), 1.81-1.75 (m, 2H), 1.38-1.30 (m, 2H), 1.33 (s, 6H). ^{13}C NMR (100 MHz, $CDCl_3$) δ 167.2, 147.1, 144.3, 143.0, 137.0, 129.7, 129.5, 129.2, 127.1, 126.5, 125.8, 123.4, 119.4, 43.4, 40.7, 38.0, 28.5, 24.6, 21.5, 19.0. FT-IR: ν (cm^{-1}) 3273, 3068, 2960, 2920, 2851, 1600, 1508, 1448, 1387, 1263. HRMS [ESI] calcd for $C_{23}H_{29}N_2O_2S$ $[M+H]^+$ 397.1944, found 397.1968.

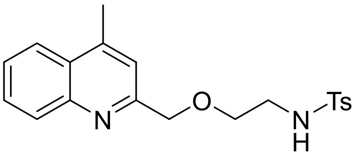

3w: yellow oil. ^1H NMR (400 MHz, $CDCl_3$) δ 8.23-8.18 (m, 1H),

7.99-7.94 (m, 1H), 7.76-7.68 (m, 3H), 7.58-7.52 (m, 1H), 7.22-7.18 (m, 3H), 6.50 (br, 1H), 4.68 (s, 2H), 3.63 (t, *J* = 4.8 Hz, 2H), 3.23-3.17 (m, 2H), 2.68 (s, 3H), 2.35 (s, 3H); ^{13}C NMR (100 MHz, $CDCl_3$) δ 157.7, 147.3, 145.4, 143.1, 137.1, 129.7, 129.6, 129.6, 127.5, 127.1, 126.4, 123.7, 119.9, 73.6, 69.2, 43.2, 21.5, 18.8. FT-IR: ν (cm^{-1}) 3551, 3477, 3414, 2924, 2866, 1638, 1566, 1447, 1384, 1326. HRMS [ESI] calcd for $C_{20}H_{23}N_2O_3S$ $[M+H]^+$ 371.1424, found 371.1432.

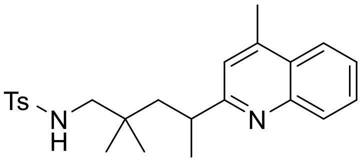

3x: white solid, m.p. 113-114 °C. ^{1}H NMR (400 MHz, $CDCl_3$) δ 8.26-8.22 (m, 1H), 7.97-7.93 (m, 1H), 7.78-7.72 (m, 1H), 7.59-7.54 (m, 1H), 7.18-7.14 (m, 2H), 7.08 (s, 1H), 6.95-6.91 (m, 2H), 6.66-6.60 (m, 1H), 3.05-2.95 (m, 1H), 2.63 (s, 3H), 2.57-2.50 (m, 1H), 2.47-2.39 (m, 1H), 2.28 (s, 3H), 2.16-2.10 (m, 1H), 1.32-1.28 (m, 1H), 1.27 (d, *J* = 6.8 Hz, 3H), 0.98 (s, 3H), 0.89 (s, 3H). ^{13}C NMR (100 MHz, $CDCl_3$) δ 166.4, 147.2, 145.2, 142.2, 137.3, 129.8, 129.5, 129.1, 127.0, 126.6, 125.9, 123.6, 121.1, 51.3, 44.3, 37.7, 34.5, 27.7, 25.5, 24.4, 21.4, 18.8. FT-IR: ν (cm^{-1}) 3064, 2965, 2928, 2874, 1599, 1510, 1457, 1375, 1286, 1183. HRMS [ESI] calcd for $C_{24}H_{31}N_2NaO_2S$ $[M+Na]^+$ 411.2101, found 411.2098.

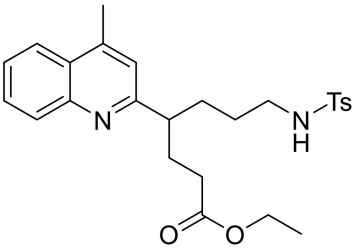

3y: yellow oil.^{1}H NMR (400 MHz, $CDCl_3$) δ 8.08-8.03 (m, 1H), 7.96-7.91 (m, 1H), 7.70-7.62 (m, 3H), 7.54-7.46 (m, 1H), 7.20-7.15 (m, 2H), 7.04 (s, 1H), 5.61-5.47 (m, 1H), 4.07-3.97(m, 2H), 2.96-2.76 (m, 3H), 2.65 (s, 3H), 2.35 (s, 3H), 2.23-1.91 (m, 4H), 1.87-1.74 (m, 1H), 1.72-1.61 (m, 1H), 1.45-1.21 (m, 2H), 1.17 (t, *J* = 7.2 Hz, 3H); ^{13}C NMR (100 MHz, $CDCl_3$) δ 173.5, 163.4, 147.5, 144.9, 143.1, 137.0, 129.6, 129.3, 127.1, 127.0, 125.9, 123.6, 120.9, 60.3, 46.9, 43.1, 32.2, 30.6, 27.0, 21.5, 18.9, 14.2. FT-IR: ν (cm^{-1}) 3282, 2938, 2869, 1733, 1653, 1559, 1456, 1375, 1158. HRMS [ESI] calcd for $C_{26}H_{33}N_2O_4S$ $[M+H]^+$ 469.2156, found 469.2166.

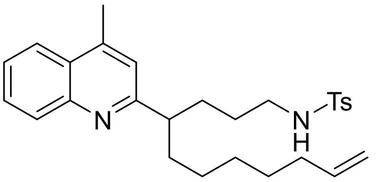

3z: yellow oil. ^{1}H NMR (400 MHz, $CDCl_3$) δ 8.16-8.10 (m, 1H), 7.98-7.94 (m, 1H), 7.73-7.64 (m, 3H), 7.53 (ddd, *J* = 8.4, 6.8, 1.2 Hz, 1H), 7.22-7.17 (m, 2H), 7.05 (s, 1H), 5.80-5.69 (m, 1H), 5.59-5.52 (m, 1H), 4.97-4.86 (m, 2H), 2.99-2.898 (m, 1H), 2.88-2.76 (m, 2H), 2.68 (s, 3H), 2.37 (s, 3H), 2.00-1.92 (m, 3H), 1.82-1.56 (m, 5H), 1.38-1.16 (m, 5H), 1.12-1.01 (m, 1H); ^{13}C NMR (100 MHz, $CDCl_3$) δ 164.7, 143.0, 139.0, 137.1, 129.5, 129.4, 129.3, 127.1, 127.0, 125.8, 123.6, 120.6, 114.2, 47.5, 43.2, 35.8, 33.7, 32.7, 29.2, 28.7, 27.4, 26.9, 21.5, 18.9. FT-IR: ν (cm^{-1}) 3282, 3065, 2927, 2854, 1639, 1561, 1447, 1379, 1157. HRMS [ESI] calcd for $C_{28}H_{37}N_2O_2S$ $[M+H]^+$ 465.2570, found 465.2563.

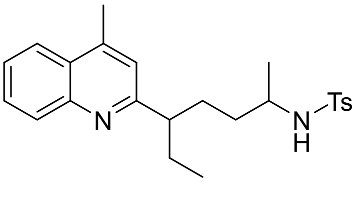

3aa-1 (*d.r.* =1/1.15, one isomer)**:** yellow oil. ^{1}H NMR (400 MHz, $CDCl_3$) δ 8.27-8.22 (m, 1H), 7.98-7.93 (m, 1H), 7.80-7.75 (m, 2H), 7.74-7.68 (m, 1H), 7.55-7.50 (m, 1H), 7.23-7.19 (m, 2H), 7.04 (s, 1H), 5.71-5.64 (m, 1H), 3.28-3.16 (m, 1H), 2.76-2.68 (m, 1H), 2.67 (s, 3H), 2.36 (s, 3H),

1.72-1.44 (m, 4H), 1.41-1.31 (m, 1H), 1.13-1.03 (m, 1H), 1.01 (d, *J* = 6.4 Hz, 3H), 0.72 (t, *J* = 7.6 Hz, 3H); ^{13}C NMR (100 MHz, $CDCl_3$) δ 164.5, 147.5, 144.5, 142.9, 138.1, 129.6, 129.5, 129.3, 127.2, 127.0, 125.7, 123.6, 121.6, 50.3, 48.9, 34.5, 29.2, 29.1, 22.3, 21.5, 18.9, 11.6. FT-IR: ν (cm^{-1}) 3278, 3064, 2962, 2872, 1601, 1508, 1448, 1379, 1158. HRMS [ESI] calcd for $C_{24}H_{31}N_2O_2S$ $[M+H]^+$ 411.2101, found 411.2098.

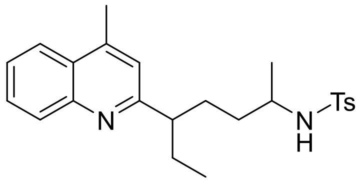

3aa-2 (*d.r.* =1/1.15, one isomer)**:** yellow oil $^{.1}$H NMR (400 MHz, $CDCl_3$) δ 8.14-8.10 (m, 1H), 7.98-7.40 (m, 1H), 7.72-7.65 (m, 3H), 7.55-7.50 (m, 1H), 7.18-7.13 (m, 2H), 7.03 (s, 1H), 5.37-5.26 (m, 1H), 3.29-3.18 (m, 1H), 2.73-2.59 (m, 1H), 2.68 (s, 3H) 2.34 (s, 3H), 1.77-1.56 (m, 4H), 1.38-1.21 (m, 1H), 1.18-1.08 (m, 1H), 1.01 (d, *J* = 6.4 Hz, 3H), 0.76 (t, *J* = 7.2 Hz, 3H); ^{13}C NMR (100 MHz, $CDCl_3$) δ 164.7, 147.4, 144.6, 142.9, 138.3, 129.5, 129.5, 129.2, 127.0, 127.0, 125.7, 123.6, 120.7, , 50.5, 49.2, 34.8, 31.1, 28.5, 22.2, 21.4, 18.9, 12.0. FT-IR: ν (cm^{-1}) 3278, 3062, 2963, 2929, 2872, 1601, 1508, 1448, 1379, 1158. HRMS [ESI] calcd for $C_{24}H_{31}N_2O_2S$ $[M+H]^+$ 411.2101, found 411.2098.

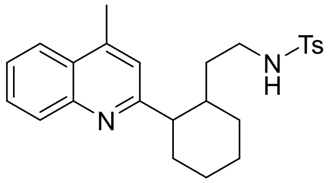

3ab (*d.r.* >19:1): yellow oil. ^{1}H NMR (400 MHz, $CDCl_3$) δ 8.28-8.24 (m, 1H), 7.95-7.91 (m, 1H), 7.71 (ddd, *J* = 8.4, 6.8, 1.6 Hz, 1H), 7.68-7.64 (m, 2H), 7.51 (ddd, *J* = 8.4, 7.2, 1.2 Hz, 1H), 7.17-7.12 (m, 2H), 7.08-7.59 (m, 1H), 6.42 (t, *J* = 4.4 Hz, 1H), 2.90-2.83 (m, 2H), 2.64 (s, 3H), 2.56-2.48 (m, 1H), 2.34 (s, 3H), 1.90-1.65 (m, 5H), 1.39-1.22 (m, 3H), 1.17-1.07 (m, 2H), 1.06-0.94 (m, 1H); ^{13}C NMR (100 MHz, $CDCl_3$) δ 165.1, 147.2, 145.0, 142.8, 136.9, 129.5, 129.4, 129.3, 127.1, 127.0, 125.8, 123.6, 121.8, 51.7, 40.7, 37.2, 34.9, 33.3, 32.3, 26.4, 26.1, 21.5, 18.8. FT-IR: ν (cm^{-1}) 3272, 3063, 2924, 2853, 1602, 1508, 1446, 1326, 1289.HRMS [ESI] calcd for $C_{25}H_{31}N_2O_2S$ $[M+H]^+$ 423.2101, found 423.2114.

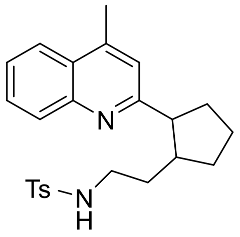

3ac (*d.r.* >19:1): yellow oil. ^{1}H NMR (400 MHz, $CDCl_3$) ^{1}H NMR (400 MHz, $CDCl_3$) δ 8.20 (d, *J* = 8.4 Hz, 1H), 7.95 (d, *J* = 8.0 Hz, 1H), 7.76-7.70 (m, 1H), 7.58-7.52 (m, 1H), 7.49-7.45 (m, 2H), 7.10 (s, 1H), 7.05 (d, *J* = 8.0 Hz, 2H), 6.50 (t, *J* = 6.0 Hz, 1H), 3.00-2.84 (m, 2H), 2.74-2.65 (m, 1H), 2.66 (s, 3H), 2.58-2.47 (m, 1H), 2.32 (s, 3H), 2.22-2.14 (m, 1H), 1.97-1.88 (m, 1H), 1.79-1.64 (m, 4H), 1.52-1.42 (m, 1H), 1.37-1.28 (m, 1H). ^{13}C NMR (100 MHz, $CDCl_3$) δ 164.5, 147.0, 144.9, 142.6, 137.3, 129.6, 129.3, 127.0, 126.9, 125.9, 123.6, 121.2, 53.1, 41.6, 41.3, 34.9, 34.5, 33.3, 24.7, 21.4, 18.8. FT-IR: ν (cm^{-1}) 3280, 3062, 2925, 2867, 1601, 1508, 1495, 1412, 1325, 1155. HRMS [ESI] calcd for $C_{24}H_{29}N_2O_2S$ $[M+H]^+$ 409.1944, found 409.1944.

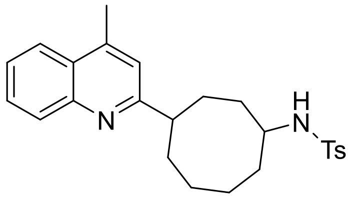

3ad (*d.r.* = 1:1.5): yellow oil. ^{1}H NMR (400 MHz, $CDCl_3$) δ 8.04-8.00 (m, 0.4H, one isomer), 8.00-7.96 (m, 0.6H, one isomer), 7.93-7.88 (m, 1H, two isomers), 7.82-7.76 (m, 2H), 7.67-7.60 (m, 1H, two isomers), 7.50-7.44 (m, 1H, two isomers), 7.28-7.22 (m, 2H, two isomers), 7.06-7.03 (m, 1H, two isomers),

5.48-5.36 (m, 0.4H, one isomer), 5.33-5.17 (m, 0.6H, one isomer), 3.58-3.43 (m, 1H), 3.06-2.92 (m, 1H, two isomers), 2.63 (s, 3H, two isomers), 2.37 (s, 3H, two isomers), 2.05-1.35 (m, 12H, two isomers); ^{13}C NMR (100 MHz, $CDCl_3$) δ 167.4 & 167.2 (two isomers), 147.3 & 147.3 (two isomers), 144.6 (overlap, two isomers), 143.1 (overlap, two isomers), 138.4 & 138.3 (two isomers), 129.6 & 129.6 (two isomers), 129.4 & 129.3 (two isomers), 129.0 & 129.0 (two isomers), 127.0 (overlap, two isomers), 126.9 & 126.9 (two isomers), 125.5 (two isomers), 123.6 (two isomers), 120.6 (two isomers), 53.9 & 53.5 (overlap, two isomers), 47.9 & 47.4 (overlap, two isomers), 32.3 & 32.03 (overlap, two isomers), 31.5 & 31.5 (overlap, two isomers), 30.1 & 28.5 (overlap, two isomers), 27.0 & 26.1 (overlap, two isomers), 23.3 & 23.3 (overlap, two isomers), 21.5 (overlap, two isomers), 18.8 (overlap, two isomers). FT-IR: ν (cm^{-1}) 3280, 3062, 3031, 2922, 2856, 1601, 1508, 1446, 1379, 1156. HRMS [ESI] calcd for $C_{25}H_{31}N_2O_2S$ $[M+H]^+$ 423.2101, found 423.2105.

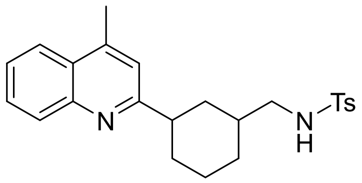

3ae (*dr* >19:1): yellow oil. ^{1}H NMR (400 MHz, $CDCl_3$) δ 8.04 - 7.99 (m, 1H), 7.96-7.92 (m, 1H), 7.75-7.71 (m, 2H), 7.69-7.63 (m, 1H), 7.53-7.47 (m, 1H), 7.30-7.25 (m, 2H), 7.01 (s, 1H), 4.74 (t, *J* = 6.4 Hz, 1H), 2.90-2.79 (m, 3H), 2.67 (s, 3H), 2.38 (s, 3H), 2.03-1.95 (m, 2H), 1.95-1.87 (m, 1H), 1.84-1.75 (m, 1H), 1.73-1.61 (m, 1H), 1.59-1.36 (m, 2H), 1.34-1.23 (m, 1H), 1.05-0.93 (m, 1H); ^{13}C NMR (100 MHz, $CDCl_3$) δ 165.4, 147.5, 144.6, 143.3, 137.0, 129.6, 129.3, 129.1, 127.1, 125.5, 123.6, 120.3, 49.4, 46.6, 37.9, 36.3, 32.4, 30.0, 25.7, 21.5, 18.8. FT-IR: ν (cm^{-1}) 3280, 3061, 2923, 2852, 1683, 1560, 1446, 1378, 1323, 1156. HRMS [ESI] calcd for $C_{24}H_{29}N_2O_2S$ $[M+H]^+$ 409.1944, found 409.1949.

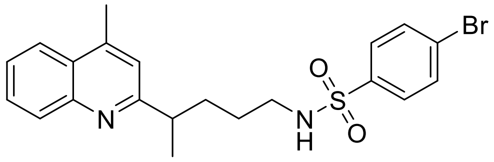

3af: yellow oil. ^{1}H NMR (400 MHz, $CDCl_3$) δ 8.12-8.08 (m, 1H), 7.97-7.93 (m, 1H), 7.72-7.66 (m, 1H), 7.66-7.59 (m, 2H), 7.55-7.48 (m, 3H), 7.08 (s, 1H), 6.17 (br, 1H), 3.03-2.91 (m, 2H), 2.91-2.82 (m, 1H), 2.67 (s, 3H), 1.90 - 1.78 (m, 1H), 1.69 - 1.57 (m, 1H), 1.50 - 1.32 (m, 2H), 1.28 (d, *J* = 7.2 Hz, 3H), ^{13}C NMR (100 MHz, $CDCl_3$) δ 165.5, 147.1, 145.1, 139.2, 132.1, 129.4, 129.2, 128.6, 127.1, 127.0, 125.8, 123.7, 120.3, 43.1, 41.5, 33.7, 26.9, 21.2, 18.9. FT-IR: ν (cm^{-1}) 3281, 3063, 2961, 2868, 1603, 1561, 1470, 1388, 1274. HRMS [ESI] calcd for $C_{21}H_{24}BrN_2O_2S$ $[M+H]^+$ 447.0736, found 447.0726.

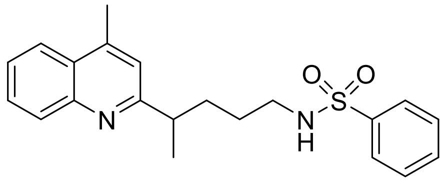

3ag: yellow oil. ^{1}H NMR (400 MHz, $CDCl_3$) δ 8.11 (d, *J* = 8.4 Hz, 1H), 7.96-7.92 (d, *J* = 8.0 Hz, 1H), 7.83-7.77 (m, 2H), 7.71-7.65 (m, 1H), 7.55-7.44 (m, 2H), 7.42-7.36 (m, 2H), 7.08 (s, 1H), 5.87-5.81 (t, *J* = 1.3 Hz, 1H), 3.03-2.84 (m, 3H), 2.66 (s, 3H), 1.87-1.75 (m, 1H), 1.68-1.57 (m, 1H), 1.50-1.32 (m, 2H), 1.28 (d, *J* = 7.2 Hz, 3H); ^{13}C NMR (100 MHz, $CDCl_3$) δ 165.5, 147.0, 145.2, 140.1, 132.3, 129.4, 129.2, 128.9, 127.0, 127.0, 125.8, 123.6, 120.2, 43.1, 41.5, 33.8, 27.1, 21.0, 18.9. FT-IR: ν (cm^{-1}) 3282, 3064, 2959, 2868, 2853, 1603, 1508, 1446, 1380, 1156. HRMS [ESI] calcd for $C_{21}H_{25}N_2O_2S$ $[M+H]^+$ 369.1631, found 369.1632.

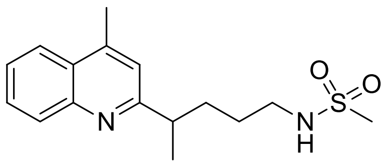

3ah: yellow oil. ^{1}H NMR (400 MHz, $CDCl_3$) δ 8.06-8.01 (m, 1H), 7.93-7.88 (m, 1H), 7.67-7.60 (m, 1H), 7.50-7.44 (m, 1H),

7.11 (s, 1H), 5.60-5.53 (m, 1H), 3.12-2.98 (m, 3H), 2.82 (s, 3H), 2.65 (s, 3H), 1.96-1.84 (m, 1H), 1.77-1.65 (m, 1H), 1.60-1.40 (m, 2H), 1.33 (d, *J* = 7.2 Hz, 3H); ^{13}C NMR (100 MHz, $CDCl_3$) δ 165.7, 147.3, 144.9, 129.2, 129.2, 127.1, 125.7, 123.7, 120.3, 43.2, 41.9, 39.7, 33.7, 27.7, 21.1, 18.9. FT-IR: ν (cm^{-1}) 3286, 2958, 2918, 2850, 1602, 1508, 1448, 1379, 1147. HRMS [ESI] calcd for $C_{16}H_{23}N_2O_2S$ [M+H]$^+$ 307.1475, found 307.1481.

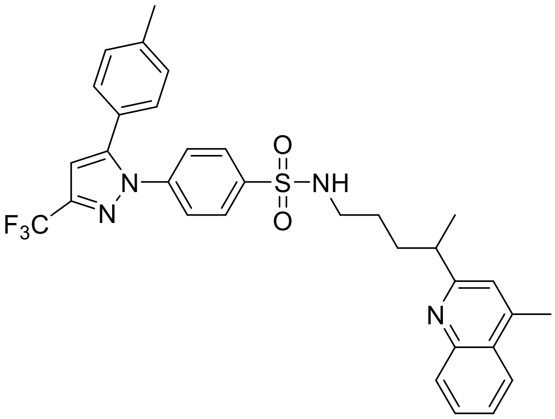

3ai: yellow oil. ^{1}H NMR (400 MHz, $CDCl_3$) δ 8.11-8.07 (m, 1H), 7.94-7.90 (m, 1H), 7.78-7.73 (m, 2H), 7.68-7.61 (m, 1H), 7.52-7.46 (m, 1H), 7.34-7.29 (m, 2H), 7.17-7.12 (m, 2H), 7.11-7.04 (m, 3H), 6.73 (s, 1H), 6.42-6.36 (m, 1H), 3.04-2.93 (m, 2H), 2.93-2.83 (m, 1H), 2.65 (s, 3H), 2.35 (s, 3H), 1.91-1.79 (m, 1H), 1.70-1.59 (m, 1H), 1.49-1.33 (m, 2H), 1.29 (d, *J* = 6.8 Hz, 3H); ^{13}C NMR (101 MHz, $CDCl_3$) δ 165.5, 147.1, 145.2 (q, $J_{C\text{-}F}$ = 4.7 Hz), 143.9 (q, $J_{C\text{-}F}$ = 38.2 Hz), 142.1, 139.8 (q, $J_{C\text{-}F}$ = 7.3 Hz), 129.7, 129.4, 129.2, 128.7, 128.0, 127.0, 125.8, 125.7, 125.4, 123.7, 121.2 (q, $J_{C\text{-}F}$ = 267.4 Hz), 120.3, 106.2, 43.1, 41.5, 33.8, 26.9, 21.3, 21.3, 18.8. ^{19}F NMR (376 MHz, $CDCl_3$) δ -62.4 (s). FT-IR: ν (cm^{-1}) 3290, 3066, 2962, 2849, 1601, 1561, 1471, 1374, 1269. HRMS [ESI] calcd for $C_{32}H_{32}F_3N_4O_2S$ [M+H]$^+$ 593.2193, found 593.2190.

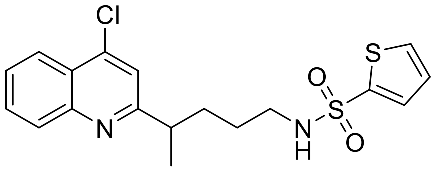

3aj: yellow oil .^{1}H NMR (400 MHz, $CDCl_3$) δ 8.19-8.15 (m, 1H), 8.12-8.07 (m, 1H), 7.77-7.71 (m, 1H), 7.62-7.56 (m, 1H), 7.53-7.50 (m, 1H), 7.47-7.43 (m, 1H), 7.35-7.33 (m, 1H), 6.99-6.95 (m, 1H), 5.79-5.71 (m, 1H), 3.1-2.95 (m, 3H), 1.89-1.77 (m, 1H), 1.73-1.62 (m, 1H), 1.54-1.33 (m, 2H), 1.32-1.28 (m, 3H); ^{13}C NMR (100 MHz, $CDCl_3$) δ 166.9, 148.3, 143.0, 141.1, 131.9, 131.5, 130.5, 129.2, 127.3, 127.0, 125.1, 123.9, 119.9, 43.4, 41.8, 33.6, 27.0, 20.9. FT-IR: ν (cm^{-1}) 3283, 3095, 2963, 2869, 1615, 1553, 1454, 1371, 1225. HRMS [ESI] calcd for $C_{18}H_{20}ClN_2O_2S$ [M+H]$^+$ 395.0649, found 395.0641.

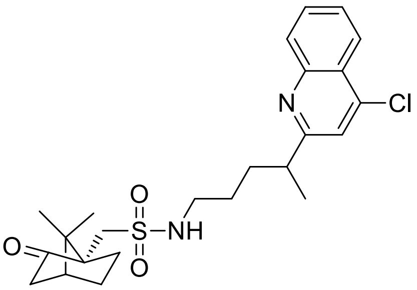

3ak (*d.r.* =1:1.2): yellow oil. ^{1}H NMR (400 MHz, $CDCl_3$) δ 8.16-8.12 (m, 1H, two isomers), 8.06-8.01 (m, 1H, two isomers), 7.73-7.67 (m, 1H, two isomers), 7.58-7.51 (m, 1H, two isomers), 7.37 (s, 1H, two isomers), 5.44-5.38 (br, 1H, two isomers), 3.36-3.34 (m, 0.46H, one isomer), 3.32-3.30 (m, 0.54H, one isomer), 3.21-3.00 (m, 3H), 2.87-2.84 (m, 0.54H, one isomer), 2.83-2.80 (m, 0.46H, one isomer), 2.35-2.25 (m, 1H, two isomers), 2.25-2.16 (m, 1H, two isomers), 2.07-2.03 (m, 1H, two isomers), 2.02-1.80 (m, 4H, two isomers), 1.80-1.69 (m, 1H, two isomers), 1.67-1.55 (m, 1H, two isomers), 1.54-1.43 (m, 1H, two isomers), 1.42-1.31 (m, 1H, two isomers), 1.34 (d, *J* = 6.8 Hz, 3H, two isomers); ^{13}C NMR (100 MHz, $CDCl_3$) δ 216.8 (overlap, two isomers), 166.2 (overlap, two isomers), 148.5 (overlap, two isomers), 142.8 (overlap,

two isomers), 130.3 (overlap, two isomers), 129.3 (overlap, two isomers), 126.8 (overlap, two isomers), 125.1 (overlap, two isomers), 123.9 (overlap, two isomers), 119.9 (overlap, two isomers), 59.1 (overlap, two isomers), 49.1 & 49.1 (two isomers), 48.7 & 48.7 (two isomers), 43.6 & 43.6 (two isomers), 42.9 & 42.7 (two isomers), 42.2 & 42.2 (two isomers), 33.6 (overlap, two isomers), 28.0 & 28.0 (two isomers), 27.0 (overlap, two isomers), 26.4 & 26.4 (two isomers), 20.8 & 20.7 (two isomers), 19.8 & 19.5(two isomers). FT-IR: ν (cm^{-1}) 3290, 3062, 2960, 1739, 1615, 1553, 1454, 1374, 1277. HRMS [ESI] calcd for $C_{24}H_{31}ClN_2NaO_3S$ $[M+Na]^+$ 485.1636, found 485.1636.

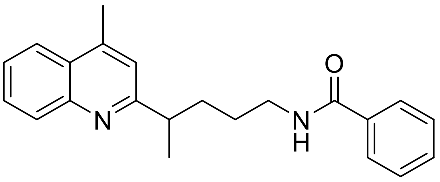

4a: yellow oil. ^{1}H NMR (400 MHz, $CDCl_3$) δ 7.99-7.91 (m, 2H), 7.81-7.76 (m, 2H), 7.62 (ddd, J = 8.4, 6.8, 1.2 Hz, 1H), 7.52-7.41 (m, 2H), 7.41-7.34 (m, 2H), 7.14 (s, 1H), 6.86 (t, J = 7.2 Hz, 1H), 3.50-3.32 (m, 2H), 3.17-3.06 (m, 1H), 2.67 (s, 3H), 1.99-1.87 (m, 1H), 1.81-1.70 (m, 1H), 1.68-1.56 (m, 1H), 1.56-1.43 (m, 1H), 1.36 (d, J = 7.2 Hz, 3H); ^{13}C NMR (100 MHz, $CDCl_3$) δ 167.6, 166.0, 147.3, 144.9, 134.8, 131.2, 129.2, 129.1, 128.4, 127.1, 127.0, 125.6, 123.7, 120.2, 42.1, 40.2, 34.5, 27.3, 21.0, 18.9. FT-IR: ν (cm^{-1}) 3321, 3062, 2961, 2929, 2869, 1637, 1578, 1489, 1373, 1158. HRMS [ESI] calcd for $C_{22}H_{24}N_2ONa$ $[M+Na]^+$ 355.1781, found 355.1792.

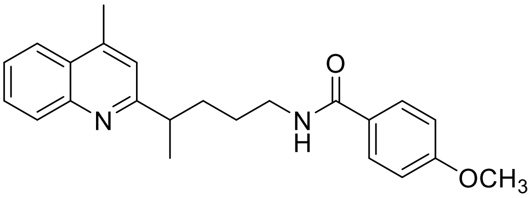

4b: yellow oil. ^{1}H NMR (400 MHz, $CDCl_3$) δ 7.98 (d, J = 8.4 Hz, 1H), 7.94 (d, J = 8.0 Hz, 1H), 7.79-7.73 (m, 2H), 7.67-7.61 (m, 1H), 7.52-7.46 (m, 1H), 7.14 (s, 1H), 6.90-6.85 (m, 2H), 6.65-6.54 (m, 1H), 3.82 (s, 3H), 3.49-3.32 (m, 2H), 3.17-3.06 (m, 1H), 2.67 (s, 3H), 1.99-1.88 (m, 1H), 1.81-1.71 (m, 1H), 1.68-1.55 (m, 1H), 1.55-1.43 (m, 1H), 1.37 (d, J = 6.8 Hz, 3H); ^{13}C NMR (100 MHz, $CDCl_3$) δ 167.1, 166.1, 162.0, 147.4, 144.8, 129.3, 129.1, 128.8, 127.2, 127.1, 125.6, 123.7, 120.3, 113.6, 55.4, 42.2, 40.2, 34.6, 27.4, 21.0, 18.9. FT-IR: ν (cm^{-1}) 3318, 3063, 2929, 2856, 1631, 1574, 1442, 1272, 1298. HRMS [ESI] calcd for $C_{23}H_{26}N_2NaO_2$ $[M+Na]^+$ 385.1886, found 385.1887.

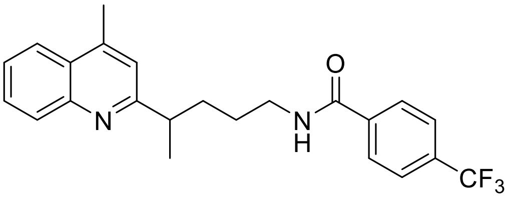

4c: yellow oil. ^{1}H NMR (400 MHz, $CDCl_3$) δ 7.94-7.85 (m, 4H), 7.61-7.56 (m, 3H), 7.50-7.40 (m, 2H), 7.13 (s, 1H), 3.50-3.31 (m, 2H), 3.16-3.05 (m, 1H), 2.65 (s, 3H), 1.98-1.85 (m, 1H), 1.80-1.68 (m, 1H), 1.66-1.44 (m, 2H), 1.34 (d, J = 7.2 Hz, 3H); ^{13}C NMR (100 MHz, $CDCl_3$) δ 166.4, 165.9, 147.1, 145.2, 138.2, 132.9 (q, $J_{C\text{-}F}$ = 32.4 Hz,), 129.3, 128.9, 127.6, 127.1, 125.8, 125.4 (q, $J_{C\text{-}F}$ = 3.7 Hz), 123.8 (q, $J_{C\text{-}F}$ = 270.8 Hz), 123.7, 120.2, 41.9, 40.5, 34.7, 26.9, 21.0, 18.9; ^{19}F NMR (376 MHz, $CDCl_3$) δ -62.9 (s). FT-IR: ν (cm^{-1}) 3301, 3070, 2963, 2871, 1642, 1549, 1376, 1262. HRMS [ESI] calcd for $C_{23}H_{24}F_3N_2O$ $[M+H]^+$ 401.1835, found 401.1848.

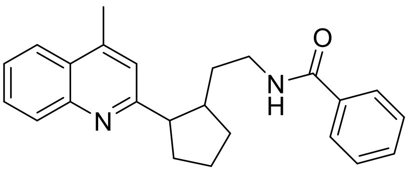

4d: yellow oil. ^{1}H NMR (400 MHz, $CDCl_3$) δ 7.96-7.87 (m, 2H), 7.76-7.71 (m, 2H), 7.62-7.56 (m, 1H), 7.51-7.42 (m,

2H), 7.40-7.34 (m, 2H), 7.18 (s, 1H), 7.01 (br, 1H), 3.47-3.37 (m, 1H), 3.29-3.19 (m, 1H), 3.07-2.98 (m, 1H), 2.67 (s, 3H), 2.59-2.47 (m, 1H), 2.26-2.17 (m, 1H), 2.17-2.07 (m, 1H), 1.92-1.76 (m, 4H), 1.69-1.60 (m, 1H), 1.52-1.41 (m, 1H); ^{13}C NMR (100 MHz, $CDCl_3$) δ 167.4, 165.3, 147.4, 144.7, 135.1, 131.1, 129.2, 129.1, 128.4, 127.1, 127.1, 125.6, 123.7, 121.2, 54.4, 43.7, 38.6, 35.0, 34.8, 33.3, 24.8, 18.8. FT-IR: ν (cm^{-1}) 3317, 3062, 2947, 2868, 1640, 1578, 1488, 1345, 1177. HRMS [ESI] calcd for $C_{24}H_{27}N_2O$ $[M+H]^+$ 359.2118, found 359.2111.

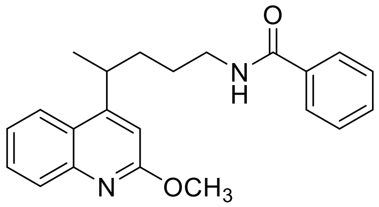

4e: yellow oil. ^{1}H NMR (400 MHz, $CDCl_3$) δ 7.94 (d, *J* = 8.0 Hz, 1H), 7.90-7.85 (m, 1H), 7.72-7.68 (m, 2H), 7.60 (ddd, *J* = 8.4, 6.8, 1.2 Hz, 1H), 7.50-7.44 (m, 1H), 7.42-7.33 (m, 3H), 6.79 (s, 1H), 6.20-6.12 (m, 1H), 4.06 (s, 3H), 3.58-3.48 (m, 1H), 3.47-3.35 (m, 2H), 1.91-1.51 (m, 4H), 1.35 (d, *J* = 6.8 Hz, 3H); ^{13}C NMR (100 MHz, $CDCl_3$) δ 167.6, 162.5, 155.6, 147.2, 134.6, 131.4, 129.2, 128.5, 128.2, 126.8, 124.2, 123.9, 122.9, 109.2, 53.2, 39.9, 34.3, 33.1, 27.7, 21.1. FT-IR: ν (cm^{-1}) 3317, 3064, 2930, 2857, 1635, 1574, 1489, 1387, 1235. HRMS [ESI] calcd for $C_{22}H_{25}N_2O_2$ $[M+H]^+$ 349.1911, found 349.1916.

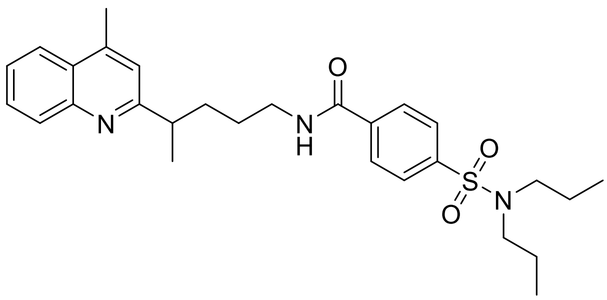

4f: yellow oil. ^{1}H NMR (400 MHz, $CDCl_3$) δ 7.97-7.92 (m, 3H), 7.88 (d, *J* = 8.4 Hz, 1H), 7.84-7.80 (m, 2H), 7.65-7.59 (m, 1H), 7.53-7.48 (m, 1H), 7.22-7.15 (m, 1H), 7.16 (s, 1H), 3.55-3.45 (m, 1H), 3.45-3.34 (m, 1H), 3.22-3.10 (m, 1H), 3.10-3.03 (m, 4H), 2.69 (s, 3H), 2.01-1.90 (m, 1H), 1.84-1.74 (m, 1H), 1.68-1.47 (m, 6H), 1.38 (d, *J* = 6.8 Hz, 3H), 0.85 (t, *J* = 7.2 Hz, 6H); ^{13}C NMR (100 MHz, $CDCl_3$) δ 166.3, 165.9, 147.2, 145.0, 142.5, 138.5, 129.2, 129.0, 127.9, 127.1, 127.1, 125.7, 123.8, 120.2, 50.0, 42.0, 40.6, 34.8, 26.8, 21.9, 21.0, 18.9, 11.2. FT-IR: ν (cm^{-1}) 3320, 2926, 2934, 2875, 1736, 1645, 1539, 1487, 1372, 1241. HRMS [ESI] calcd for $C_{28}H_{37}N_3NaO_3S$ $[M+Na]^+$ 518.2448, found 518.2446.

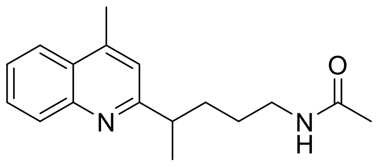

4g: yellow oil. ^{1}H NMR (400 MHz, $CDCl_3$) δ 8.04-7.99 (m, 1H), 7.96-7.91 (m, 1H), 7.68-7.62 (m, 1H), 7.52-7.46 (m, 1H), 7.12 (s, 1H), 6.12 (br, 1H), 3.28-3.11 (m, 2H), 3.10-3.00 (m, 1H), 2.67 (s, 3H), 1.93 (s, 3H), 1.90-1.80 (m, 1H), 1.74-1.63 (m, 1H), 1.55-1.44 (m, 1H), 1.44-1.36 (m, 1H), 1.34 (d, *J* = 7.2 Hz, 3H); ^{13}C NMR (100 MHz, $CDCl_3$) δ 170.1, 166.1, 147.4, 144.8, 129.2, 129.1, 127.1, 125.6, 123.7, 120.3, 42.2, 39.7, 34.4, 27.3, 23.3, 21.0, 18.9. FT-IR: ν (cm^{-1}) 3284, 3063, 2958, 2850, 1649, 1558, 1447, 1367, 1294. HRMS [ESI] calcd for $C_{17}H_{23}N_2O$ $[M+H]^+$ 271.1805, found 271.1811.

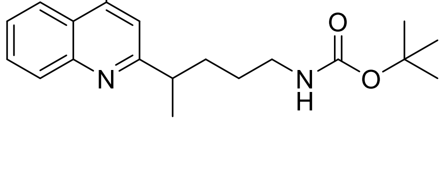

4h: yellow oil. ^{1}H NMR (400 MHz, $CDCl_3$) δ 8.03-7.99 (m, 1H), 7.97-7.31 (m, 1H), 7.67 (ddd, *J* = 8.4, 7.2, 1.6 Hz, 1H), 7.51 (ddd, *J* = 8.0, 6.8, 1.2 Hz, 1H), 7.16-7.15

(m, 1H), 5.86 (s, 1H), 3.15-3.05 (m, 1H), 2.68 (s, 3H), 2.13-1.92 (m, 5H), 1.38 (d, *J* = 6.8 Hz, 3H), 1.35-1.30 (m, 1H), 1.33 (s, 9H); ^{13}C NMR (100 MHz, $CDCl_3$) δ 172.4, 165.7, 147.3, 144.9, 129.3, 129.1, 127.1, 125.7, 123.7, 119.9, 51.0, 41.7, 35.7, 33.3, 28.8, 20.8, 18.9. FT-IR: ν (cm^{-1}) 3312, 3300, 3065, 2964, 2871, 2851, 1648, 1545, 1391, 1269. HRMS [ESI] calcd for $C_{20}H_{29}N_2O_2$ $[M+H]^+$ 329.2224, found 329.2219.

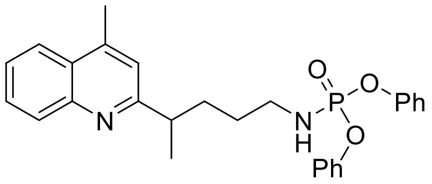

5a: yellow oil. ^{1}H NMR (400 MHz, $CDCl_3$) δ 8.06 (d, *J* = 8.4 Hz, 1H), 7.97-7.92 (m, 1H), 7.67 (ddd, *J* = 8.4, 7.2, 1.6 Hz, 1H), 7.51 (ddd, *J* = 8.4, 7.2, 1.2 Hz, 1H), 7.29-7.16 (m, 8H), 7.13-7.06 (m, 2H), 7.08 (s, 1H), 3.49-3.37 (m, 1H), 3.12-2.95 (m, 3H), 2.66 (s, 3H), 1.89-1.78 (m, 1H), 1.70-1.58 (m, 1H), 1.56-1.44 (m, 1H), 1.43-1.33 (m, 1H), 1.30 (d, *J* = 6.8 Hz, 3H); ^{13}C NMR (100 MHz, $CDCl_3$) δ 165.9, 150.9 (d, J_{C-P} = 2.3 Hz), 150.8 (d, J_{C-P} = 2.4 Hz), 150.9, 150.8, 150.8, 147.4, 144.7, 129.6, 129.4, 129.2, 127.1, 125.7, 124.8, 123.6, 120.2, 42.2, 41.8, 33.6, 29.4 (d, J_{C-P} = 6.4 Hz), 21.0, 18.9; ^{31}P NMR (161 MHz, $CDCl_3$) δ -0.6 (s). FT-IR: ν (cm^{-1}) 3227, 3063, 2959, 2871, 1591, 1488, 1379, 1254. HRMS [ESI] calcd for $C_{27}H_{30}N_2O_3P$ $[M+H]^+$ 461.1989, found 461.1996.

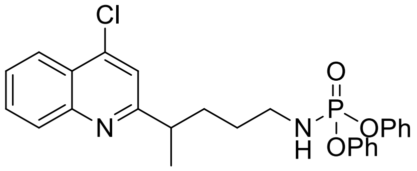

5b: yellow oil. ^{1}H NMR (400 MHz, $CDCl_3$) δ 8.20-8.16 (m, 1H), 8.06 (d, *J* = 8.4 Hz, 1H), 7.76-7.70 (m, 1H), 7.61-7.56 (m, 1H), 7.32 (s, 1H), 7.29-7.17 (m, 8H), 7.13-7.06 (m, 2H), 3.60-3.42 (m, 1H), 3.13-2.94 (m, 3H), 1.86-1.75 (m, 1H), 1.68-1.57 (m, 1H), 1.55-1.31 (m, 2H), 1.29 (d, *J* = 7.2 Hz, 3H); ^{13}C NMR (100 MHz, $CDCl_3$) δ 166.3, 150.8 (d, J_{C-P} = 2.0 Hz), 150.8 (d, J_{C-P} = 2.0 Hz), 148.5, 142.9, 130.4, 129.6, 129.3, 126.9, 125.2, 124.8, 123.3, 120.2 (d, J_{C-P} = 1.8 Hz), 120.2 (d, J_{C-P} = 1.8 Hz), 119.8, 42.2, 41.7, 33.50, 29.3 (d, J_{C-P} = 6.2 Hz), 20.8. ^{31}P NMR (161 MHz, $CDCl_3$) δ -0.6 (s). FT-IR: ν (cm^{-1}) 3229, 3068, 2963, 2873, 1589, 1489, 1376, 1220. HRMS [ESI] calcd for $C_{26}H_{27}ClN_2O_3P$ $[M+H]^+$ 481.1442, found 481.1449.

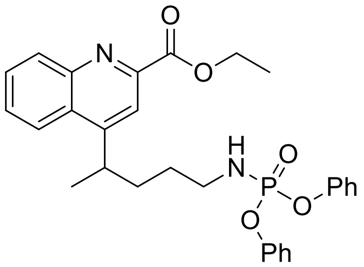

5c: yellow oil. ^{1}H NMR (400 MHz, $CDCl_3$) δ 8.32 (d, *J* = 8.4 Hz, 1H), 8.03 (d, *J* = 8.8 Hz, 1H), 8.00 (s, 1H), 7.76-7.71 (m, 1H), 7.63-7.57 (m, 1H), 7.28-7.21 (m, 4H), 7.20-7.15 (m, 4H), 7.12-7.15 (m, 2H), 4.54 (q, *J* = 7.2 Hz, 2H), 3.58-3.37 (m, 2H), 3.08-2.97 (m, 2H), 1.80-1.61 (m, 2H), 1.53-1.42 (m, 4H), 1.42-1.34 (m, 1H), 1.33-1.29 (m, 3H); ^{13}C NMR (100 MHz, $CDCl_3$) δ 165.8, 154.7, 150.8 (d, J_{C-P} = 6.6 Hz), 148.1, 147.9, 131.8, 129.7, 129.6, 128.3, 128.1, 124.7, 122.8, 120.1 (d, J_{C-P} = 3.7 Hz), 120.1 (d, J_{C-P} = 3.8 Hz), 117.5, 62.3, 41.7, 34.0, 33.3, 29.5 (d, J_{C-P} = 5.9 Hz), 21.1, 14.4; ^{31}P NMR (161 MHz, $CDCl_3$) δ -0.6 (s). FT-IR: ν (cm-1) 3227, 3069, 2965, 2874, 1716, 1559, 1488, 1369, 1243. HRMS [ESI] calcd for $C_{29}H_{32}N_2O_5P$ $[M+H]^+$ 519.2043, found 519.2033.

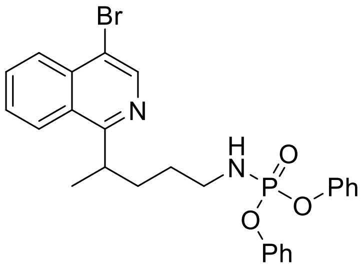

5d: yellow oil. ^{1}H NMR (400 MHz, $CDCl_3$) δ 8.65 (s, 1H), 8.20 (d, *J* = 8.4 Hz, 1H), 8.14 (d, *J* = 8.4 Hz, 1H), 7.80-7.75 (m, 1H), 7.66-7.60 (m, 1H), 7.31-7.23 (m, 4H), 7.22-7.17 (m, 4H),

7.15-7.08 (m, 2H), 3.74-3.63 (m, 1H), 3.29-3.21 (m, 1H), 3.09-2.96 (m, 2H), 2.08-1.97 (m, 1H), 1.74-1.63 (m, 1H), 1.53-1.42 (m, 1H), 1.41-1.32 (m, 1H), 1.32 (d, J = 6.8 Hz, 3H); ^{13}C NMR (100 MHz, $CDCl_3$) δ 164.7, 150.8 (d, $J_{C\text{-}P}$ = 6.5 Hz), 143.6, 134.9, 131.0, 129.6, 128.1, 128.0, 126.9, 124.9, 124.8 (d, $J_{C\text{-}P}$ = 2.6 Hz), 120.2 (t, $J_{C\text{-}P}$ = 4.4 Hz), 120.2 ($J_{C\text{-}P}$ = 4.5 Hz), 117.7, 41.9, 35.8, 32.8, 29.6 ($J_{C\text{-}P}$ = 5.9 Hz), 21.1. ^{31}P NMR (161 MHz, $CDCl_3$) δ -0.7 (s). FT-IR: ν (cm^{-1}) 3228, 3070, 2961, 2871, 2852, 1615, 1565, 1448, 1338, 1256. HRMS [ESI] calcd for $C_{26}H_{27}BrN_2O_3P$ $[M+H]^+$ 525.0937, found 525.0936.

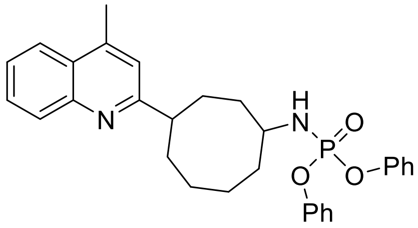

5e (dr = 1/1.4): yellow oil. ^{1}H NMR (400 MHz, $CDCl_3$) δ 8.03-7.97 (m, 1H, two isomers), 7.87-7.82 (m, 1H, two isomers), 7.62-7.55 (m, 1H, two isomers), 7.44-7.38 (m, 1H, two isomers), 7.28-7.20 (m, 8H, two isomers), 7.10-7.00 (m, 3H, two isomers), 4.20-3.85 (m, 1H, two isomers), 3.67-3.50 (m, 1H, two isomers), 3.05-2.95 (m, 1H, two isomers), 2.56 (s, 3H, two isomers), 2.10-1.67 (m, 8H, two isomers), 1.64-1.36 (m, 4H, two isomers); ^{13}C NMR (100 MHz, $CDCl_3$) δ 167.6 & 167.5 (overlap, two isomers), 151.1 (overlap, two isomers, d, $J_{C\text{-}P}$ = 6.5 Hz), 147.4 (overlap, two isomers), 144.5 (overlap, two isomers), 129.7 (overlap, two isomers), 129.5 (overlap, two isomers), 129.0 (overlap, two isomers), 126.9 (overlap, two isomers), 125.5 (overlap, two isomers), 124.7 (overlap, two isomers), 123.6 (overlap, two isomers), 120.6 (overlap, two isomers, $J_{C\text{-}P}$ = 9.8 Hz), 120.3 &120.2 (two isomers), 52.5 & 52.0 (two isomers), 48.1 & 47.6 (two isomers), 33.7 (single isomer, d, $J_{C\text{-}P}$ = 5.2 Hz) , 33.6 (single isomer, d, $J_{C\text{-}P}$ = 5.2 Hz), 33.1 (single isomer, d, $J_{C\text{-}P}$ = 4.7 Hz), 33.0 (single isomer, d, $J_{C\text{-}P}$ = 5.2 Hz), 31.8 & 31.6 (two isomers), 30.3 & 28.9 (two isomers), 27.0 & 26.4 (two isomers), 23.6 & 23.3 (two isomers), 18.8 (overlap, two isomers). ^{31}P NMR (161 MHz, $CDCl_3$) δ -1.4 (s) & -1.5 (s). FT-IR: ν (cm^{-1}) 3210, 3064, 3042, 2922, 2852, 1698, 1560, 1447, 1252. HRMS [ESI] calcd for $C_{30}H_{34}N_2O_3P$ $[M+H]^+$ 501.2302, found 501.2315.

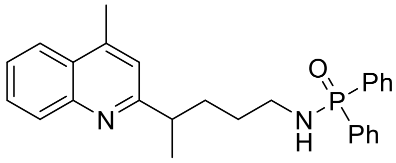

5f: yellow oil. ^{1}H NMR (400 MHz, $CDCl_3$) δ 7.95-7.91 (m, 2H), 7.87-7.79 (m, 4H), 7.65-7.09 (m, 1H), 7.51-7.34 (m, 7H), 7.08 (s, 1H), 3.07-2.08 (m, 2H), 2.97-2.88 (m, 2H), 2.65 (s, 3H), 1.95-1.84 (m, 1H), 1.73-1.53 (m, 2H), 1.50-1.38 (m, 1H), 1.33 (d, J = 7.2 Hz, 3H); ^{13}C NMR (100 MHz, $CDCl_3$) δ 166.0, 147.5, 144.5, 133.2 (d, $J_{C\text{-}P}$ = 4.2 Hz), 132.1 (d, $J_{C\text{-}P}$ = 9.3 Hz), 132.1 (d, $J_{C\text{-}P}$ = 9.3 Hz), 131.9 (d, $J_{C\text{-}P}$ = 4.4 Hz), 131.7 (d, $J_{C\text{-}P}$ = 2.4 Hz), 129.2 (d, $J_{C\text{-}P}$ = 44.1 Hz), 128.5 (d, $J_{C\text{-}P}$ = 12.5 Hz), 127.0, 125.5, 123.6, 120.4, 42.4, 40.7 (d, $J_{C\text{-}P}$ = 1.1 Hz), 33.8, 30.2 (d, $J_{C\text{-}P}$ = 7.0 Hz), 21.0, 18.8; ^{31}P NMR (161 MHz, $CDCl_3$) δ 23.5 (s). FT-IR: ν (cm^{-1}) 3356, 3196, 3059, 2959, 2854, 1719, 1661, 1561, 1438, 1379, 1263. HRMS [ESI] calcd for $C_{27}H_{30}N_2OP$ $[M+H]^+$ 429.2090, found 429.2101.

7. ^{1}H, ^{13}C, ^{19}F, ^{31}P NMR spectra

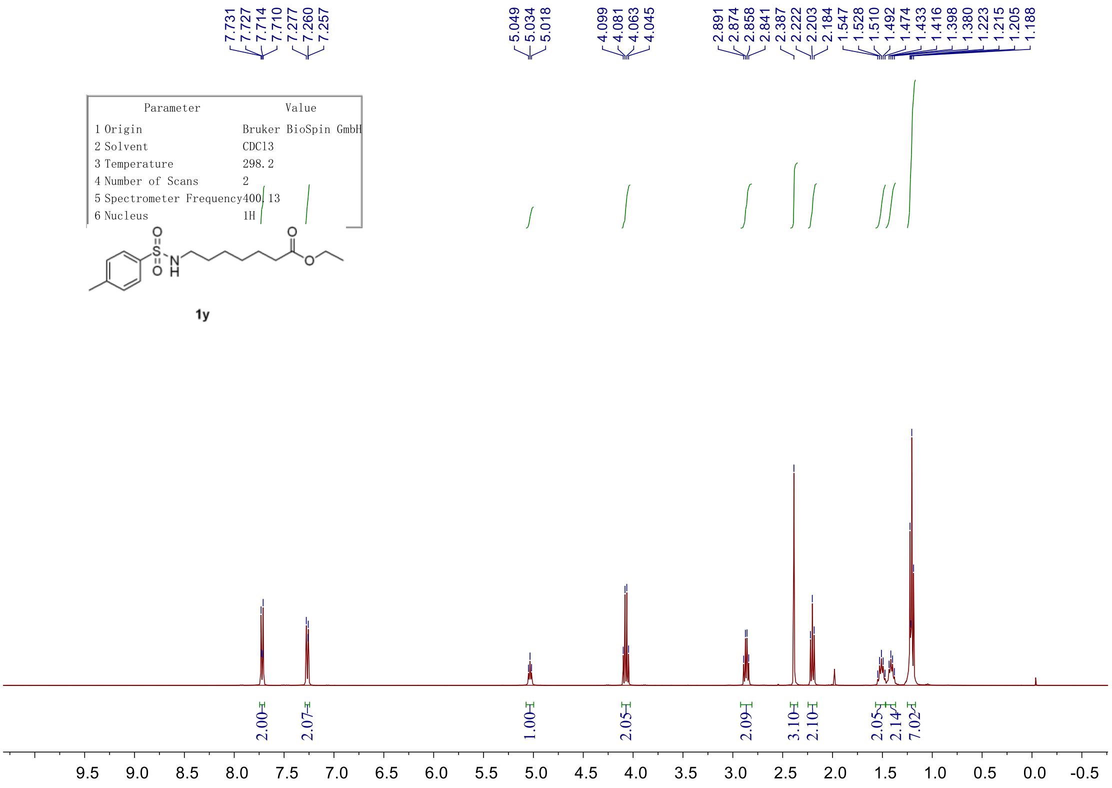
7.731 7.727 7.714 7.710 7.277 7.260 7.257
5.049 5.034 5.018
4.099 4.081 4.063 4.045
2.891 2.874 2.858 2.841 2.387 2.222 2.203 2.184 1.547 1.528 1.510 1.492 1.474 1.433 1.416 1.398 1.380 1.223 1.215 1.205 1.188
Parameter Value
1 Origin Bruker BioSpin GmbH
2 Solvent CDCl3
3 Temperature 298.2
4 Number of Scans 2
5 Spectrometer Frequency 400.13
6 Nucleus 1H
1y
2.00 2.07 1.00 2.05 2.09 3.10 2.10 2.05 2.14 7.02
9.5 9.0 8.5 8.0 7.5 7.0 6.5 6.0 5.5 5.0 4.5 4.0 3.5 3.0 2.5 2.0 1.5 1.0 0.5 0.0 -0.5

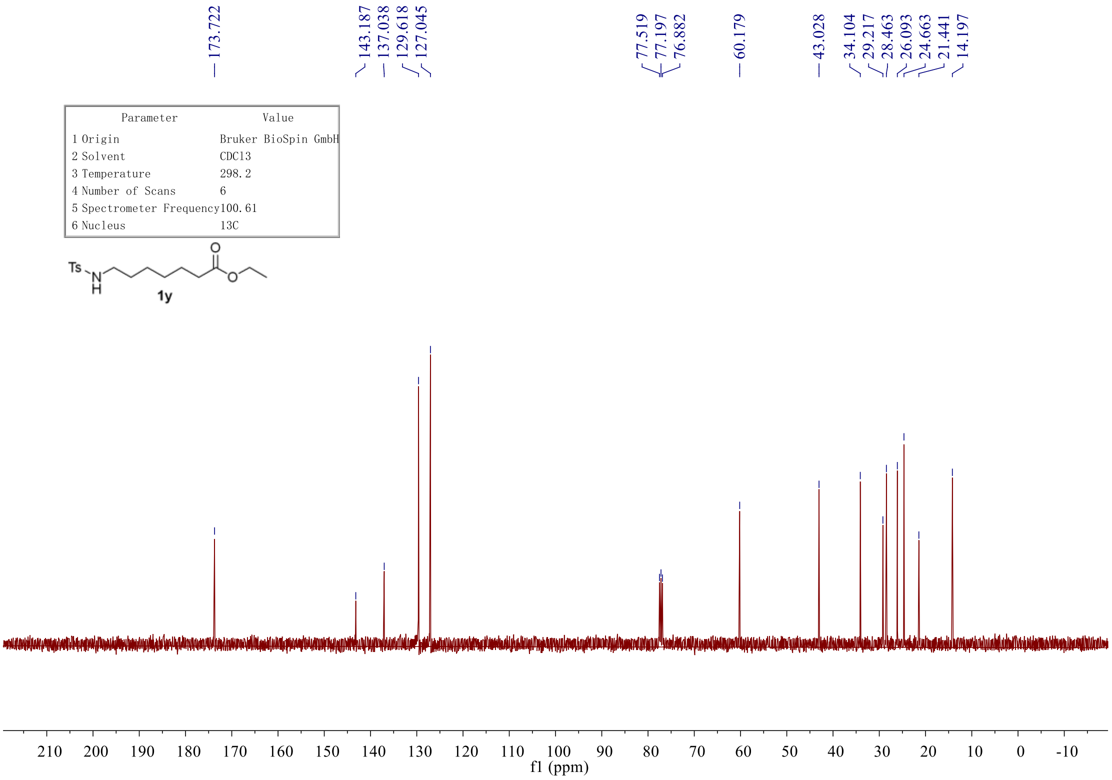
173.722
143.187 137.038 129.618 127.045
77.519 77.197 76.882
60.179
43.028
34.104 29.217 28.463 26.093 24.663 21.441 14.197
Parameter Value
1 Origin Bruker BioSpin GmbH
2 Solvent CDCl3
3 Temperature 298.2
4 Number of Scans 6
5 Spectrometer Frequency 100.61
6 Nucleus 13C
Ts
1y
210 200 190 180 170 160 150 140 130 120 110 100 90 80 70 60 50 40 30 20 10 0 -10
f1 (ppm)

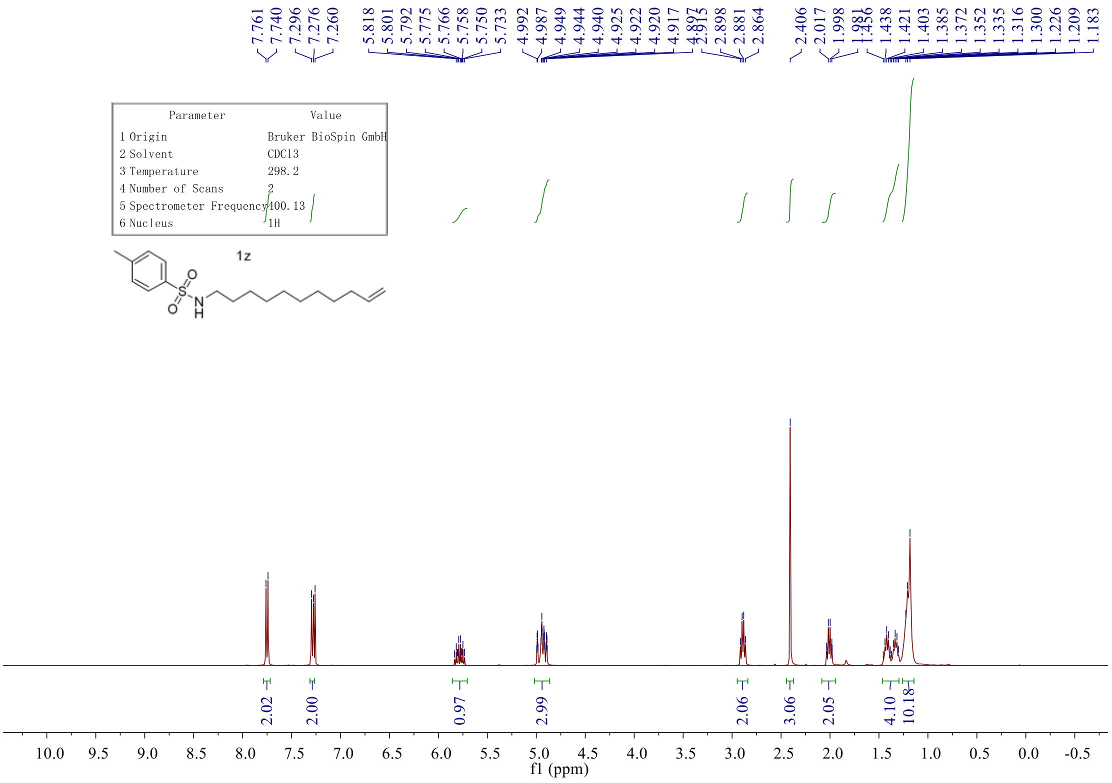

Parameter Value
1 Origin Bruker BioSpin GmbH
2 Solvent CDCl3
3 Temperature 298.2
4 Number of Scans 2
5 Spectrometer Frequency 400.13
6 Nucleus 1H
1z
f1 (ppm)

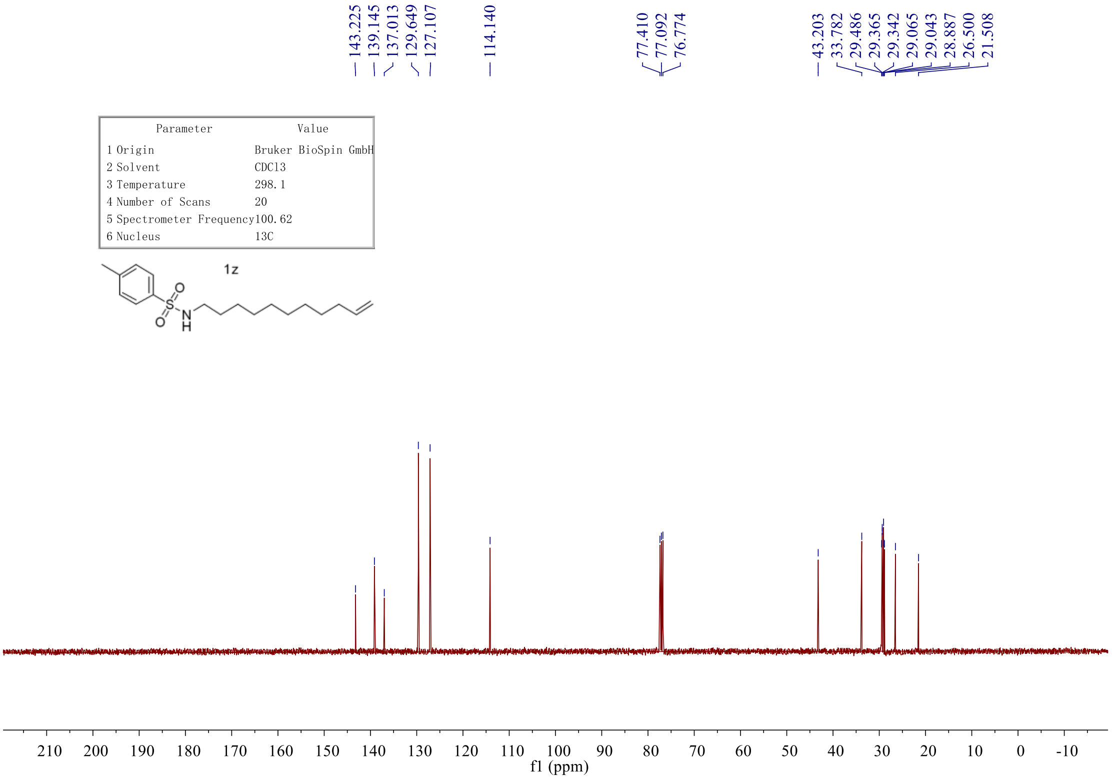

Parameter Value
1 Origin Bruker BioSpin GmbH
2 Solvent CDCl3
3 Temperature 298.1
4 Number of Scans 20
5 Spectrometer Frequency 100.62
6 Nucleus 13C
1z
f1 (ppm)

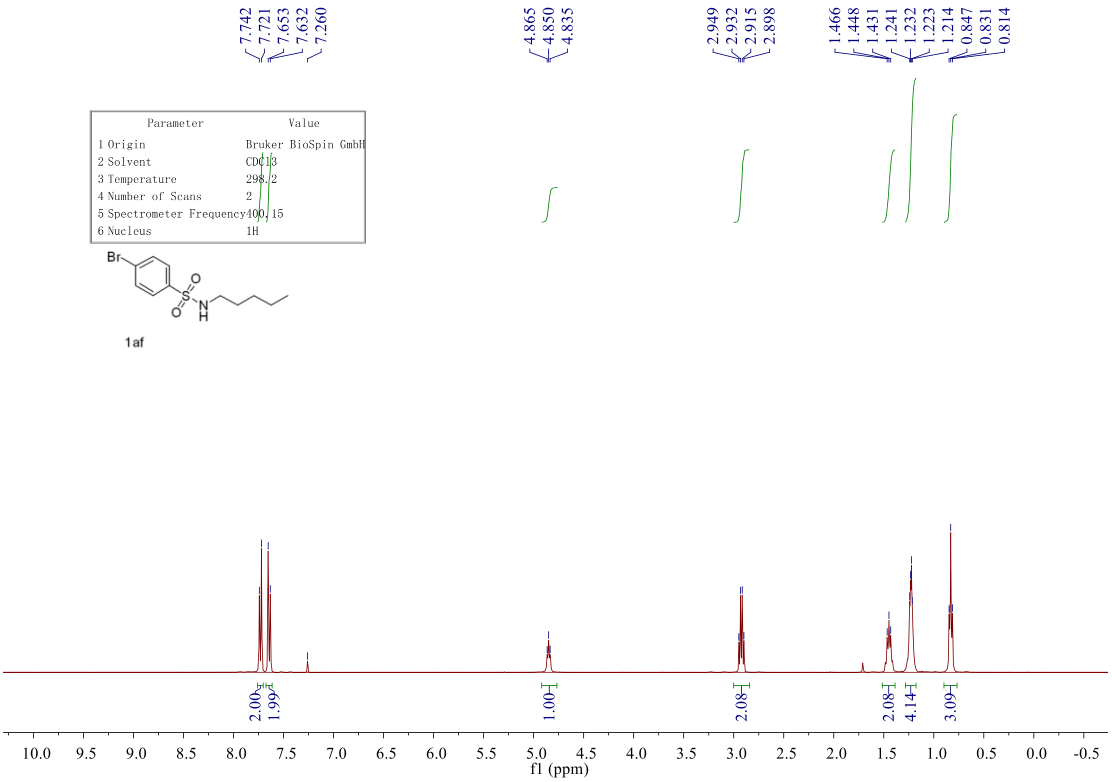

Parameter	Value
1 Origin	Bruker BioSpin GmbH
2 Solvent	CDCl3
3 Temperature	298.2
4 Number of Scans	2
5 Spectrometer Frequency	400.15
6 Nucleus	1H

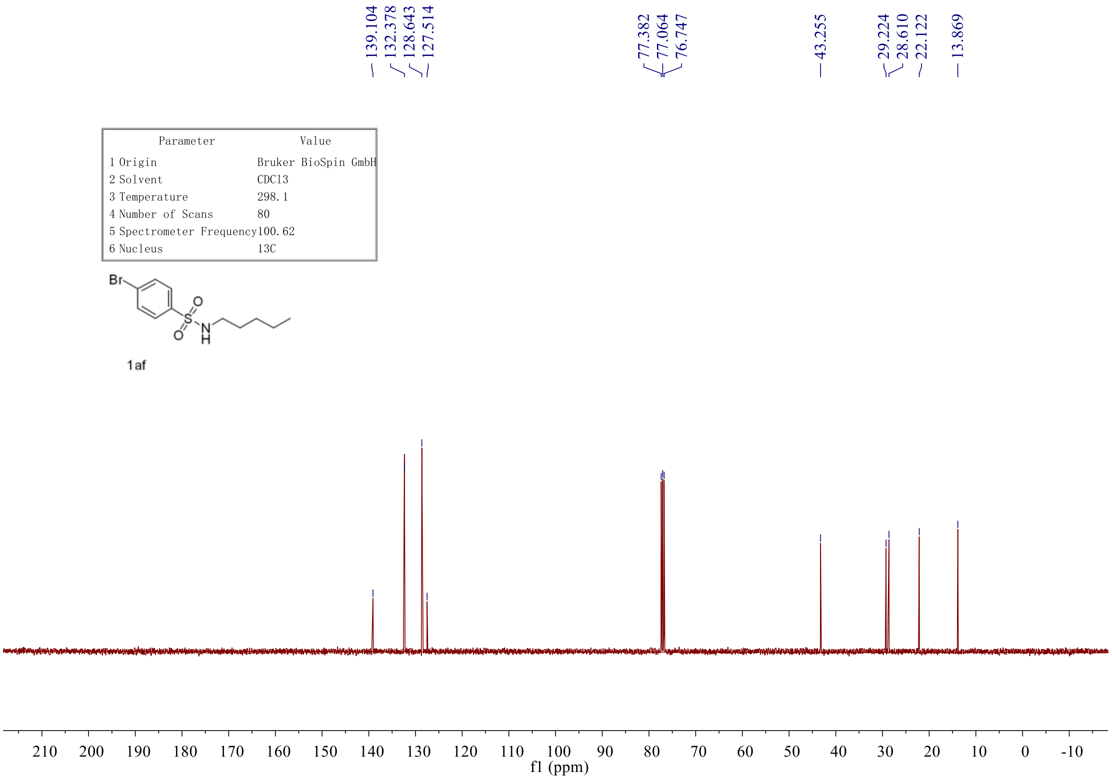

Parameter	Value
1 Origin	Bruker BioSpin GmbH
2 Solvent	CDCl3
3 Temperature	298.1
4 Number of Scans	80
5 Spectrometer Frequency	100.62
6 Nucleus	13C

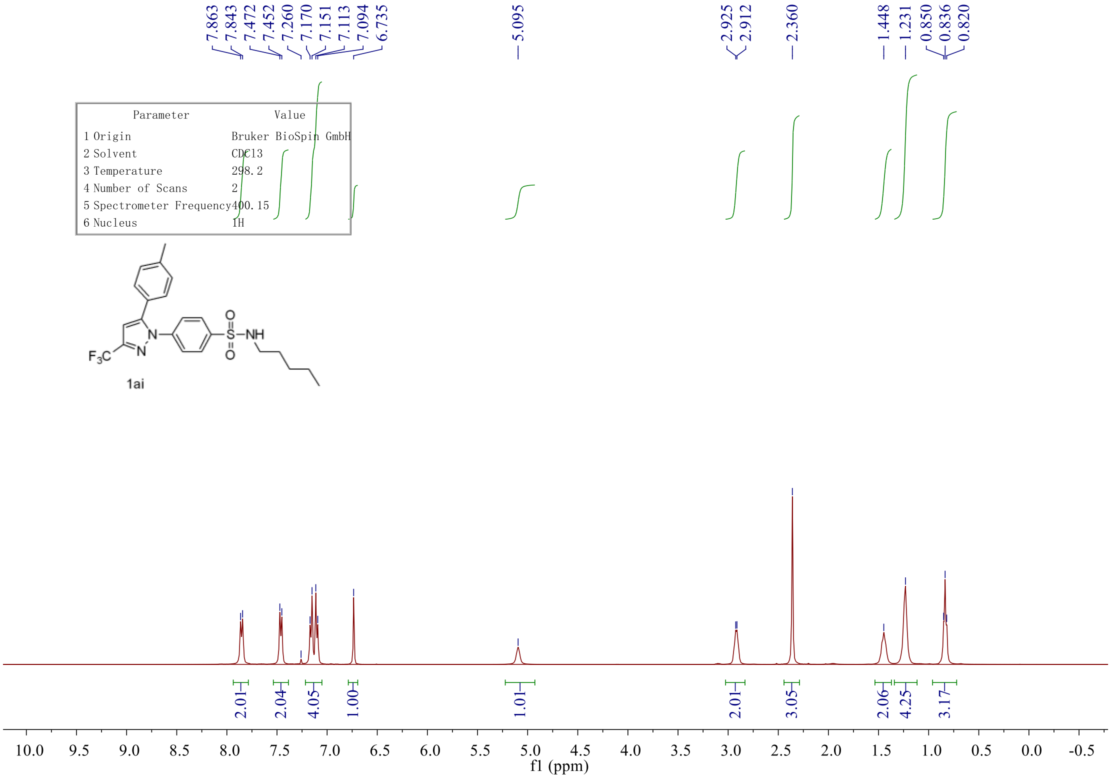

Parameter	Value
1 Origin	Bruker BioSpin GmbH
2 Solvent	CDCl3
3 Temperature	298.2
4 Number of Scans	2
5 Spectrometer Frequency	400.15
6 Nucleus	1H

1ai

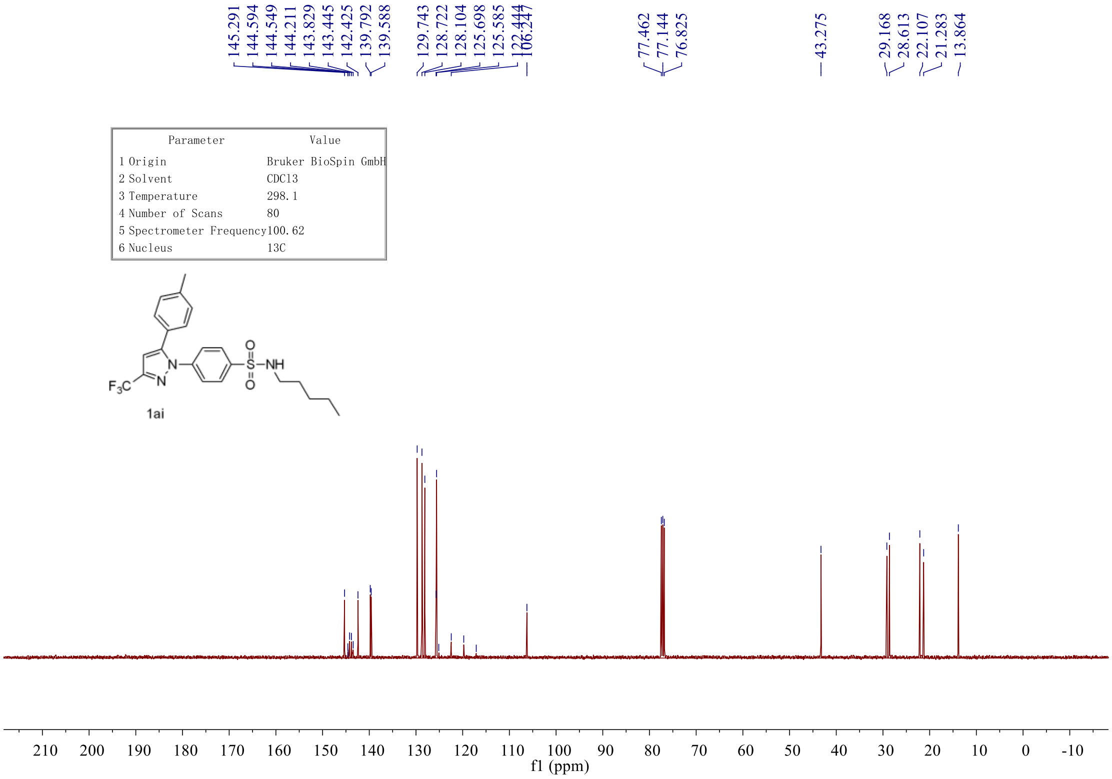

Parameter	Value
1 Origin	Bruker BioSpin GmbH
2 Solvent	CDCl3
3 Temperature	298.1
4 Number of Scans	80
5 Spectrometer Frequency	100.62
6 Nucleus	13C

1ai

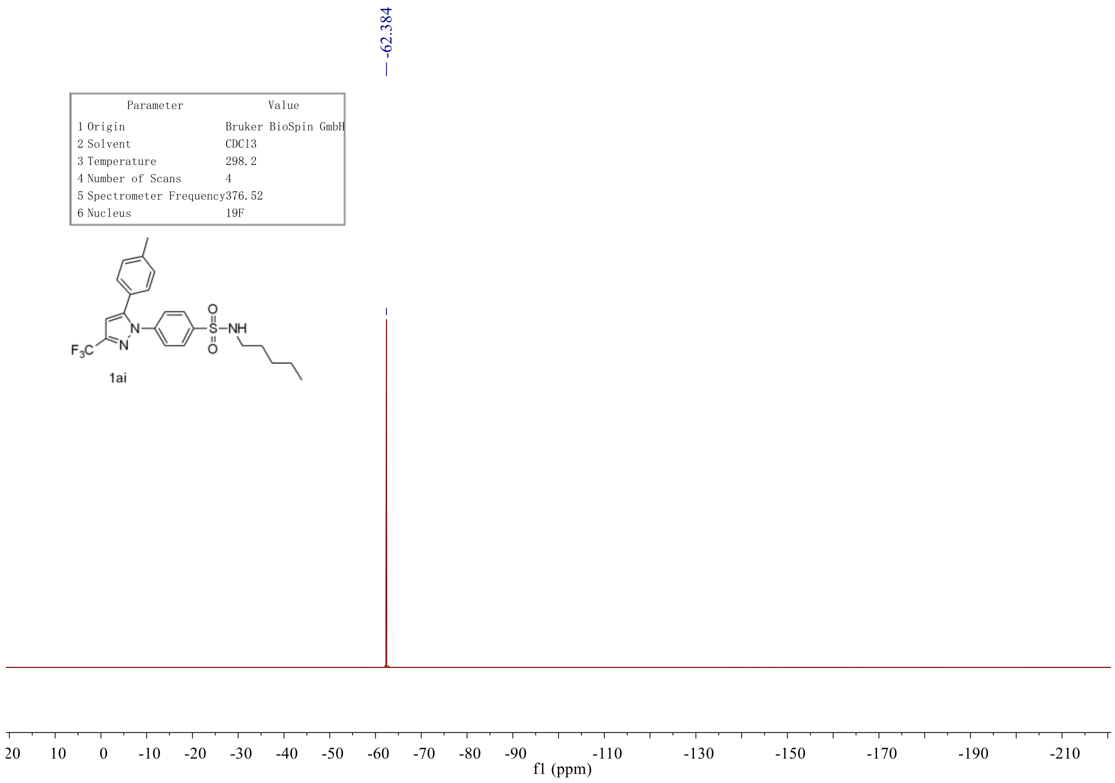
-62.384
Parameter | Value
1 Origin | Bruker BioSpin GmbH
2 Solvent | CDCl3
3 Temperature | 298.2
4 Number of Scans | 4
5 Spectrometer Frequency | 376.52
6 Nucleus | 19F
1ai
20 10 0 -10 -20 -30 -40 -50 -60 -70 -80 -90 -110 -130 -150 -170 -190 -210
f1 (ppm)

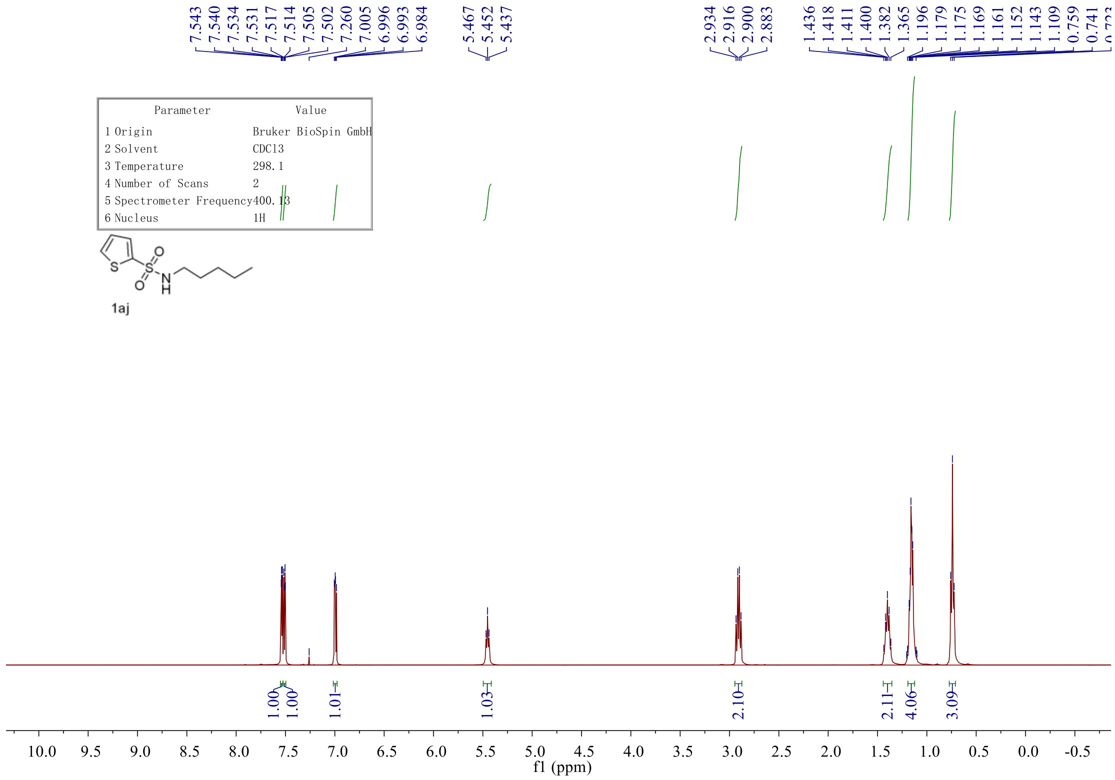
7.543 7.540 7.534 7.531 7.517 7.514 7.505 7.502 7.260 7.005 6.996 6.993 6.984
5.467 5.452 5.437
2.934 2.916 2.900 2.883
1.436 1.418 1.411 1.400 1.382 1.365 1.196 1.179 1.175 1.169 1.161 1.152 1.143 1.109 0.759 0.741
Parameter | Value
1 Origin | Bruker BioSpin GmbH
2 Solvent | CDCl3
3 Temperature | 298.1
4 Number of Scans | 2
5 Spectrometer Frequency | 400.13
6 Nucleus | 1H
1aj
1.00 1.00 1.01 1.03 2.10 2.11 4.06 3.09
10.0 9.5 9.0 8.5 8.0 7.5 7.0 6.5 6.0 5.5 5.0 4.5 4.0 3.5 3.0 2.5 2.0 1.5 1.0 0.5 0.0 -0.5
f1 (ppm)

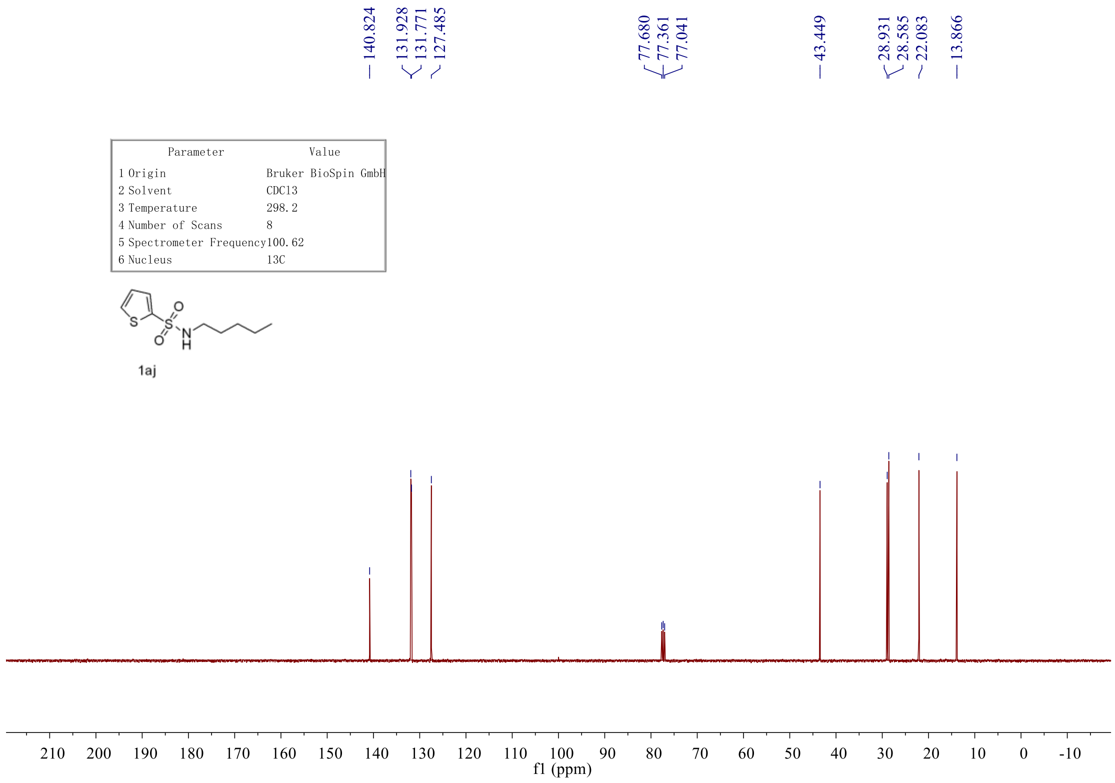
140.824
131.928
131.771
127.485
77.680
77.361
77.041
43.449
28.931
28.585
22.083
13.866
Parameter Value
1 Origin Bruker BioSpin GmbH
2 Solvent CDCl3
3 Temperature 298.2
4 Number of Scans 8
5 Spectrometer Frequency 100.62
6 Nucleus 13C
1aj
210 200 190 180 170 160 150 140 130 120 110 100 90 80 70 60 50 40 30 20 10 0 -10
f1 (ppm)

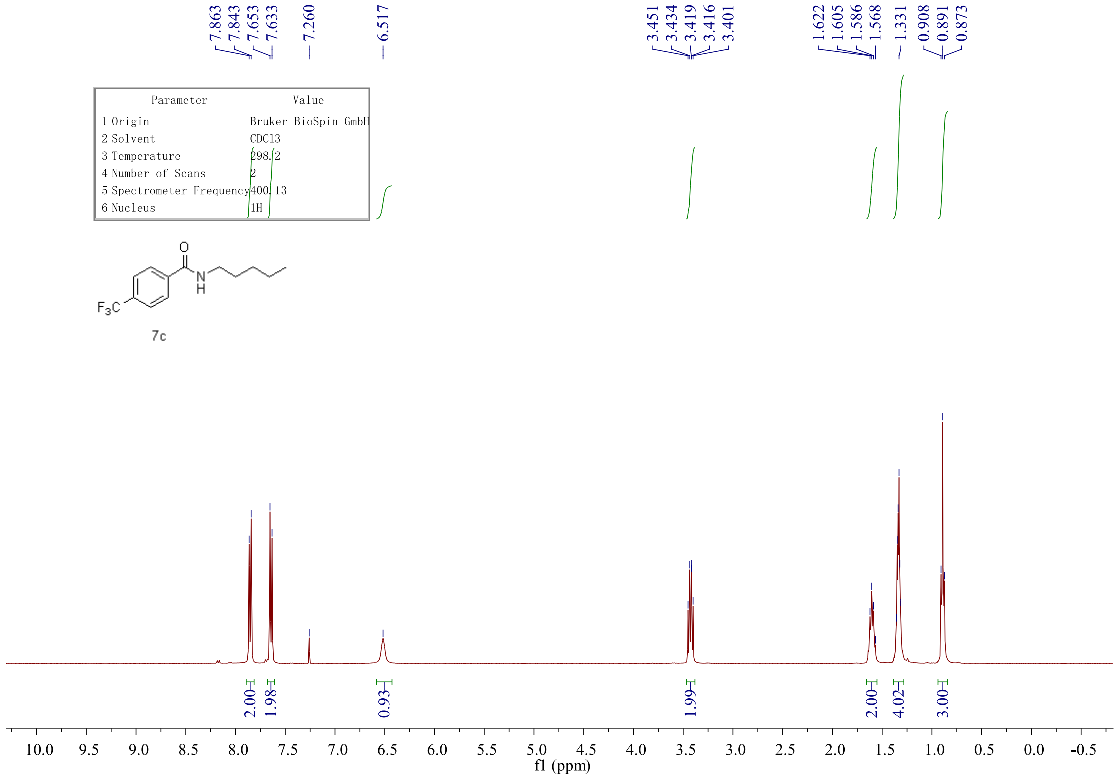
7.863
7.843
7.653
7.633
7.260
6.517
3.451
3.434
3.419
3.416
3.401
1.622
1.605
1.586
1.568
1.331
0.908
0.891
0.873
Parameter Value
1 Origin Bruker BioSpin GmbH
2 Solvent CDCl3
3 Temperature 298.2
4 Number of Scans 2
5 Spectrometer Frequency 400.13
6 Nucleus 1H
7c
2.00
1.98
0.93
1.99
2.00
4.02
3.00
10.0 9.5 9.0 8.5 8.0 7.5 7.0 6.5 6.0 5.5 5.0 4.5 4.0 3.5 3.0 2.5 2.0 1.5 1.0 0.5 0.0 -0.5
f1 (ppm)

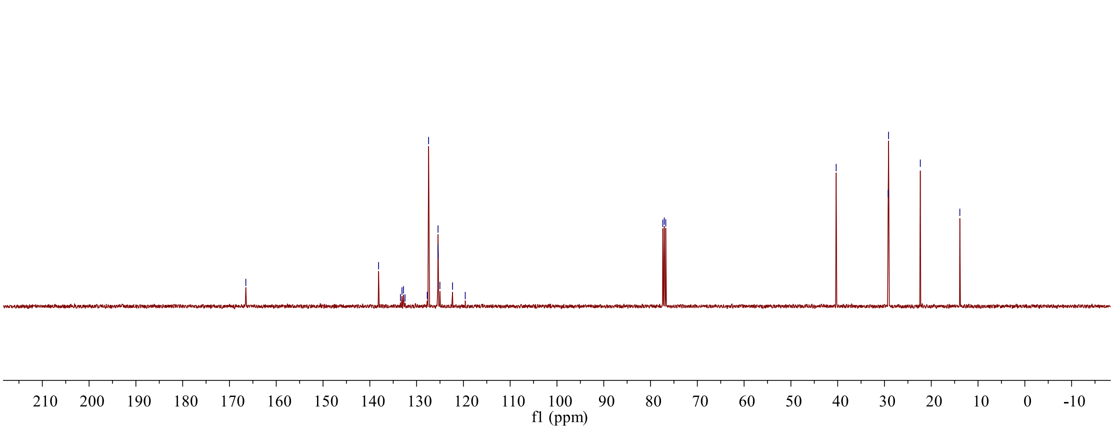

Parameter	Value
1 Origin	Bruker BioSpin GmbH
2 Solvent	CDCl3
3 Temperature	298.1
4 Number of Scans	80
5 Spectrometer Frequency	100.62
6 Nucleus	13C

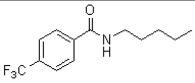

7c

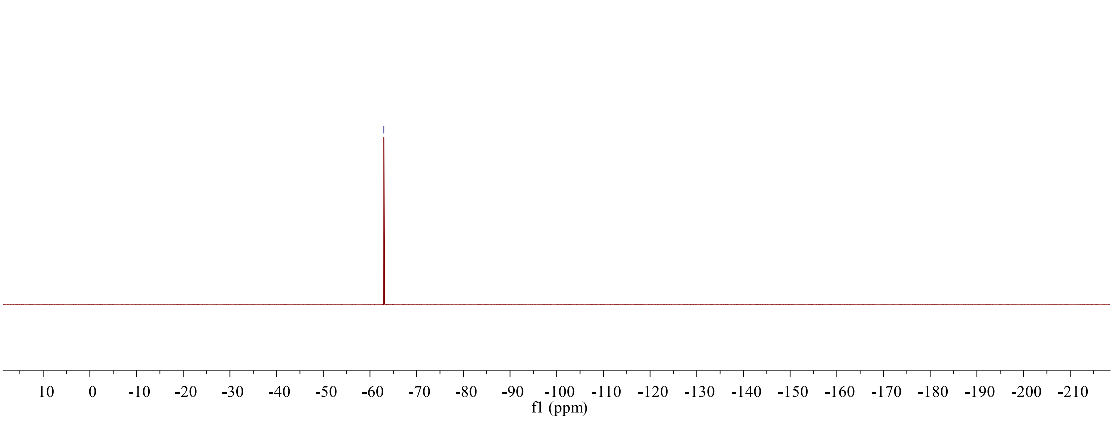

Parameter	Value
1 Origin	Bruker BioSpin GmbH
2 Solvent	CDCl3
3 Temperature	298.2
4 Number of Scans	4
5 Spectrometer Frequency	376.50
6 Nucleus	19F

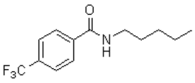

7c

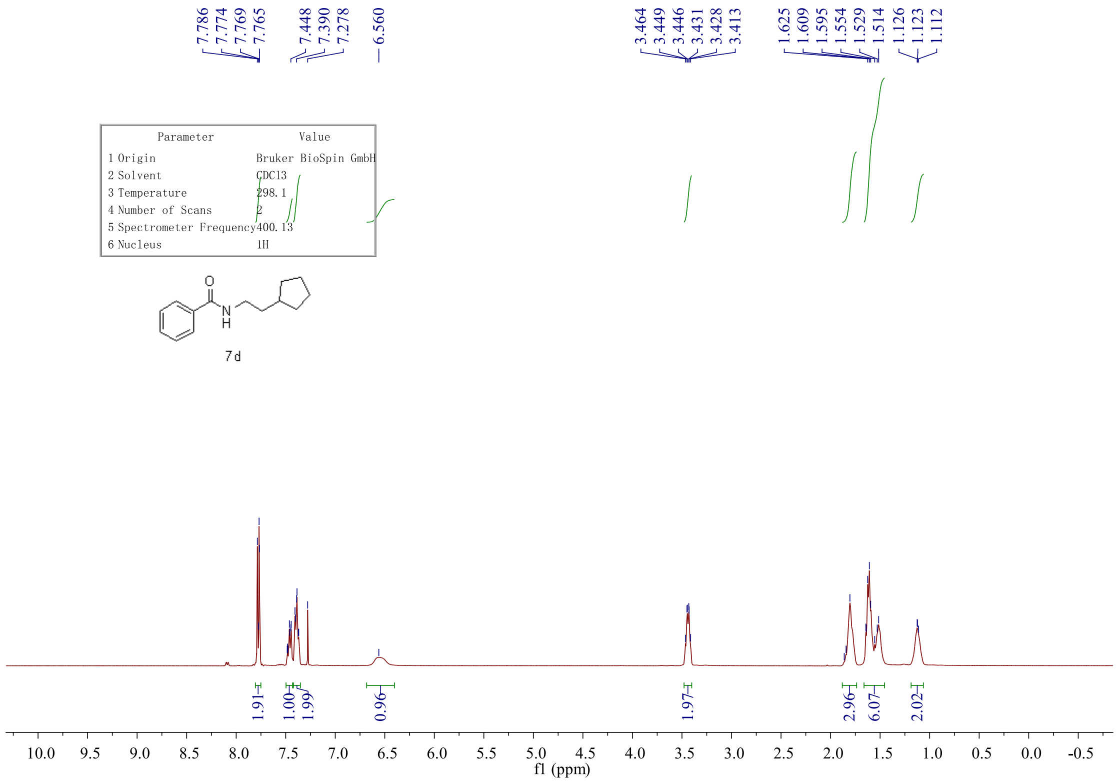
7.786
7.774
7.769
7.765
7.448
7.390
7.278
6.560
3.464
3.449
3.446
3.431
3.428
3.413
1.625
1.609
1.595
1.554
1.529
1.514
1.126
1.123
1.112
Parameter Value
1 Origin Bruker BioSpin GmbH
2 Solvent CDCl3
3 Temperature 298.1
4 Number of Scans 2
5 Spectrometer Frequency 400.13
6 Nucleus 1H
O
N
H
7d
1.91
1.00
1.99
0.96
1.97
2.96
6.07
2.02
10.0 9.5 9.0 8.5 8.0 7.5 7.0 6.5 6.0 5.5 5.0 4.5 4.0 3.5 3.0 2.5 2.0 1.5 1.0 0.5 0.0 -0.5
f1 (ppm)

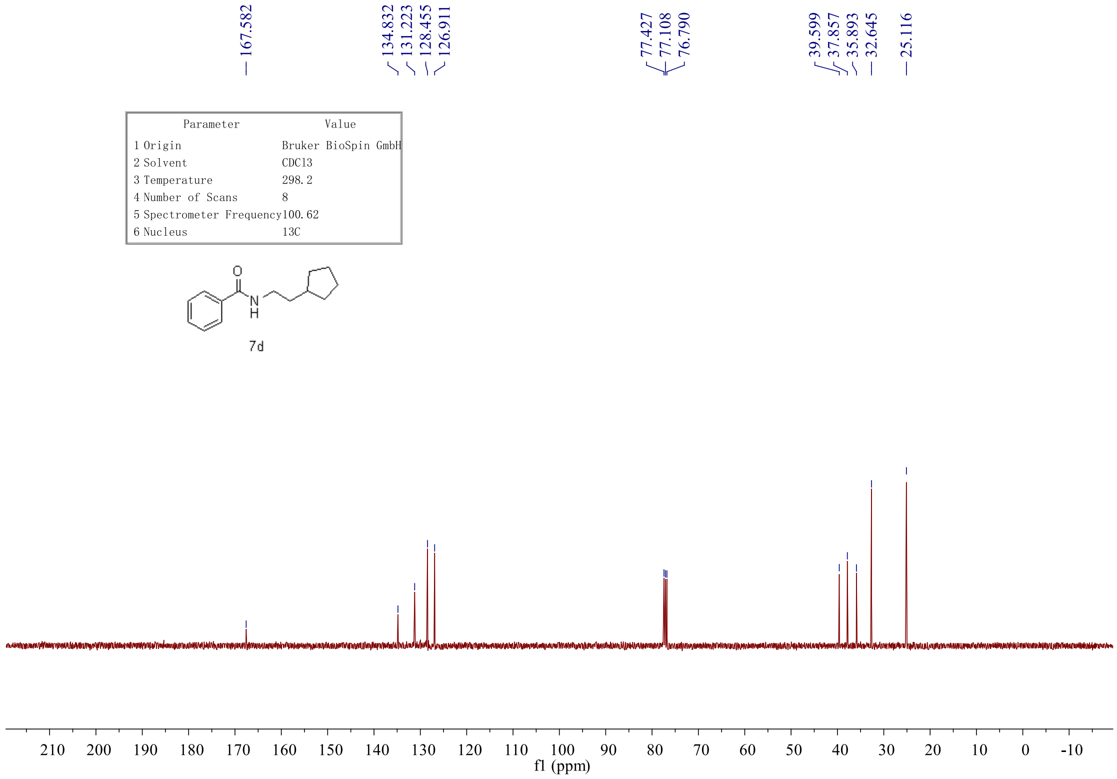
167.582
134.832
131.223
128.455
126.911
77.427
77.108
76.790
39.599
37.857
35.893
32.645
25.116
Parameter Value
1 Origin Bruker BioSpin GmbH
2 Solvent CDCl3
3 Temperature 298.2
4 Number of Scans 8
5 Spectrometer Frequency 100.62
6 Nucleus 13C
O
N
H
7d
210 200 190 180 170 160 150 140 130 120 110 100 90 80 70 60 50 40 30 20 10 0 -10
f1 (ppm)

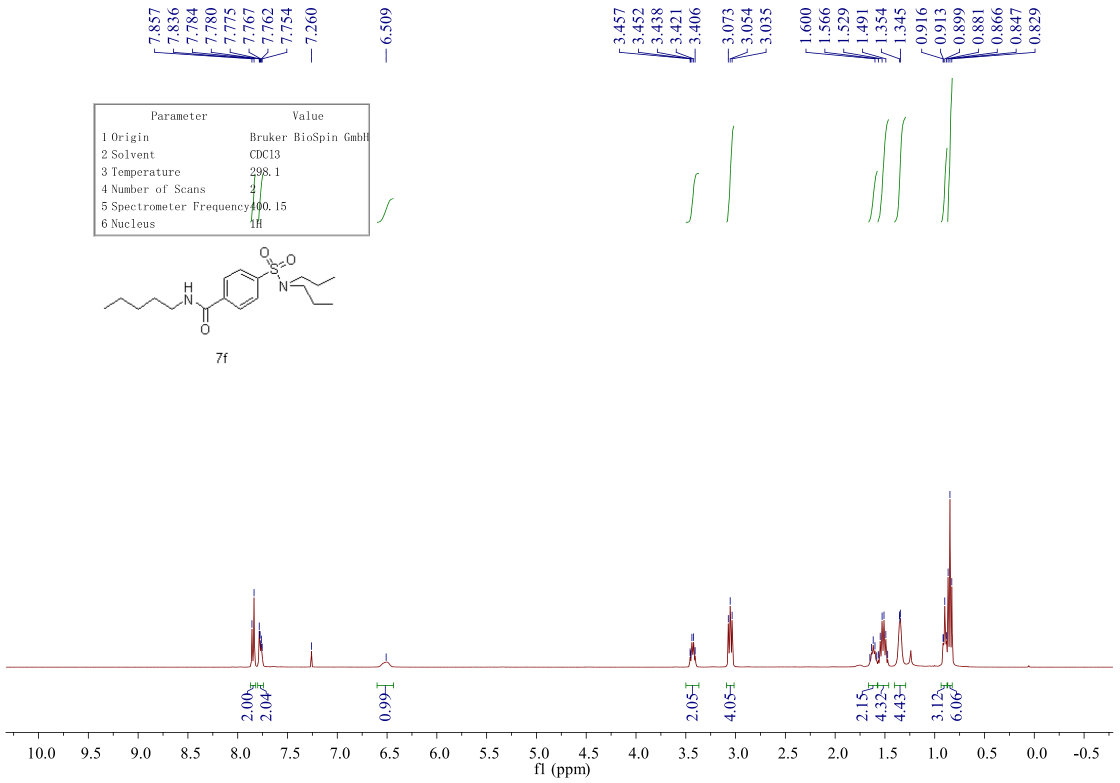
7.857
7.836
7.784
7.780
7.775
7.767
7.762
7.754
7.260
6.509
3.457
3.452
3.438
3.421
3.406
3.073
3.054
3.035
1.600
1.566
1.529
1.491
1.354
1.345
0.916
0.913
0.899
0.881
0.866
0.847
0.829
Parameter Value
1 Origin Bruker BioSpin GmbH
2 Solvent CDCl3
3 Temperature 298.1
4 Number of Scans 2
5 Spectrometer Frequency 400.15
6 Nucleus 1H
7f
2.00
2.04
0.99
2.05
4.05
2.15
4.32
4.43
3.12
6.06
10.0 9.5 9.0 8.5 8.0 7.5 7.0 6.5 6.0 5.5 5.0 4.5 4.0 3.5 3.0 2.5 2.0 1.5 1.0 0.5 0.0 -0.5
f1 (ppm)

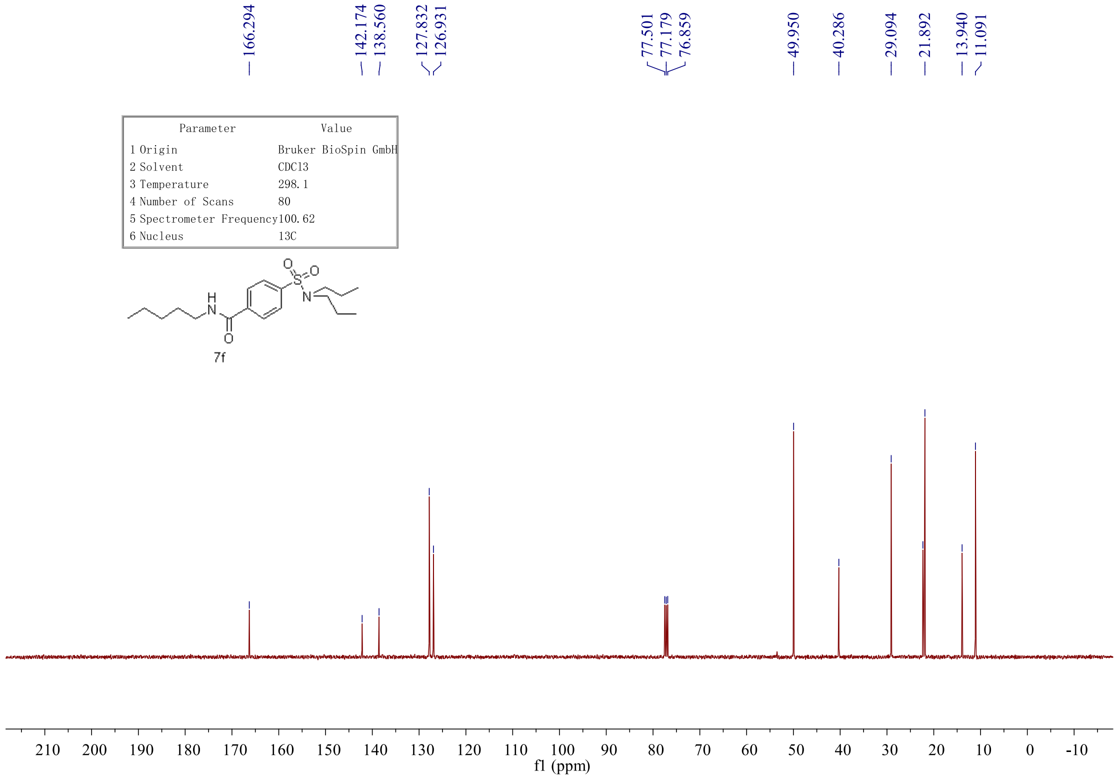
166.294
142.174
138.560
127.832
126.931
77.501
77.179
76.859
49.950
40.286
29.094
21.892
13.940
11.091
Parameter Value
1 Origin Bruker BioSpin GmbH
2 Solvent CDCl3
3 Temperature 298.1
4 Number of Scans 80
5 Spectrometer Frequency 100.62
6 Nucleus 13C
7f
210 200 190 180 170 160 150 140 130 120 110 100 90 80 70 60 50 40 30 20 10 0 -10
f1 (ppm)

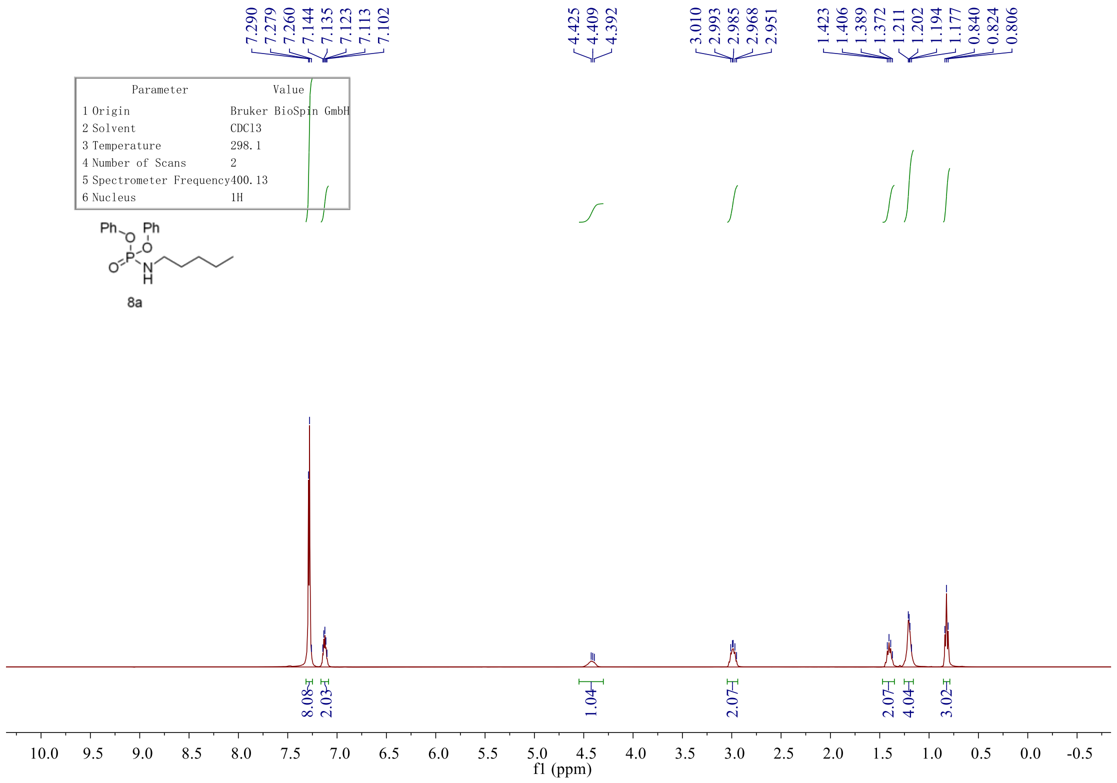
7.290
7.279
7.260
7.144
7.135
7.123
7.113
7.102
4.425
4.409
4.392
3.010
2.993
2.985
2.968
2.951
1.423
1.406
1.389
1.372
1.211
1.202
1.194
1.177
0.840
0.824
0.806
Parameter Value
1 Origin Bruker BioSpin GmbH
2 Solvent CDCl3
3 Temperature 298.1
4 Number of Scans 2
5 Spectrometer Frequency 400.13
6 Nucleus 1H
Ph O Ph O P O N H
8a
8.08
2.03
1.04
2.07
2.07
4.04
3.02
10.0 9.5 9.0 8.5 8.0 7.5 7.0 6.5 6.0 5.5 5.0 4.5 4.0 3.5 3.0 2.5 2.0 1.5 1.0 0.5 0.0 -0.5
f1 (ppm)

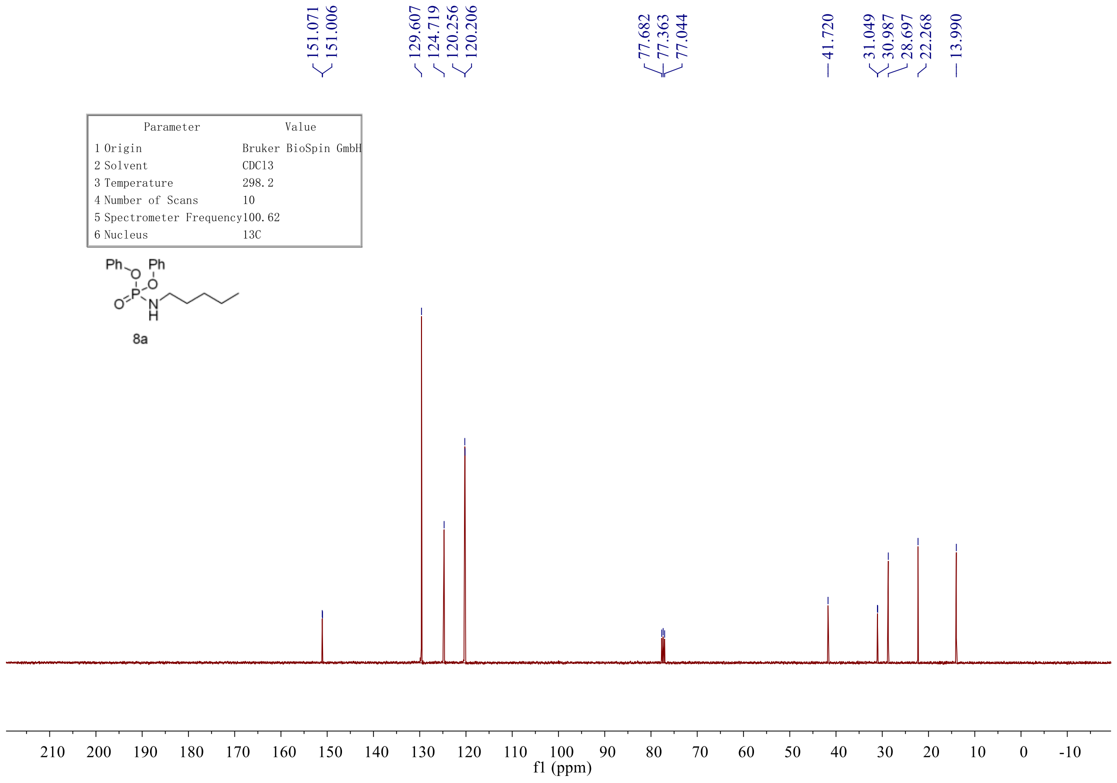
151.071
151.006
129.607
124.719
120.256
120.206
77.682
77.363
77.044
41.720
31.049
30.987
28.697
22.268
13.990
Parameter Value
1 Origin Bruker BioSpin GmbH
2 Solvent CDCl3
3 Temperature 298.2
4 Number of Scans 10
5 Spectrometer Frequency 100.62
6 Nucleus 13C
Ph O Ph O P O N H
8a
210 200 190 180 170 160 150 140 130 120 110 100 90 80 70 60 50 40 30 20 10 0 -10
f1 (ppm)

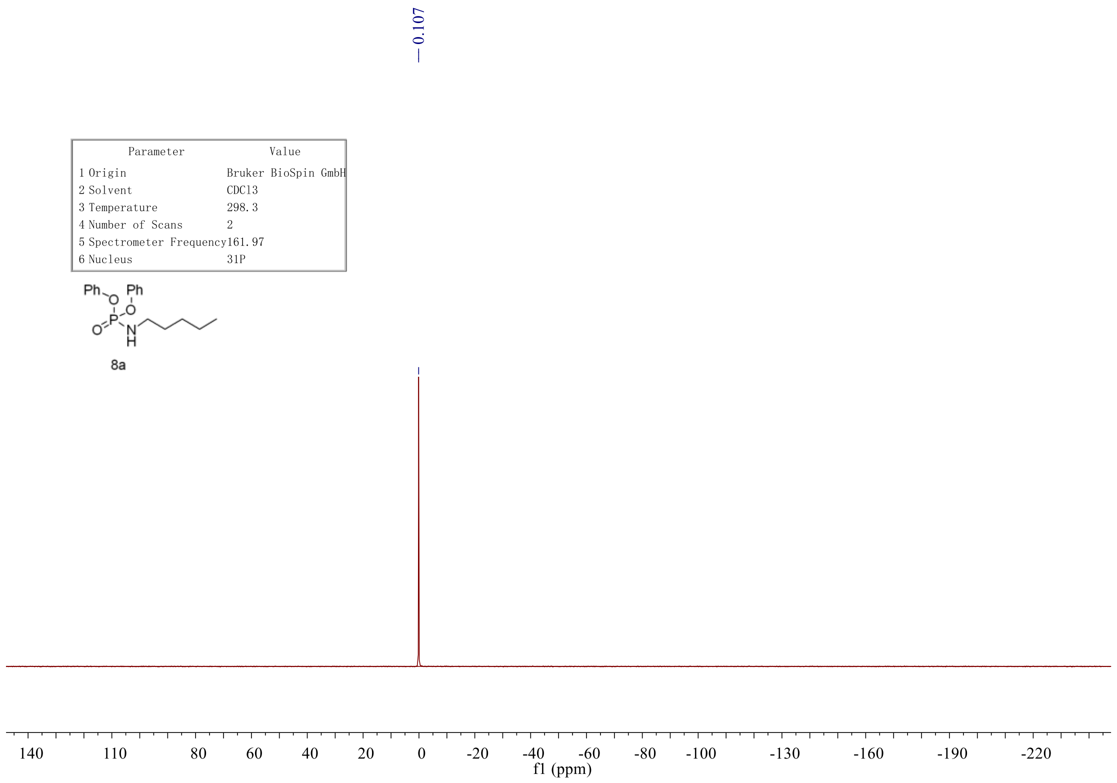
0.107
Parameter
Value
1 Origin
Bruker BioSpin GmbH
2 Solvent
CDCl3
3 Temperature
298.3
4 Number of Scans
2
5 Spectrometer Frequency
161.97
6 Nucleus
31P
Ph
O
Ph
O
P
O
N
H
8a
140
110
80
60
40
20
0
-20
-40
-60
-80
-100
-130
-160
-190
-220
f1 (ppm)

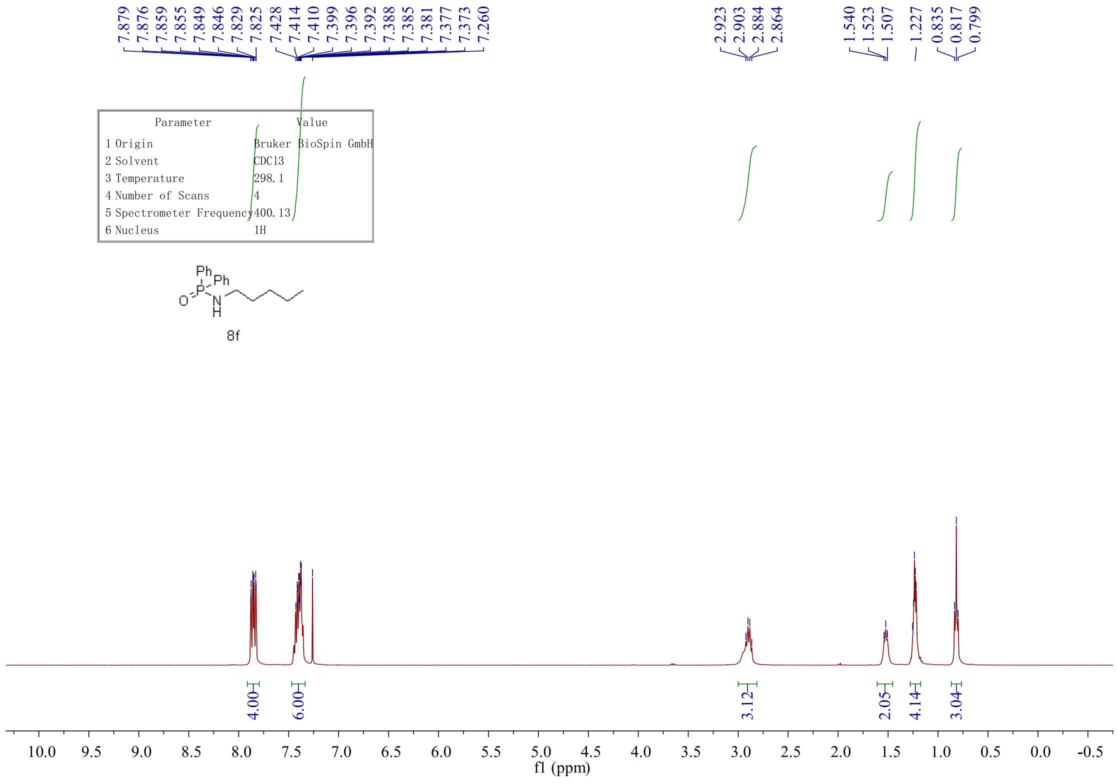
7.879
7.876
7.859
7.855
7.849
7.846
7.829
7.825
7.428
7.414
7.410
7.399
7.396
7.392
7.388
7.385
7.381
7.377
7.373
7.260
2.923
2.903
2.884
2.864
1.540
1.523
1.507
1.227
0.835
0.817
0.799
Parameter
Value
1 Origin
Bruker BioSpin GmbH
2 Solvent
CDCl3
3 Temperature
298.1
4 Number of Scans
4
5 Spectrometer Frequency
400.13
6 Nucleus
1H
Ph
Ph
P
O
N
H
8f
4.00
6.00
3.12
2.05
4.14
3.04
10.0
9.5
9.0
8.5
8.0
7.5
7.0
6.5
6.0
5.5
5.0
4.5
4.0
3.5
3.0
2.5
2.0
1.5
1.0
0.5
0.0
-0.5
f1 (ppm)

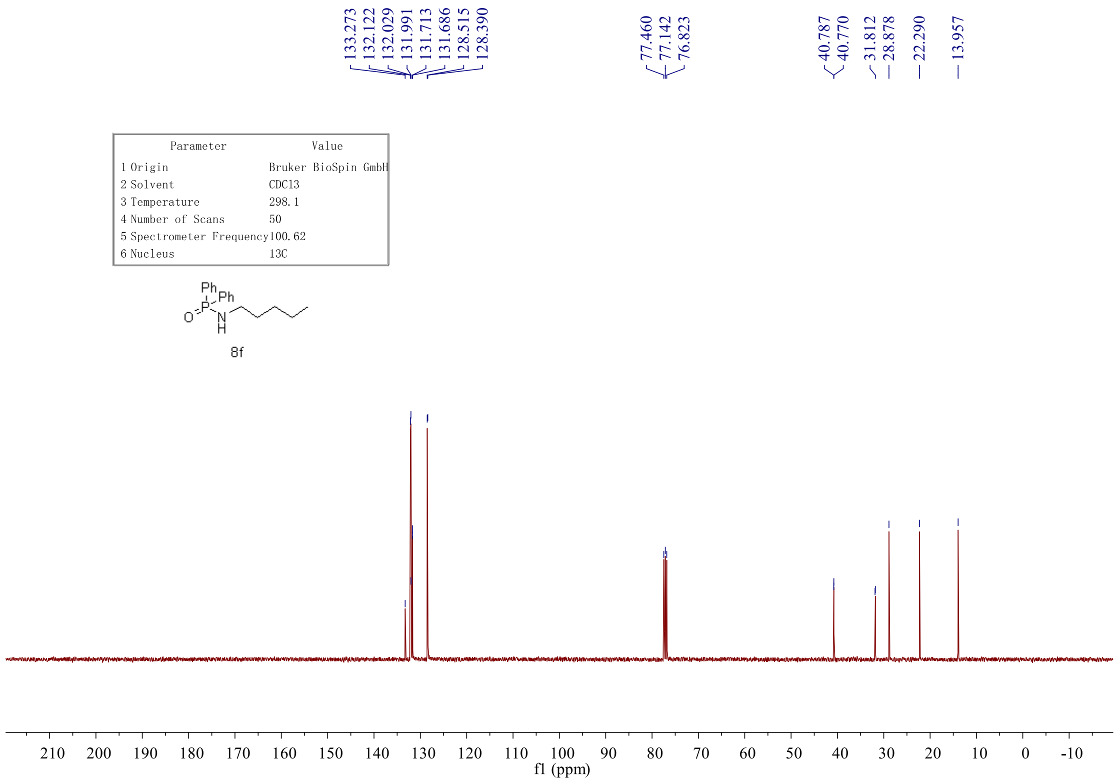
133.273
132.122
132.029
131.991
131.713
131.686
128.515
128.390
77.460
77.142
76.823
40.787
40.770
31.812
28.878
22.290
13.957
Parameter Value
1 Origin Bruker BioSpin GmbH
2 Solvent CDCl3
3 Temperature 298.1
4 Number of Scans 50
5 Spectrometer Frequency 100.62
6 Nucleus 13C
Ph
Ph
O
P
N
H
8f
210 200 190 180 170 160 150 140 130 120 110 100 90 80 70 60 50 40 30 20 10 0 -10
f1 (ppm)

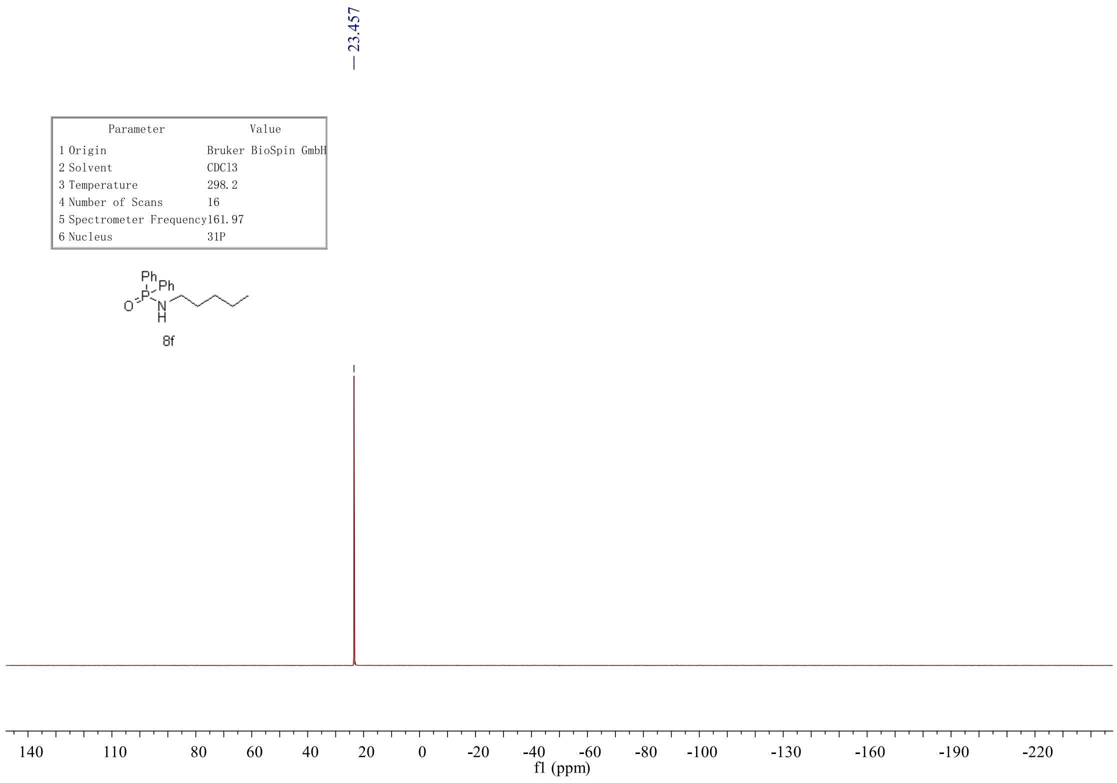
23.457
Parameter Value
1 Origin Bruker BioSpin GmbH
2 Solvent CDCl3
3 Temperature 298.2
4 Number of Scans 16
5 Spectrometer Frequency 161.97
6 Nucleus 31P
Ph
Ph
O
P
N
H
8f
140 110 80 60 40 20 0 -20 -40 -60 -80 -100 -130 -160 -190 -220
f1 (ppm)

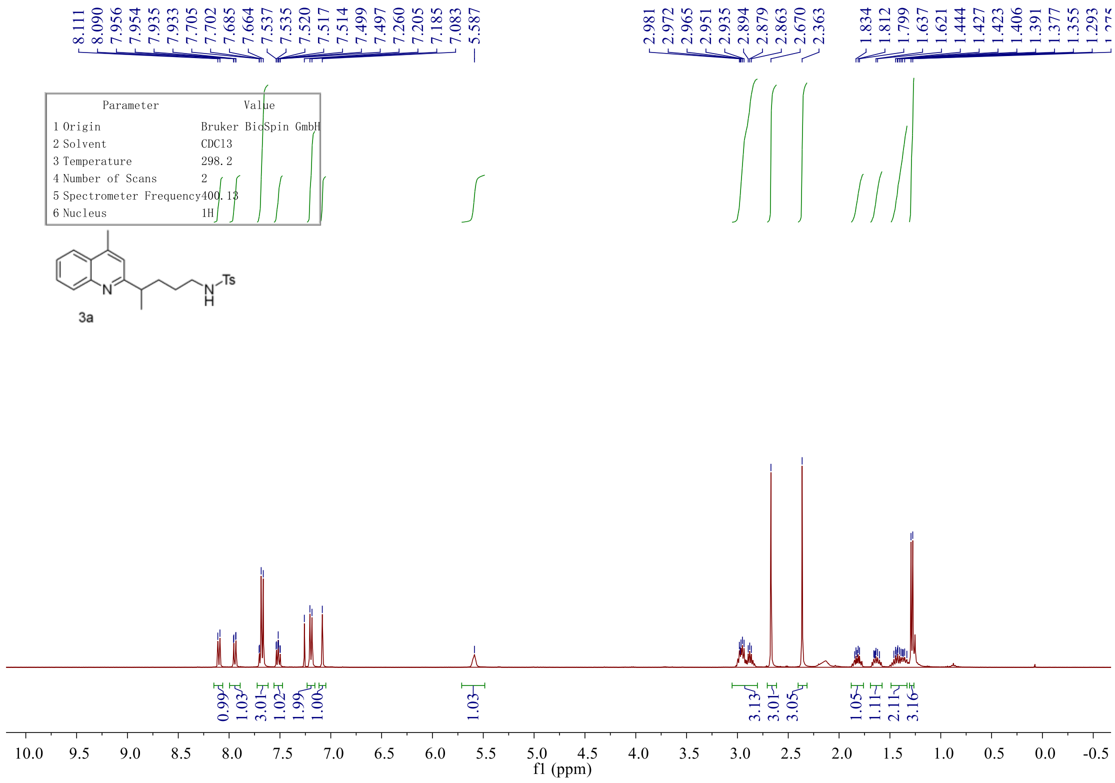
Parameter Value
1 Origin Bruker BioSpin GmbH
2 Solvent CDCl3
3 Temperature 298.2
4 Number of Scans 2
5 Spectrometer Frequency 400.13
6 Nucleus 1H
3a
f1 (ppm)

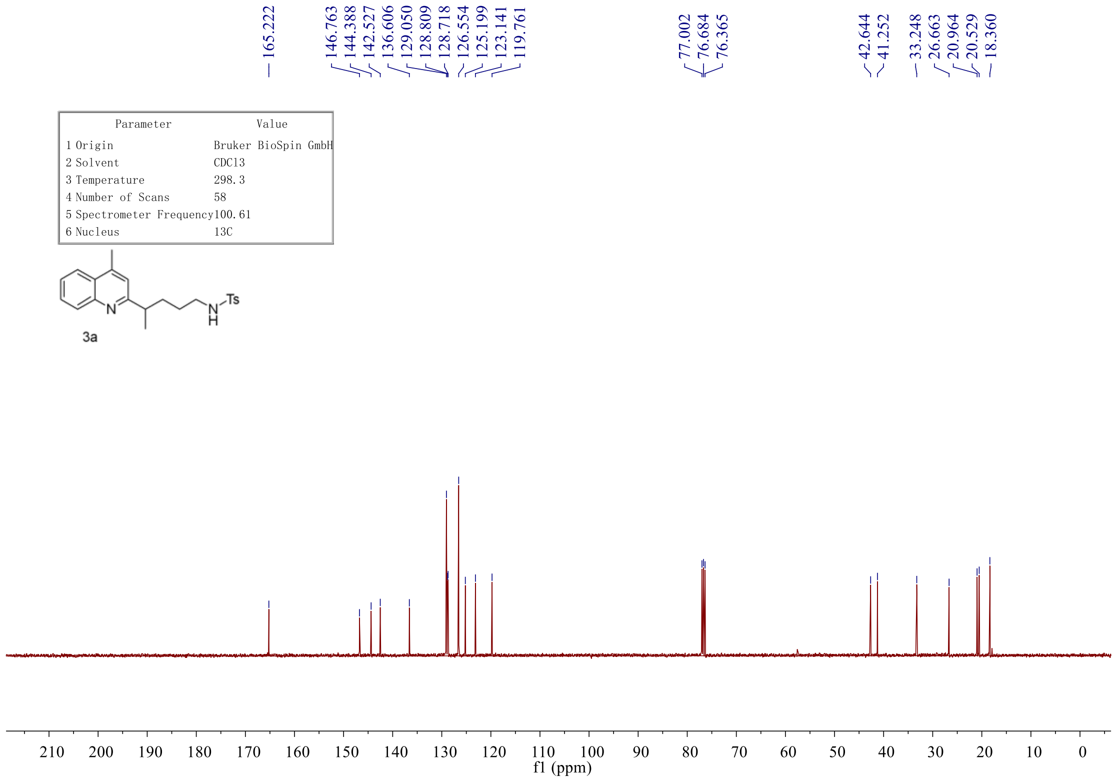
Parameter Value
1 Origin Bruker BioSpin GmbH
2 Solvent CDCl3
3 Temperature 298.3
4 Number of Scans 58
5 Spectrometer Frequency 100.61
6 Nucleus 13C
3a
f1 (ppm)

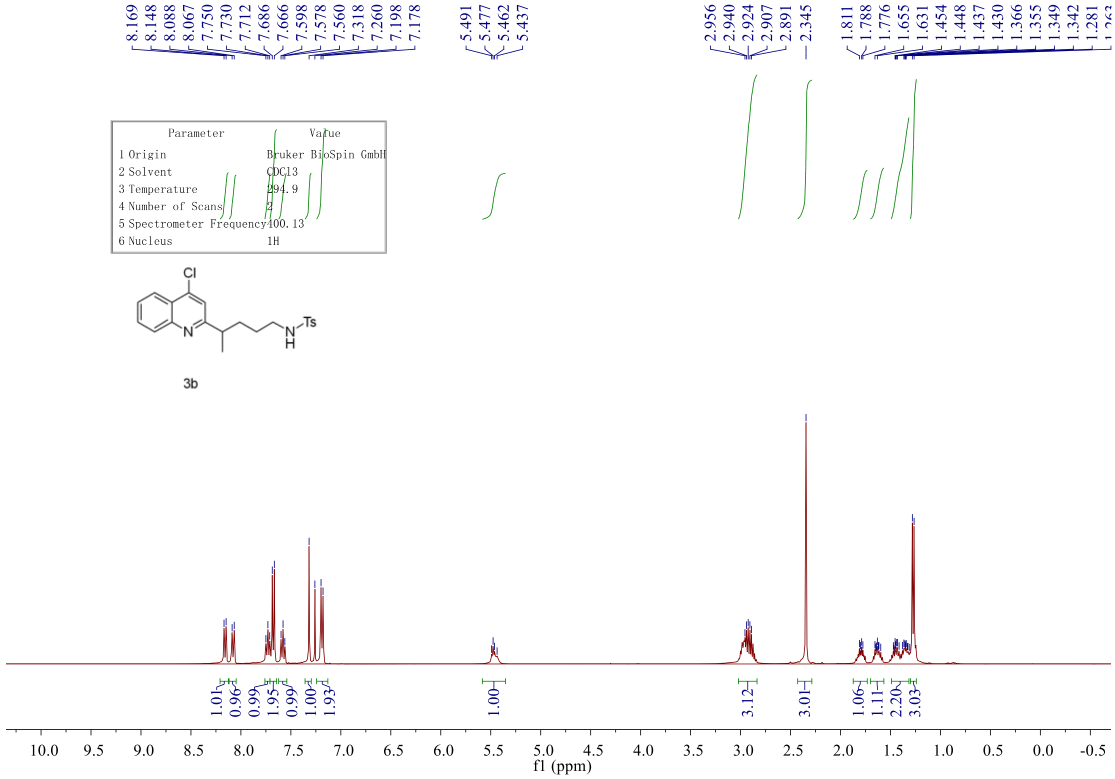
8.169 8.148 8.088 8.067 7.750 7.730 7.712 7.686 7.666 7.598 7.578 7.560 7.318 7.260 7.198 7.178
5.491 5.477 5.462 5.437
2.956 2.940 2.924 2.907 2.891 2.345
1.811 1.788 1.776 1.655 1.631 1.454 1.448 1.437 1.430 1.366 1.355 1.349 1.342 1.281 [illegible]
Parameter Value
1 Origin Bruker BioSpin GmbH
2 Solvent CDCl3
3 Temperature 294.9
4 Number of Scans 2
5 Spectrometer Frequency 400.13
6 Nucleus 1H
Cl
N
N
H
Ts
3b
1.01 0.96 0.99 1.95 0.99 1.00 1.93 1.00 3.12 3.01 1.06 1.11 2.20 3.03
10.0 9.5 9.0 8.5 8.0 7.5 7.0 6.5 6.0 5.5 5.0 4.5 4.0 3.5 3.0 2.5 2.0 1.5 1.0 0.5 0.0 -0.5
f1 (ppm)

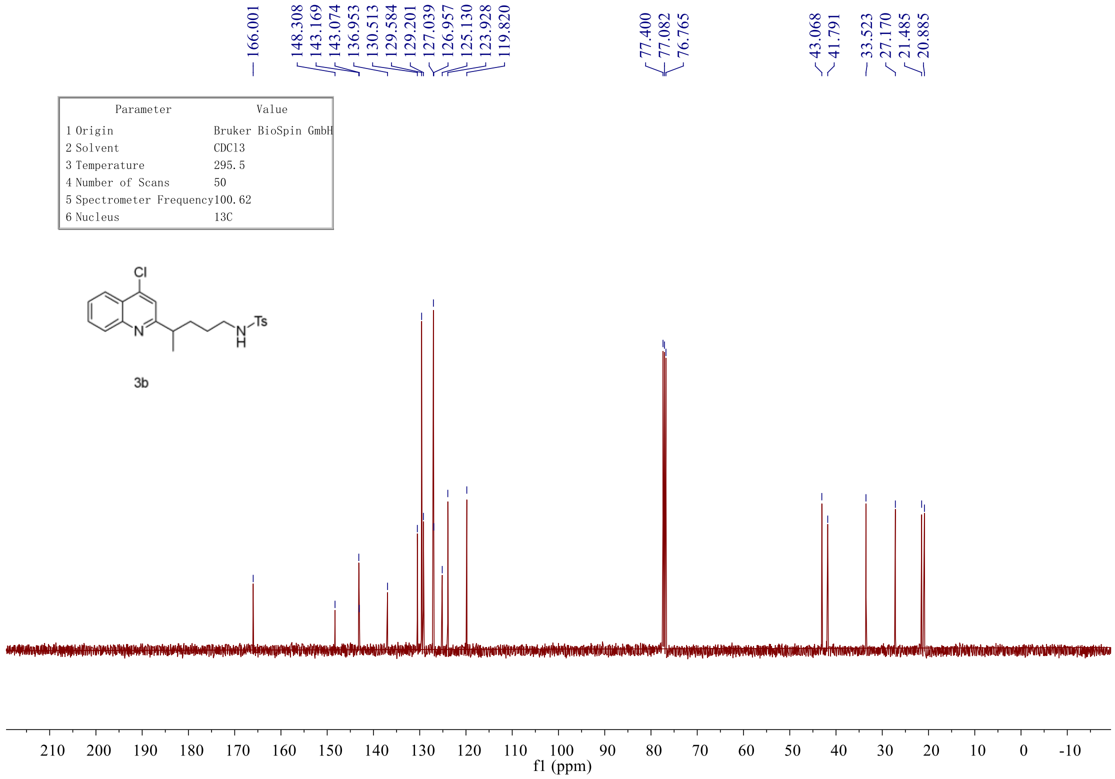
166.001
148.308 143.169 143.074 136.953 130.513 129.584 129.201 127.039 126.957 125.130 123.928 119.820
77.400 77.082 76.765
43.068 41.791 33.523 27.170 21.485 20.885
Parameter Value
1 Origin Bruker BioSpin GmbH
2 Solvent CDCl3
3 Temperature 295.5
4 Number of Scans 50
5 Spectrometer Frequency 100.62
6 Nucleus 13C
Cl
N
N
H
Ts
3b
210 200 190 180 170 160 150 140 130 120 110 100 90 80 70 60 50 40 30 20 10 0 -10
f1 (ppm)

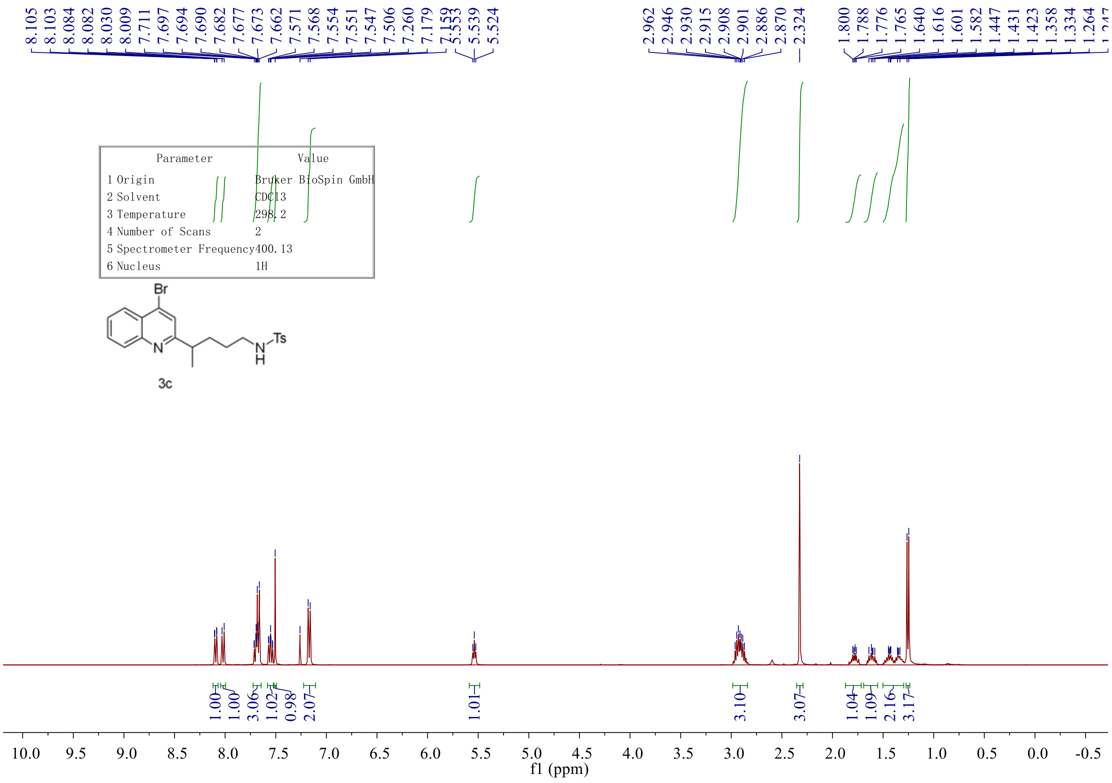
8.105 8.103 8.084 8.082 8.030 8.009 7.711 7.697 7.694 7.690 7.682 7.677 7.673 7.662 7.571 7.568 7.554 7.551 7.547 7.506 7.260 7.179 7.159 5.553 5.539 5.524
2.962 2.946 2.930 2.915 2.908 2.901 2.886 2.870 2.324
1.800 1.788 1.776 1.765 1.640 1.616 1.601 1.582 1.447 1.431 1.423 1.358 1.334 1.264
Parameter Value
1 Origin Bruker BioSpin GmbH
2 Solvent CDCl3
3 Temperature 298.2
4 Number of Scans 2
5 Spectrometer Frequency 400.13
6 Nucleus 1H
Br
N
N
H
Ts
3c
1.00 1.00 3.06 1.02 0.98 2.07 1.01 3.10 3.07 1.04 1.09 2.16 3.17
10.0 9.5 9.0 8.5 8.0 7.5 7.0 6.5 6.0 5.5 5.0 4.5 4.0 3.5 3.0 2.5 2.0 1.5 1.0 0.5 0.0 -0.5
f1 (ppm)

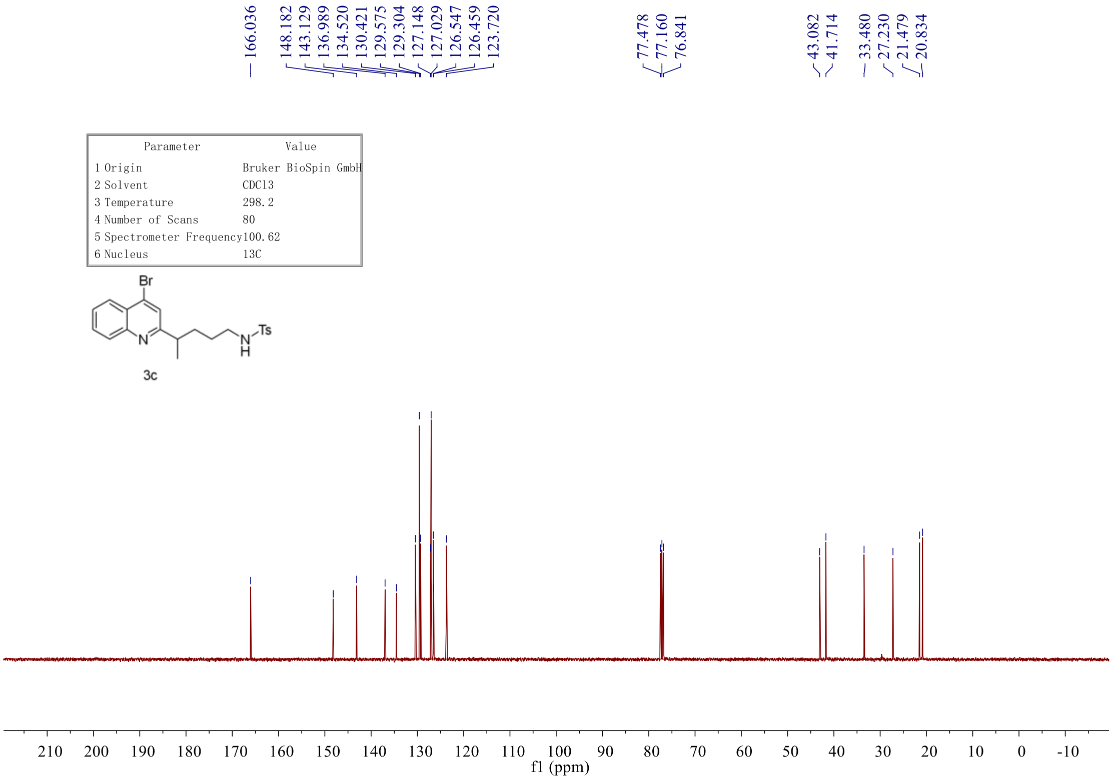
166.036
148.182 143.129 136.989 134.520 130.421 129.575 129.304 127.148 127.029 126.547 126.459 123.720
77.478 77.160 76.841
43.082 41.714 33.480 27.230 21.479 20.834
Parameter Value
1 Origin Bruker BioSpin GmbH
2 Solvent CDCl3
3 Temperature 298.2
4 Number of Scans 80
5 Spectrometer Frequency 100.62
6 Nucleus 13C
Br
N
N
H
Ts
3c
210 200 190 180 170 160 150 140 130 120 110 100 90 80 70 60 50 40 30 20 10 0 -10
f1 (ppm)

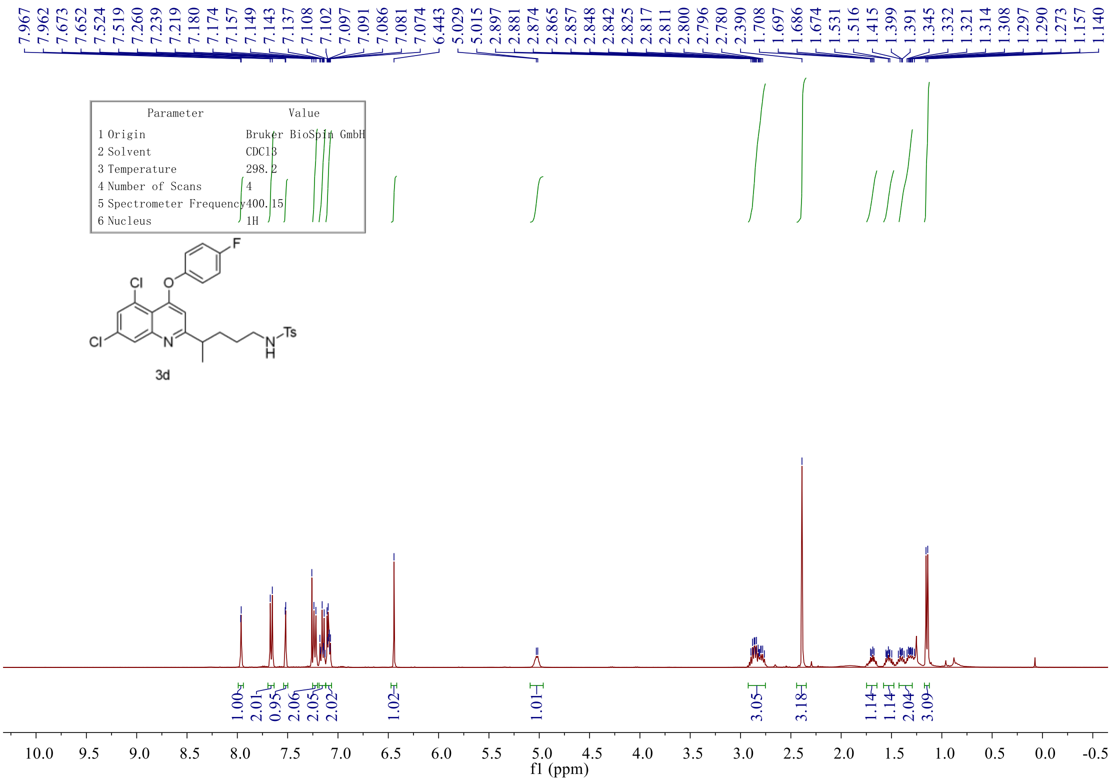
7.967 7.962 7.673 7.652 7.524 7.519 7.260 7.239 7.219 7.180 7.174 7.157 7.149 7.143 7.137 7.108 7.102 7.097 7.091 7.086 7.081 7.074 6.443 5.029 5.015 2.897 2.881 2.874 2.865 2.857 2.848 2.842 2.825 2.817 2.811 2.800 2.796 2.780 2.390 1.708 1.697 1.686 1.674 1.531 1.516 1.415 1.399 1.391 1.345 1.332 1.321 1.314 1.308 1.297 1.290 1.273 1.157 1.140
Parameter Value
1 Origin Bruker BioSpin GmbH
2 Solvent CDCl3
3 Temperature 298.2
4 Number of Scans 4
5 Spectrometer Frequency 400.15
6 Nucleus 1H
3d
1.00 2.01 0.95 2.06 2.05 2.02 1.02 1.01 3.05 3.18 1.14 1.14 2.04 3.09
f1 (ppm)

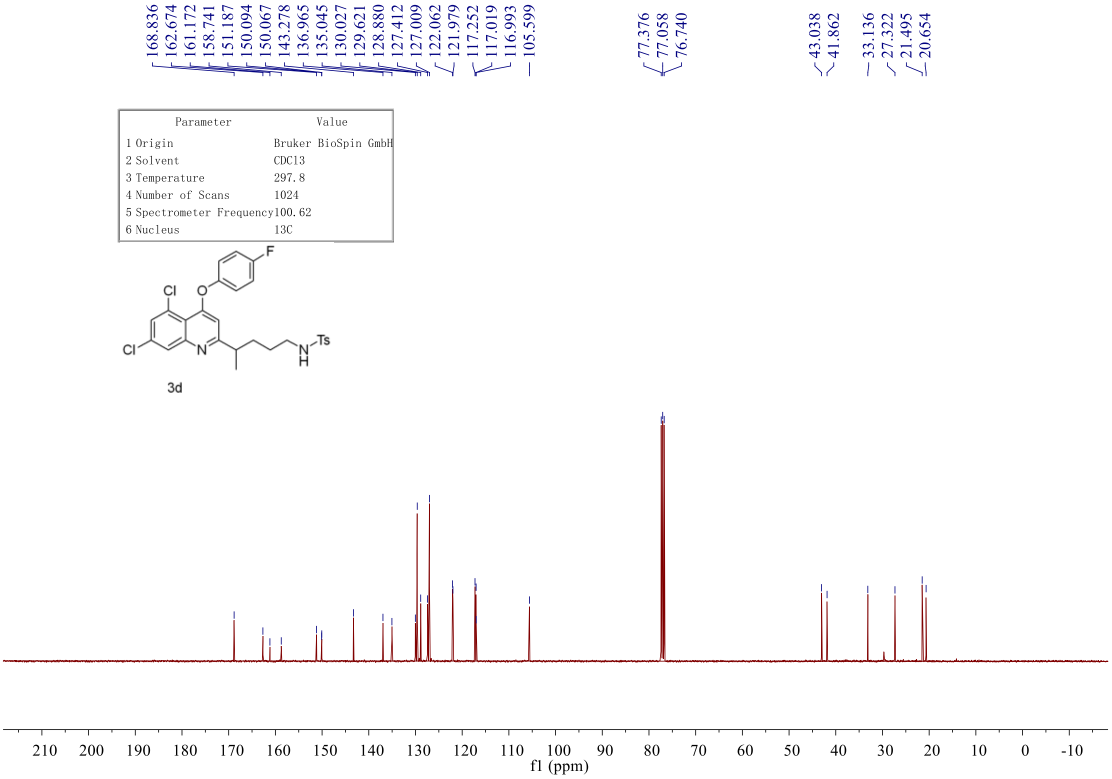
168.836 162.674 161.172 158.741 151.187 150.094 150.067 143.278 136.965 135.045 130.027 129.621 128.880 127.412 127.009 122.062 121.979 117.252 117.019 116.993 105.599
77.376 77.058 76.740
43.038 41.862 33.136 27.322 21.495 20.654
Parameter Value
1 Origin Bruker BioSpin GmbH
2 Solvent CDCl3
3 Temperature 297.8
4 Number of Scans 1024
5 Spectrometer Frequency 100.62
6 Nucleus 13C
3d
f1 (ppm)

Parameter	Value
1 Origin	Bruker BioSpin GmbH
2 Solvent	CDCl3
3 Temperature	298.2
4 Number of Scans	4
5 Spectrometer Frequency	376.52
6 Nucleus	19F

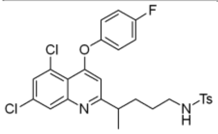

3d

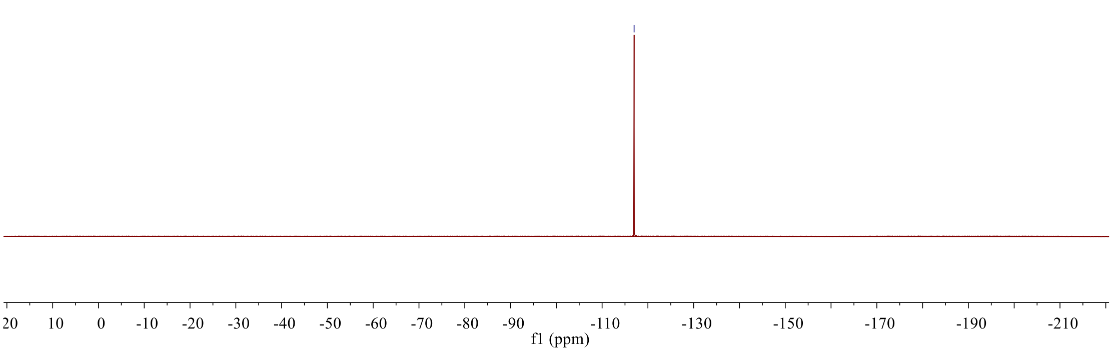

Parameter	Value
1 Origin	Bruker BioSpin GmbH
2 Solvent	CDCl3
3 Temperature	298.1
4 Number of Scans	2
5 Spectrometer Frequency	400.15
6 Nucleus	1H

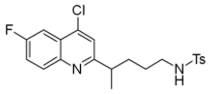

3e

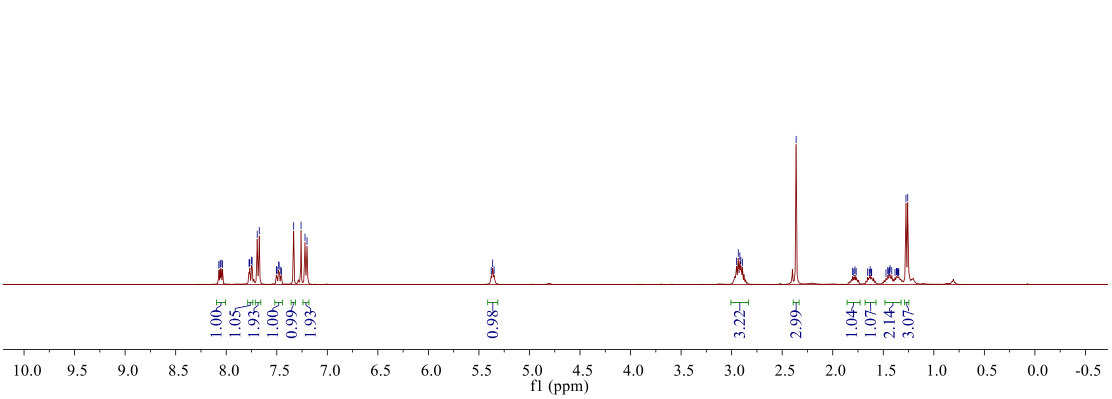

Parameter	Value
1 Origin	Bruker BioSpin GmbH
2 Solvent	CDCl3
3 Temperature	298.1
4 Number of Scans	478
5 Spectrometer Frequency	100.62
6 Nucleus	13C

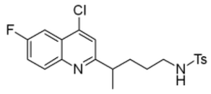

3e

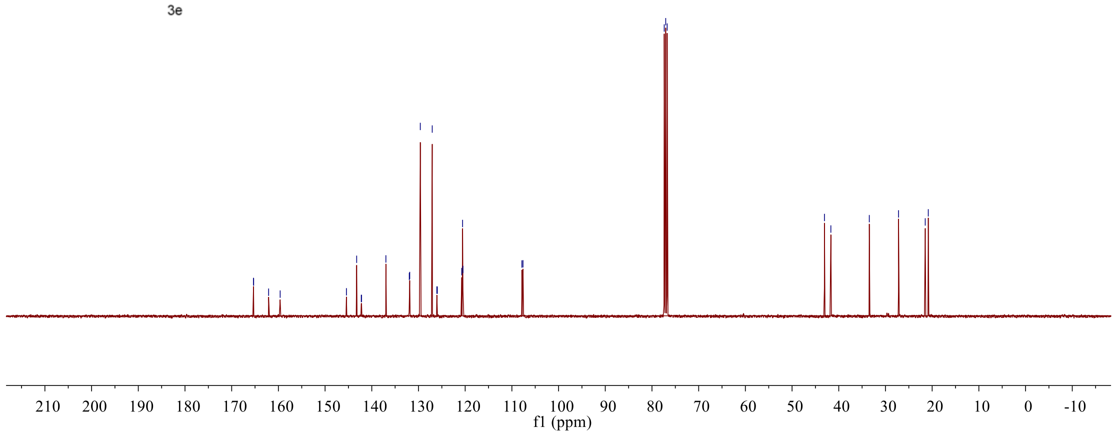

Parameter	Value
1 Origin	Bruker BioSpin GmbH
2 Solvent	CDCl3
3 Temperature	298.2
4 Number of Scans	4
5 Spectrometer Frequency	376.52
6 Nucleus	19F

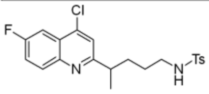

3e

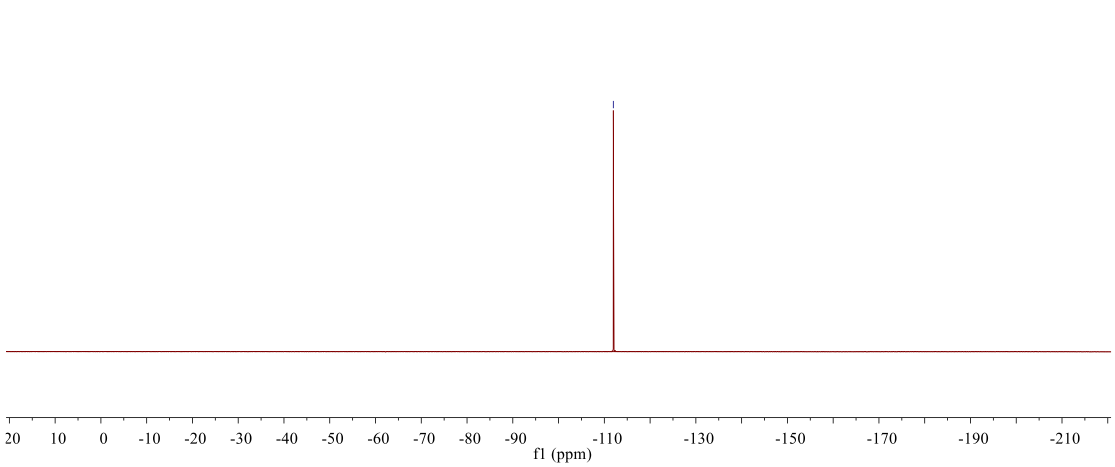

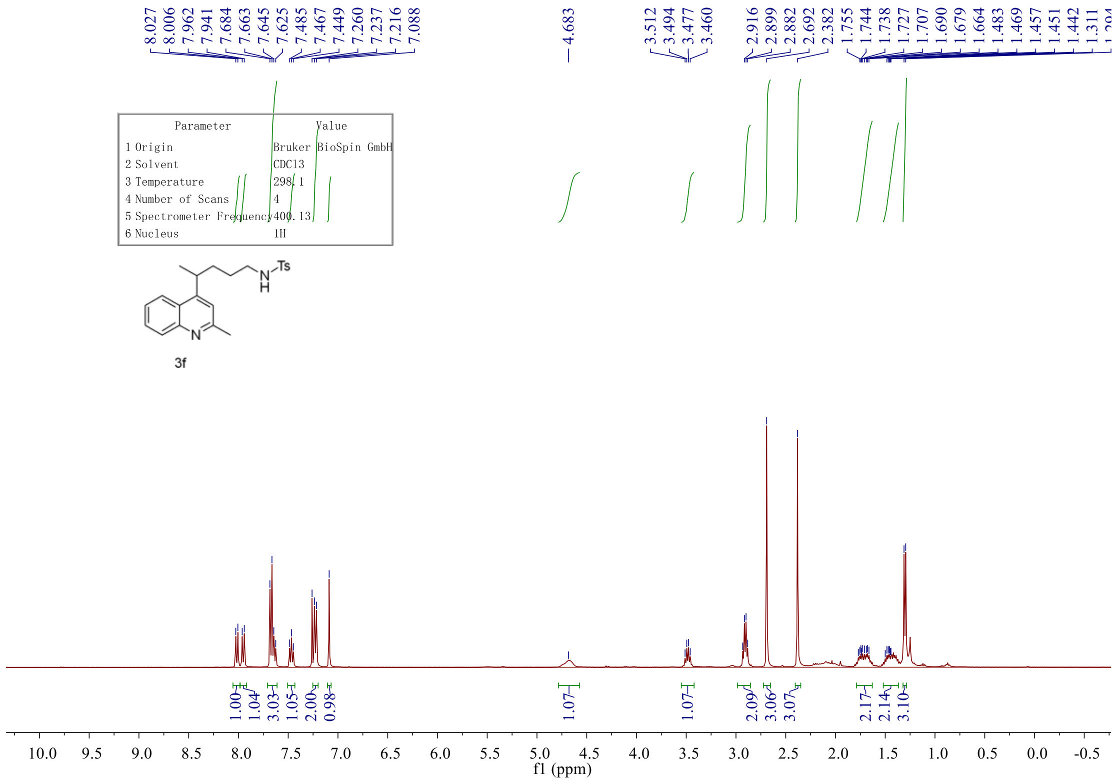
8.027 8.006 7.962 7.941 7.684 7.663 7.645 7.625 7.485 7.467 7.449 7.260 7.237 7.216 7.088
4.683
3.512 3.494 3.477 3.460
2.916 2.899 2.882 2.692 2.382 1.755 1.744 1.738 1.727 1.707 1.690 1.679 1.664 1.483 1.469 1.457 1.451 1.442 1.311
Parameter Value
1 Origin Bruker BioSpin GmbH
2 Solvent CDCl3
3 Temperature 298.1
4 Number of Scans 4
5 Spectrometer Frequency 400.13
6 Nucleus 1H
Ts
N
H
N
3f
1.00 1.04 3.03 1.05 2.00 0.98 1.07 1.07 2.09 3.06 3.07 2.17 2.14 3.10
10.0 9.5 9.0 8.5 8.0 7.5 7.0 6.5 6.0 5.5 5.0 4.5 4.0 3.5 3.0 2.5 2.0 1.5 1.0 0.5 0.0 -0.5
f1 (ppm)

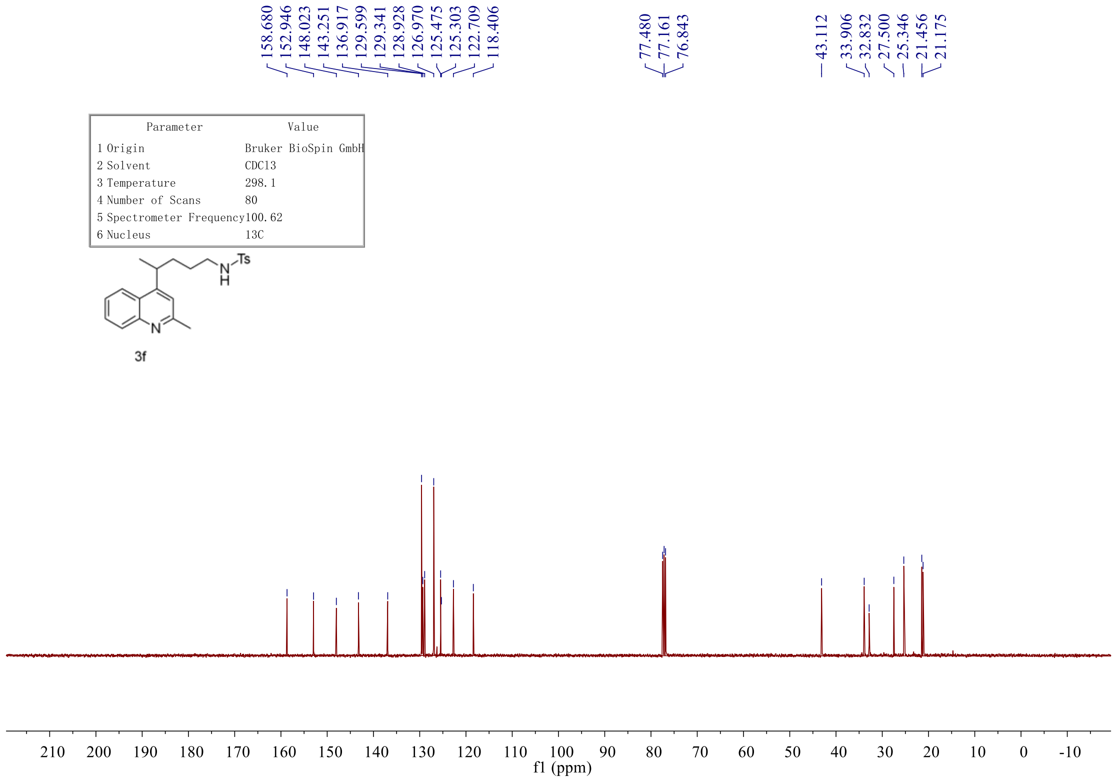
158.680 152.946 148.023 143.251 136.917 129.599 129.341 128.928 126.970 125.475 125.303 122.709 118.406
77.480 77.161 76.843
43.112 33.906 32.832 27.500 25.346 21.456 21.175
Parameter Value
1 Origin Bruker BioSpin GmbH
2 Solvent CDCl3
3 Temperature 298.1
4 Number of Scans 80
5 Spectrometer Frequency 100.62
6 Nucleus 13C
Ts
N
H
N
3f
210 200 190 180 170 160 150 140 130 120 110 100 90 80 70 60 50 40 30 20 10 0 -10
f1 (ppm)

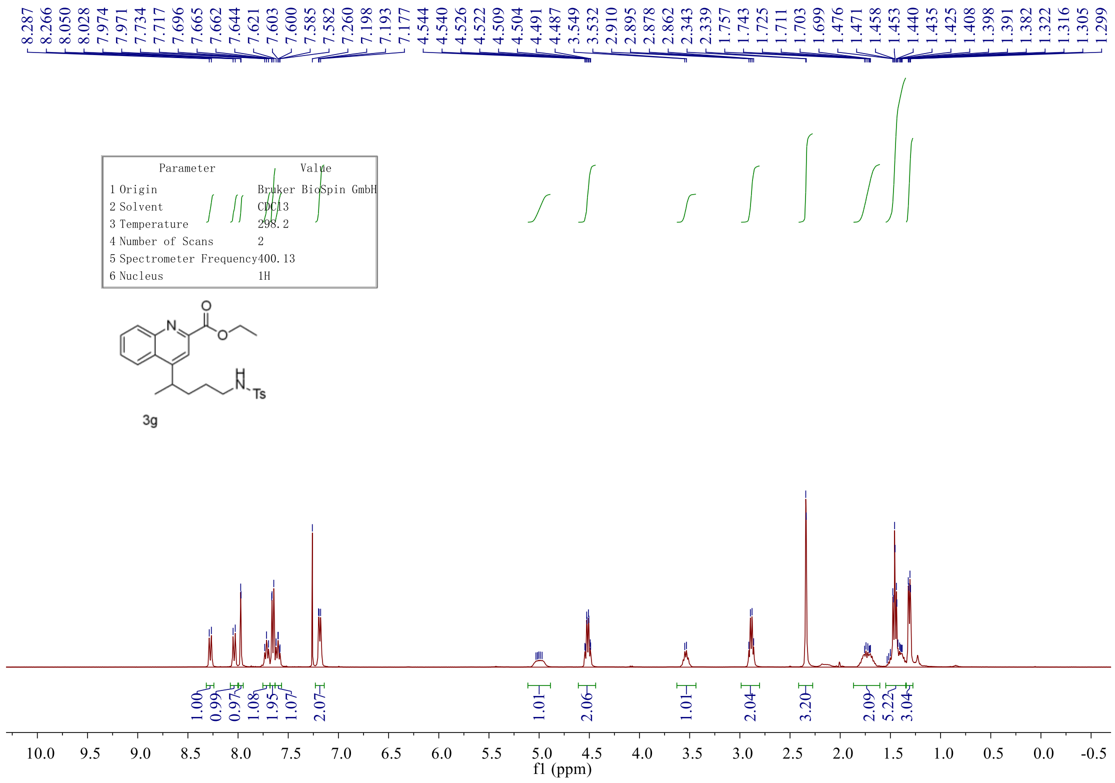
8.287 8.266 8.050 8.028 7.974 7.971 7.734 7.717 7.696 7.665 7.662 7.644 7.621 7.603 7.600 7.585 7.582 7.260 7.198 7.193 7.177 4.544 4.540 4.526 4.522 4.509 4.504 4.491 4.487 3.549 3.532 2.910 2.895 2.878 2.862 2.343 2.339 1.757 1.743 1.725 1.711 1.703 1.699 1.476 1.471 1.458 1.453 1.440 1.435 1.425 1.408 1.398 1.391 1.382 1.322 1.316 1.305 1.299
Parameter Value
1 Origin Bruker BioSpin GmbH
2 Solvent CDCl3
3 Temperature 298.2
4 Number of Scans 2
5 Spectrometer Frequency 400.13
6 Nucleus 1H
3g
1.00 0.99 0.97 1.08 1.95 1.07 2.07 1.01 2.06 1.01 2.04 3.20 2.09 5.22 3.04
f1 (ppm)

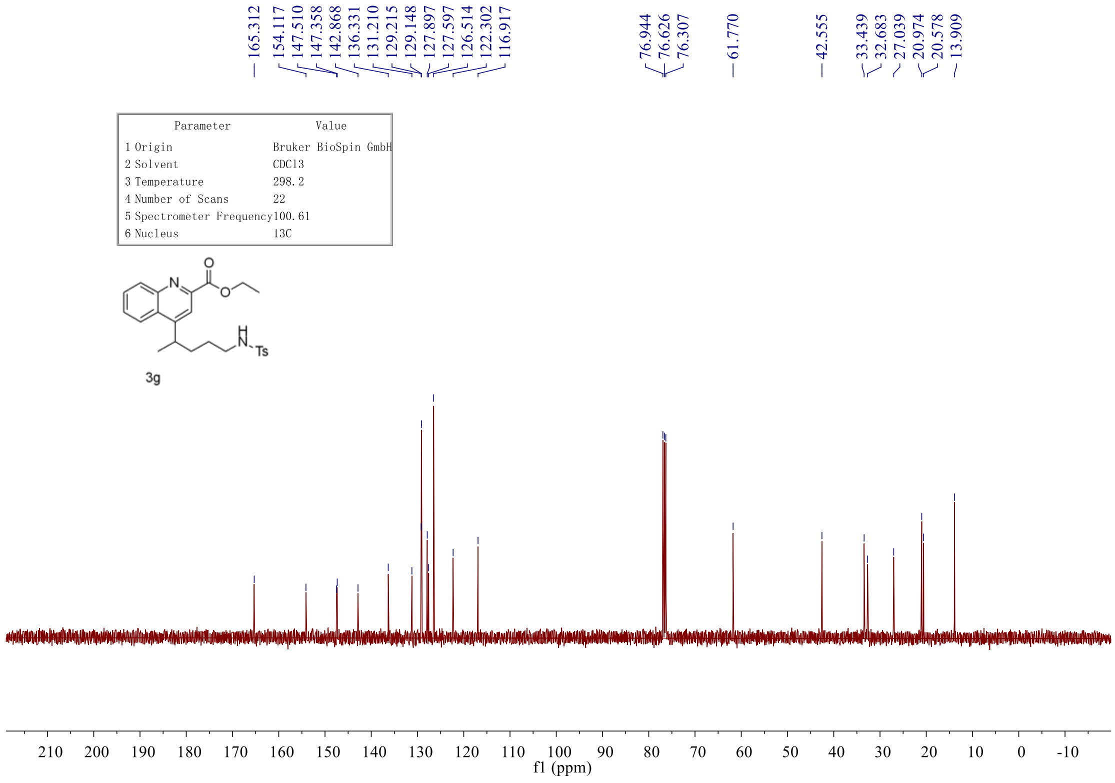
165.312 154.117 147.510 147.358 142.868 136.331 131.210 129.215 129.148 127.897 127.597 126.514 122.302 116.917 76.944 76.626 76.307 61.770 42.555 33.439 32.683 27.039 20.974 20.578 13.909
Parameter Value
1 Origin Bruker BioSpin GmbH
2 Solvent CDCl3
3 Temperature 298.2
4 Number of Scans 22
5 Spectrometer Frequency 100.61
6 Nucleus 13C
3g
f1 (ppm)

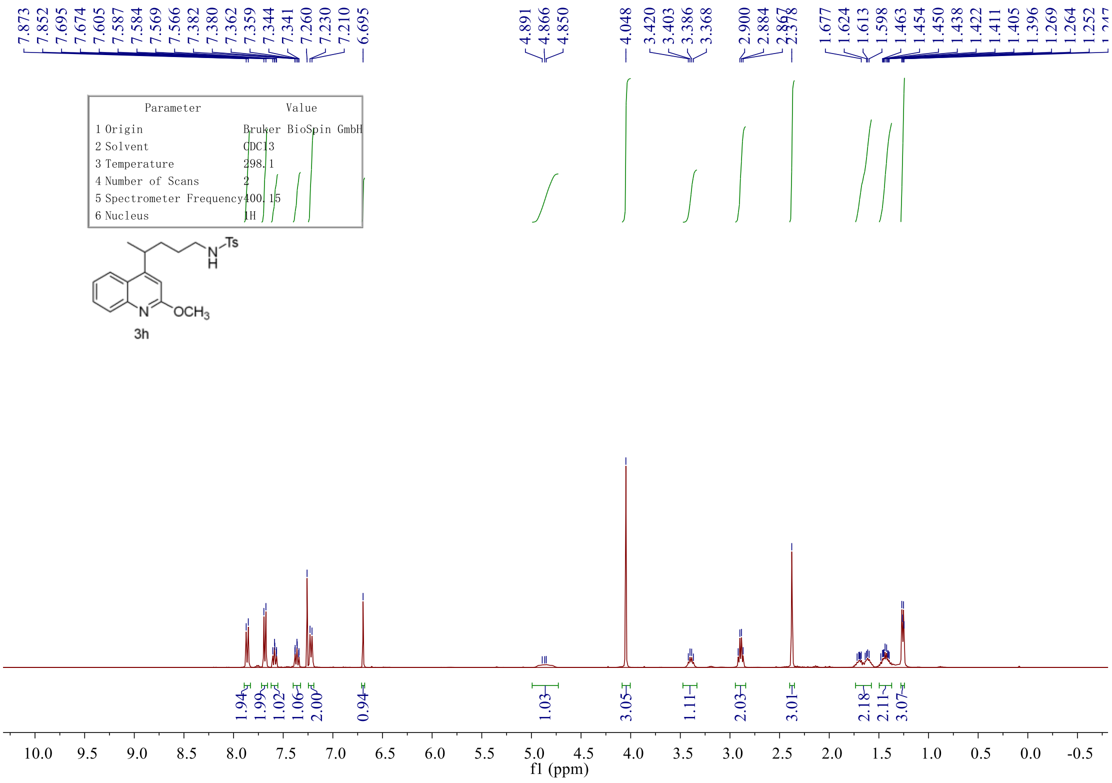
7.873 7.852 7.695 7.674 7.605 7.587 7.584 7.569 7.566 7.382 7.380 7.362 7.359 7.344 7.341 7.260 7.230 7.210 6.695
4.891 4.866 4.850
4.048 3.420 3.403 3.386 3.368
2.900 2.884 2.867 2.378
1.677 1.624 1.613 1.598 1.463 1.454 1.450 1.438 1.422 1.411 1.405 1.396 1.269 1.264 1.252
Parameter Value
1 Origin Bruker BioSpin GmbH
2 Solvent CDCl3
3 Temperature 298.1
4 Number of Scans 2
5 Spectrometer Frequency 400.15
6 Nucleus 1H
Ts
N
H
N
OCH3
3h
1.94 1.99 1.02 1.06 2.00 0.94 1.03 3.05 1.11 2.03 3.01 2.18 2.11 3.07
f1 (ppm)

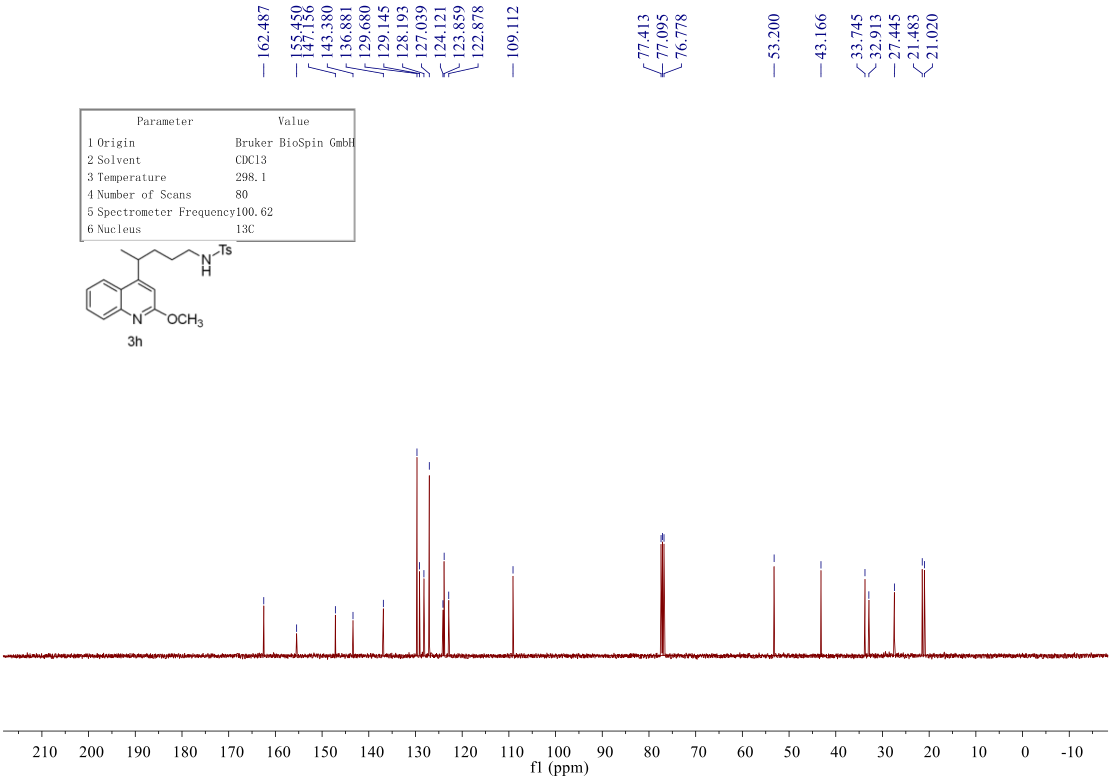
162.487 155.450 147.156 143.380 136.881 129.680 129.145 128.193 127.039 124.121 123.859 122.878 109.112
77.413 77.095 76.778
53.200 43.166 33.745 32.913 27.445 21.483 21.020
Parameter Value
1 Origin Bruker BioSpin GmbH
2 Solvent CDCl3
3 Temperature 298.1
4 Number of Scans 80
5 Spectrometer Frequency 100.62
6 Nucleus 13C
Ts
N
H
N
OCH3
3h
f1 (ppm)

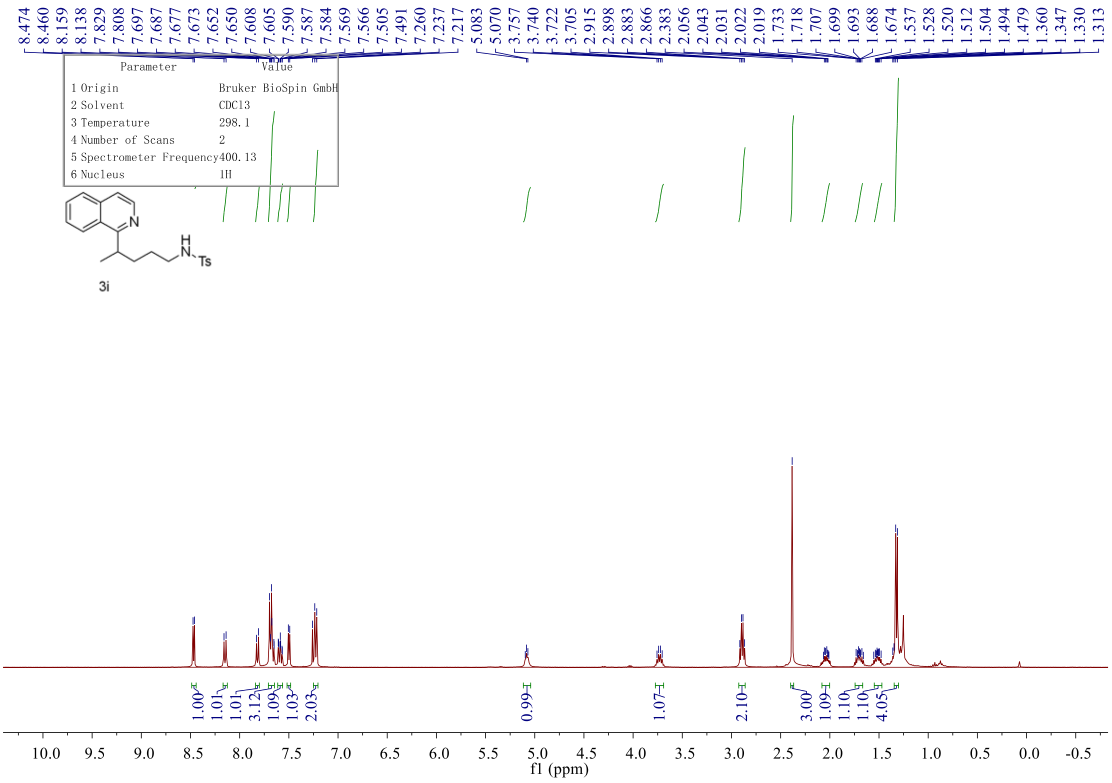
8.474
8.460
8.159
8.138
7.829
7.808
7.697
7.687
7.677
7.673
7.652
7.650
7.608
7.605
7.590
7.587
7.584
7.569
7.566
7.505
7.491
7.260
7.237
7.217
5.083
5.070
3.757
3.740
3.722
3.705
2.915
2.898
2.883
2.866
2.383
2.056
2.043
2.031
2.022
2.019
1.733
1.718
1.707
1.699
1.693
1.688
1.674
1.537
1.528
1.520
1.512
1.504
1.494
1.479
1.360
1.347
1.330
1.313
Parameter Value
1 Origin Bruker BioSpin GmbH
2 Solvent CDCl3
3 Temperature 298.1
4 Number of Scans 2
5 Spectrometer Frequency 400.13
6 Nucleus 1H
N
H
N
Ts
3i
1.00
1.01
1.01
3.12
1.09
1.03
2.03
0.99
1.07
2.10
3.00
1.09
1.10
1.10
4.05
10.0 9.5 9.0 8.5 8.0 7.5 7.0 6.5 6.0 5.5 5.0 4.5 4.0 3.5 3.0 2.5 2.0 1.5 1.0 0.5 0.0 -0.5
f1 (ppm)

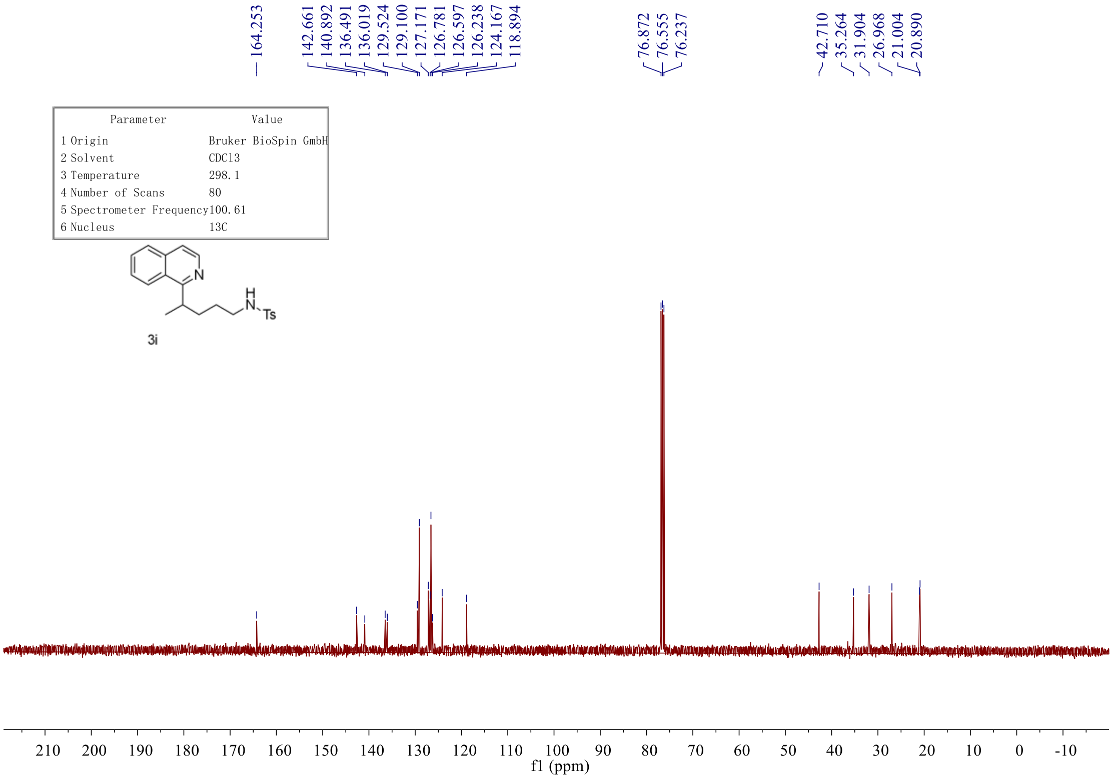
164.253
142.661
140.892
136.491
136.019
129.524
129.100
127.171
126.781
126.597
126.238
124.167
118.894
76.872
76.555
76.237
42.710
35.264
31.904
26.968
21.004
20.890
Parameter Value
1 Origin Bruker BioSpin GmbH
2 Solvent CDCl3
3 Temperature 298.1
4 Number of Scans 80
5 Spectrometer Frequency 100.61
6 Nucleus 13C
N
H
N
Ts
3i
210 200 190 180 170 160 150 140 130 120 110 100 90 80 70 60 50 40 30 20 10 0 -10
f1 (ppm)

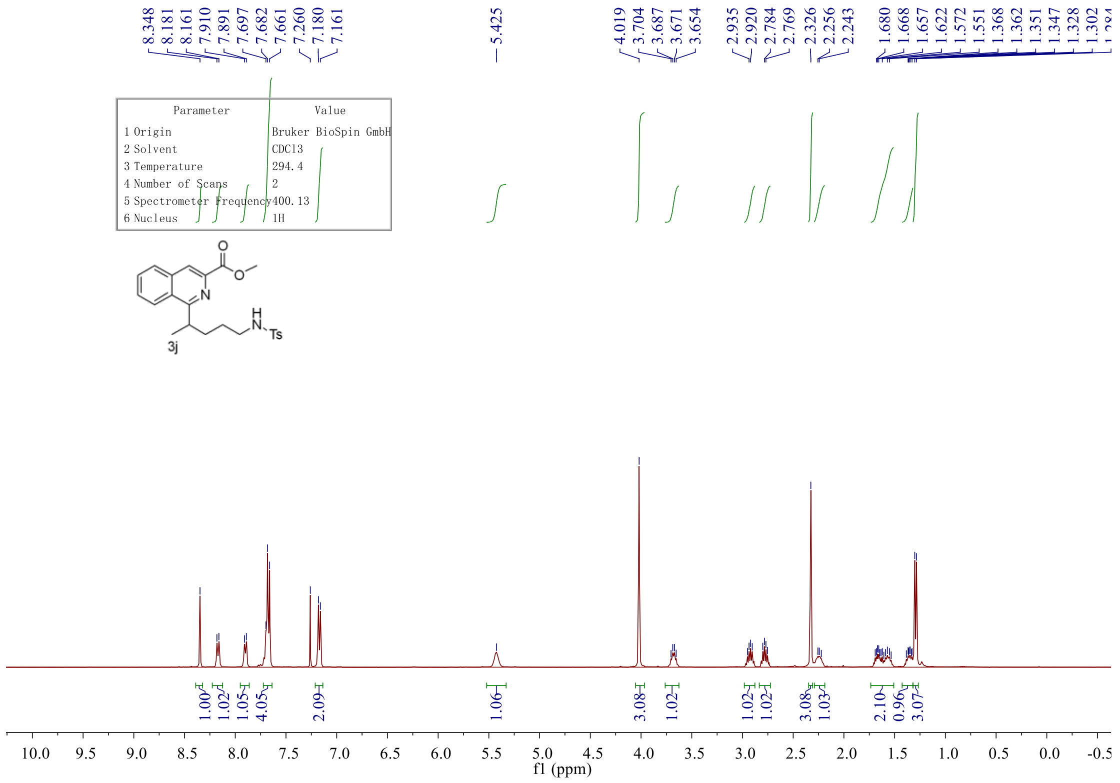
8.348
8.181
8.161
7.910
7.891
7.697
7.682
7.661
7.260
7.180
7.161
5.425
4.019
3.704
3.687
3.671
3.654
2.935
2.920
2.784
2.769
2.326
2.256
2.243
1.680
1.668
1.657
1.622
1.572
1.551
1.368
1.362
1.351
1.347
1.328
1.302
Parameter Value
1 Origin Bruker BioSpin GmbH
2 Solvent CDCl3
3 Temperature 294.4
4 Number of Scans 2
5 Spectrometer Frequency 400.13
6 Nucleus 1H
3j
Ts
1.00
1.02
1.05
4.05
2.09
1.06
3.08
1.02
1.02
1.02
3.08
1.03
2.10
0.96
3.07
f1 (ppm)

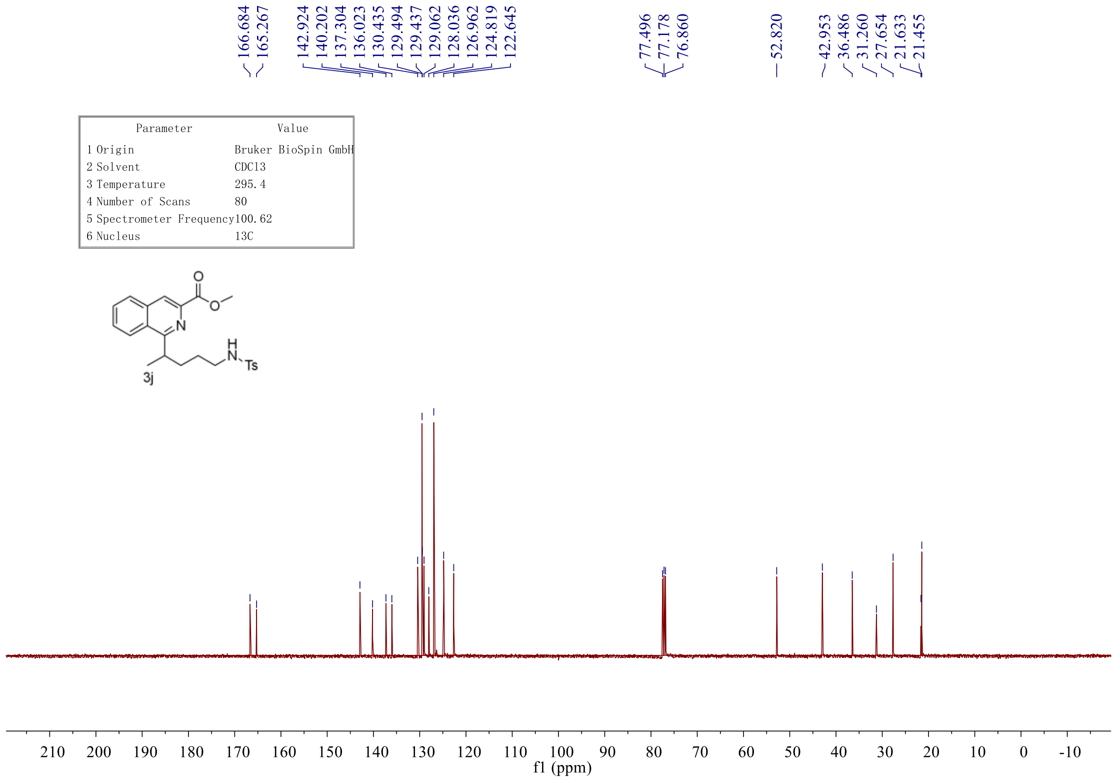
166.684
165.267
142.924
140.202
137.304
136.023
130.435
129.494
129.437
129.062
128.036
126.962
124.819
122.645
77.496
77.178
76.860
52.820
42.953
36.486
31.260
27.654
21.633
21.455
Parameter Value
1 Origin Bruker BioSpin GmbH
2 Solvent CDCl3
3 Temperature 295.4
4 Number of Scans 80
5 Spectrometer Frequency 100.62
6 Nucleus 13C
3j
Ts
f1 (ppm)

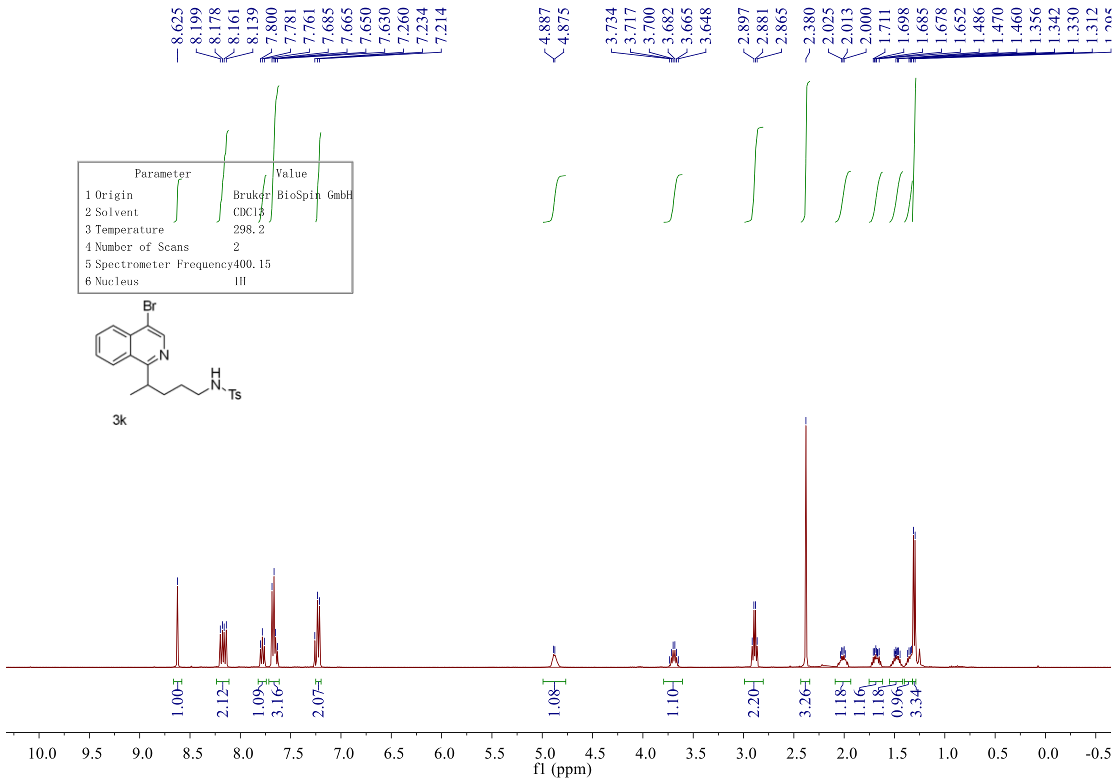
8.625 8.199 8.178 8.161 8.139 7.800 7.781 7.761 7.685 7.665 7.650 7.630 7.260 7.234 7.214
4.887 4.875
3.734 3.717 3.700 3.682 3.665 3.648
2.897 2.881 2.865 2.380 2.025 2.013 2.000 1.711 1.698 1.685 1.678 1.652 1.486 1.470 1.460 1.356 1.342 1.330 1.312
Parameter Value
1 Origin Bruker BioSpin GmbH
2 Solvent CDCl3
3 Temperature 298.2
4 Number of Scans 2
5 Spectrometer Frequency 400.15
6 Nucleus 1H
Br
N
H
N
Ts
3k
1.00 2.12 1.09 3.16 2.07 1.08 1.10 2.20 3.26 1.18 1.16 1.18 0.96 3.34
10.0 9.5 9.0 8.5 8.0 7.5 7.0 6.5 6.0 5.5 5.0 4.5 4.0 3.5 3.0 2.5 2.0 1.5 1.0 0.5 0.0 -0.5
f1 (ppm)

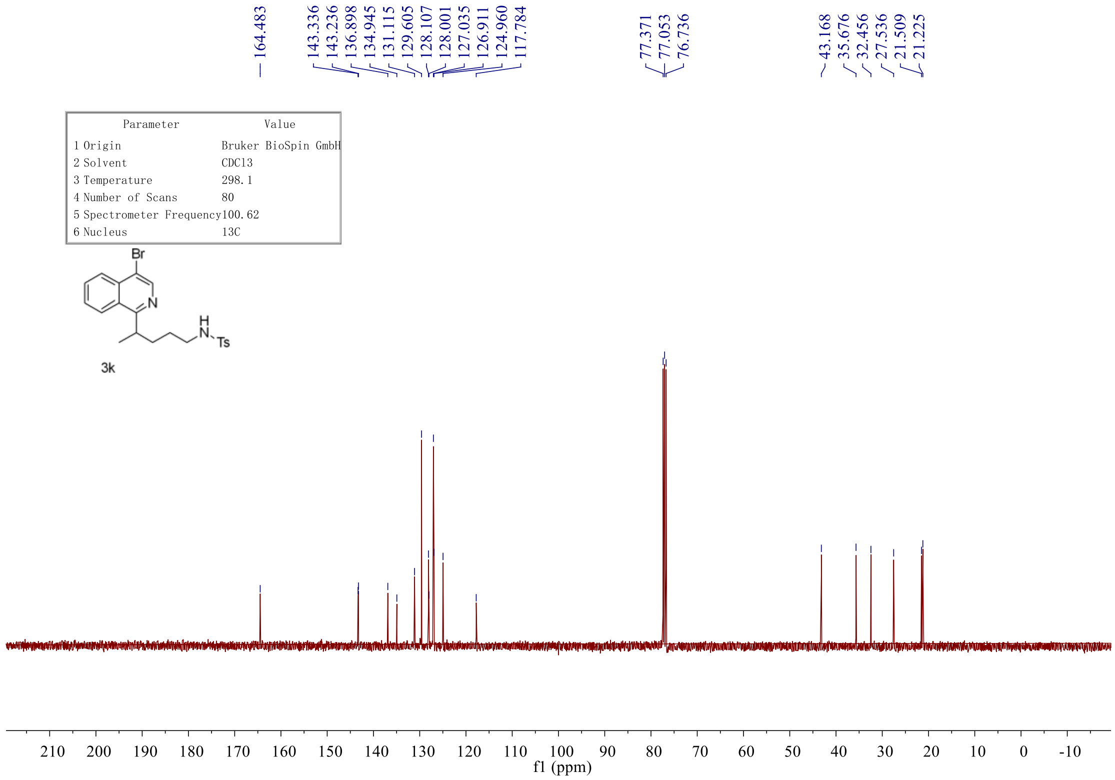
164.483
143.336 143.236 136.898 134.945 131.115 129.605 128.107 128.001 127.035 126.911 124.960 117.784
77.371 77.053 76.736
43.168 35.676 32.456 27.536 21.509 21.225
Parameter Value
1 Origin Bruker BioSpin GmbH
2 Solvent CDCl3
3 Temperature 298.1
4 Number of Scans 80
5 Spectrometer Frequency 100.62
6 Nucleus 13C
Br
N
H
N
Ts
3k
210 200 190 180 170 160 150 140 130 120 110 100 90 80 70 60 50 40 30 20 10 0 -10
f1 (ppm)

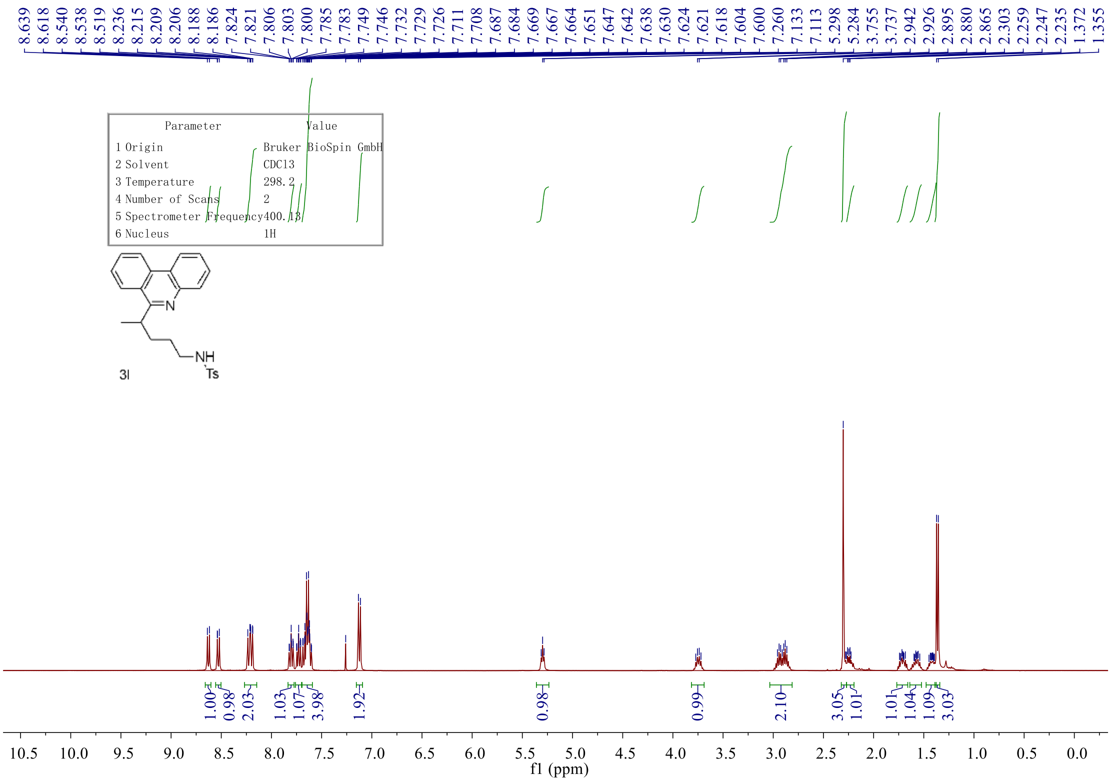
8.639 8.618 8.540 8.538 8.519 8.236 8.215 8.209 8.206 8.188 8.186 7.824 7.821 7.806 7.803 7.800 7.785 7.783 7.749 7.746 7.732 7.729 7.726 7.711 7.708 7.687 7.684 7.669 7.667 7.664 7.651 7.647 7.642 7.638 7.630 7.624 7.621 7.618 7.604 7.600 7.260 7.133 7.113 5.298 5.284 3.755 3.737 2.942 2.926 2.895 2.880 2.865 2.303 2.259 2.247 2.235 1.372 1.355
Parameter Value
1 Origin Bruker BioSpin GmbH
2 Solvent CDCl3
3 Temperature 298.2
4 Number of Scans 2
5 Spectrometer Frequency 400.13
6 Nucleus 1H
N
NH
Ts
3l
1.00 0.98 2.03 1.03 1.07 3.98 1.92 0.98 0.99 2.10 3.05 1.01 1.01 1.04 1.09 3.03
10.5 10.0 9.5 9.0 8.5 8.0 7.5 7.0 6.5 6.0 5.5 5.0 4.5 4.0 3.5 3.0 2.5 2.0 1.5 1.0 0.5 0.0
f1 (ppm)

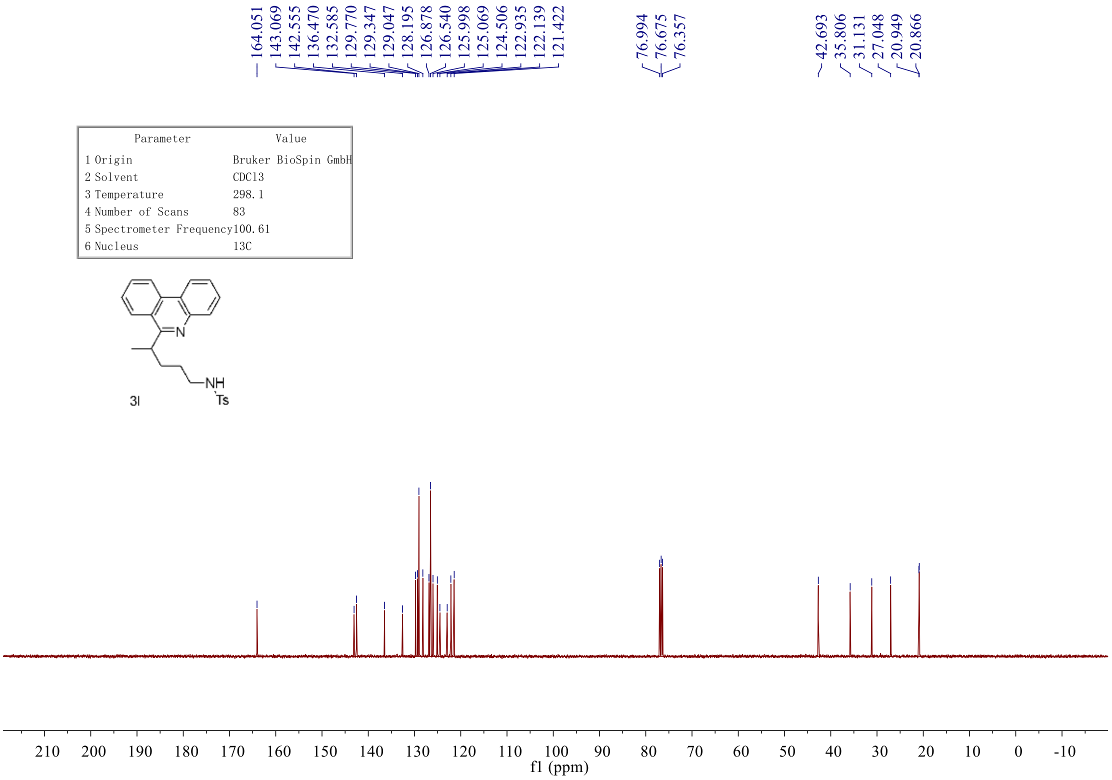
164.051 143.069 142.555 136.470 132.585 129.770 129.347 129.047 128.195 126.878 126.540 125.998 125.069 124.506 122.935 122.139 121.422
76.994 76.675 76.357
42.693 35.806 31.131 27.048 20.949 20.866
Parameter Value
1 Origin Bruker BioSpin GmbH
2 Solvent CDCl3
3 Temperature 298.1
4 Number of Scans 83
5 Spectrometer Frequency 100.61
6 Nucleus 13C
N
NH
Ts
3l
210 200 190 180 170 160 150 140 130 120 110 100 90 80 70 60 50 40 30 20 10 0 -10
f1 (ppm)

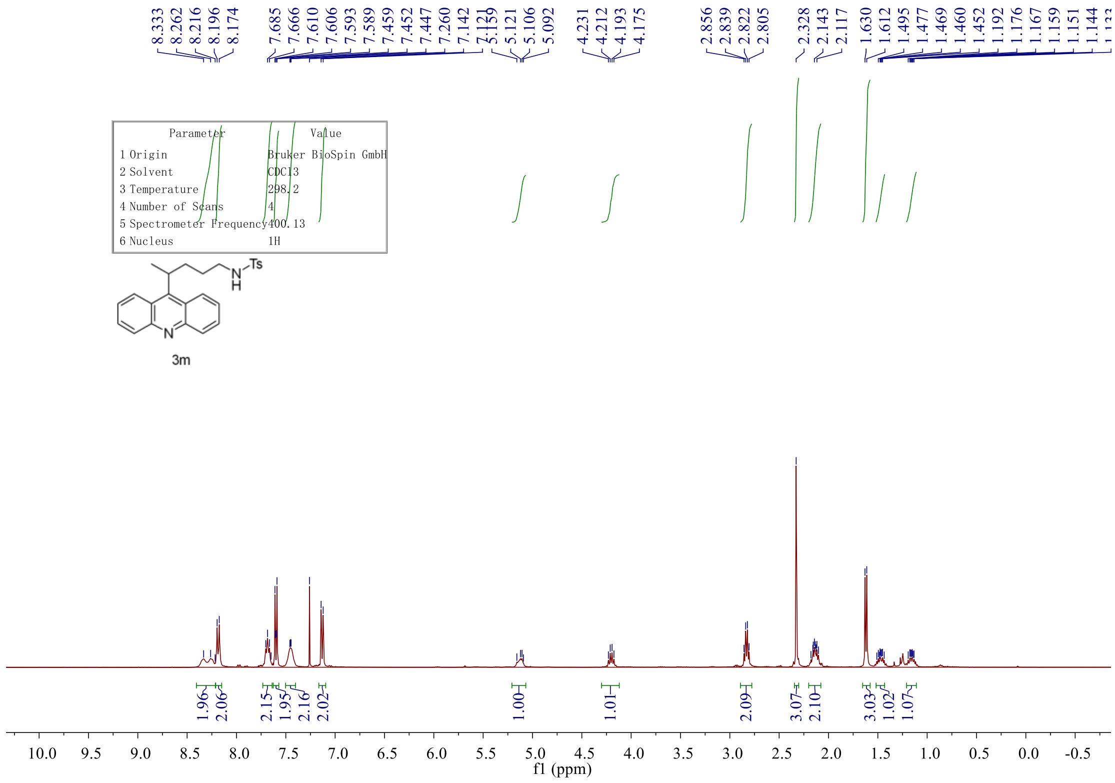
8.333 8.262 8.216 8.196 8.174 7.685 7.666 7.610 7.606 7.593 7.589 7.459 7.452 7.447 7.260 7.142 7.121 5.159 5.121 5.106 5.092 4.231 4.212 4.193 4.175 2.856 2.839 2.822 2.805 2.328 2.143 2.117 1.630 1.612 1.495 1.477 1.469 1.460 1.452 1.192 1.176 1.167 1.159 1.151 1.144
Parameter Value
1 Origin Bruker BioSpin GmbH
2 Solvent CDCl3
3 Temperature 298.2
4 Number of Scans 4
5 Spectrometer Frequency 400.13
6 Nucleus 1H
Ts
N
H
N
3m
1.96 2.06 2.15 1.95 2.16 2.02 1.00 1.01 2.09 3.07 2.10 3.03 1.02 1.07
10.0 9.5 9.0 8.5 8.0 7.5 7.0 6.5 6.0 5.5 5.0 4.5 4.0 3.5 3.0 2.5 2.0 1.5 1.0 0.5 0.0 -0.5
f1 (ppm)

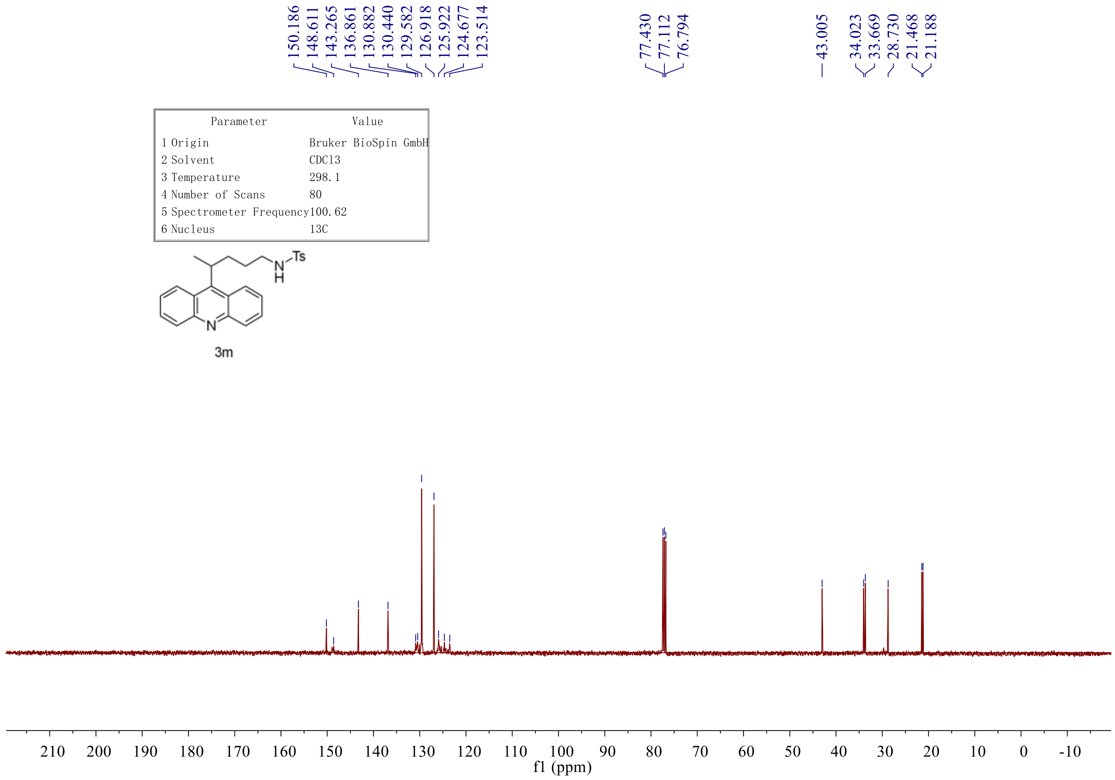
150.186 148.611 143.265 136.861 130.882 130.440 129.582 126.918 125.922 124.677 123.514
77.430 77.112 76.794
43.005
34.023 33.669 28.730 21.468 21.188
Parameter Value
1 Origin Bruker BioSpin GmbH
2 Solvent CDCl3
3 Temperature 298.1
4 Number of Scans 80
5 Spectrometer Frequency 100.62
6 Nucleus 13C
Ts
N
H
N
3m
210 200 190 180 170 160 150 140 130 120 110 100 90 80 70 60 50 40 30 20 10 0 -10
f1 (ppm)

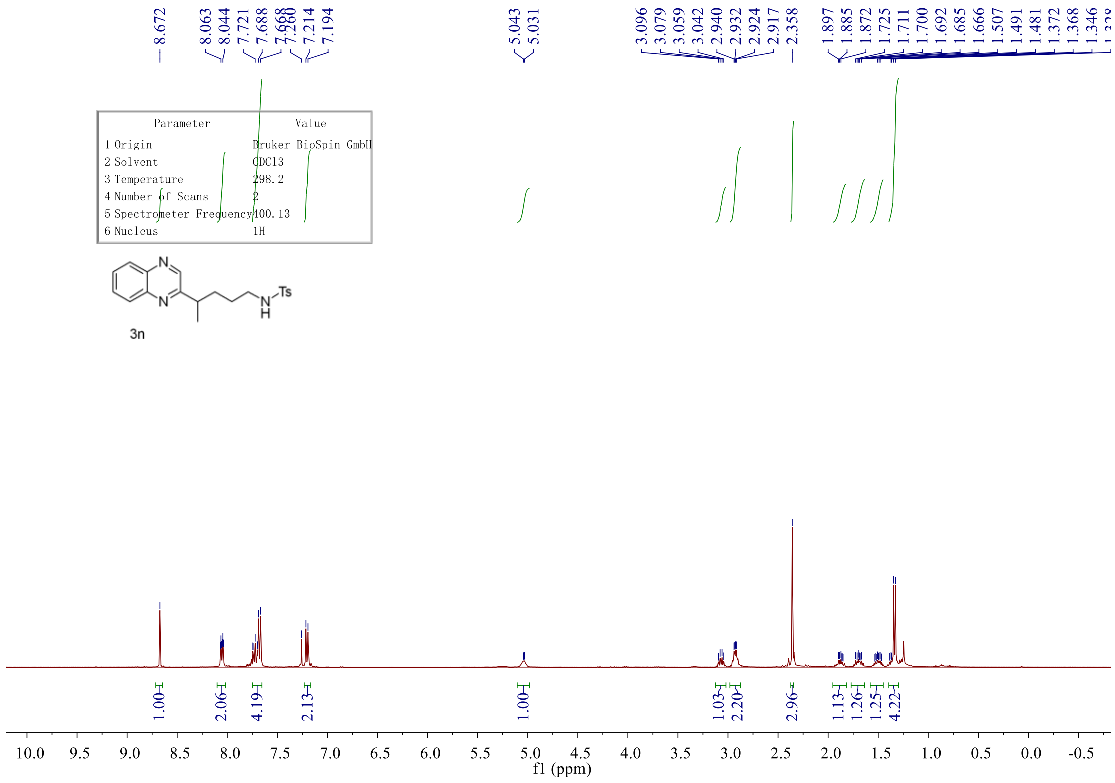
8.672
8.063
8.044
7.721
7.688
7.668
7.260
7.214
7.194
5.043
5.031
3.096
3.079
3.059
3.042
2.940
2.932
2.924
2.917
2.358
1.897
1.885
1.872
1.725
1.711
1.700
1.692
1.685
1.666
1.507
1.491
1.481
1.372
1.368
1.346
Parameter | Value
1 Origin | Bruker BioSpin GmbH
2 Solvent | CDCl3
3 Temperature | 298.2
4 Number of Scans | 2
5 Spectrometer Frequency | 400.13
6 Nucleus | 1H
N
N
N
H
Ts
3n
1.00
2.06
4.19
2.13
1.00
1.03
2.20
2.96
1.13
1.26
1.25
4.22
10.0 9.5 9.0 8.5 8.0 7.5 7.0 6.5 6.0 5.5 5.0 4.5 4.0 3.5 3.0 2.5 2.0 1.5 1.0 0.5 0.0 -0.5
f1 (ppm)

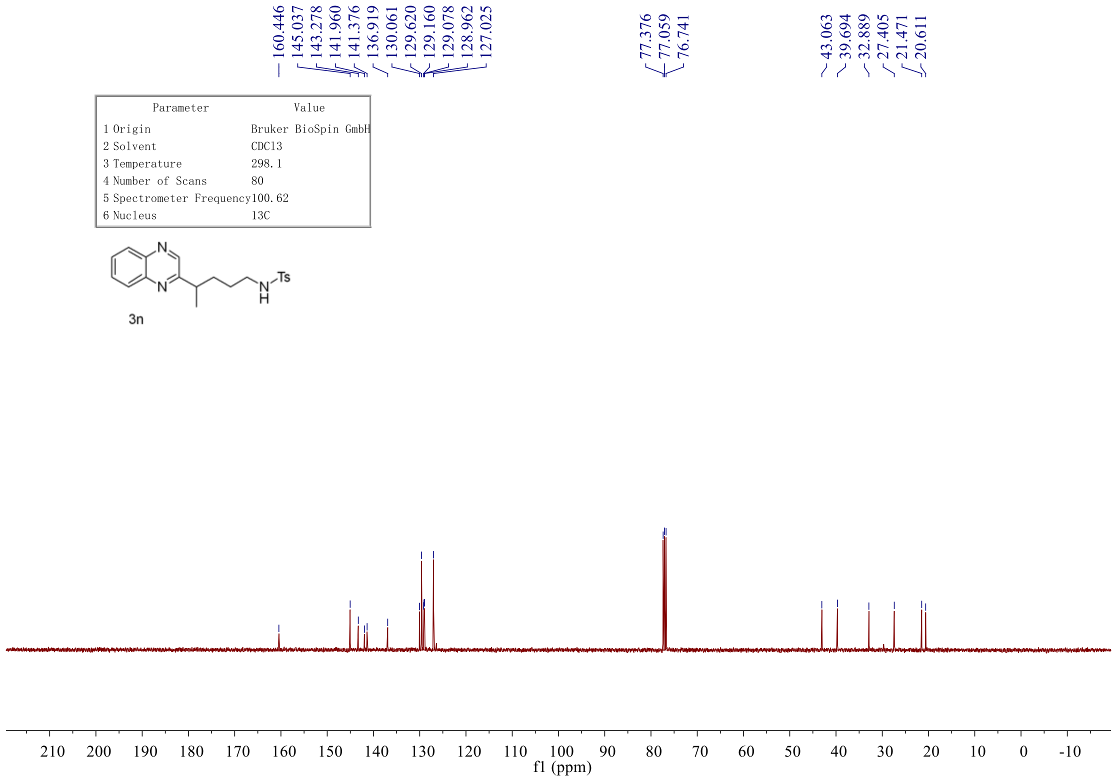
160.446
145.037
143.278
141.960
141.376
136.919
130.061
129.620
129.160
129.078
128.962
127.025
77.376
77.059
76.741
43.063
39.694
32.889
27.405
21.471
20.611
Parameter | Value
1 Origin | Bruker BioSpin GmbH
2 Solvent | CDCl3
3 Temperature | 298.1
4 Number of Scans | 80
5 Spectrometer Frequency | 100.62
6 Nucleus | 13C
N
N
N
H
Ts
3n
210 200 190 180 170 160 150 140 130 120 110 100 90 80 70 60 50 40 30 20 10 0 -10
f1 (ppm)

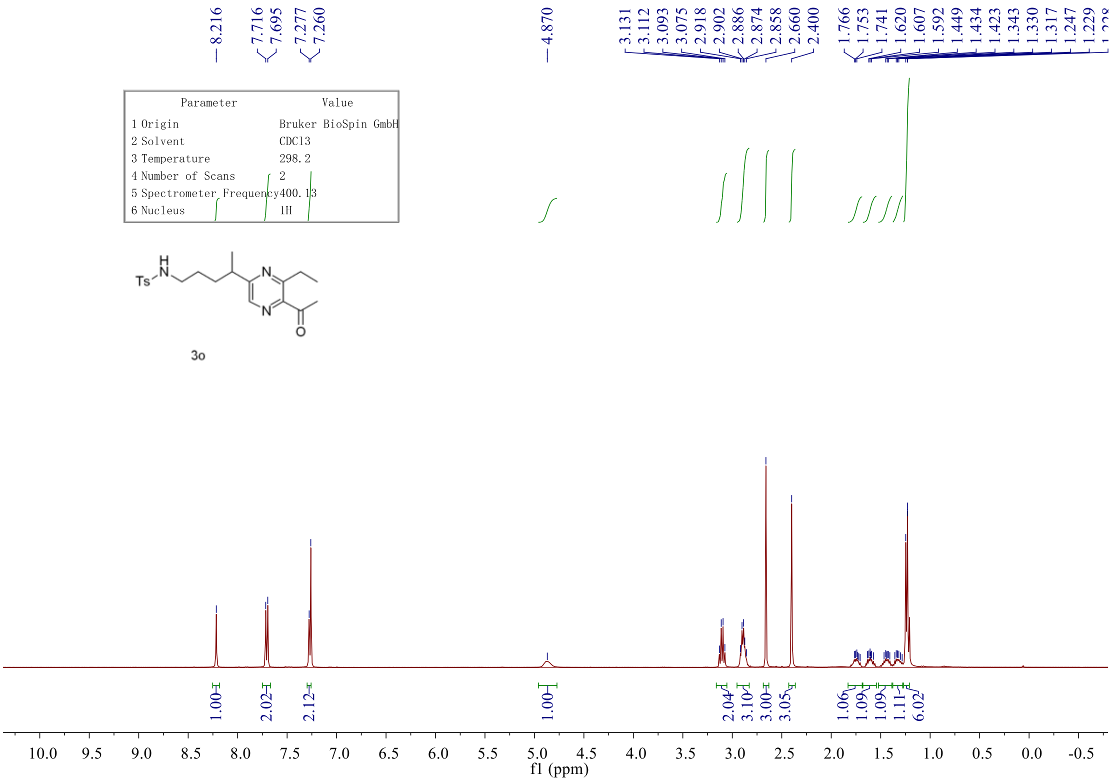
8.216
7.716
7.695
7.277
7.260
4.870
3.131
3.112
3.093
3.075
2.918
2.902
2.886
2.874
2.858
2.660
2.400
1.766
1.753
1.741
1.620
1.607
1.592
1.449
1.434
1.423
1.343
1.330
1.317
1.247
1.229
Parameter Value
1 Origin Bruker BioSpin GmbH
2 Solvent CDCl3
3 Temperature 298.2
4 Number of Scans 2
5 Spectrometer Frequency 400.13
6 Nucleus 1H
Ts
H
N
N
N
O
3o
1.00
2.02
2.12
1.00
2.04
3.10
3.00
3.05
1.06
1.09
1.09
1.11
6.02
10.0 9.5 9.0 8.5 8.0 7.5 7.0 6.5 6.0 5.5 5.0 4.5 4.0 3.5 3.0 2.5 2.0 1.5 1.0 0.5 0.0 -0.5
f1 (ppm)

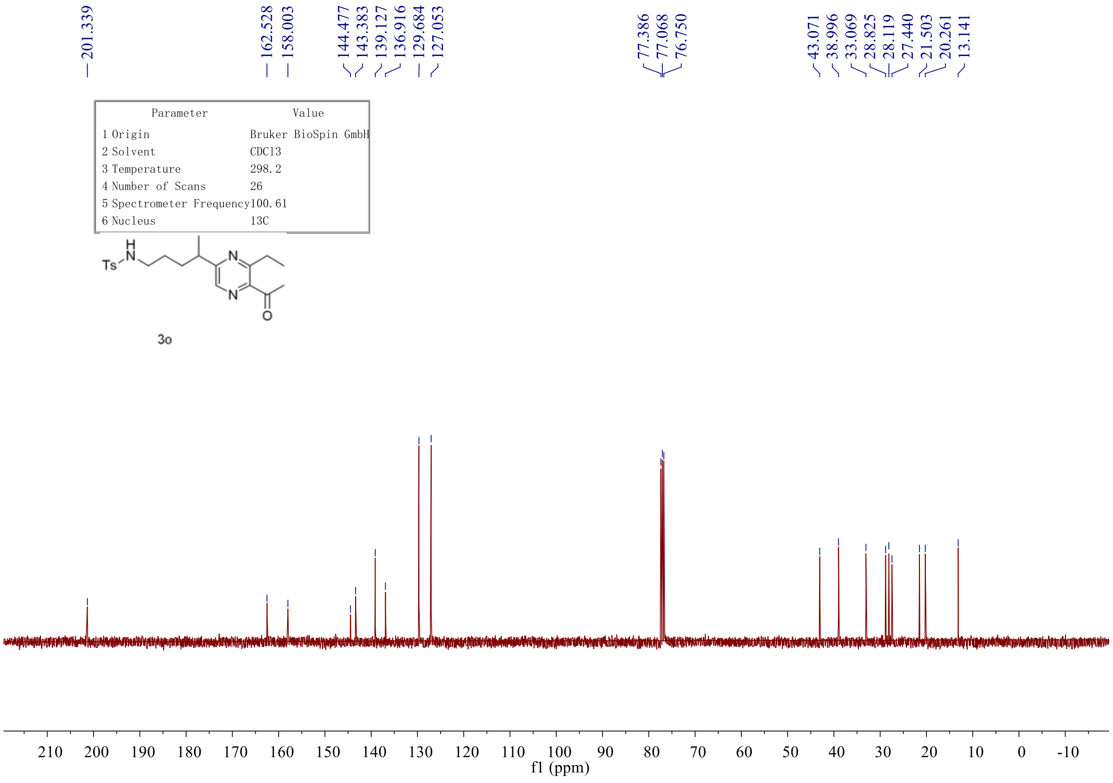
201.339
162.528
158.003
144.477
143.383
139.127
136.916
129.684
127.053
77.386
77.068
76.750
43.071
38.996
33.069
28.825
28.119
27.440
21.503
20.261
13.141
Parameter Value
1 Origin Bruker BioSpin GmbH
2 Solvent CDCl3
3 Temperature 298.2
4 Number of Scans 26
5 Spectrometer Frequency 100.61
6 Nucleus 13C
Ts
H
N
N
N
O
3o
210 200 190 180 170 160 150 140 130 120 110 100 90 80 70 60 50 40 30 20 10 0 -10
f1 (ppm)

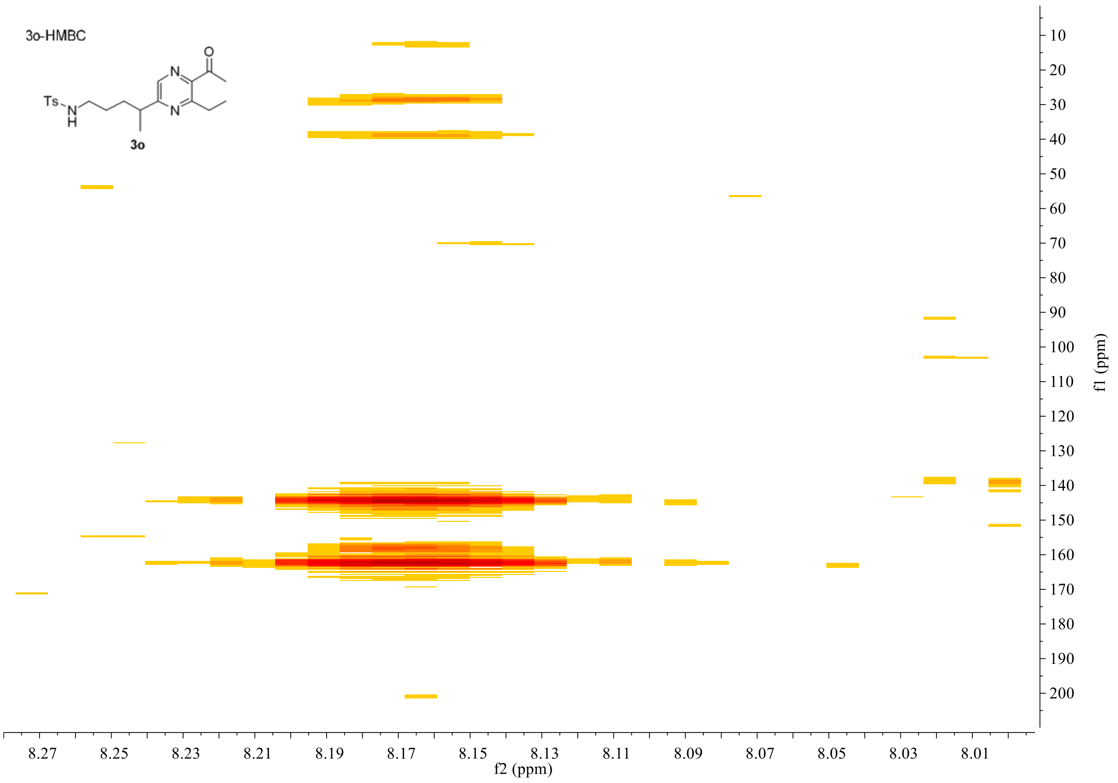
3o-HMBC
3o
f2 (ppm)
f1 (ppm)

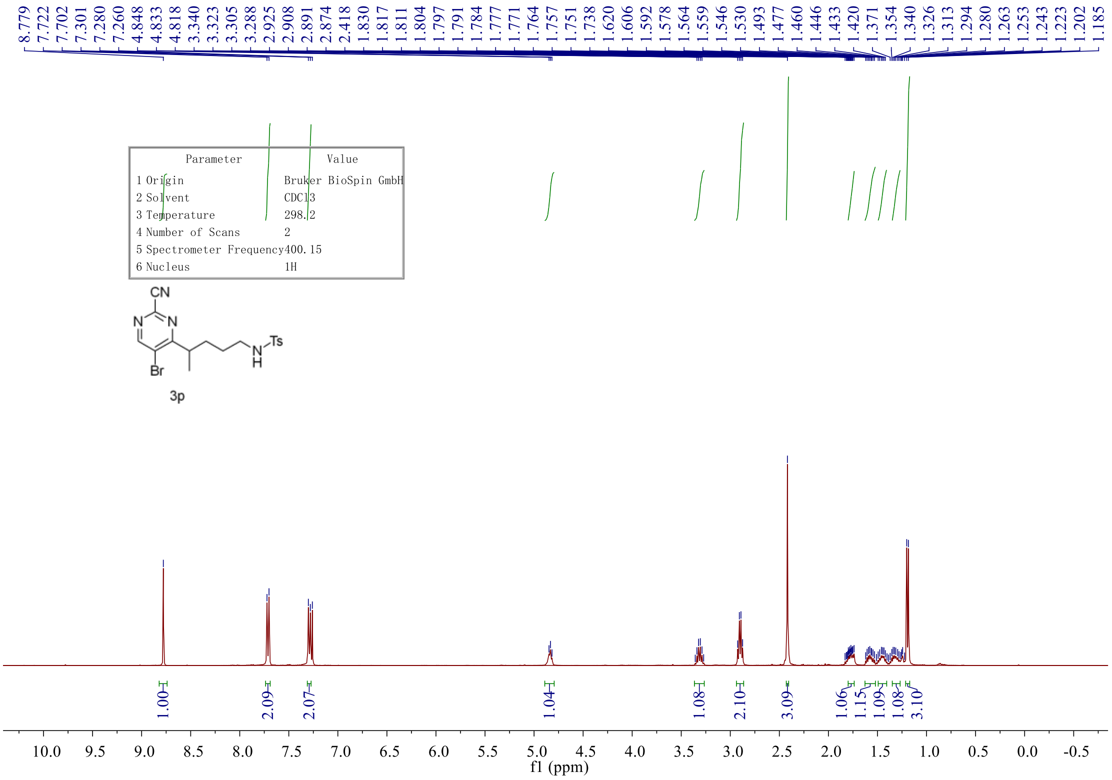
Parameter Value
1 Origin Bruker BioSpin GmbH
2 Solvent CDCl3
3 Temperature 298.2
4 Number of Scans 2
5 Spectrometer Frequency 400.15
6 Nucleus 1H
3p
f1 (ppm)

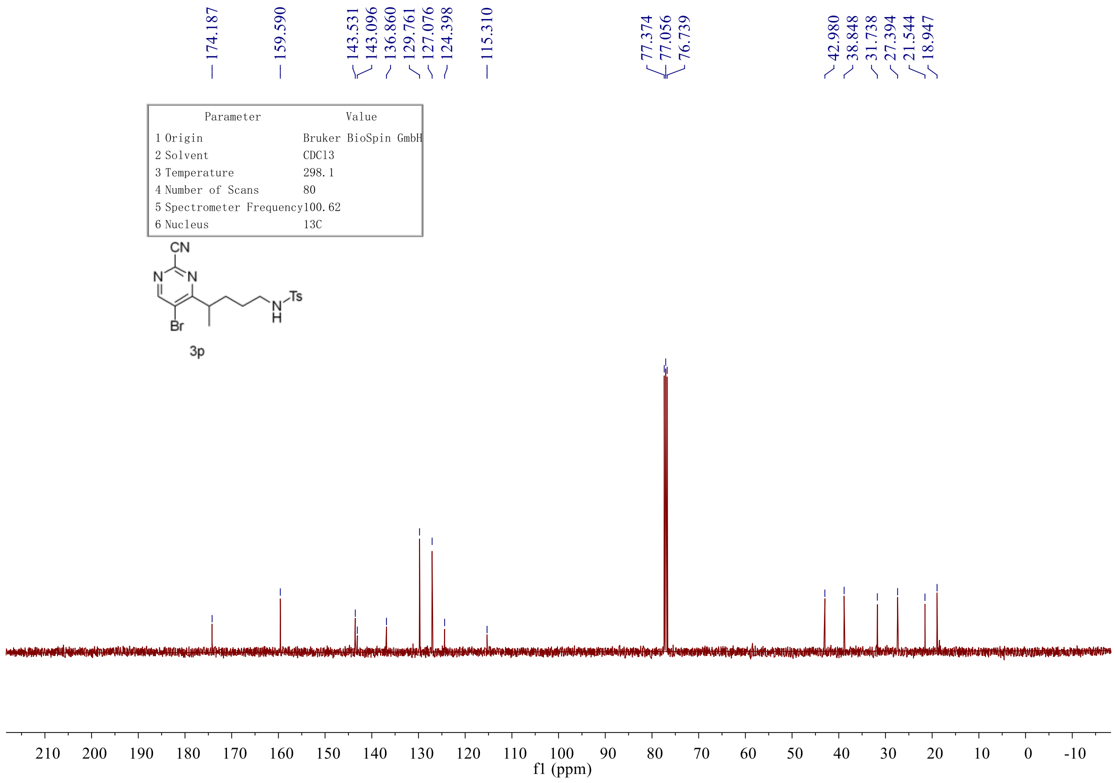
174.187
159.590
143.531
143.096
136.860
129.761
127.076
124.398
115.310
77.374
77.056
76.739
42.980
38.848
31.738
27.394
21.544
18.947
Parameter Value
1 Origin Bruker BioSpin GmbH
2 Solvent CDCl3
3 Temperature 298.1
4 Number of Scans 80
5 Spectrometer Frequency 100.62
6 Nucleus 13C
CN
N
N
N
Ts
H
Br
3p
210 200 190 180 170 160 150 140 130 120 110 100 90 80 70 60 50 40 30 20 10 0 -10
f1 (ppm)

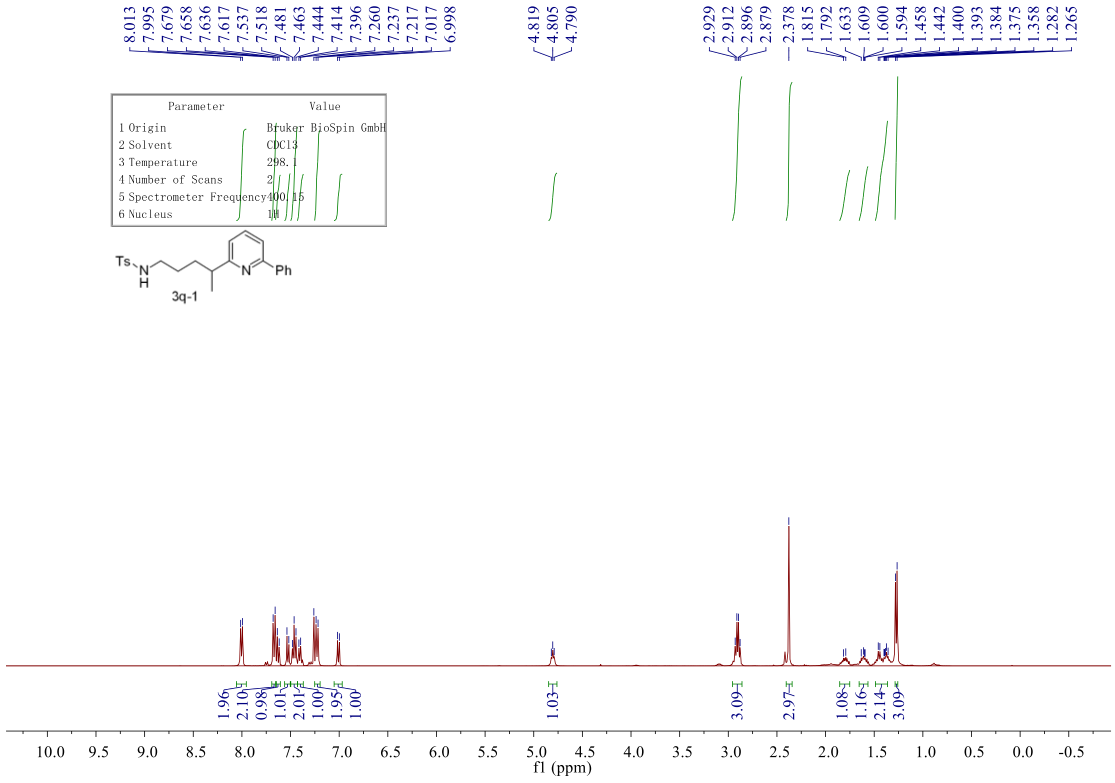
8.013
7.995
7.679
7.658
7.636
7.617
7.537
7.518
7.481
7.463
7.444
7.414
7.396
7.260
7.237
7.217
7.017
6.998
4.819
4.805
4.790
2.929
2.912
2.896
2.879
2.378
1.815
1.792
1.633
1.609
1.600
1.594
1.458
1.442
1.400
1.393
1.384
1.375
1.358
1.282
1.265
Parameter Value
1 Origin Bruker BioSpin GmbH
2 Solvent CDCl3
3 Temperature 298.1
4 Number of Scans 2
5 Spectrometer Frequency 400.15
6 Nucleus 1H
Ts
N
H
N
Ph
3q-1
1.96
2.10
0.98
1.01
2.01
1.00
1.95
1.00
1.03
3.09
2.97
1.08
1.16
2.14
3.09
10.0 9.5 9.0 8.5 8.0 7.5 7.0 6.5 6.0 5.5 5.0 4.5 4.0 3.5 3.0 2.5 2.0 1.5 1.0 0.5 0.0 -0.5
f1 (ppm)

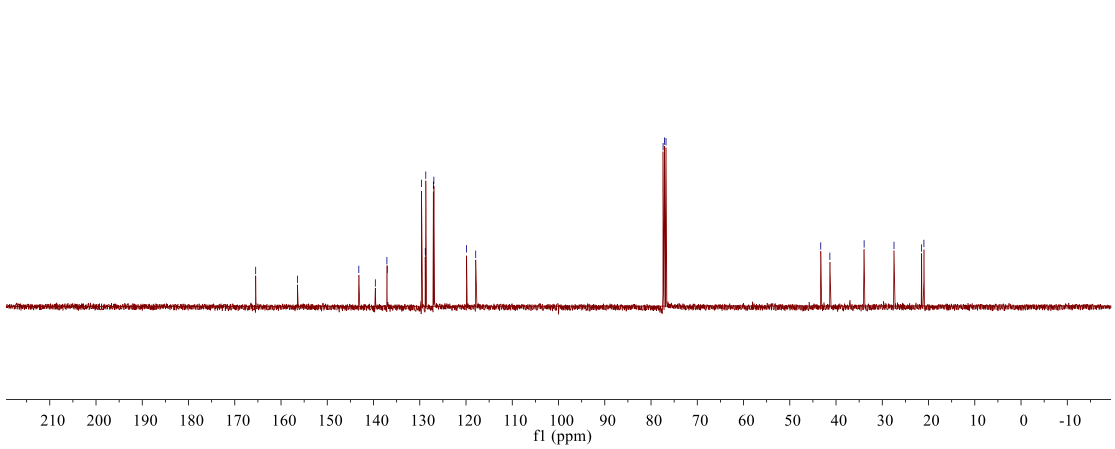

Parameter	Value
1 Origin	Bruker BioSpin GmbH
2 Solvent	CDCl3
3 Temperature	298.2
4 Number of Scans	48
5 Spectrometer Frequency	100.61
6 Nucleus	13C

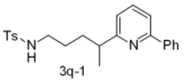

3q-1

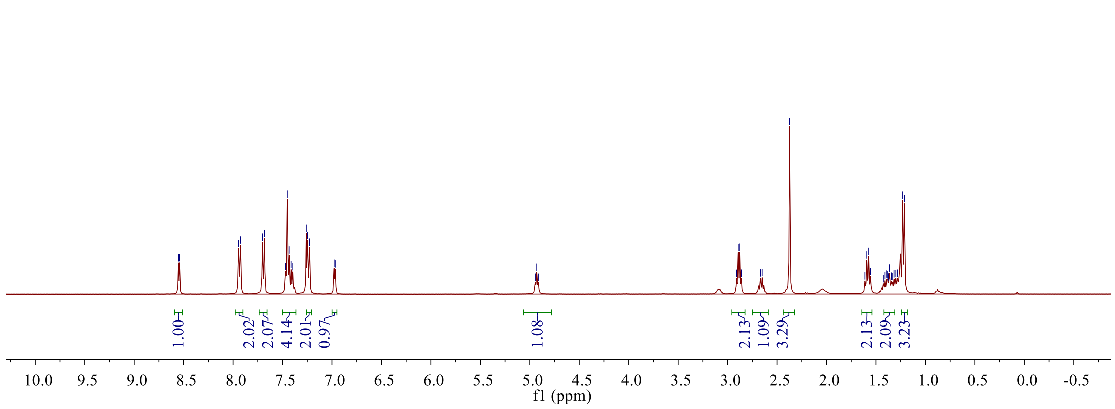

Parameter	Value
1 Origin	Bruker BioSpin GmbH
2 Solvent	CDCl3
3 Temperature	298.2
4 Number of Scans	2
5 Spectrometer Frequency	400.15
6 Nucleus	1H

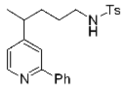

3q-2

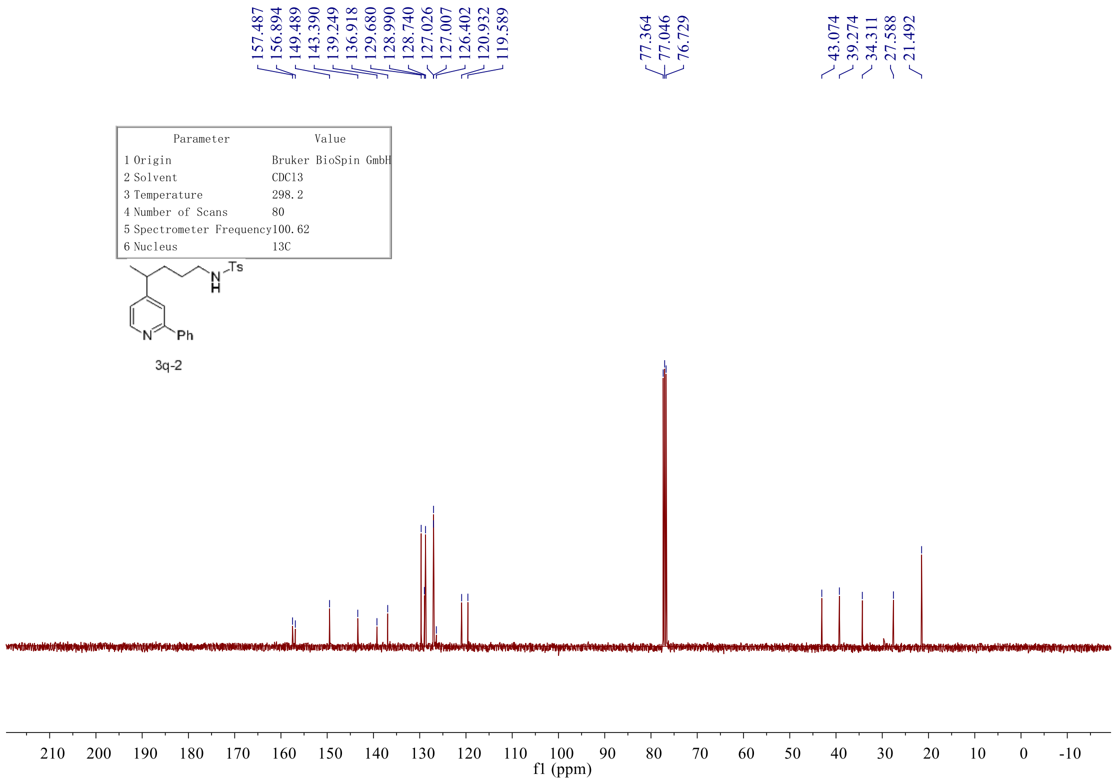
157.487
156.894
149.489
143.390
139.249
136.918
129.680
128.990
128.740
127.026
127.007
126.402
120.932
119.589
77.364
77.046
76.729
43.074
39.274
34.311
27.588
21.492
Parameter Value
1 Origin Bruker BioSpin GmbH
2 Solvent CDCl3
3 Temperature 298.2
4 Number of Scans 80
5 Spectrometer Frequency 100.62
6 Nucleus 13C
Ts
N
H
N
Ph
3q-2
f1 (ppm)

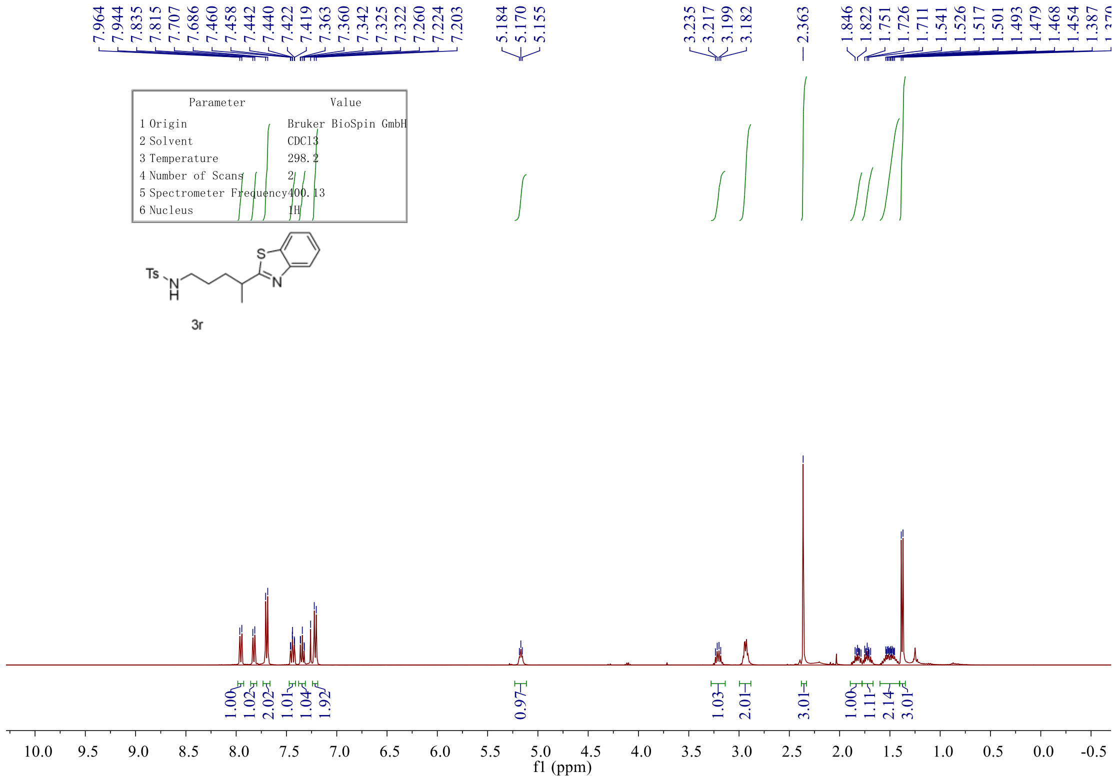
7.964
7.944
7.835
7.815
7.707
7.686
7.460
7.458
7.442
7.440
7.422
7.419
7.363
7.360
7.342
7.325
7.322
7.260
7.224
7.203
5.184
5.170
5.155
3.235
3.217
3.199
3.182
2.363
1.846
1.822
1.751
1.726
1.711
1.541
1.526
1.517
1.501
1.493
1.479
1.468
1.454
1.387
Parameter Value
1 Origin Bruker BioSpin GmbH
2 Solvent CDCl3
3 Temperature 298.2
4 Number of Scans 2
5 Spectrometer Frequency 400.13
6 Nucleus 1H
Ts
N
H
S
N
3r
1.00
1.02
2.02
1.01
1.04
1.92
0.97
1.03
2.01
3.01
1.00
1.11
2.14
3.01
f1 (ppm)

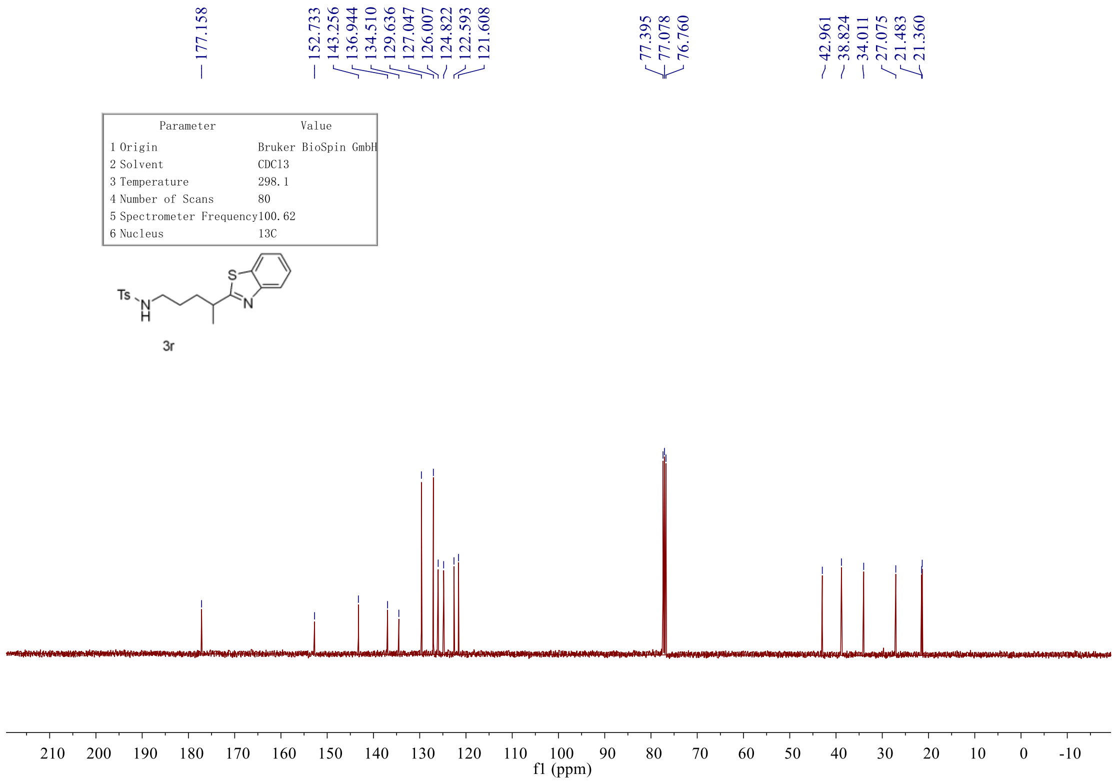
177.158
152.733
143.256
136.944
134.510
129.636
127.047
126.007
124.822
122.593
121.608
77.395
77.078
76.760
42.961
38.824
34.011
27.075
21.483
21.360
Parameter Value
1 Origin Bruker BioSpin GmbH
2 Solvent CDCl3
3 Temperature 298.1
4 Number of Scans 80
5 Spectrometer Frequency 100.62
6 Nucleus 13C
Ts
N
H
S
N
3r
210 200 190 180 170 160 150 140 130 120 110 100 90 80 70 60 50 40 30 20 10 0 -10
f1 (ppm)

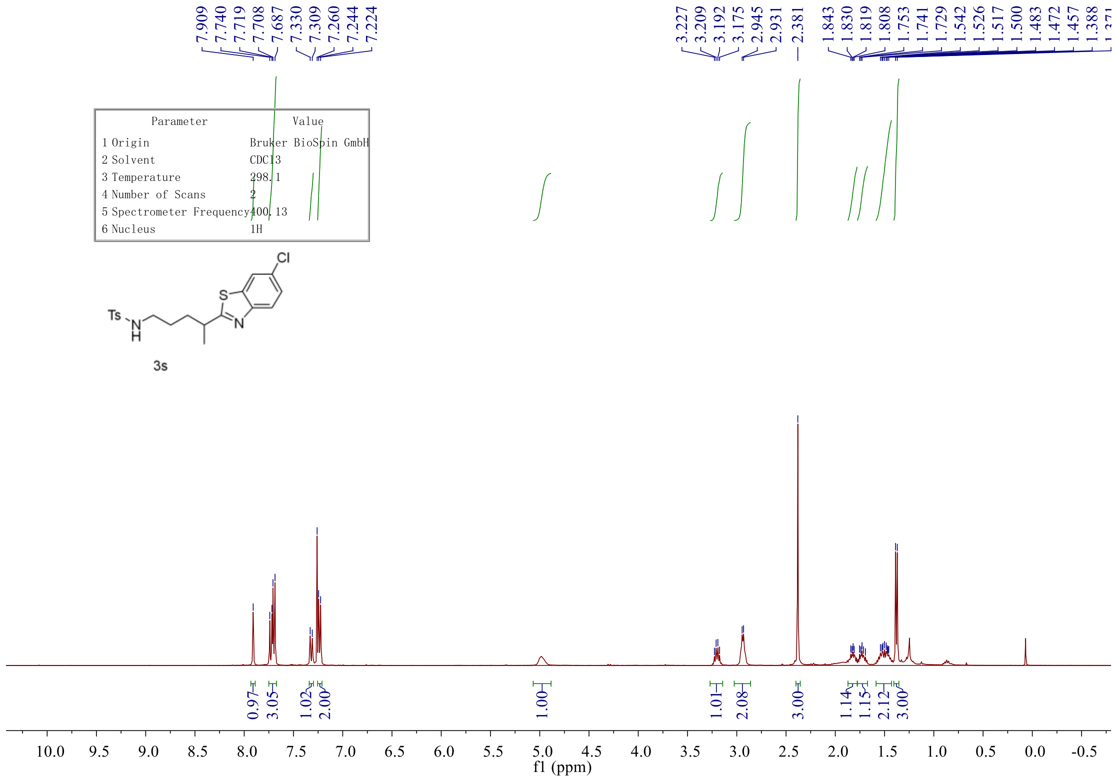
7.909
7.740
7.719
7.708
7.687
7.330
7.309
7.260
7.244
7.224
3.227
3.209
3.192
3.175
2.945
2.931
2.381
1.843
1.830
1.819
1.808
1.753
1.741
1.729
1.542
1.526
1.517
1.500
1.483
1.472
1.457
1.388
Parameter Value
1 Origin Bruker BioSpin GmbH
2 Solvent CDCl3
3 Temperature 298.1
4 Number of Scans 2
5 Spectrometer Frequency 400.13
6 Nucleus 1H
Cl
S
N
Ts
N
H
3s
0.97
3.05
1.02
2.00
1.00
1.01
2.08
3.00
1.14
1.15
2.12
3.00
10.0 9.5 9.0 8.5 8.0 7.5 7.0 6.5 6.0 5.5 5.0 4.5 4.0 3.5 3.0 2.5 2.0 1.5 1.0 0.5 0.0 -0.5
f1 (ppm)

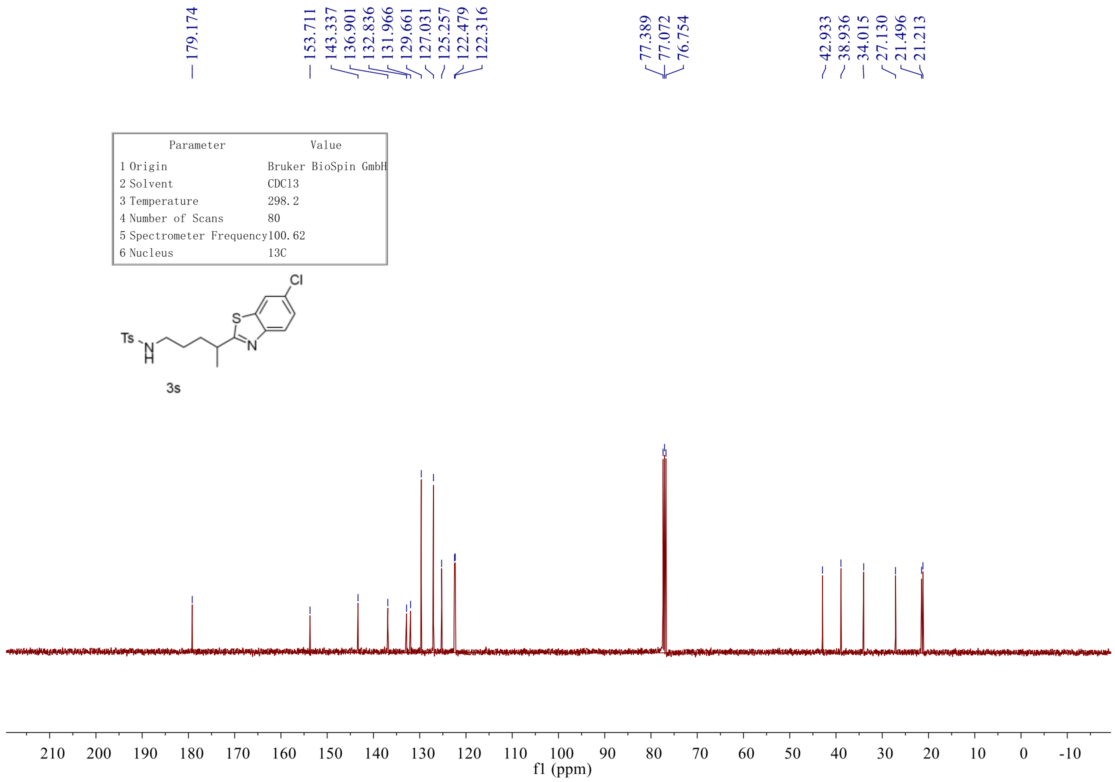
179.174
153.711
143.337
136.901
132.836
131.966
129.661
127.031
125.257
122.479
122.316
77.389
77.072
76.754
42.933
38.936
34.015
27.130
21.496
21.213
Parameter Value
1 Origin Bruker BioSpin GmbH
2 Solvent CDCl3
3 Temperature 298.2
4 Number of Scans 80
5 Spectrometer Frequency 100.62
6 Nucleus 13C
Cl
S
N
Ts
N
H
3s
210 200 190 180 170 160 150 140 130 120 110 100 90 80 70 60 50 40 30 20 10 0 -10
f1 (ppm)

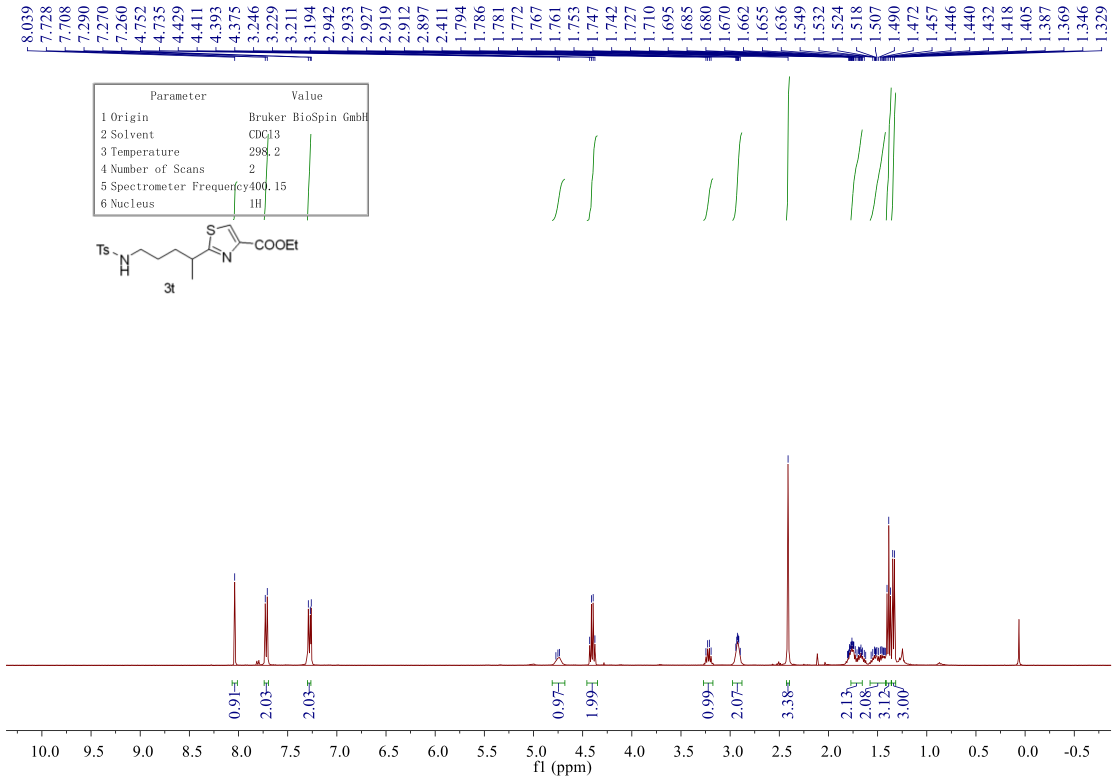
8.039
7.728
7.708
7.290
7.270
7.260
4.752
4.735
4.429
4.411
4.393
4.375
3.246
3.229
3.211
3.194
2.942
2.933
2.927
2.919
2.912
2.897
2.411
1.794
1.786
1.781
1.772
1.767
1.761
1.753
1.747
1.742
1.727
1.710
1.695
1.685
1.680
1.670
1.662
1.655
1.636
1.549
1.532
1.524
1.518
1.507
1.490
1.472
1.457
1.446
1.440
1.432
1.418
1.405
1.387
1.369
1.346
1.329
Parameter Value
1 Origin Bruker BioSpin GmbH
2 Solvent CDCl3
3 Temperature 298.2
4 Number of Scans 2
5 Spectrometer Frequency 400.15
6 Nucleus 1H
S
N
COOEt
Ts
N
H
3t
0.91
2.03
2.03
0.97
1.99
0.99
2.07
3.38
2.13
2.08
3.12
3.00
10.0 9.5 9.0 8.5 8.0 7.5 7.0 6.5 6.0 5.5 5.0 4.5 4.0 3.5 3.0 2.5 2.0 1.5 1.0 0.5 0.0 -0.5
f1 (ppm)

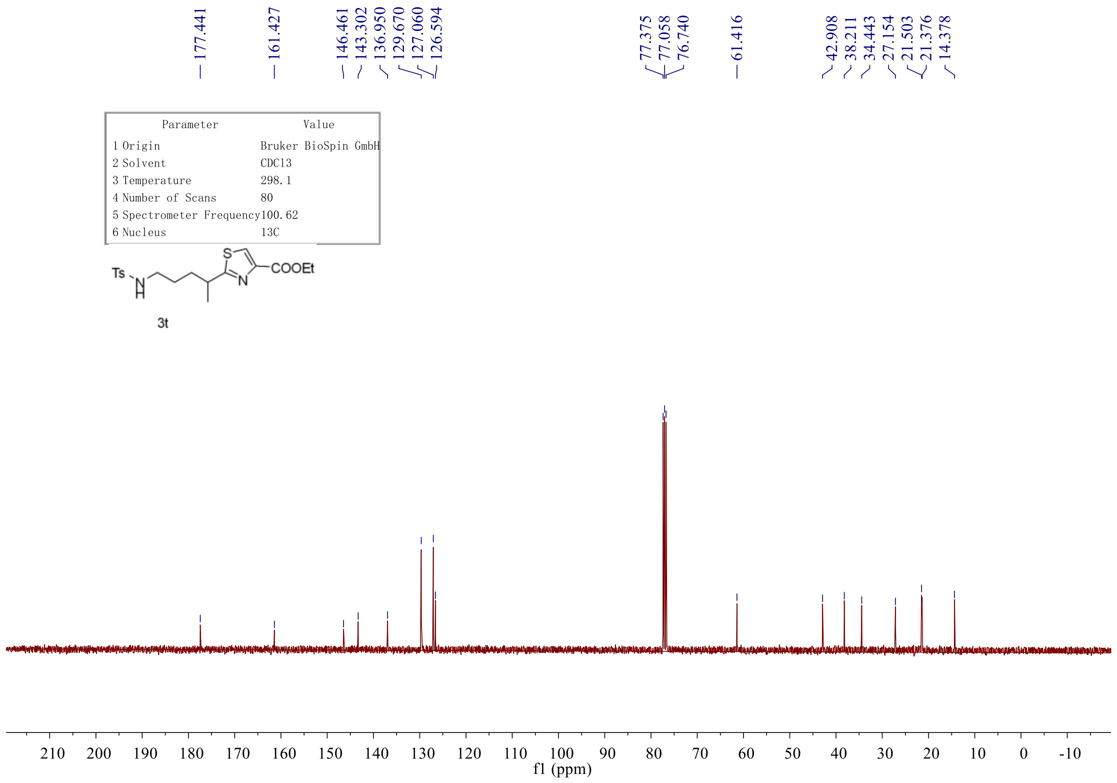
177.441
161.427
146.461
143.302
136.950
129.670
127.060
126.594
77.375
77.058
76.740
61.416
42.908
38.211
34.443
27.154
21.503
21.376
14.378
Parameter Value
1 Origin Bruker BioSpin GmbH
2 Solvent CDCl3
3 Temperature 298.1
4 Number of Scans 80
5 Spectrometer Frequency 100.62
6 Nucleus 13C
Ts
N
H
S
N
COOEt
3t
210 200 190 180 170 160 150 140 130 120 110 100 90 80 70 60 50 40 30 20 10 0 -10
f1 (ppm)

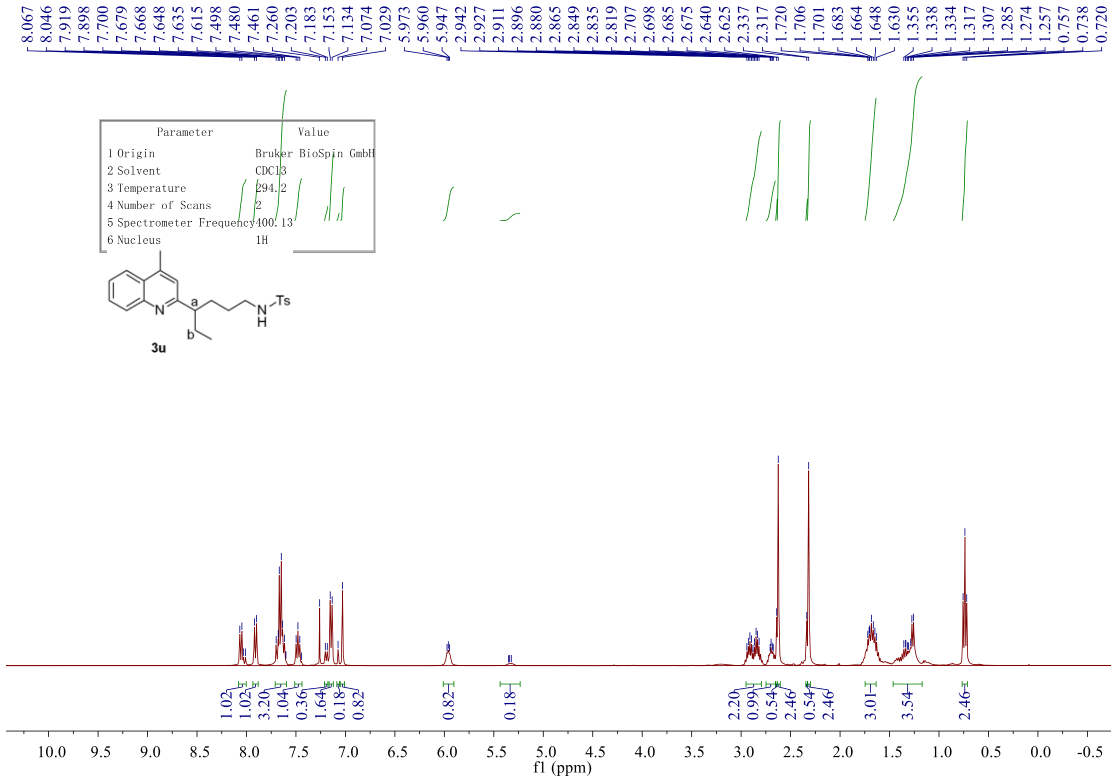
8.067
8.046
7.919
7.898
7.700
7.679
7.668
7.648
7.635
7.615
7.498
7.480
7.461
7.260
7.203
7.183
7.153
7.134
7.074
7.029
5.973
5.960
5.947
2.942
2.927
2.911
2.896
2.880
2.865
2.849
2.835
2.819
2.707
2.698
2.685
2.675
2.640
2.625
2.337
2.317
1.720
1.706
1.701
1.683
1.664
1.648
1.630
1.355
1.338
1.334
1.317
1.307
1.285
1.274
1.257
0.757
0.738
0.720
Parameter Value
1 Origin Bruker BioSpin GmbH
2 Solvent CDCl3
3 Temperature 294.2
4 Number of Scans 2
5 Spectrometer Frequency 400.13
6 Nucleus 1H
N
a
b
N
H
Ts
3u
1.02
1.02
3.20
1.04
0.36
1.64
0.18
0.82
0.82
0.18
2.20
0.99
0.54
2.46
0.54
2.46
3.01
3.54
2.46
10.0 9.5 9.0 8.5 8.0 7.5 7.0 6.5 6.0 5.5 5.0 4.5 4.0 3.5 3.0 2.5 2.0 1.5 1.0 0.5 0.0 -0.5
f1 (ppm)

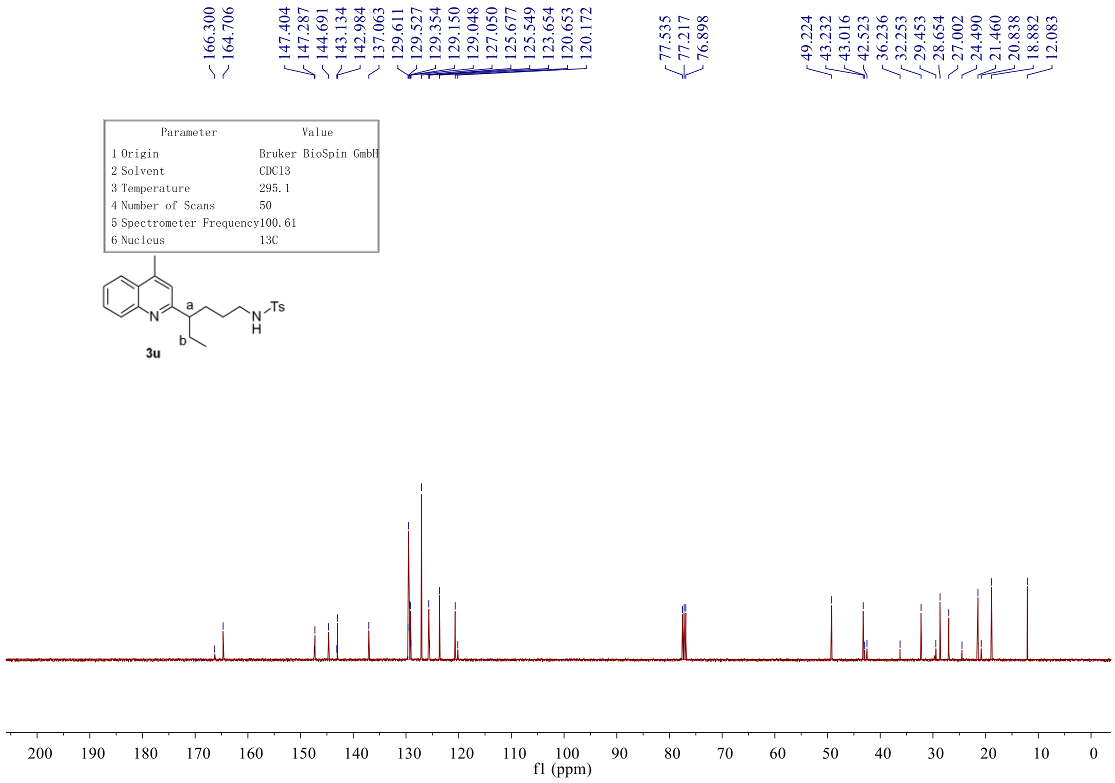
166.300
164.706
147.404
147.287
144.691
143.134
142.984
137.063
129.611
129.527
129.354
129.150
129.048
127.050
125.677
125.549
123.654
120.653
120.172
77.535
77.217
76.898
49.224
43.232
43.016
42.523
36.236
32.253
29.453
28.654
27.002
24.490
21.460
20.838
18.882
12.083
Parameter Value
1 Origin Bruker BioSpin GmbH
2 Solvent CDCl3
3 Temperature 295.1
4 Number of Scans 50
5 Spectrometer Frequency 100.61
6 Nucleus 13C
a
b
N
Ts
H
3u
200 190 180 170 160 150 140 130 120 110 100 90 80 70 60 50 40 30 20 10 0
f1 (ppm)

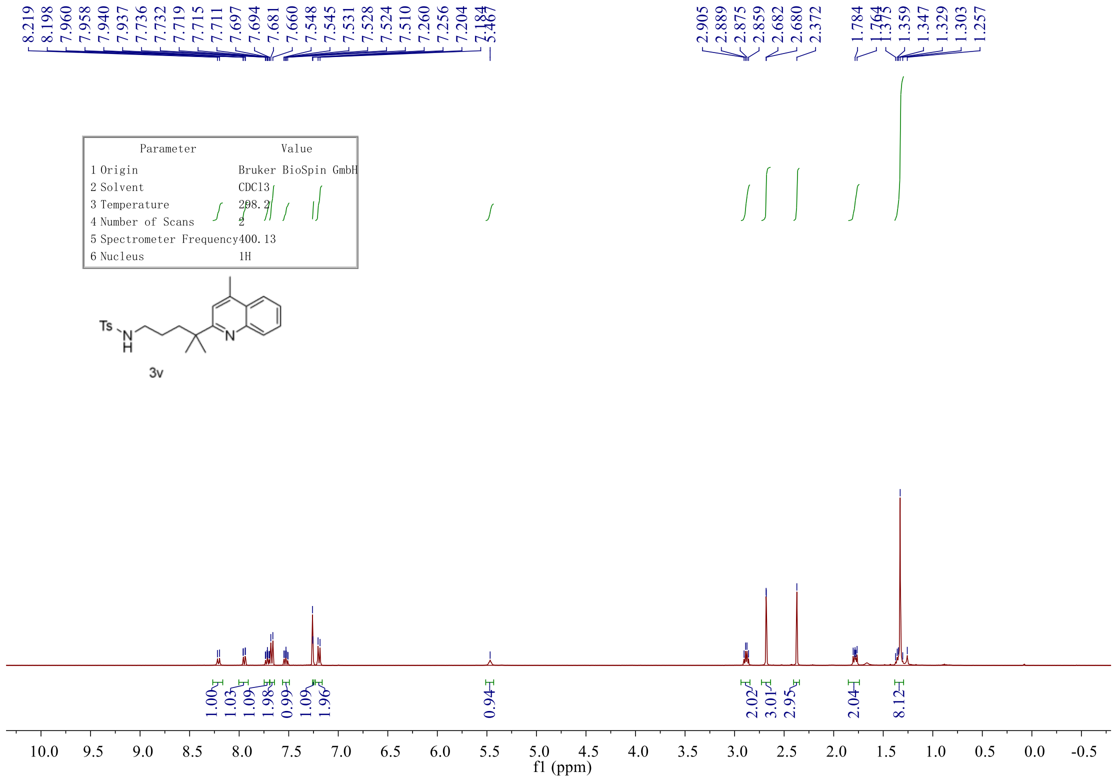
8.219
8.198
7.960
7.958
7.940
7.937
7.736
7.732
7.719
7.715
7.711
7.697
7.694
7.681
7.660
7.548
7.545
7.531
7.528
7.524
7.510
7.260
7.256
7.204
7.184
5.467
2.905
2.889
2.875
2.859
2.682
2.680
2.372
1.784
1.764
1.375
1.359
1.347
1.329
1.303
1.257
Parameter Value
1 Origin Bruker BioSpin GmbH
2 Solvent CDCl3
3 Temperature 298.2
4 Number of Scans 2
5 Spectrometer Frequency 400.13
6 Nucleus 1H
Ts
N
H
3v
1.00
1.03
1.09
1.98
0.99
1.09
1.96
0.94
2.02
3.01
2.95
2.04
8.12
10.0 9.5 9.0 8.5 8.0 7.5 7.0 6.5 6.0 5.5 5.0 4.5 4.0 3.5 3.0 2.5 2.0 1.5 1.0 0.5 0.0 -0.5
f1 (ppm)

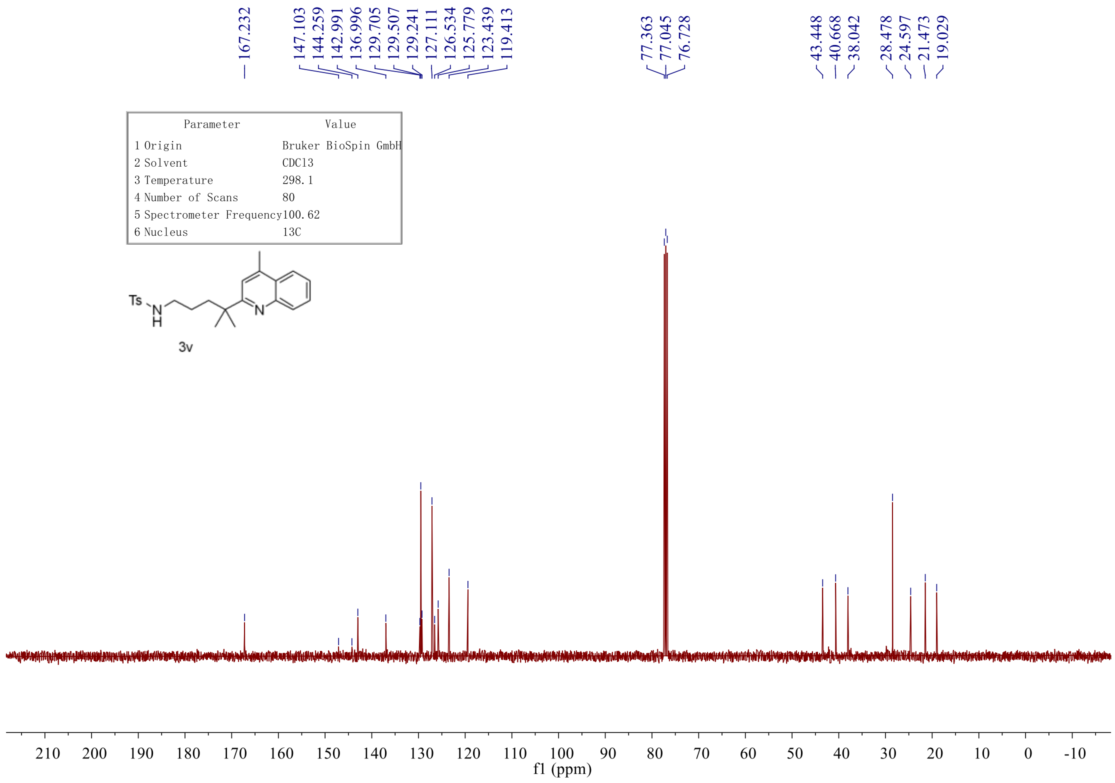
167.232
147.103 144.259 142.991 136.996 129.705 129.507 129.241 127.111 126.534 125.779 123.439 119.413
77.363 77.045 76.728
43.448 40.668 38.042
28.478 24.597 21.473 19.029
Parameter Value
1 Origin Bruker BioSpin GmbH
2 Solvent CDCl3
3 Temperature 298.1
4 Number of Scans 80
5 Spectrometer Frequency 100.62
6 Nucleus 13C
Ts
N
H
N
3v
210 200 190 180 170 160 150 140 130 120 110 100 90 80 70 60 50 40 30 20 10 0 -10
f1 (ppm)

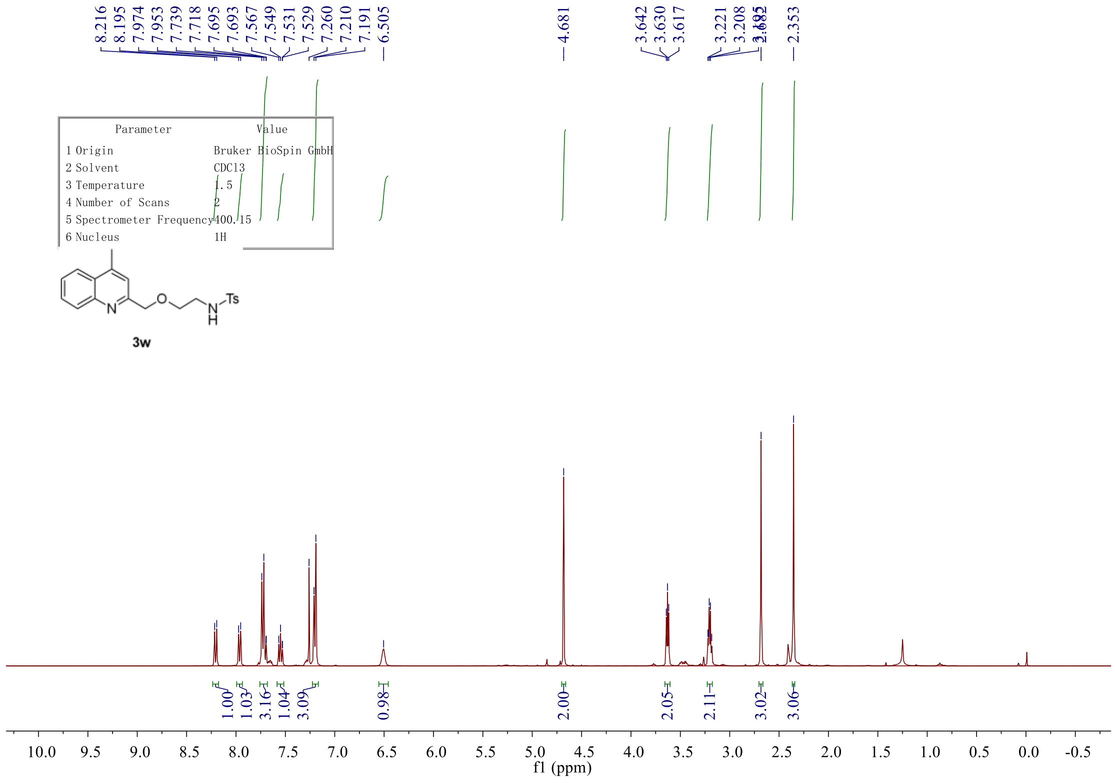
8.216 8.195 7.974 7.953 7.739 7.718 7.695 7.693 7.567 7.549 7.531 7.529 7.260 7.210 7.191 6.505
4.681
3.642 3.630 3.617
3.221 3.208
2.353
Parameter Value
1 Origin Bruker BioSpin GmbH
2 Solvent CDCl3
4 Number of Scans
5 Spectrometer Frequency 400.15
6 Nucleus 1H
N
O
N
H
Ts
3w
1.00 1.03 3.16 1.04 3.09 0.98 2.00 2.05 2.11 3.02 3.06
10.0 9.5 9.0 8.5 8.0 7.5 7.0 6.5 6.0 5.5 5.0 4.5 4.0 3.5 3.0 2.5 2.0 1.5 1.0 0.5 0.0 -0.5
f1 (ppm)

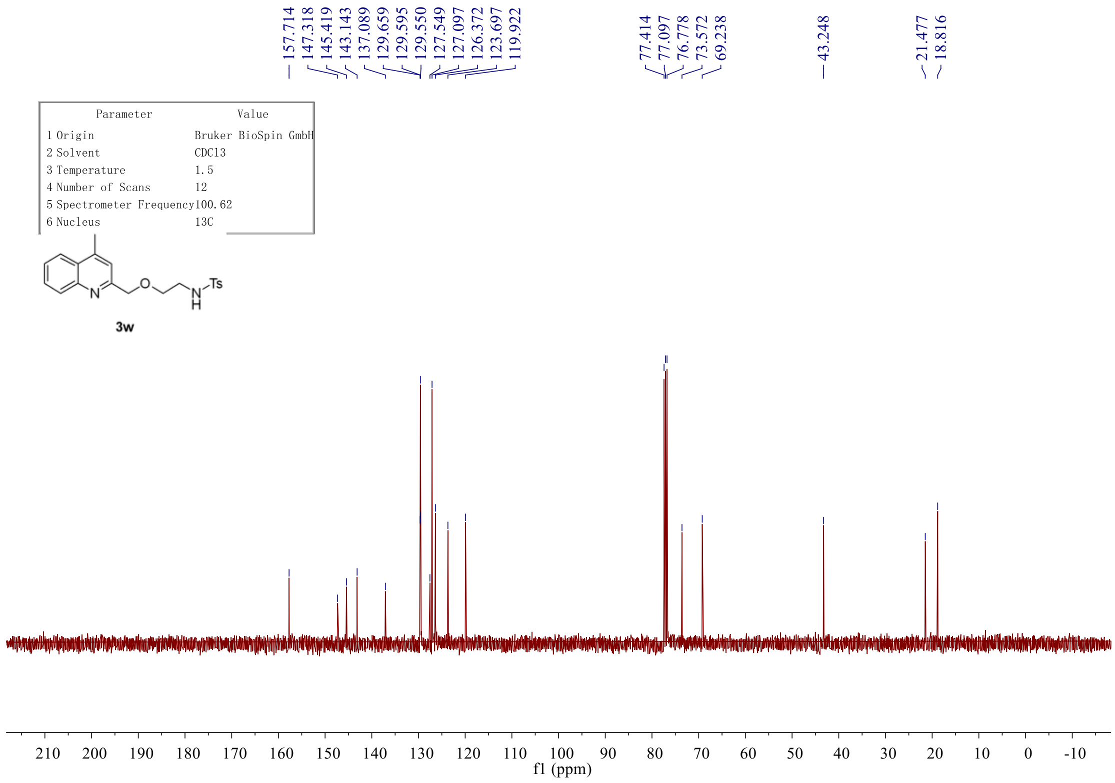
157.714
147.318
145.419
143.143
137.089
129.659
129.595
129.550
127.549
127.097
126.372
123.697
119.922
77.414
77.097
76.778
73.572
69.238
43.248
21.477
18.816
Parameter Value
1 Origin Bruker BioSpin GmbH
2 Solvent CDCl3
3 Temperature 1.5
4 Number of Scans 12
5 Spectrometer Frequency 100.62
6 Nucleus 13C
N
O
N
H
Ts
3w
f1 (ppm)

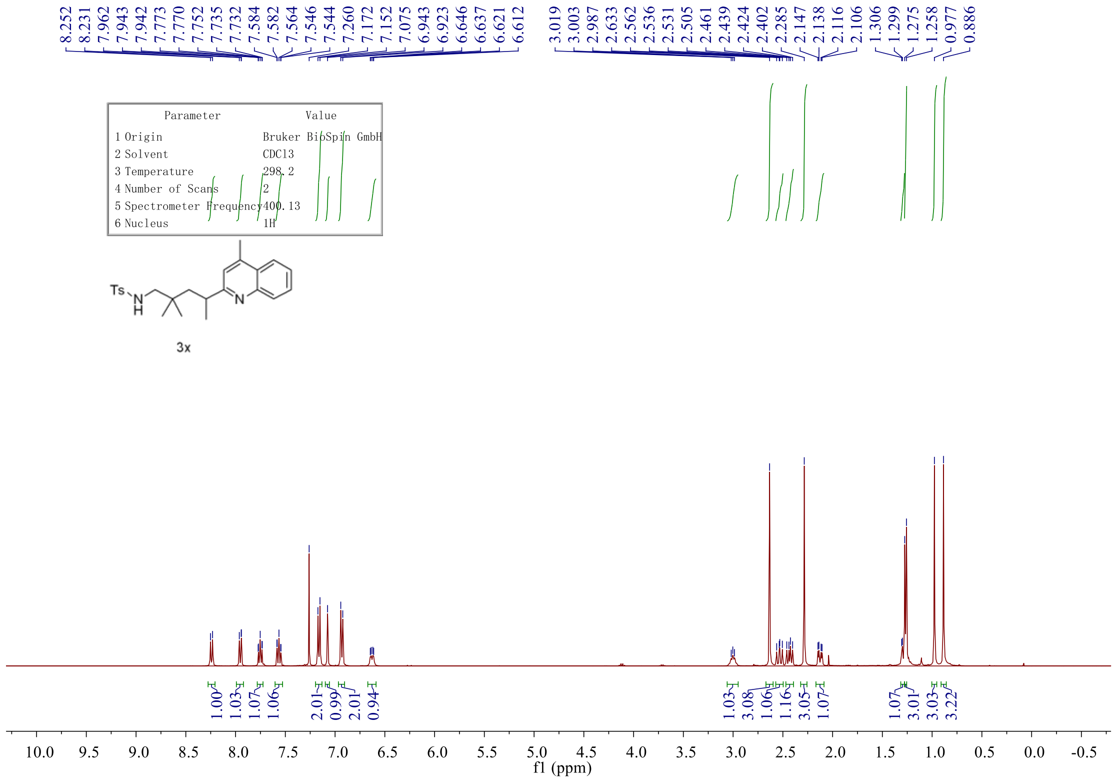
8.252
8.231
7.962
7.943
7.942
7.773
7.770
7.752
7.735
7.732
7.584
7.582
7.564
7.546
7.544
7.260
7.172
7.152
7.075
6.943
6.923
6.646
6.637
6.621
6.612
3.019
3.003
2.987
2.633
2.562
2.536
2.531
2.505
2.461
2.439
2.424
2.402
2.285
2.147
2.138
2.116
2.106
1.306
1.299
1.275
1.258
0.977
0.886
Parameter Value
1 Origin Bruker BioSpin GmbH
2 Solvent CDCl3
3 Temperature 298.2
4 Number of Scans 2
5 Spectrometer Frequency 400.13
6 Nucleus 1H
Ts
N
H
N
3x
1.00
1.03
1.07
1.06
2.01
0.99
2.01
0.94
1.03
3.08
1.06
1.16
3.05
1.07
1.07
3.01
3.03
3.22
f1 (ppm)

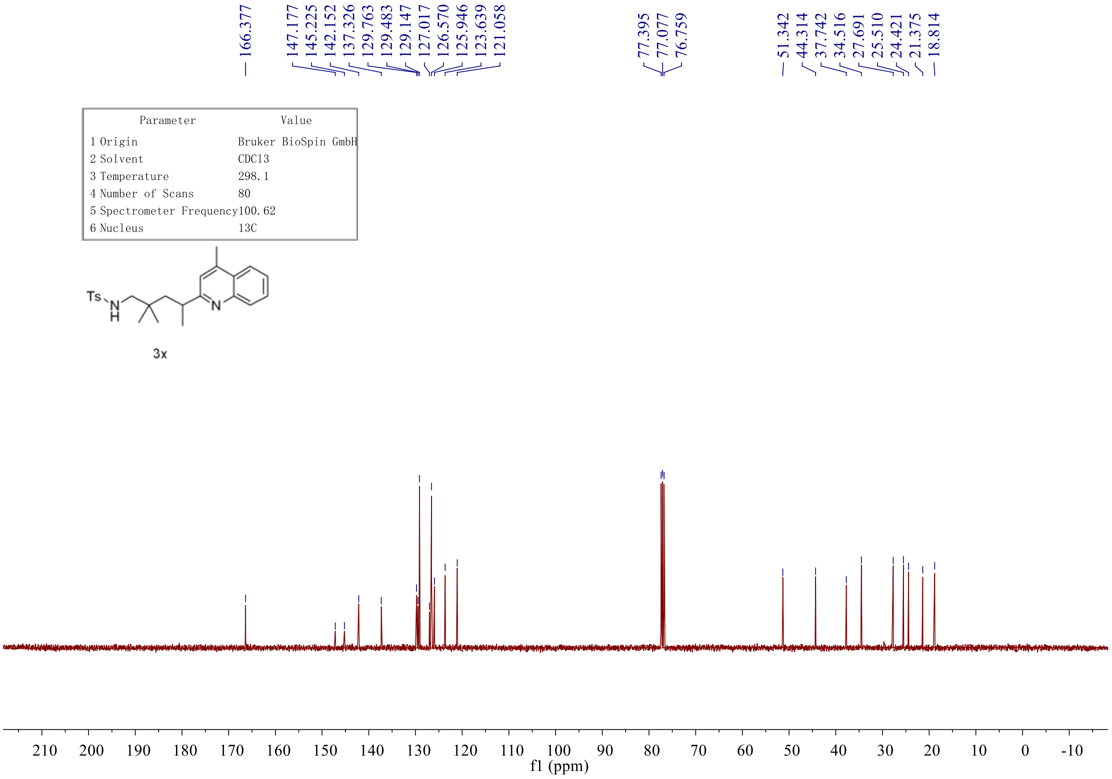
166.377
147.177
145.225
142.152
137.326
129.763
129.483
129.147
127.017
126.570
125.946
123.639
121.058
77.395
77.077
76.759
51.342
44.314
37.742
34.516
27.691
25.510
24.421
21.375
18.814
Parameter Value
1 Origin Bruker BioSpin GmbH
2 Solvent CDCl3
3 Temperature 298.1
4 Number of Scans 80
5 Spectrometer Frequency 100.62
6 Nucleus 13C
Ts
N
H
N
3x
f1 (ppm)

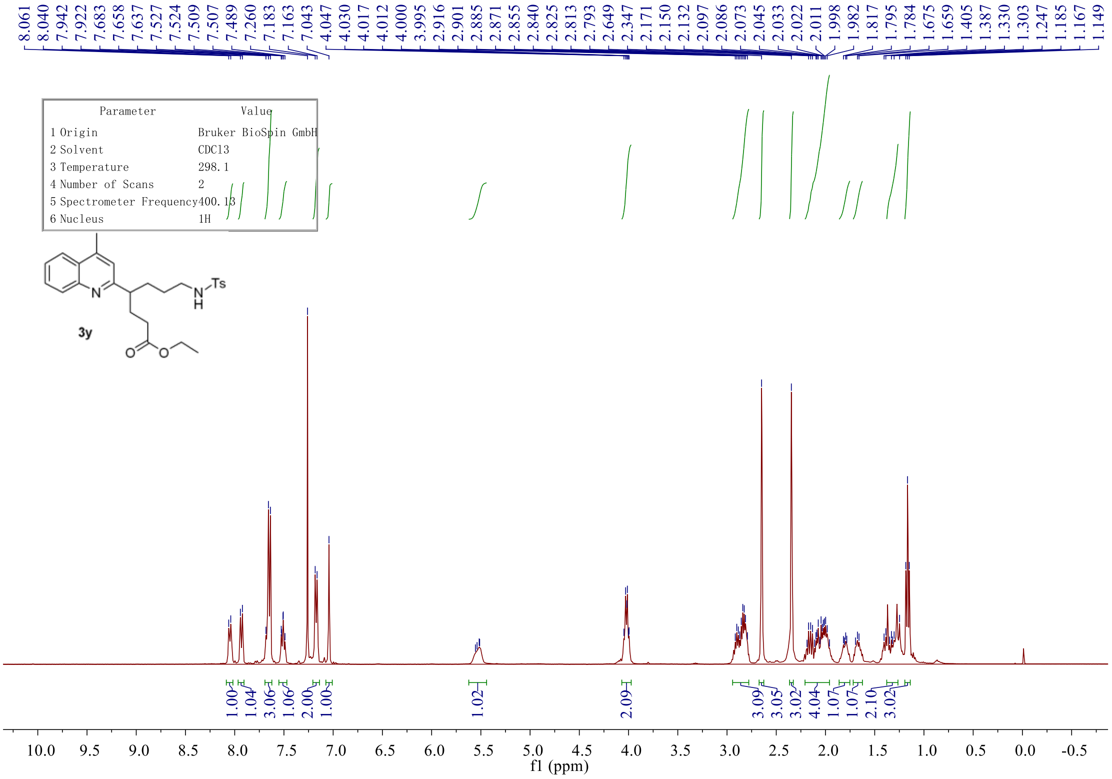
8.061
8.040
7.942
7.922
7.683
7.658
7.637
7.527
7.524
7.509
7.507
7.489
7.260
7.183
7.163
7.043
4.047
4.030
4.017
4.012
4.000
3.995
2.916
2.901
2.885
2.871
2.855
2.840
2.825
2.813
2.793
2.649
2.347
2.171
2.150
2.132
2.097
2.086
2.073
2.045
2.033
2.022
2.011
1.998
1.982
1.817
1.795
1.784
1.675
1.659
1.405
1.387
1.330
1.303
1.247
1.185
1.167
1.149
Parameter Value
1 Origin Bruker BioSpin GmbH
2 Solvent CDCl3
3 Temperature 298.1
4 Number of Scans 2
5 Spectrometer Frequency 400.13
6 Nucleus 1H
N
N
H
Ts
3y
O
O
1.00
1.04
3.06
1.06
2.00
1.00
1.02
2.09
3.09
3.05
3.02
4.04
1.07
1.07
2.10
3.02
f1 (ppm)

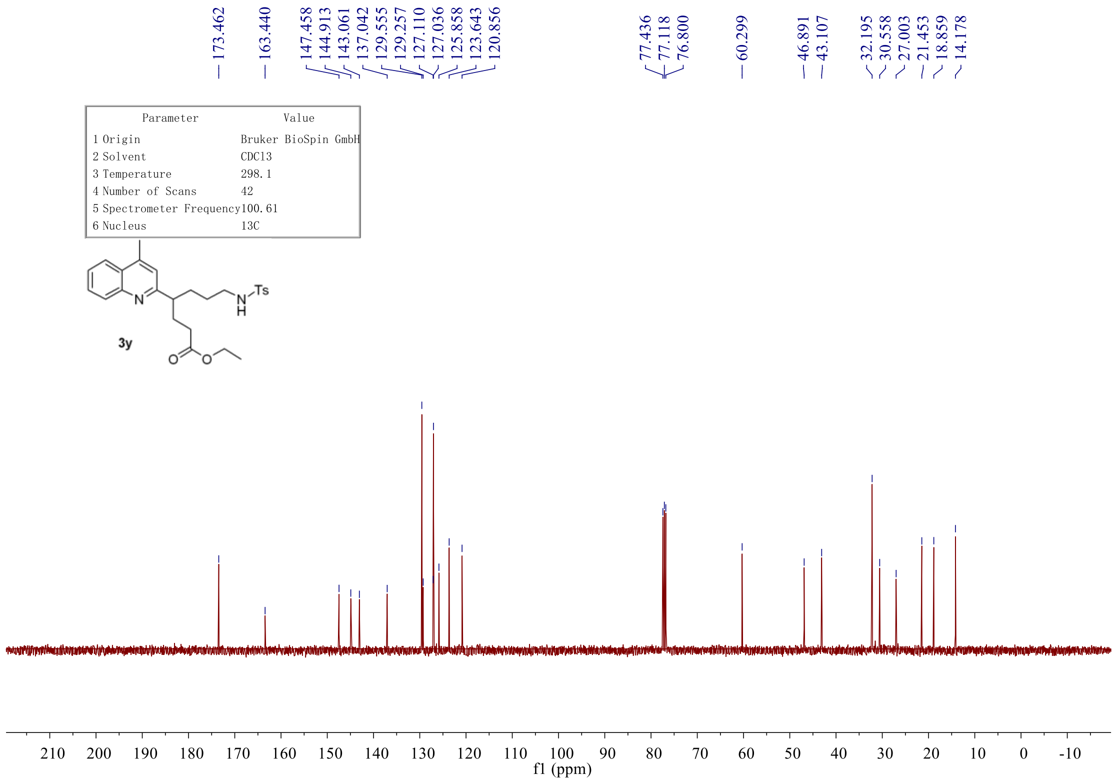
173.462
163.440
147.458
144.913
143.061
137.042
129.555
129.257
127.110
127.036
125.858
123.643
120.856
77.436
77.118
76.800
60.299
46.891
43.107
32.195
30.558
27.003
21.453
18.859
14.178
Parameter Value
1 Origin Bruker BioSpin GmbH
2 Solvent CDCl3
3 Temperature 298.1
4 Number of Scans 42
5 Spectrometer Frequency 100.61
6 Nucleus 13C
3y
f1 (ppm)

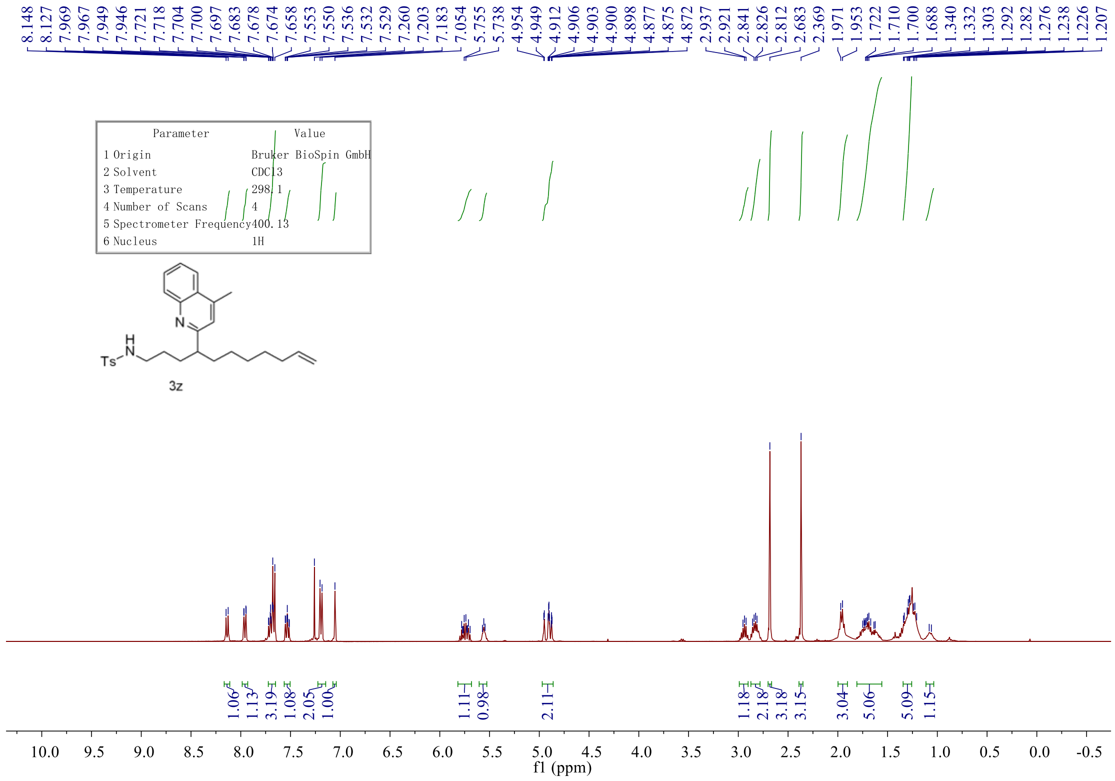
8.148
8.127
7.969
7.967
7.949
7.946
7.721
7.718
7.704
7.700
7.697
7.683
7.678
7.674
7.658
7.553
7.550
7.536
7.532
7.529
7.260
7.203
7.183
7.054
5.755
5.738
4.954
4.949
4.912
4.906
4.903
4.900
4.898
4.877
4.875
4.872
2.937
2.921
2.841
2.826
2.812
2.683
2.369
1.971
1.953
1.722
1.710
1.700
1.688
1.340
1.332
1.303
1.292
1.282
1.276
1.238
1.226
1.207
Parameter Value
1 Origin Bruker BioSpin GmbH
2 Solvent CDCl3
3 Temperature 298.1
4 Number of Scans 4
5 Spectrometer Frequency 400.13
6 Nucleus 1H
3z
1.06
1.13
3.19
1.08
2.05
1.00
1.11
0.98
2.11
1.18
2.18
3.18
3.15
3.04
5.06
5.09
1.15
f1 (ppm)

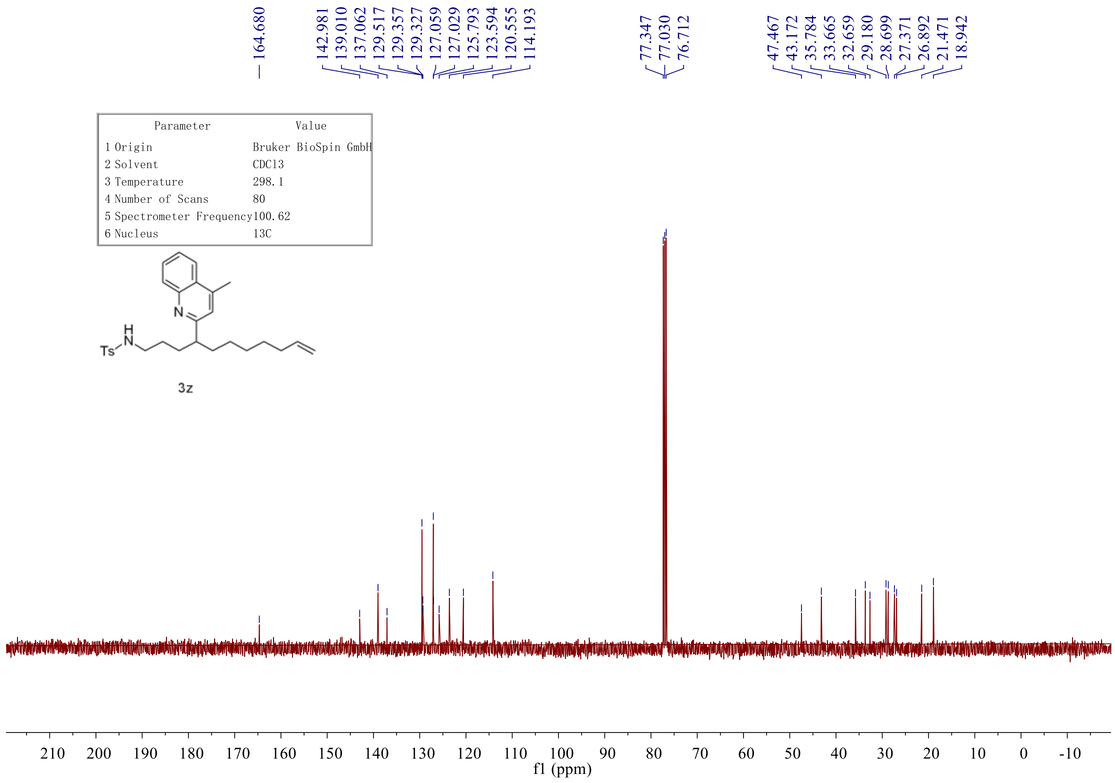
164.680
142.981 139.010 137.062 129.517 129.357 129.327 127.059 127.029 125.793 123.594 120.555 114.193
77.347 77.030 76.712
47.467 43.172 35.784 33.665 32.659 29.180 28.699 27.371 26.892 21.471 18.942
Parameter Value
1 Origin Bruker BioSpin GmbH
2 Solvent CDCl3
3 Temperature 298.1
4 Number of Scans 80
5 Spectrometer Frequency 100.62
6 Nucleus 13C
Ts N H
N
3z
f1 (ppm)

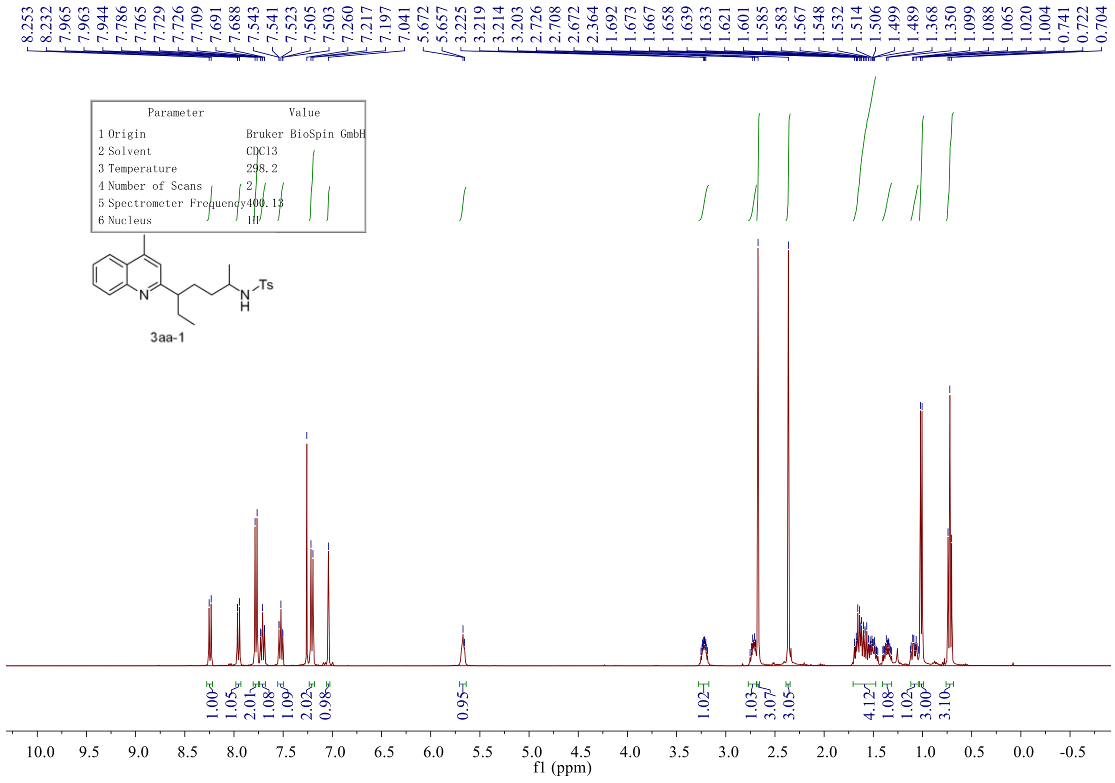
8.253 8.232 7.965 7.963 7.944 7.786 7.765 7.729 7.726 7.709 7.691 7.688 7.543 7.541 7.523 7.505 7.503 7.260 7.217 7.197 7.041 5.672 5.657 3.225 3.219 3.214 3.203 2.726 2.708 2.672 2.364 1.692 1.673 1.667 1.658 1.639 1.633 1.621 1.601 1.585 1.583 1.567 1.548 1.532 1.514 1.506 1.499 1.489 1.368 1.350 1.099 1.088 1.065 1.020 1.004 0.741 0.722 0.704
Parameter Value
1 Origin Bruker BioSpin GmbH
2 Solvent CDCl3
3 Temperature 298.2
4 Number of Scans 2
5 Spectrometer Frequency 400.13
6 Nucleus 1H
N
N H Ts
3aa-1
1.00 1.05 2.01 1.08 1.09 2.02 0.98 0.95 1.02 1.03 3.07 3.05 4.12 1.08 1.02 3.00 3.10
f1 (ppm)

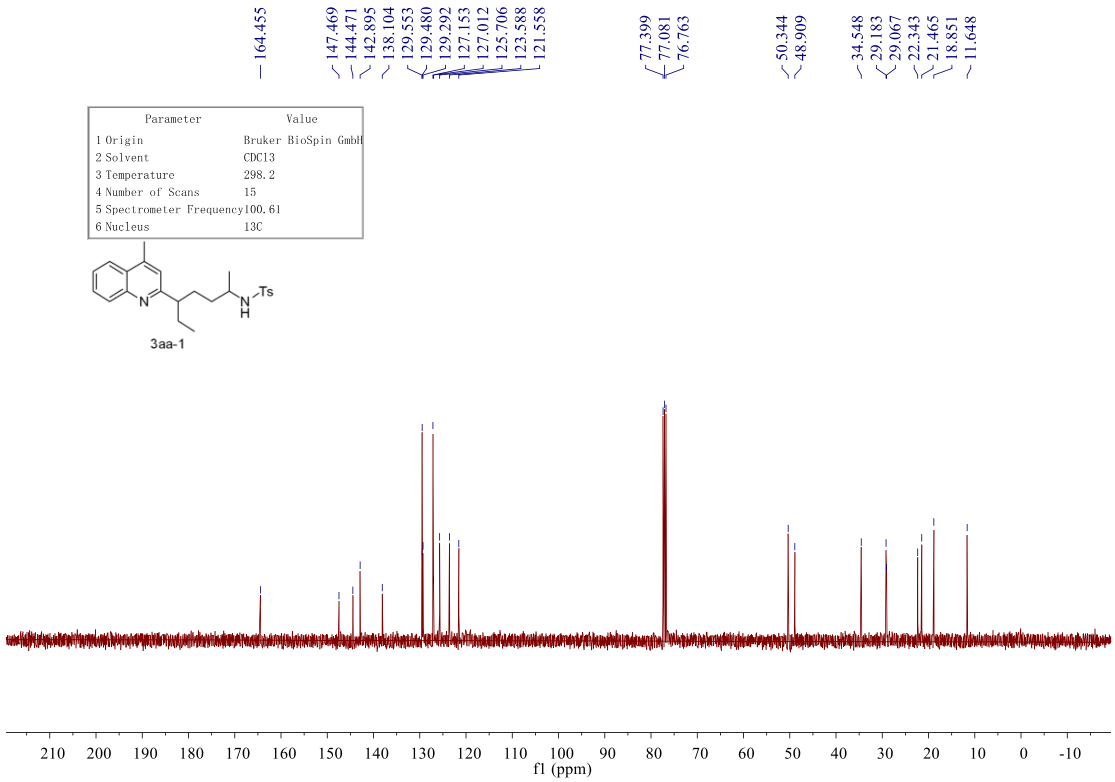
164.455
147.469
144.471
142.895
138.104
129.553
129.480
129.292
127.153
127.012
125.706
123.588
121.558
77.399
77.081
76.763
50.344
48.909
34.548
29.183
29.067
22.343
21.465
18.851
11.648
Parameter Value
1 Origin Bruker BioSpin GmbH
2 Solvent CDCl3
3 Temperature 298.2
4 Number of Scans 15
5 Spectrometer Frequency 100.61
6 Nucleus 13C
N
Ts
N
H
3aa-1
210 200 190 180 170 160 150 140 130 120 110 100 90 80 70 60 50 40 30 20 10 0 -10
f1 (ppm)

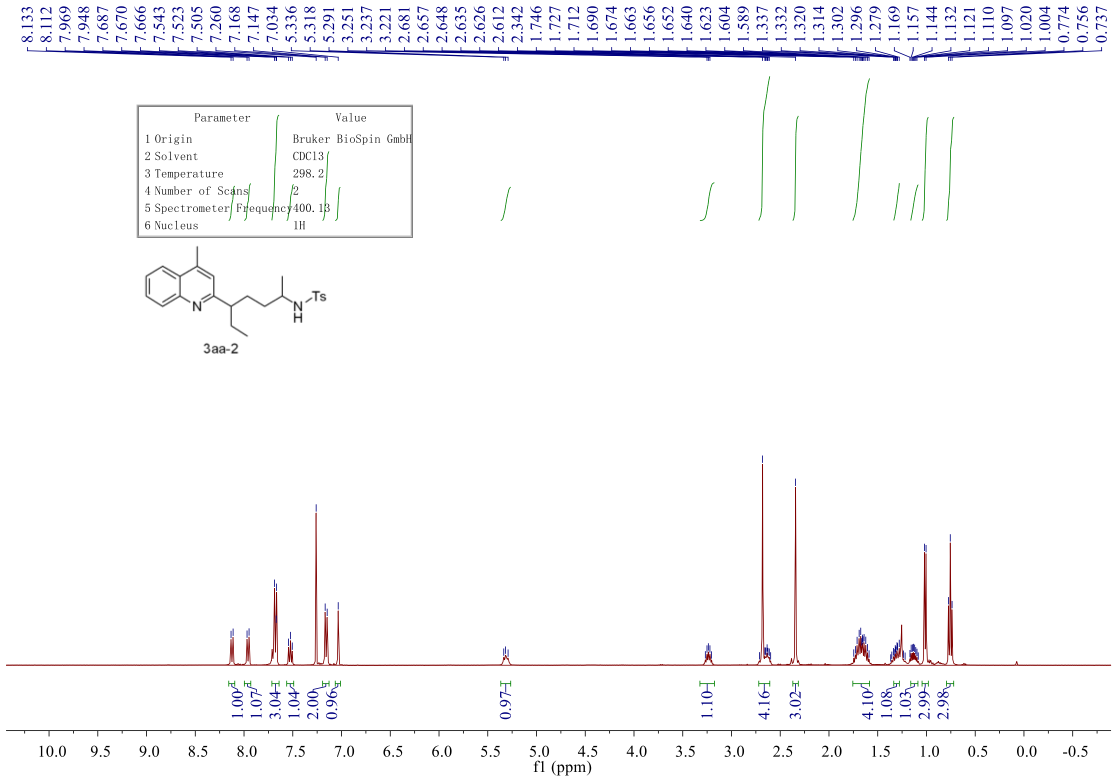
8.133
8.112
7.969
7.948
7.687
7.670
7.666
7.543
7.523
7.505
7.260
7.168
7.147
7.034
5.336
5.318
5.291
3.251
3.237
3.221
2.681
2.657
2.648
2.635
2.626
2.612
2.342
1.746
1.727
1.712
1.690
1.674
1.663
1.656
1.652
1.640
1.623
1.604
1.589
1.337
1.332
1.320
1.314
1.302
1.296
1.279
1.169
1.157
1.144
1.132
1.121
1.110
1.097
1.020
1.004
0.774
0.756
0.737
Parameter Value
1 Origin Bruker BioSpin GmbH
2 Solvent CDCl3
3 Temperature 298.2
4 Number of Scans 2
5 Spectrometer Frequency 400.13
6 Nucleus 1H
N
Ts
N
H
3aa-2
1.00
1.07
3.04
1.04
2.00
0.96
0.97
1.10
4.16
3.02
4.10
1.08
1.03
2.99
2.98
10.0 9.5 9.0 8.5 8.0 7.5 7.0 6.5 6.0 5.5 5.0 4.5 4.0 3.5 3.0 2.5 2.0 1.5 1.0 0.5 0.0 -0.5
f1 (ppm)

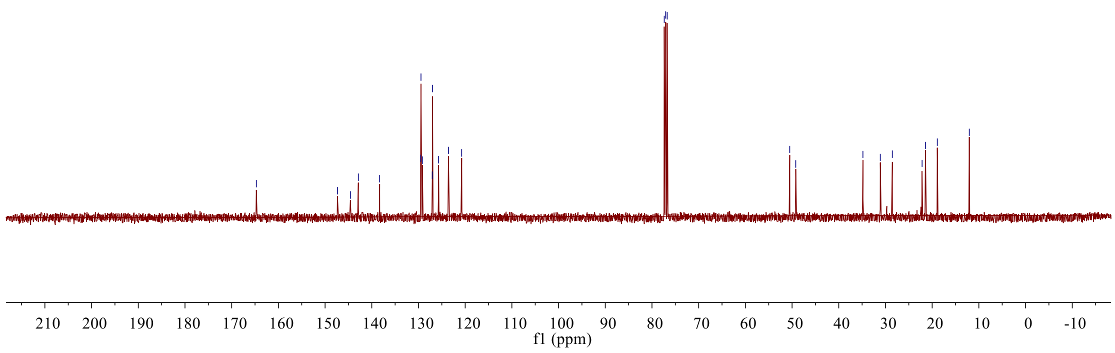

Parameter	Value
1 Origin	Bruker BioSpin GmbH
2 Solvent	CDCl3
3 Temperature	298.2
4 Number of Scans	82
5 Spectrometer Frequency	100.62
6 Nucleus	13C

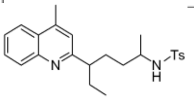

3aa-2

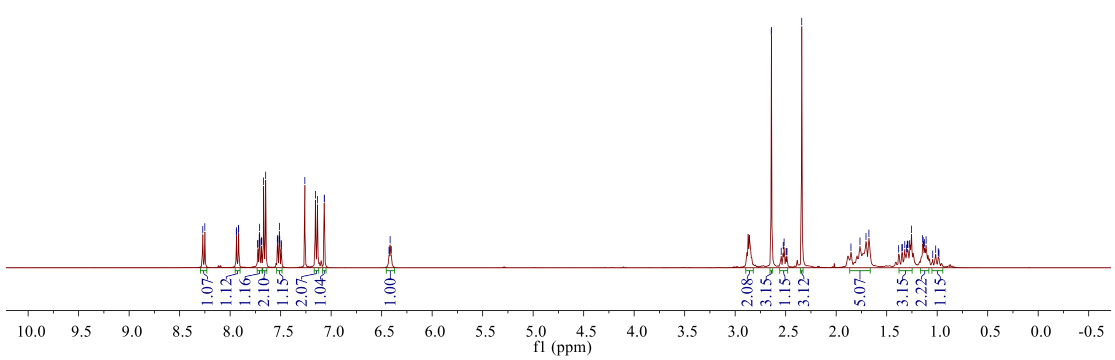

Parameter	Value
1 Origin	Bruker BioSpin GmbH
2 Solvent	CDCl3
3 Temperature	298.1
4 Number of Scans	2
5 Spectrometer Frequency	400.13
6 Nucleus	1H

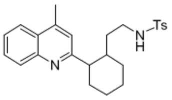

3ab

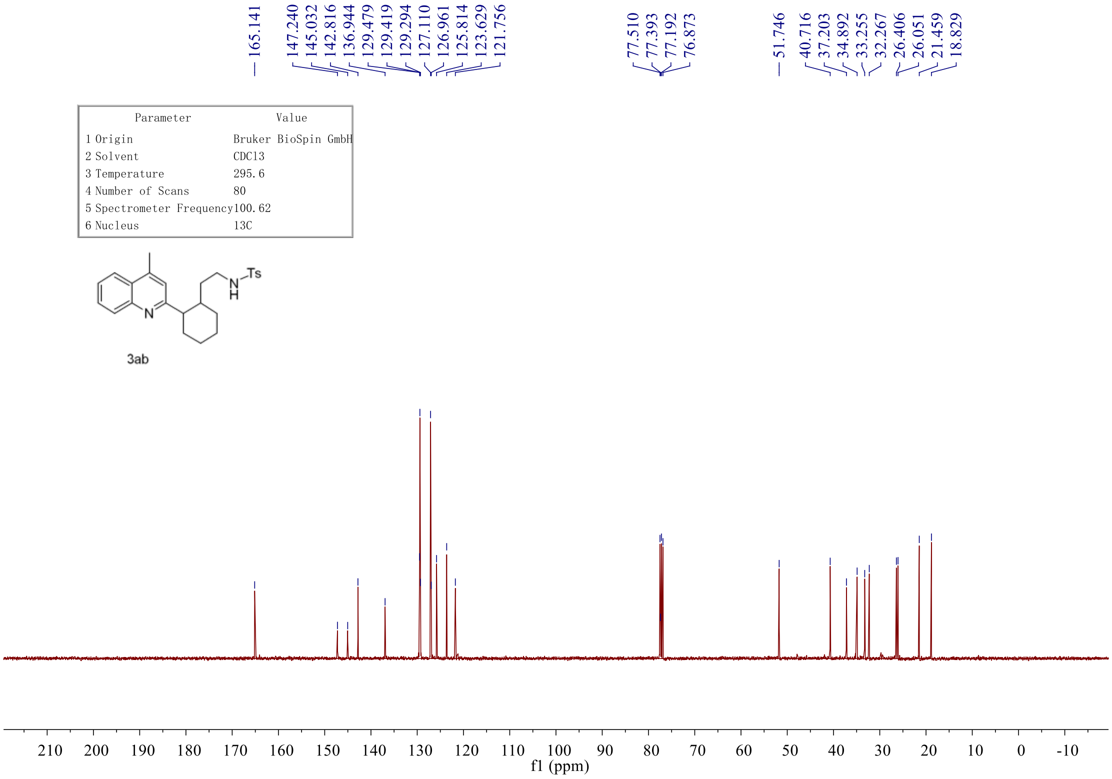
165.141
147.240
145.032
142.816
136.944
129.479
129.419
129.294
127.110
126.961
125.814
123.629
121.756
77.510
77.393
77.192
76.873
51.746
40.716
37.203
34.892
33.255
32.267
26.406
26.051
21.459
18.829
Parameter Value
1 Origin Bruker BioSpin GmbH
2 Solvent CDCl3
3 Temperature 295.6
4 Number of Scans 80
5 Spectrometer Frequency 100.62
6 Nucleus 13C
Ts
N
H
N
3ab
210 200 190 180 170 160 150 140 130 120 110 100 90 80 70 60 50 40 30 20 10 0 -10
f1 (ppm)

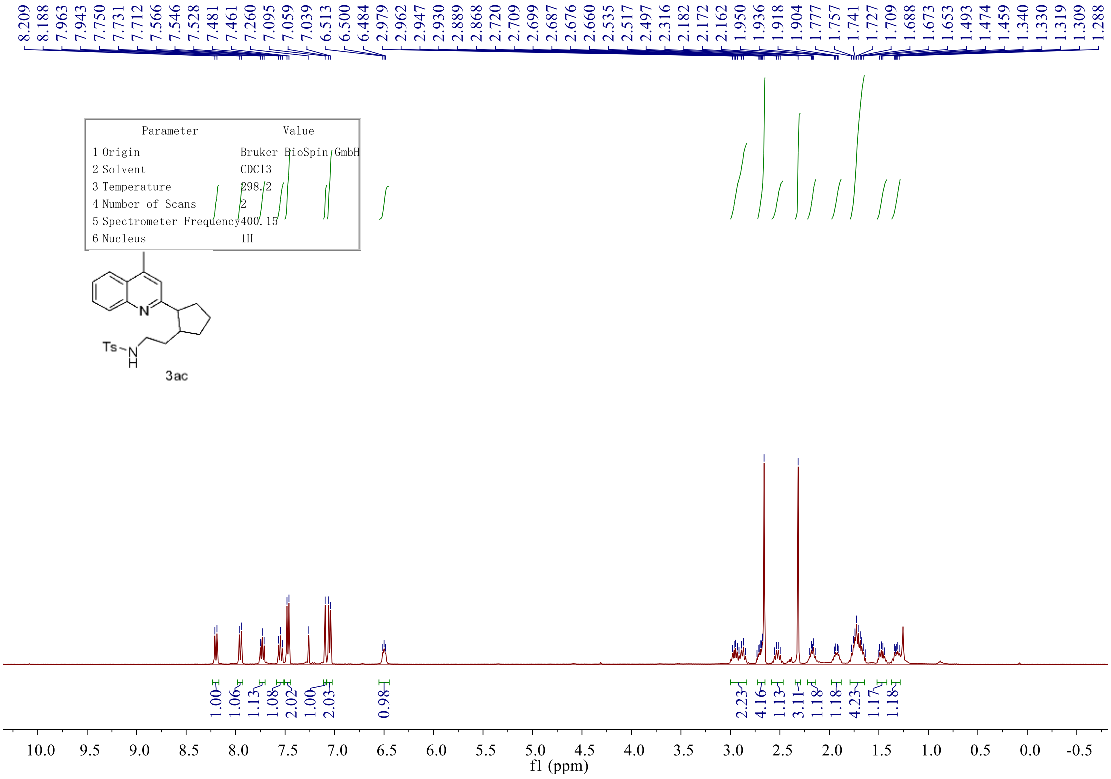
8.209
8.188
7.963
7.943
7.750
7.731
7.712
7.566
7.546
7.528
7.481
7.461
7.260
7.095
7.059
7.039
6.513
6.500
6.484
2.979
2.962
2.947
2.930
2.889
2.868
2.720
2.709
2.699
2.687
2.676
2.660
2.535
2.517
2.497
2.316
2.182
2.172
2.162
1.950
1.936
1.918
1.904
1.777
1.757
1.741
1.727
1.709
1.688
1.673
1.653
1.493
1.474
1.459
1.340
1.330
1.319
1.309
1.288
Parameter Value
1 Origin Bruker BioSpin GmbH
2 Solvent CDCl3
3 Temperature 298.2
4 Number of Scans 2
5 Spectrometer Frequency 400.15
6 Nucleus 1H
N
Ts
N
H
3ac
1.00
1.06
1.13
1.08
2.02
1.00
2.03
0.98
2.23
4.16
1.13
3.11
1.18
1.18
4.23
1.17
1.18
10.0 9.5 9.0 8.5 8.0 7.5 7.0 6.5 6.0 5.5 5.0 4.5 4.0 3.5 3.0 2.5 2.0 1.5 1.0 0.5 0.0 -0.5
f1 (ppm)

Parameter	Value
1 Origin	Bruker BioSpin GmbH
2 Solvent	CDCl3
3 Temperature	298.1
4 Number of Scans	80
5 Spectrometer Frequency	100.62
6 Nucleus	13C

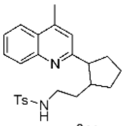

3ac

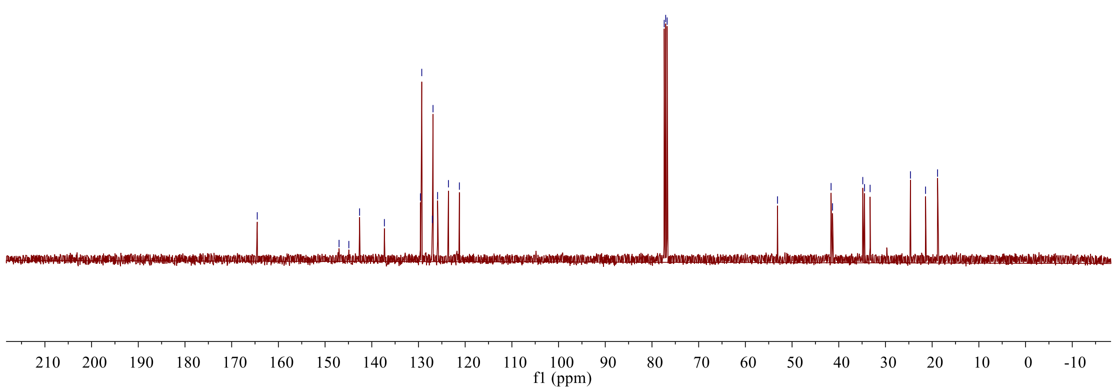

Parameter	Value
1 Origin	Bruker BioSpin GmbH
2 Solvent	CDCl3
3 Temperature	298.2
4 Number of Scans	2
5 Spectrometer Frequency	400.13
6 Nucleus	1H

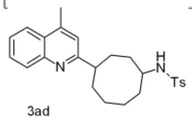

3ad

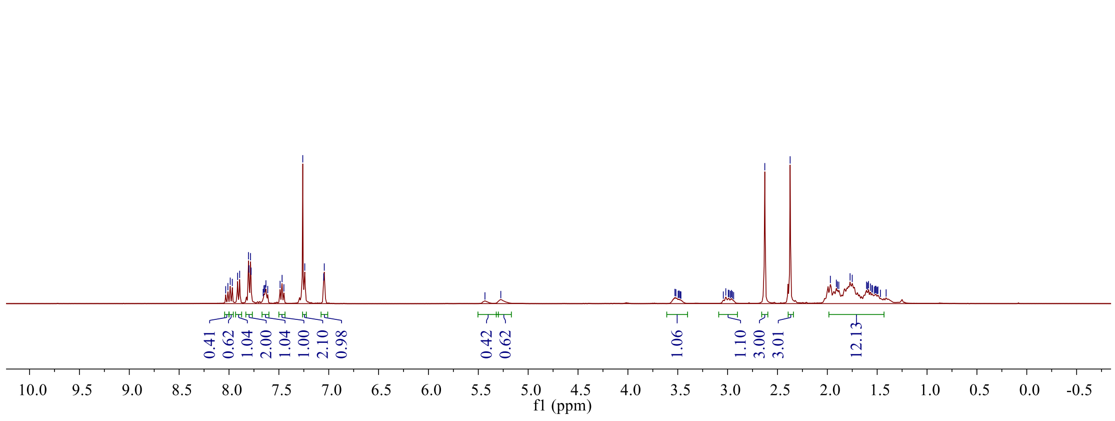

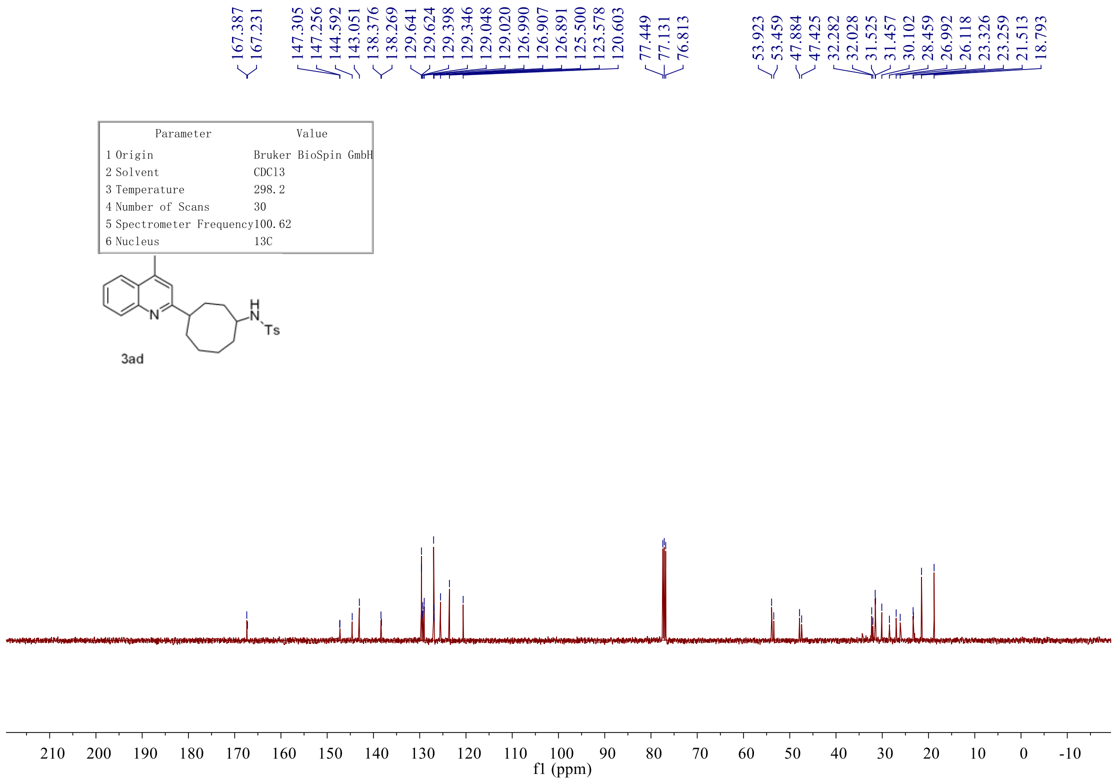
167.387
167.231
147.305
147.256
144.592
143.051
138.376
138.269
129.641
129.624
129.398
129.346
129.048
129.020
126.990
126.907
126.891
125.500
123.578
120.603
77.449
77.131
76.813
53.923
53.459
47.884
47.425
32.282
32.028
31.525
31.457
30.102
28.459
26.992
26.118
23.326
23.259
21.513
18.793
Parameter Value
1 Origin Bruker BioSpin GmbH
2 Solvent CDCl3
3 Temperature 298.2
4 Number of Scans 30
5 Spectrometer Frequency 100.62
6 Nucleus 13C
N
H
N
Ts
3ad
f1 (ppm)

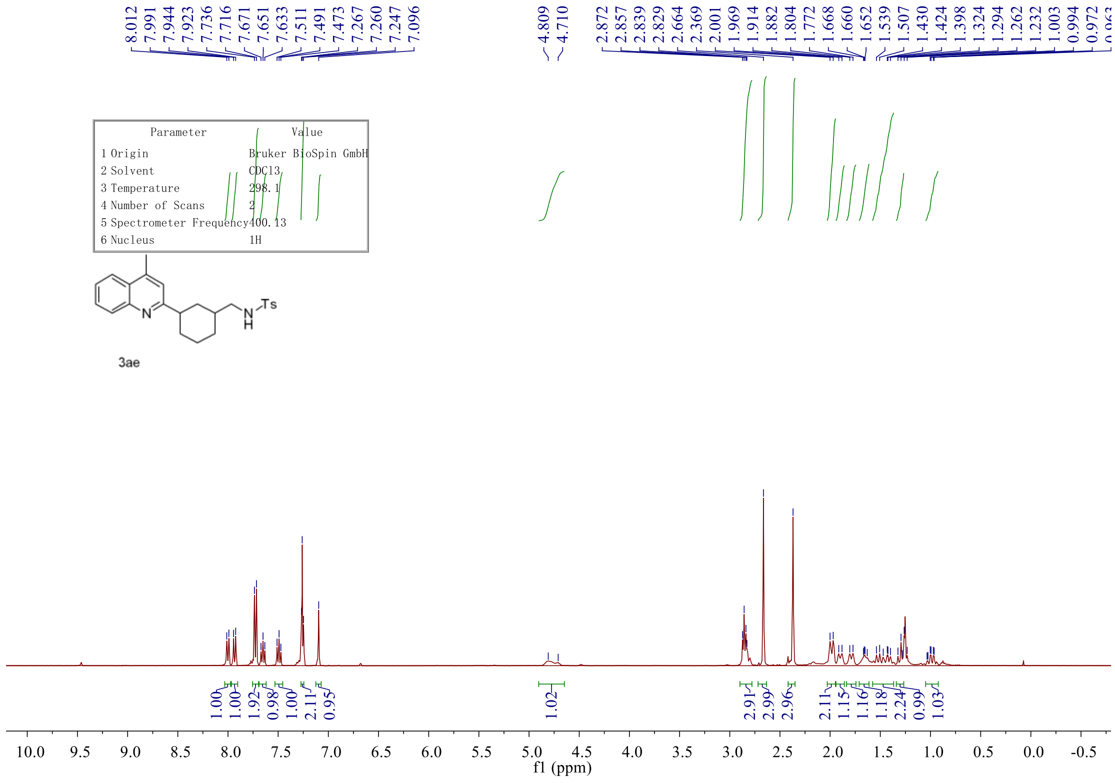
8.012
7.991
7.944
7.923
7.736
7.716
7.671
7.651
7.633
7.511
7.491
7.473
7.267
7.260
7.247
7.096
4.809
4.710
2.872
2.857
2.839
2.829
2.664
2.369
2.001
1.969
1.914
1.882
1.804
1.772
1.668
1.660
1.652
1.539
1.507
1.430
1.424
1.398
1.324
1.294
1.262
1.232
1.003
0.994
0.972
Parameter Value
1 Origin Bruker BioSpin GmbH
2 Solvent CDCl3
3 Temperature 298.1
4 Number of Scans 2
5 Spectrometer Frequency 400.13
6 Nucleus 1H
N
N
H
Ts
3ae
1.00
1.00
1.92
0.98
1.00
2.11
0.95
1.02
2.91
2.99
2.96
2.11
1.15
1.16
1.18
2.24
0.99
1.03
f1 (ppm)

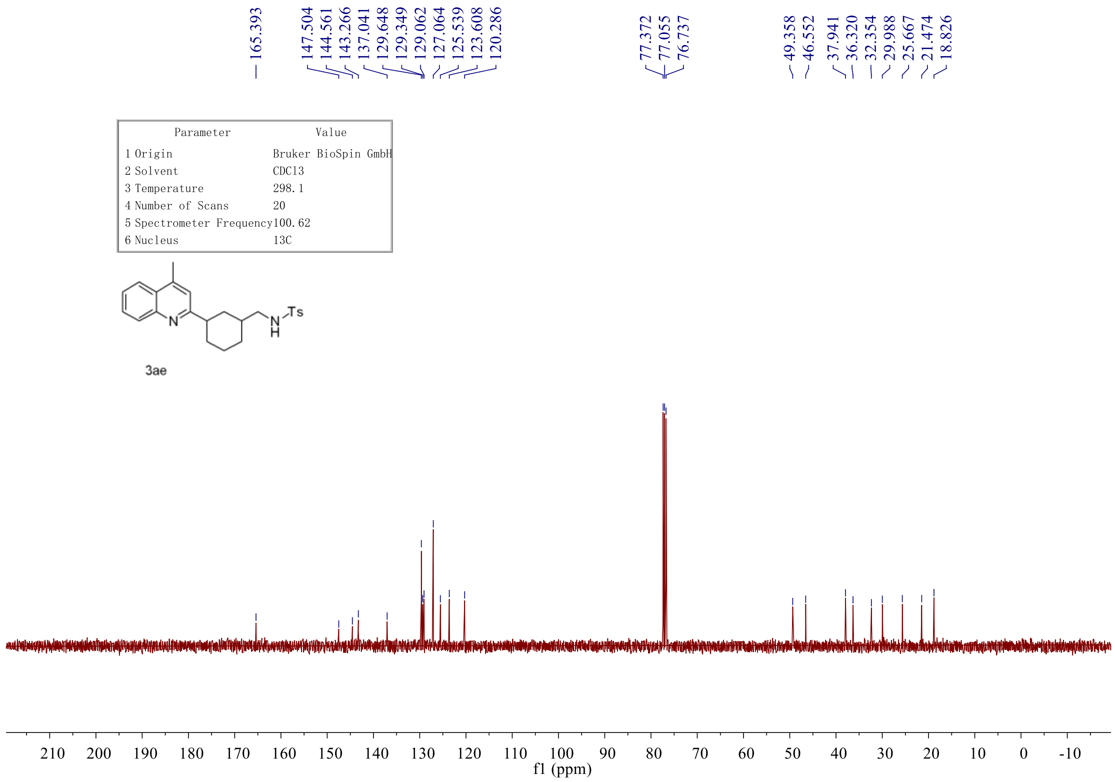

Parameter	Value
1 Origin	Bruker BioSpin GmbH
2 Solvent	CDCl3
3 Temperature	298.1
4 Number of Scans	20
5 Spectrometer Frequency	100.62
6 Nucleus	13C

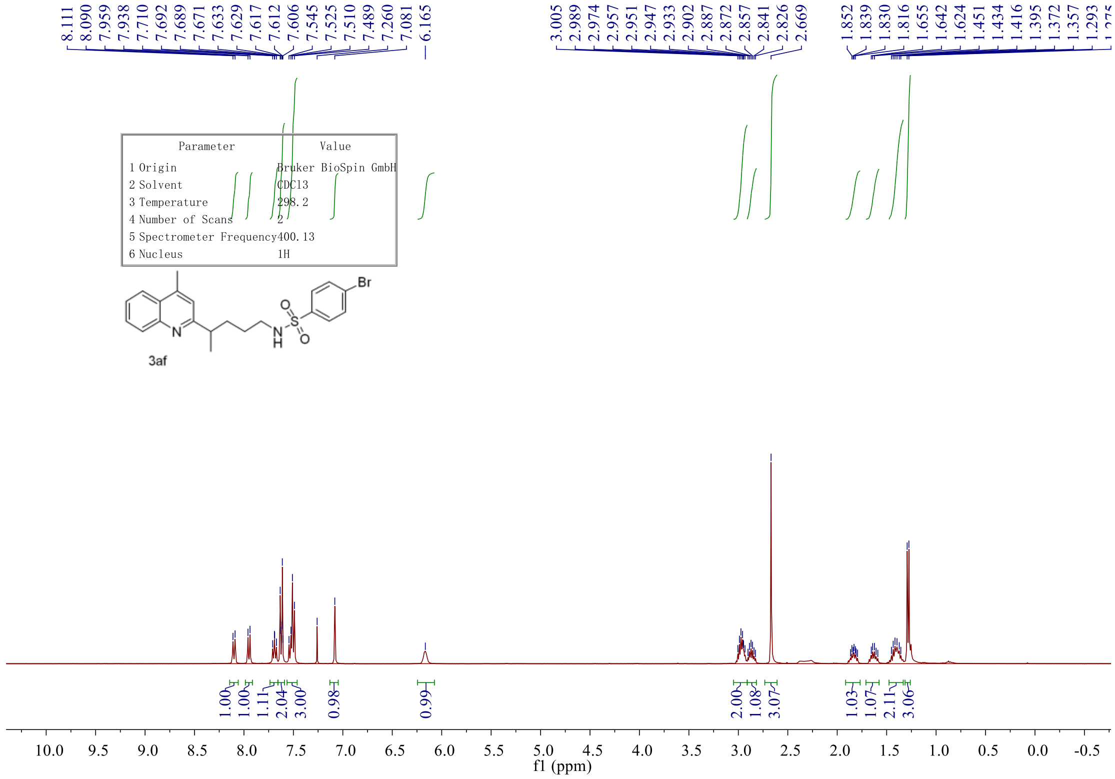

Parameter	Value
1 Origin	Bruker BioSpin GmbH
2 Solvent	CDCl3
3 Temperature	298.2
4 Number of Scans	2
5 Spectrometer Frequency	400.13
6 Nucleus	1H

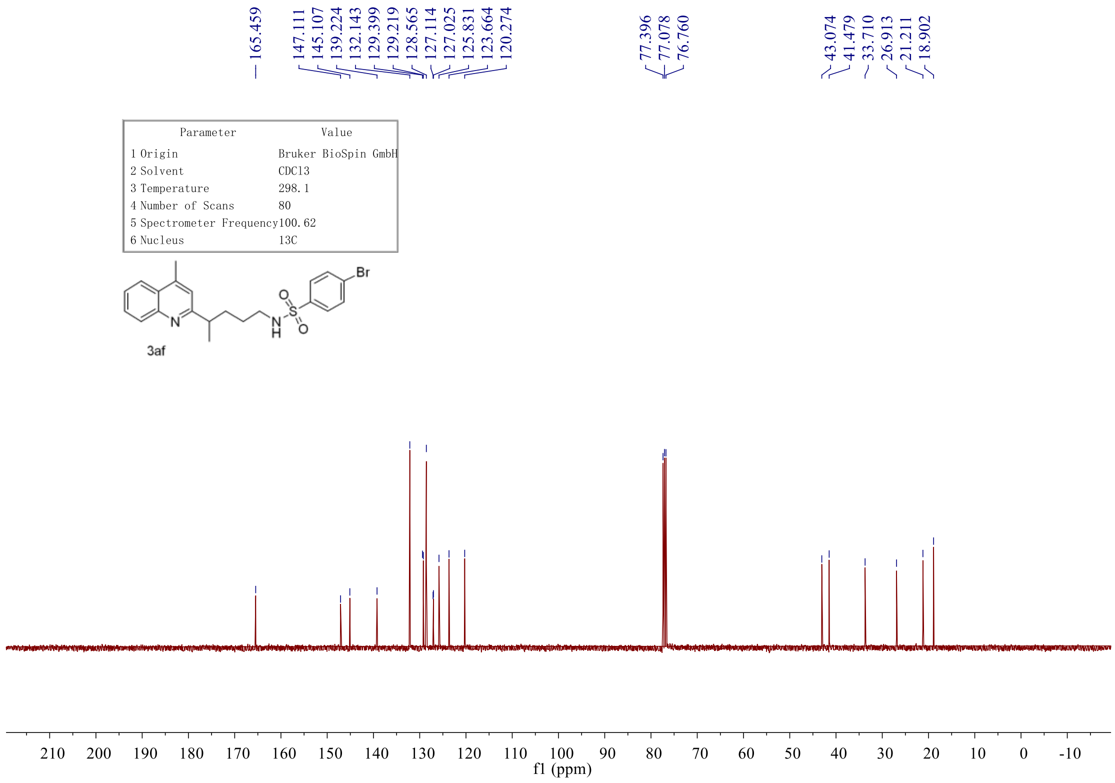
165.459
147.111 145.107 139.224 132.143 129.399 129.219 128.565 127.114 127.025 125.831 123.664 120.274
77.396 77.078 76.760
43.074 41.479 33.710 26.913 21.211 18.902
Parameter Value
1 Origin Bruker BioSpin GmbH
2 Solvent CDCl3
3 Temperature 298.1
4 Number of Scans 80
5 Spectrometer Frequency 100.62
6 Nucleus 13C
Br
O
S
O
N
H
N
3af
210 200 190 180 170 160 150 140 130 120 110 100 90 80 70 60 50 40 30 20 10 0 -10
f1 (ppm)

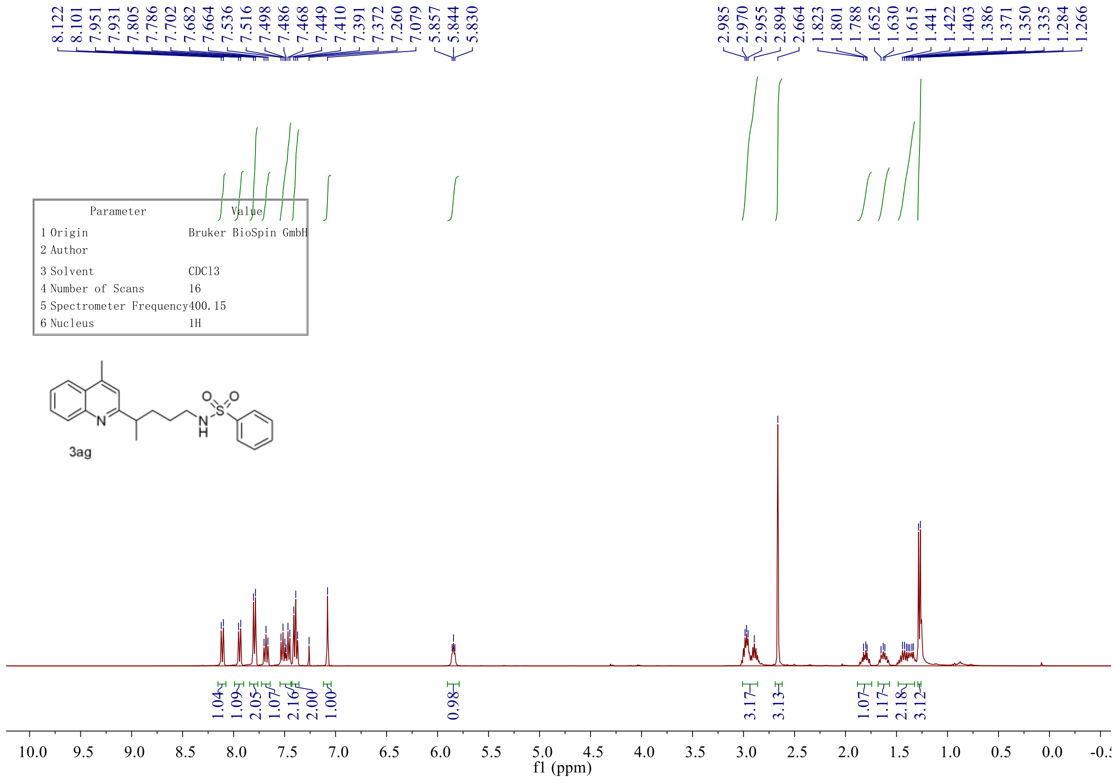
8.122 8.101 7.951 7.931 7.805 7.786 7.702 7.682 7.664 7.536 7.516 7.498 7.486 7.468 7.449 7.410 7.391 7.372 7.260 7.079 5.857 5.844 5.830
2.985 2.970 2.955 2.894 2.664 1.823 1.801 1.788 1.652 1.630 1.615 1.441 1.422 1.403 1.386 1.371 1.350 1.335 1.284 1.266
Parameter Value
1 Origin Bruker BioSpin GmbH
2 Author
3 Solvent CDCl3
4 Number of Scans 16
5 Spectrometer Frequency 400.15
6 Nucleus 1H
O
S
O
N
H
N
3ag
1.04 1.09 2.05 1.07 2.16 2.00 1.00 0.98 3.17 3.13 1.07 1.17 2.18 3.12
10.0 9.5 9.0 8.5 8.0 7.5 7.0 6.5 6.0 5.5 5.0 4.5 4.0 3.5 3.0 2.5 2.0 1.5 1.0 0.5 0.0
f1 (ppm)

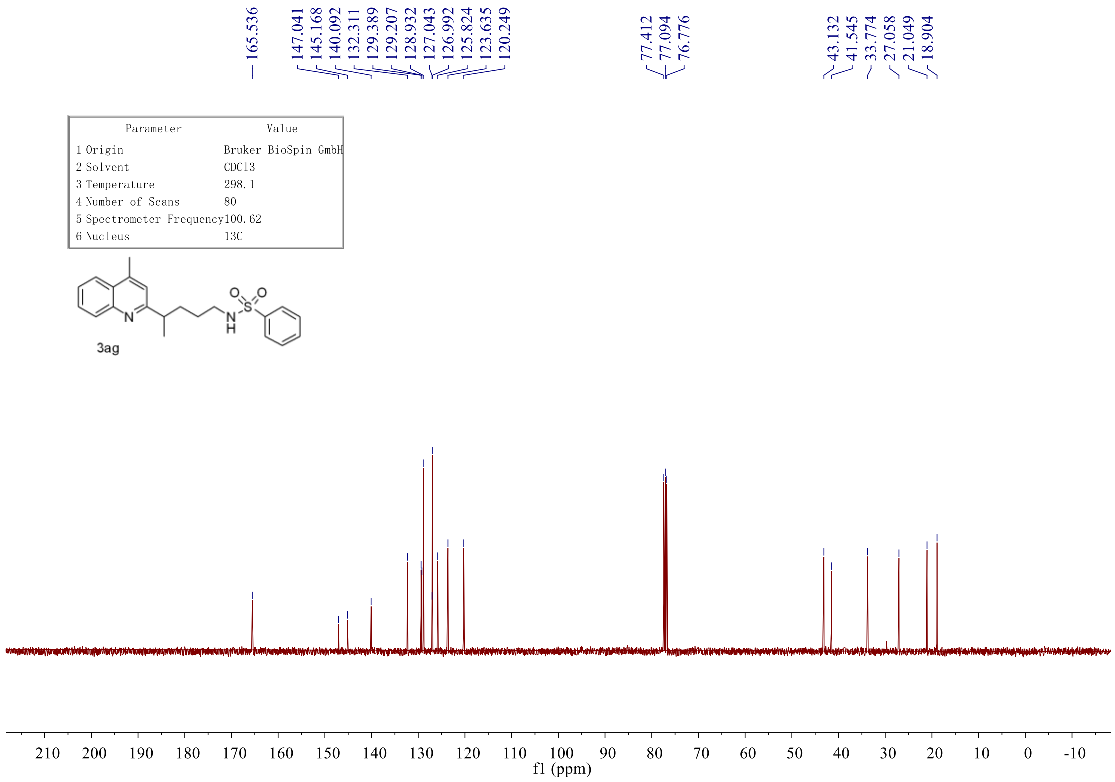
165.536
147.041 145.168 140.092 132.311 129.389 129.207 128.932 127.043 126.992 125.824 123.635 120.249
77.412 77.094 76.776
43.132 41.545 33.774 27.058 21.049 18.904
Parameter Value
1 Origin Bruker BioSpin GmbH
2 Solvent CDCl3
3 Temperature 298.1
4 Number of Scans 80
5 Spectrometer Frequency 100.62
6 Nucleus 13C
3ag
210 200 190 180 170 160 150 140 130 120 110 100 90 80 70 60 50 40 30 20 10 0 -10
f1 (ppm)

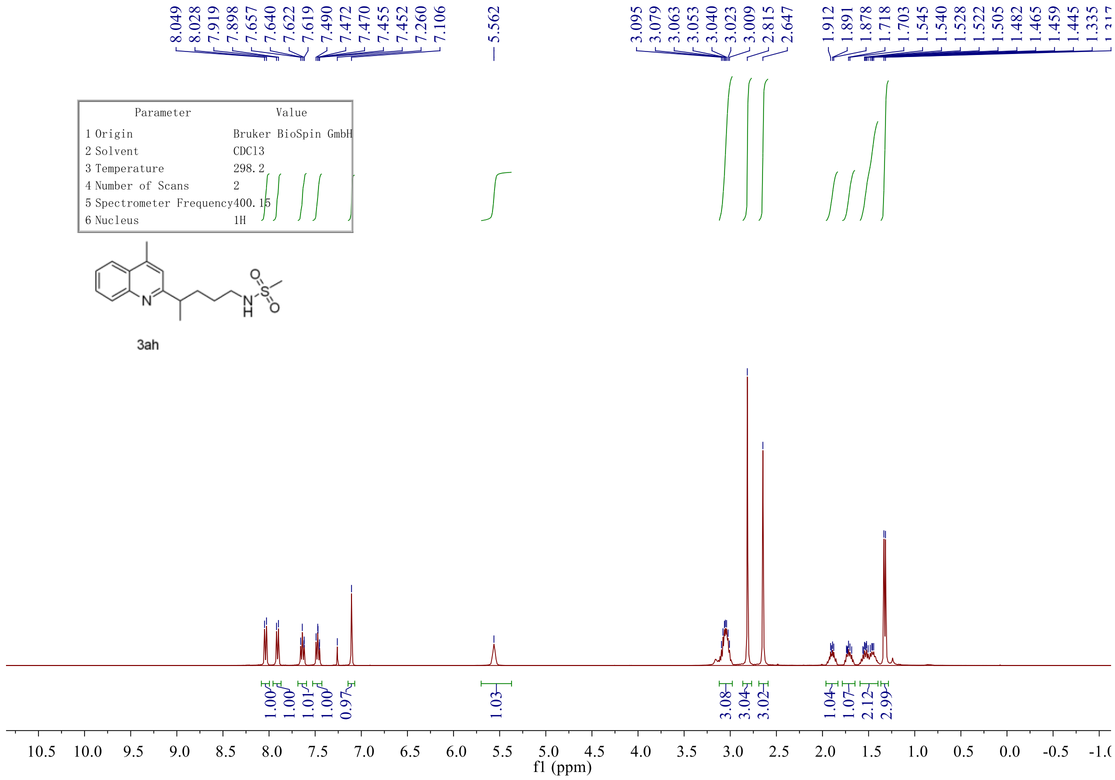
8.049 8.028 7.919 7.898 7.657 7.640 7.622 7.619 7.490 7.472 7.470 7.455 7.452 7.260 7.106
5.562
3.095 3.079 3.063 3.053 3.040 3.023 3.009 2.815 2.647
1.912 1.891 1.878 1.718 1.703 1.545 1.540 1.528 1.522 1.505 1.482 1.465 1.459 1.445 1.335
Parameter Value
1 Origin Bruker BioSpin GmbH
2 Solvent CDCl3
3 Temperature 298.2
4 Number of Scans 2
5 Spectrometer Frequency 400.15
6 Nucleus 1H
3ah
1.00 1.00 1.01 1.00 0.97 1.03 3.08 3.04 3.02 1.04 1.07 2.12 2.99
10.5 10.0 9.5 9.0 8.5 8.0 7.5 7.0 6.5 6.0 5.5 5.0 4.5 4.0 3.5 3.0 2.5 2.0 1.5 1.0 0.5 0.0 -0.5
f1 (ppm)

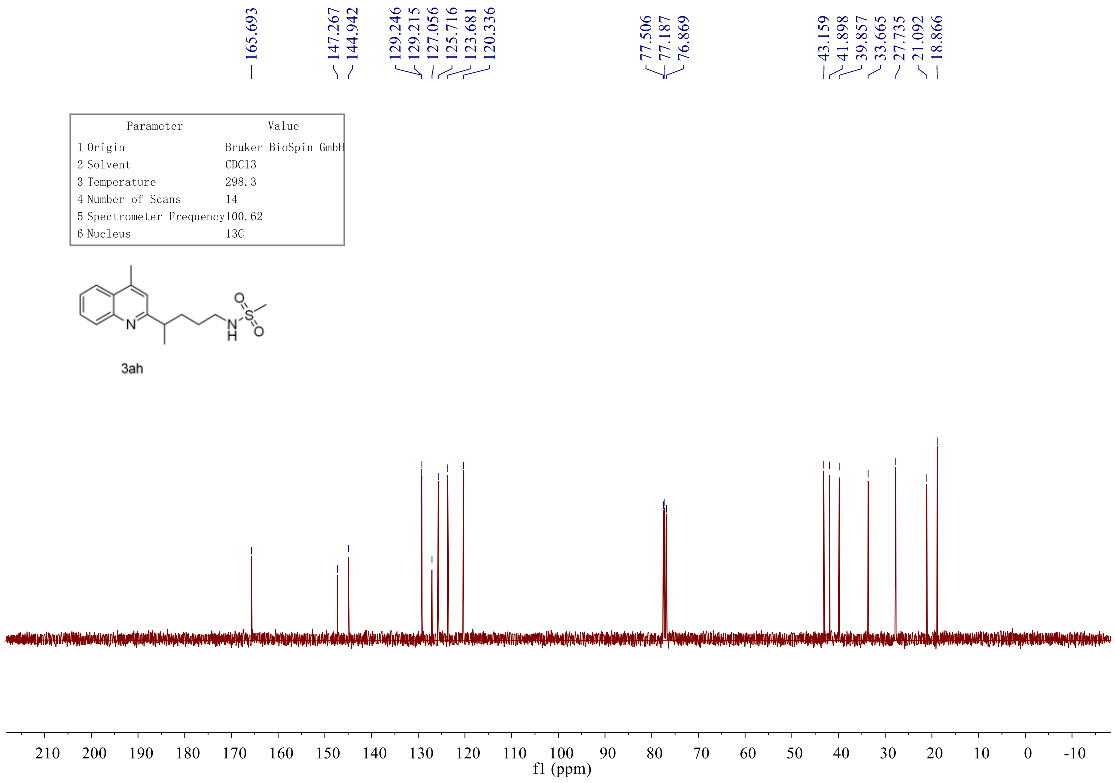
165.693
147.267
144.942
129.246
129.215
127.056
125.716
123.681
120.336
77.506
77.187
76.869
43.159
41.898
39.857
33.665
27.735
21.092
18.866
Parameter Value
1 Origin Bruker BioSpin GmbH
2 Solvent CDCl3
3 Temperature 298.3
4 Number of Scans 14
5 Spectrometer Frequency 100.62
6 Nucleus 13C
3ah
210 200 190 180 170 160 150 140 130 120 110 100 90 80 70 60 50 40 30 20 10 0 -10
f1 (ppm)

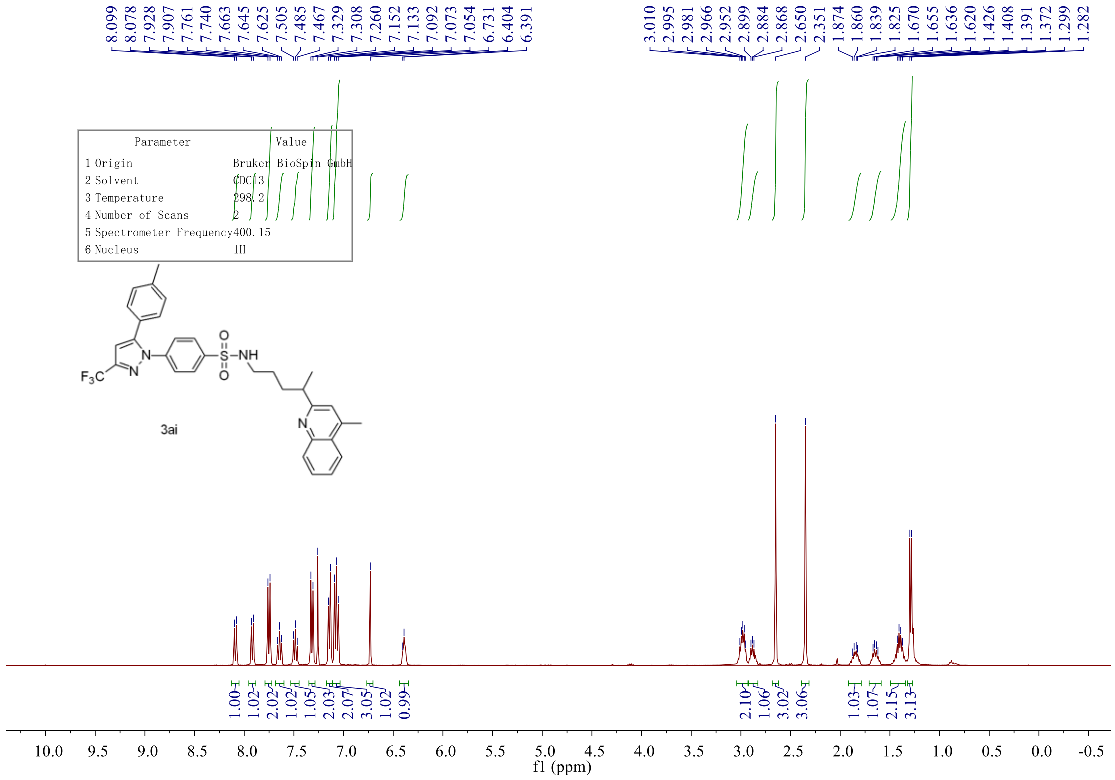
8.099
8.078
7.928
7.907
7.761
7.740
7.663
7.645
7.625
7.505
7.485
7.467
7.329
7.308
7.260
7.152
7.133
7.092
7.073
7.054
6.731
6.404
6.391
3.010
2.995
2.981
2.966
2.952
2.899
2.884
2.868
2.650
2.351
1.874
1.860
1.839
1.825
1.670
1.655
1.636
1.620
1.426
1.408
1.391
1.372
1.299
1.282
Parameter Value
1 Origin Bruker BioSpin GmbH
2 Solvent CDCl3
3 Temperature 298.2
4 Number of Scans 2
5 Spectrometer Frequency 400.15
6 Nucleus 1H
3ai
1.00
1.02
2.02
1.02
1.05
2.03
2.07
3.05
1.02
0.99
2.10
1.06
3.02
3.06
1.03
1.07
2.15
3.13
10.0 9.5 9.0 8.5 8.0 7.5 7.0 6.5 6.0 5.5 5.0 4.5 4.0 3.5 3.0 2.5 2.0 1.5 1.0 0.5 0.0 -0.5
f1 (ppm)

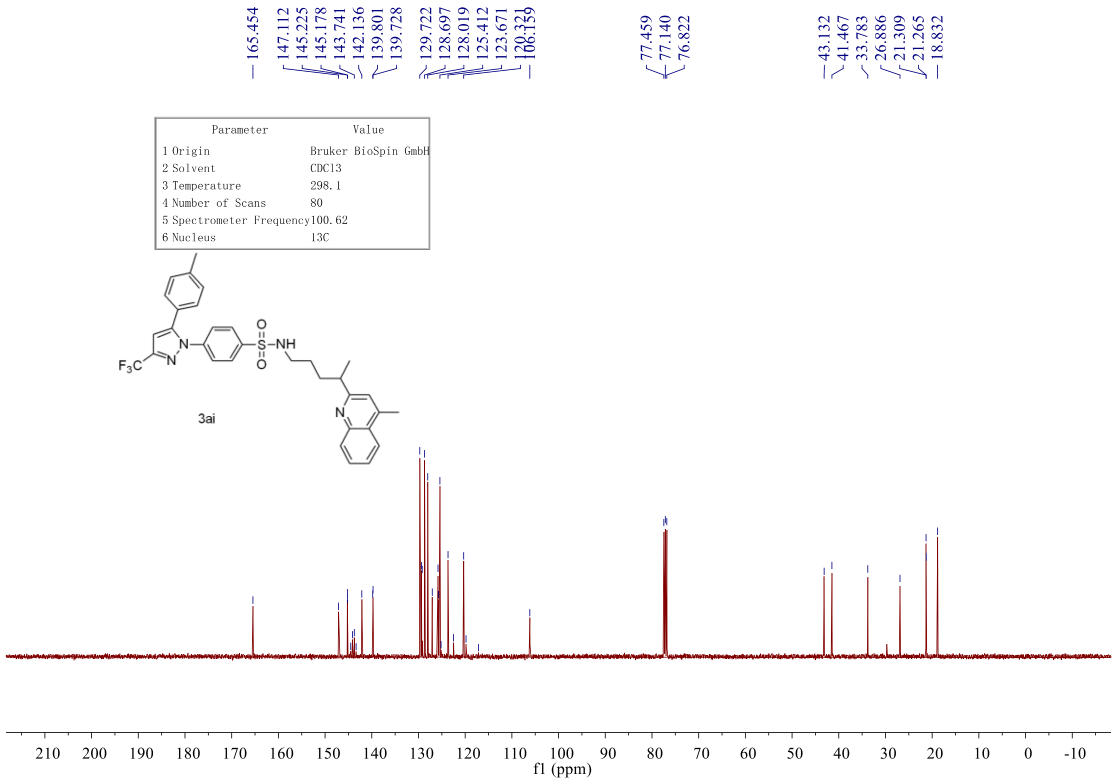
165.454
147.112
145.225
145.178
143.741
142.136
139.801
139.728
129.722
128.697
128.019
125.412
123.671
120.321
106.159
77.459
77.140
76.822
43.132
41.467
33.783
26.886
21.309
21.265
18.832
Parameter Value
1 Origin Bruker BioSpin GmbH
2 Solvent CDCl3
3 Temperature 298.1
4 Number of Scans 80
5 Spectrometer Frequency 100.62
6 Nucleus 13C
3ai
f1 (ppm)

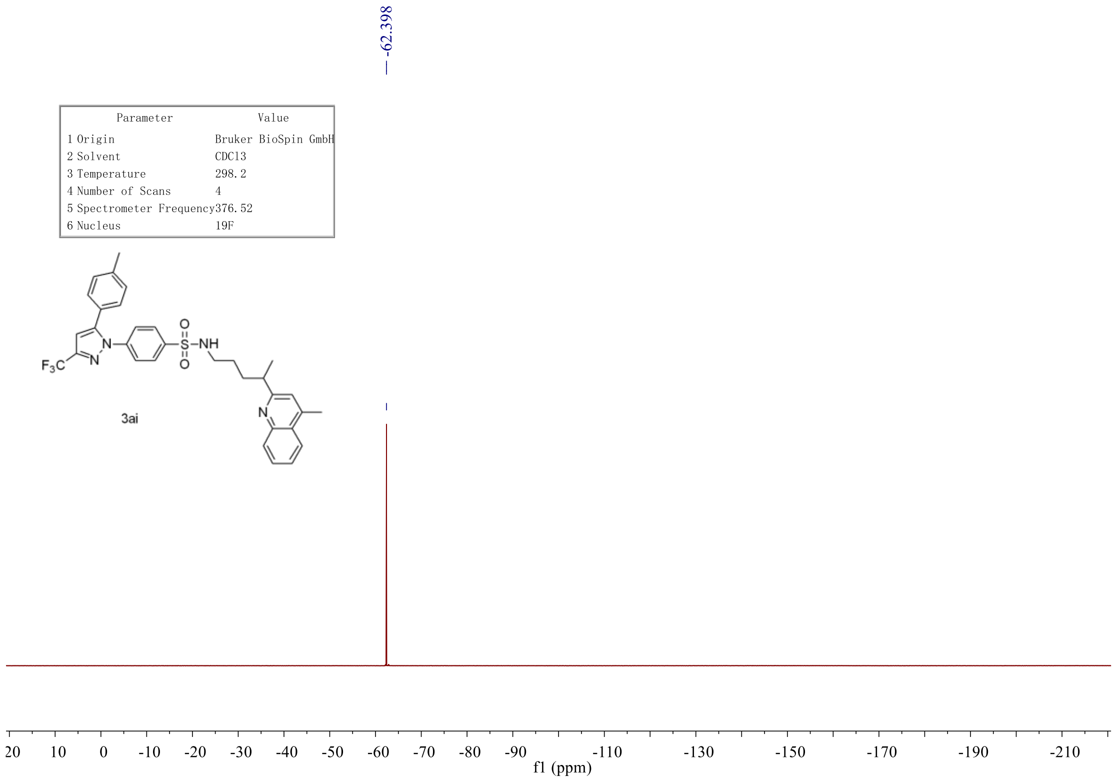
-62.398
Parameter Value
1 Origin Bruker BioSpin GmbH
2 Solvent CDCl3
3 Temperature 298.2
4 Number of Scans 4
5 Spectrometer Frequency 376.52
6 Nucleus 19F
3ai
f1 (ppm)

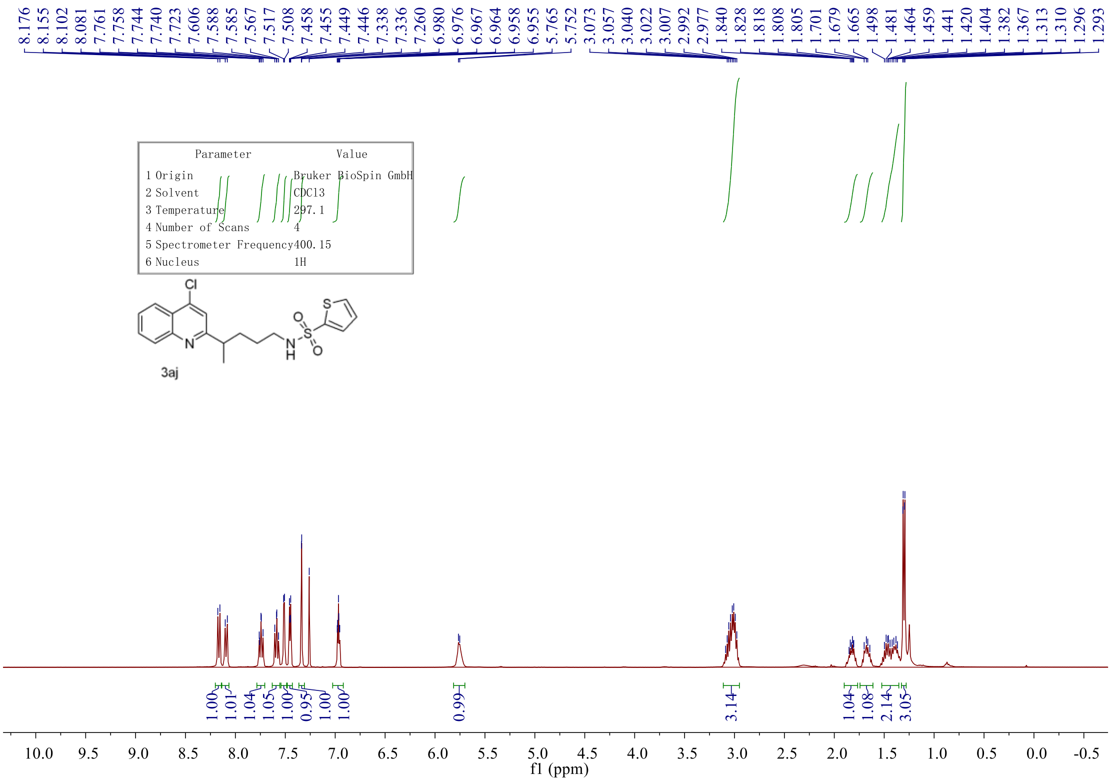

Parameter	Value
1 Origin	Bruker BioSpin GmbH
2 Solvent	CDCl3
3 Temperature	297.1
4 Number of Scans	4
5 Spectrometer Frequency	400.15
6 Nucleus	1H

3aj

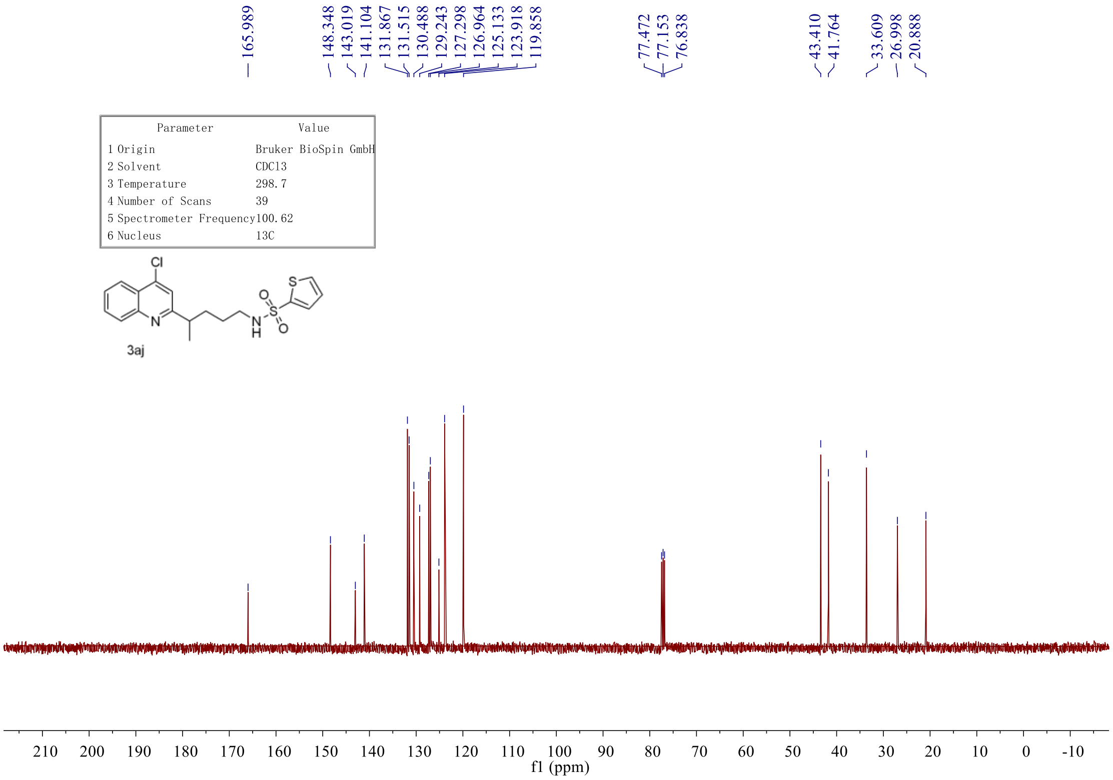

Parameter	Value
1 Origin	Bruker BioSpin GmbH
2 Solvent	CDCl3
3 Temperature	298.7
4 Number of Scans	39
5 Spectrometer Frequency	100.62
6 Nucleus	13C

3aj

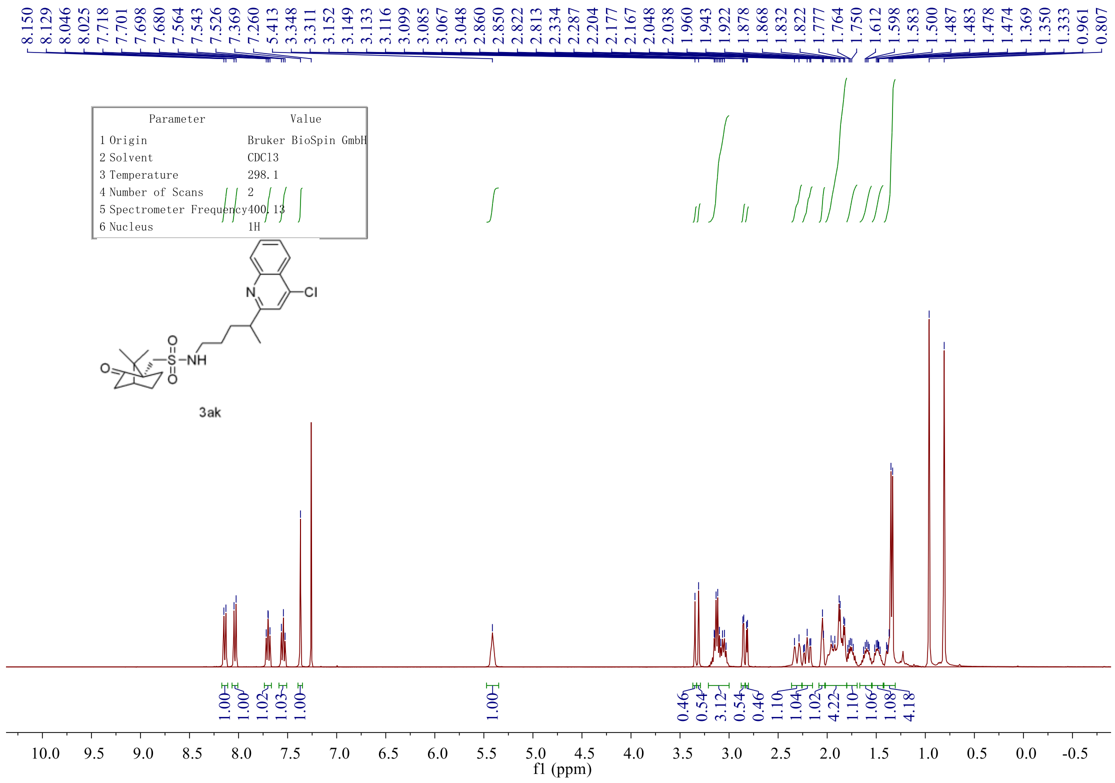
8.150
8.129
8.046
8.025
7.718
7.701
7.698
7.680
7.564
7.543
7.526
7.369
7.260
5.413
3.348
3.311
3.152
3.149
3.133
3.116
3.099
3.085
3.067
3.048
2.860
2.850
2.822
2.813
2.334
2.287
2.204
2.177
2.167
2.048
2.038
1.960
1.943
1.922
1.878
1.868
1.832
1.822
1.777
1.764
1.750
1.612
1.598
1.583
1.500
1.487
1.483
1.478
1.474
1.369
1.350
1.333
0.961
0.807
Parameter
Value
1 Origin
Bruker BioSpin GmbH
2 Solvent
CDCl3
3 Temperature
298.1
4 Number of Scans
2
5 Spectrometer Frequency
400.13
6 Nucleus
1H
N
Cl
O
S
NH
3ak
1.00
1.00
1.02
1.03
1.00
1.00
0.46
0.54
3.12
0.54
0.46
1.10
1.04
1.02
4.22
1.10
1.06
1.08
4.18
f1 (ppm)

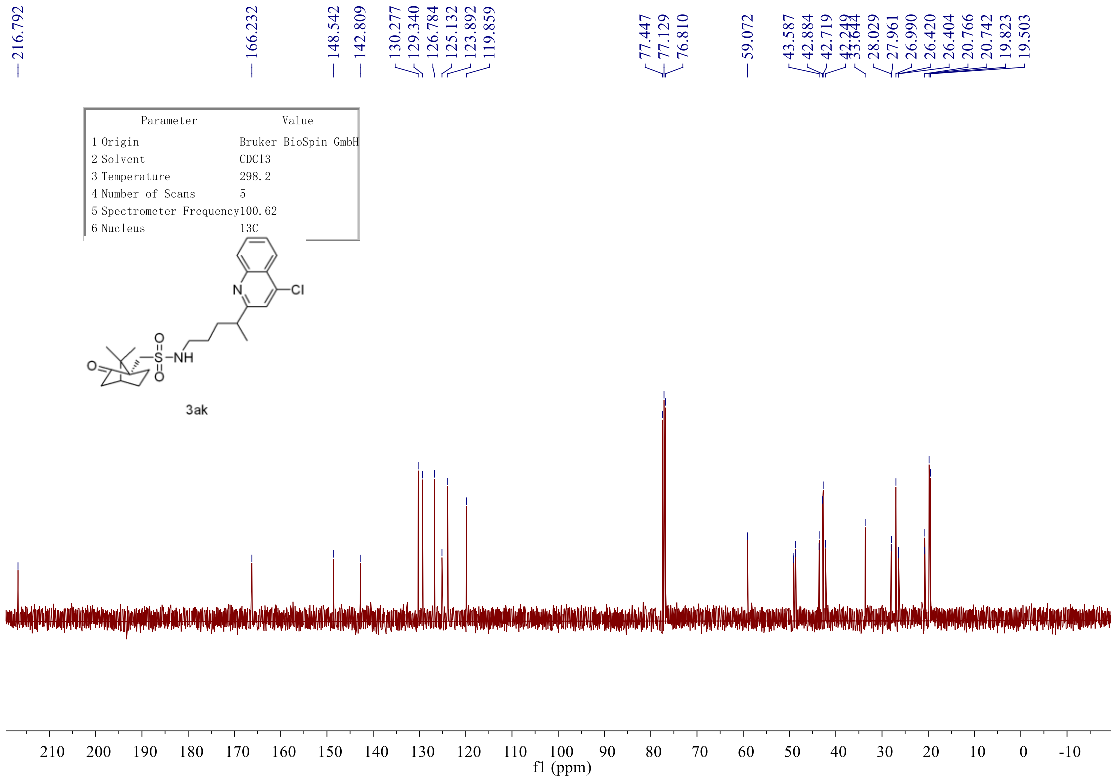
216.792
166.232
148.542
142.809
130.277
129.340
126.784
125.132
123.892
119.859
77.447
77.129
76.810
59.072
43.587
42.884
42.719
42.249
33.644
28.029
27.961
26.990
26.420
26.404
20.766
20.742
19.823
19.503
Parameter
Value
1 Origin
Bruker BioSpin GmbH
2 Solvent
CDCl3
3 Temperature
298.2
4 Number of Scans
5
5 Spectrometer Frequency
100.62
6 Nucleus
13C
N
Cl
O
S
NH
3ak
f1 (ppm)

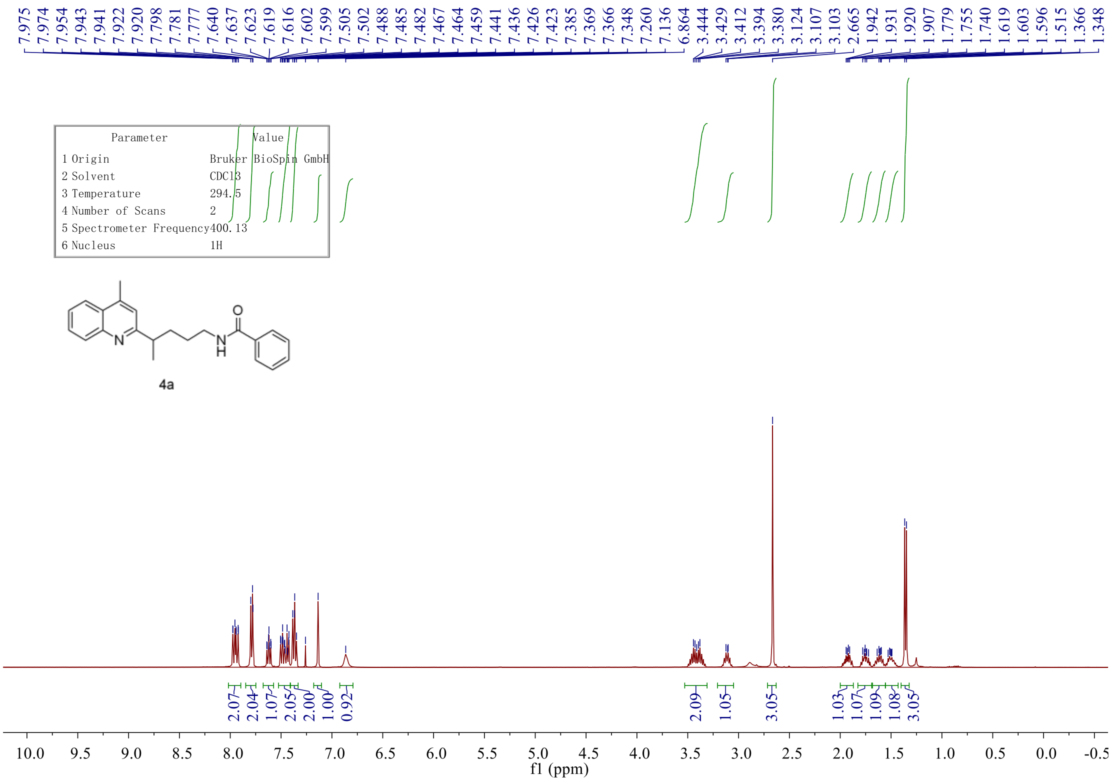

Parameter Value
1 Origin Bruker BioSpin GmbH
2 Solvent CDCl3
3 Temperature 294.5
4 Number of Scans 2
5 Spectrometer Frequency 400.13
6 Nucleus 1H
4a
f1 (ppm)

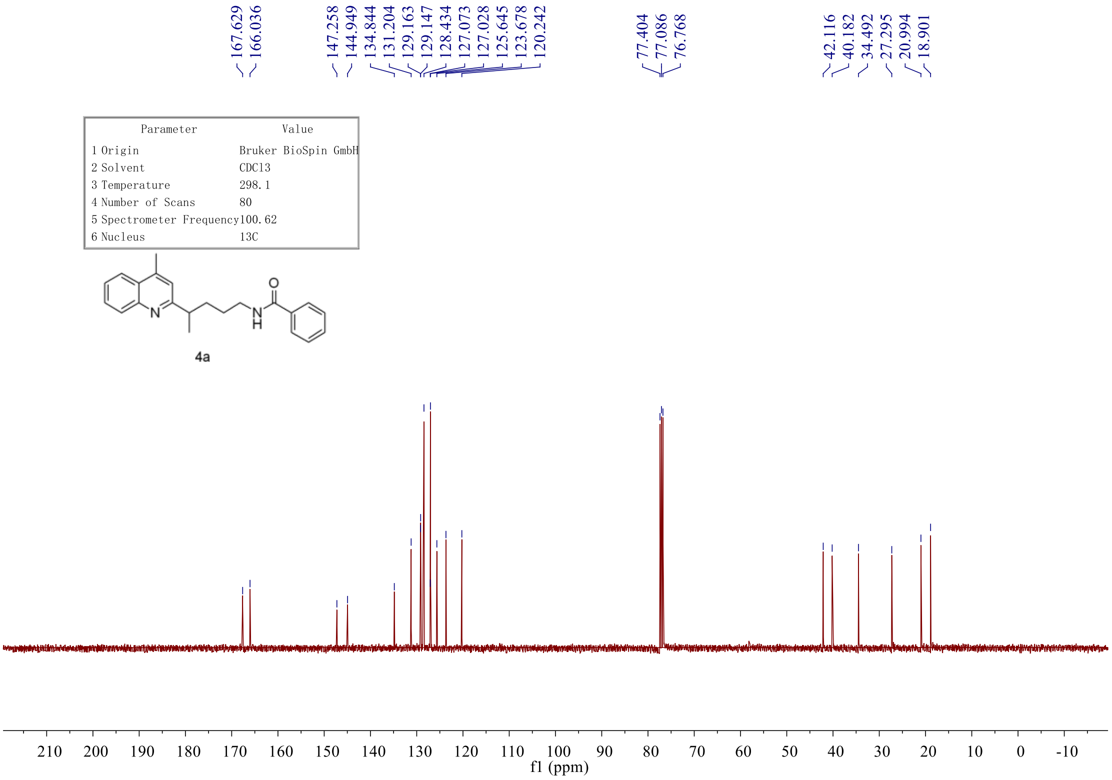

Parameter Value
1 Origin Bruker BioSpin GmbH
2 Solvent CDCl3
3 Temperature 298.1
4 Number of Scans 80
5 Spectrometer Frequency 100.62
6 Nucleus 13C
4a
f1 (ppm)

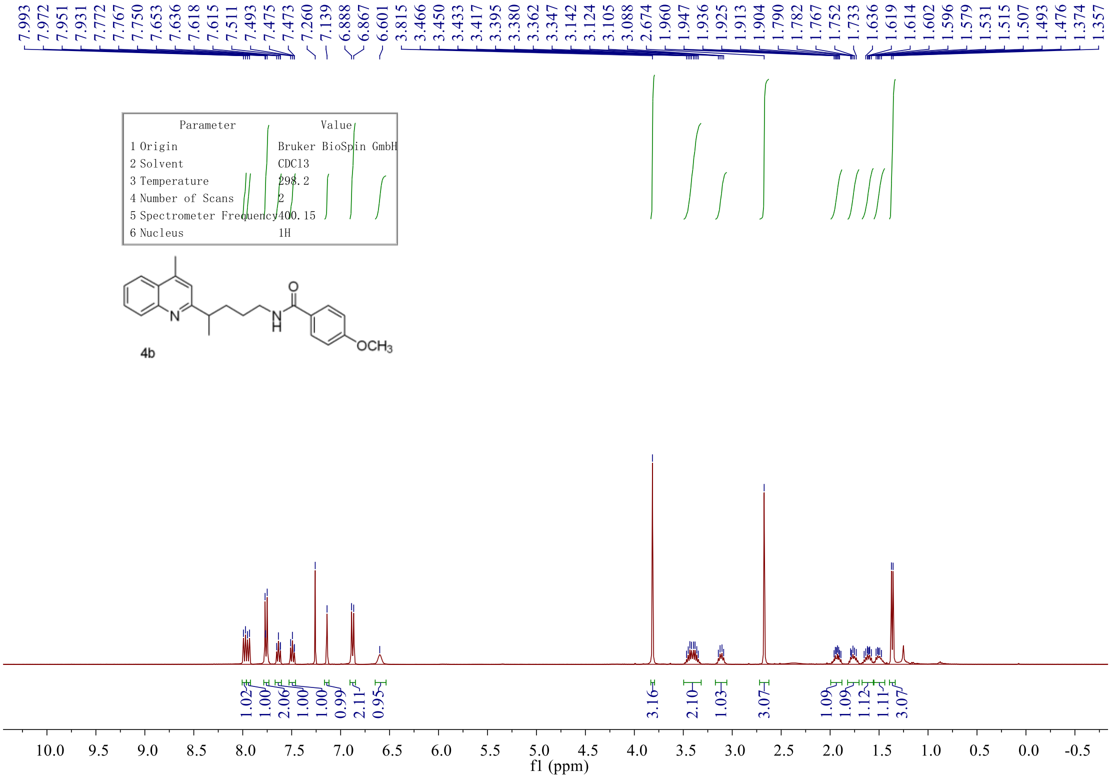
7.993
7.972
7.951
7.931
7.772
7.767
7.750
7.653
7.636
7.618
7.615
7.511
7.493
7.475
7.473
7.260
7.139
6.888
6.867
6.601
3.815
3.466
3.450
3.433
3.417
3.395
3.380
3.362
3.347
3.142
3.124
3.105
3.088
2.674
1.960
1.947
1.936
1.925
1.913
1.904
1.790
1.782
1.767
1.752
1.733
1.636
1.619
1.614
1.602
1.596
1.579
1.531
1.515
1.507
1.493
1.476
1.374
1.357
Parameter
Value
1 Origin
Bruker BioSpin GmbH
2 Solvent
CDCl3
3 Temperature
298.2
4 Number of Scans
2
5 Spectrometer Frequency
400.15
6 Nucleus
1H
N
H
O
OCH3
4b
1.02
1.00
2.06
1.00
1.00
0.99
2.11
0.95
3.16
2.10
1.03
3.07
1.09
1.09
1.12
1.11
3.07
10.0
9.5
9.0
8.5
8.0
7.5
7.0
6.5
6.0
5.5
5.0
4.5
4.0
3.5
3.0
2.5
2.0
1.5
1.0
0.5
0.0
-0.5
f1 (ppm)

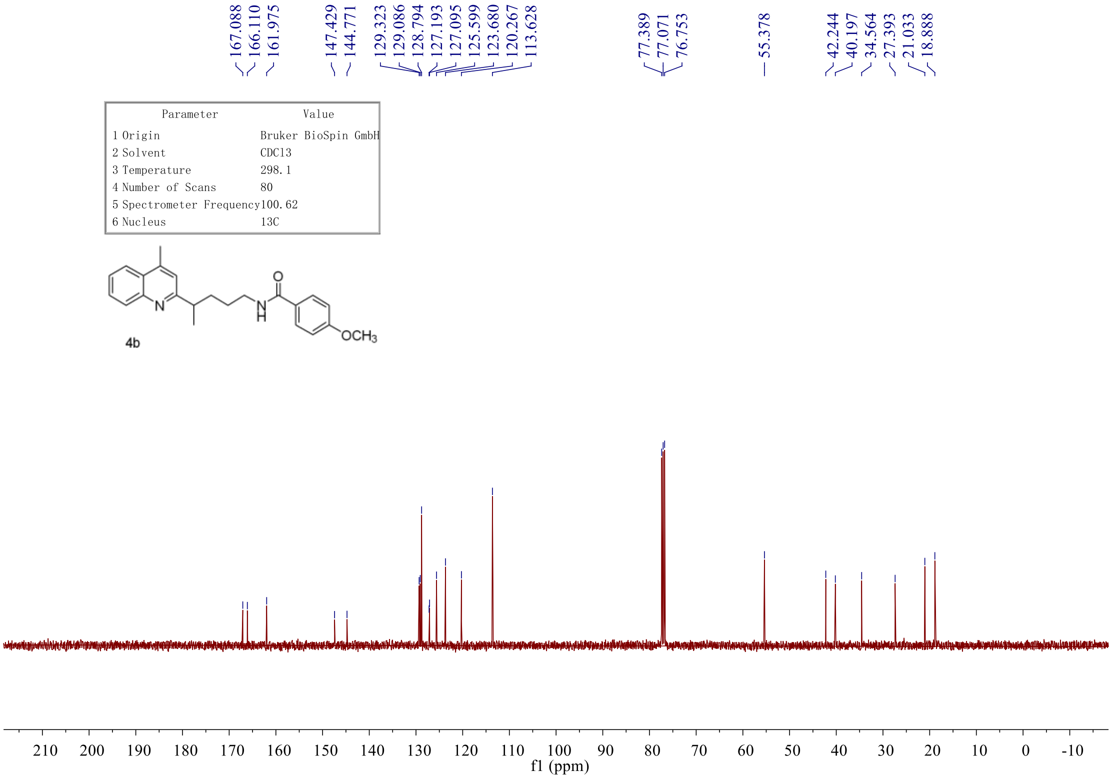
167.088
166.110
161.975
147.429
144.771
129.323
129.086
128.794
127.193
127.095
125.599
123.680
120.267
113.628
77.389
77.071
76.753
55.378
42.244
40.197
34.564
27.393
21.033
18.888
Parameter
Value
1 Origin
Bruker BioSpin GmbH
2 Solvent
CDCl3
3 Temperature
298.1
4 Number of Scans
80
5 Spectrometer Frequency
100.62
6 Nucleus
13C
N
H
O
OCH3
4b
210
200
190
180
170
160
150
140
130
120
110
100
90
80
70
60
50
40
30
20
10
0
-10
f1 (ppm)

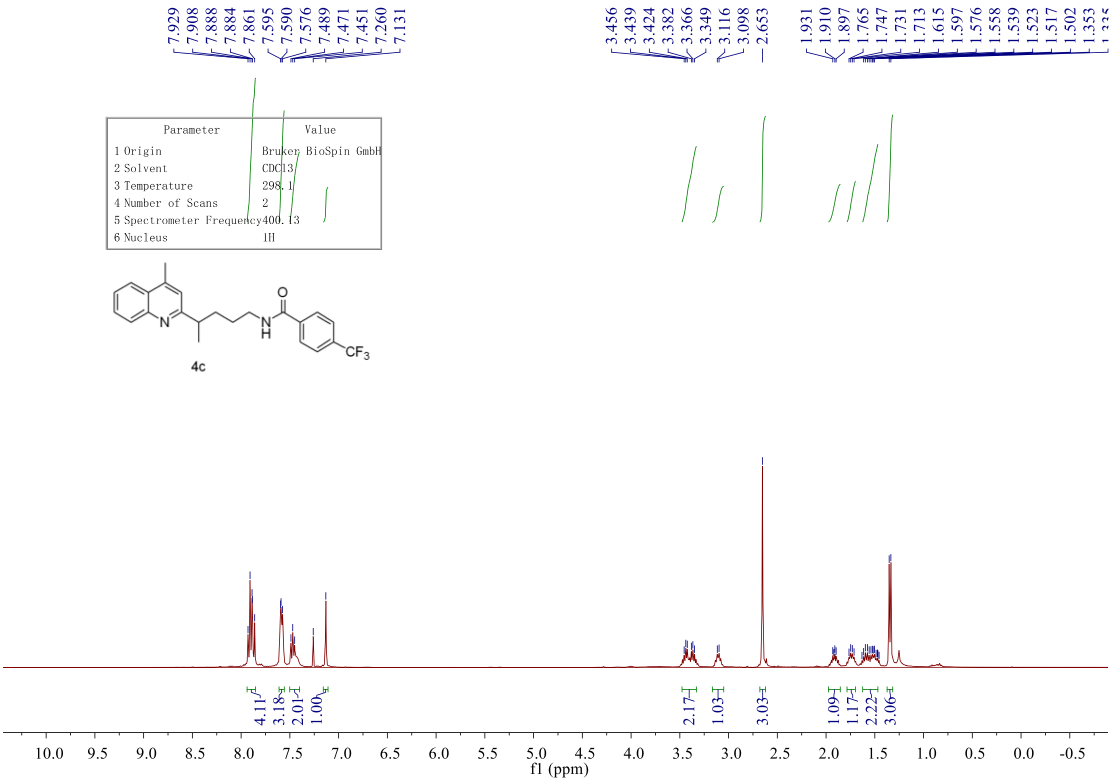
7.929 7.908 7.888 7.884 7.861 7.595 7.590 7.576 7.489 7.471 7.451 7.260 7.131
3.456 3.439 3.424 3.382 3.366 3.349 3.116 3.098 2.653
1.931 1.910 1.897 1.765 1.747 1.731 1.713 1.615 1.597 1.576 1.558 1.539 1.523 1.517 1.502 1.353
Parameter Value
1 Origin Bruker BioSpin GmbH
2 Solvent CDCl3
3 Temperature 298.1
4 Number of Scans 2
5 Spectrometer Frequency 400.13
6 Nucleus 1H
N
N H
O
CF3
4c
4.11 3.18 2.01 1.00 2.17 1.03 3.03 1.09 1.17 2.22 3.06
10.0 9.5 9.0 8.5 8.0 7.5 7.0 6.5 6.0 5.5 5.0 4.5 4.0 3.5 3.0 2.5 2.0 1.5 1.0 0.5 0.0 -0.5
f1 (ppm)

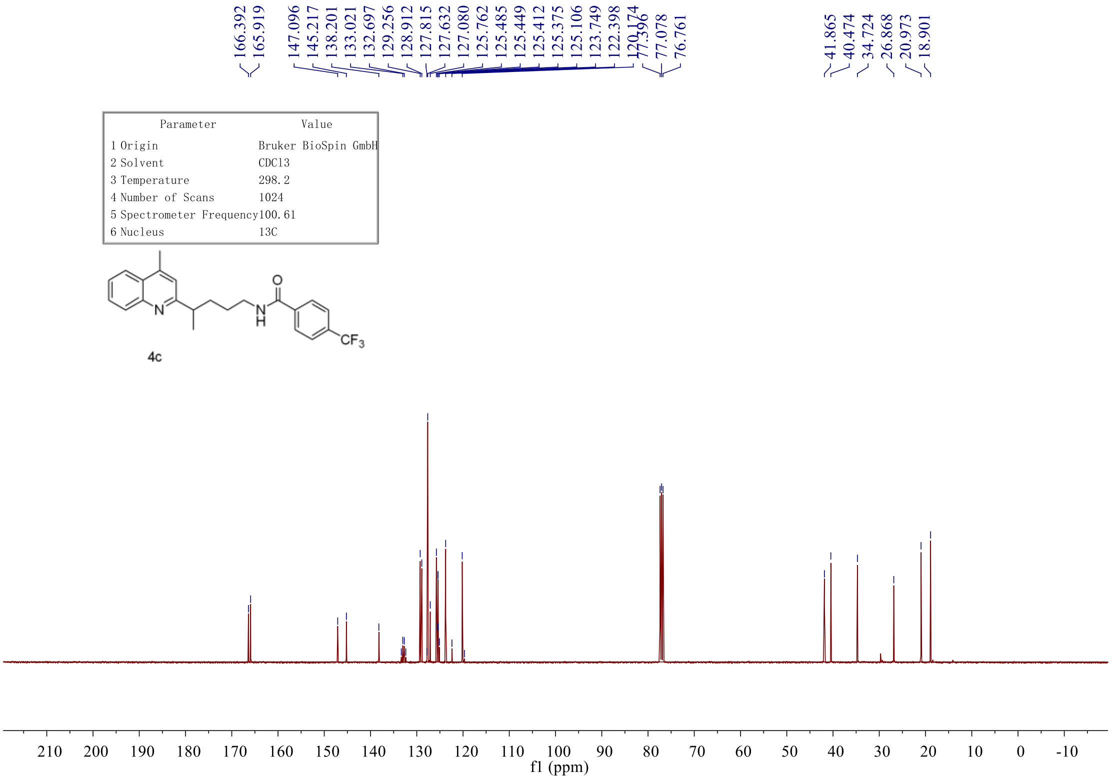
166.392 165.919
147.096 145.217 138.201 133.021 132.697 129.256 128.912 127.815 127.632 127.080 125.762 125.485 125.449 125.412 125.375 125.106 123.749 122.398 120.174 77.396 77.078 76.761
41.865 40.474 34.724 26.868 20.973 18.901
Parameter Value
1 Origin Bruker BioSpin GmbH
2 Solvent CDCl3
3 Temperature 298.2
4 Number of Scans 1024
5 Spectrometer Frequency 100.61
6 Nucleus 13C
N
N H
O
CF3
4c
210 200 190 180 170 160 150 140 130 120 110 100 90 80 70 60 50 40 30 20 10 0 -10
f1 (ppm)

Parameter	Value
1 Origin	Bruker BioSpin GmbH
2 Solvent	CDCl3
3 Temperature	298.2
4 Number of Scans	4
5 Spectrometer Frequency	376.46
6 Nucleus	19F

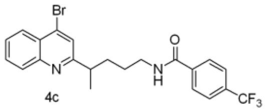

4c

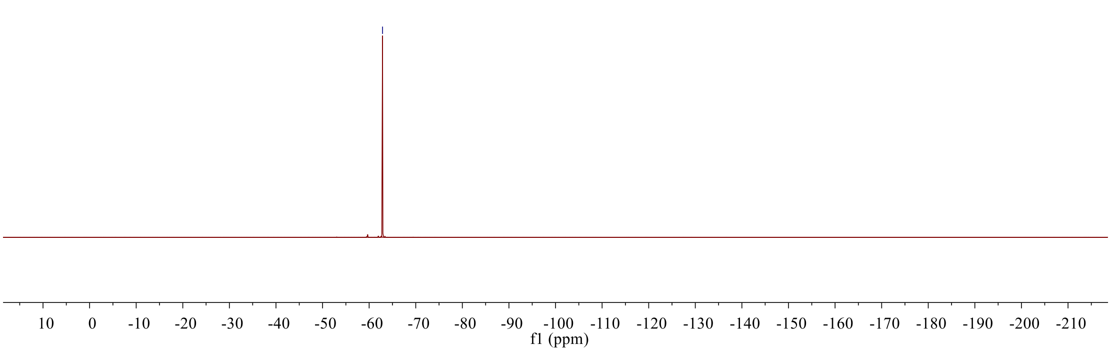

Parameter	Value
1 Origin	Bruker BioSpin GmbH
2 Solvent	CDCl3
3 Temperature	298.1
4 Number of Scans	2
5 Spectrometer Frequency	400.15
6 Nucleus	1H

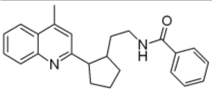

4d

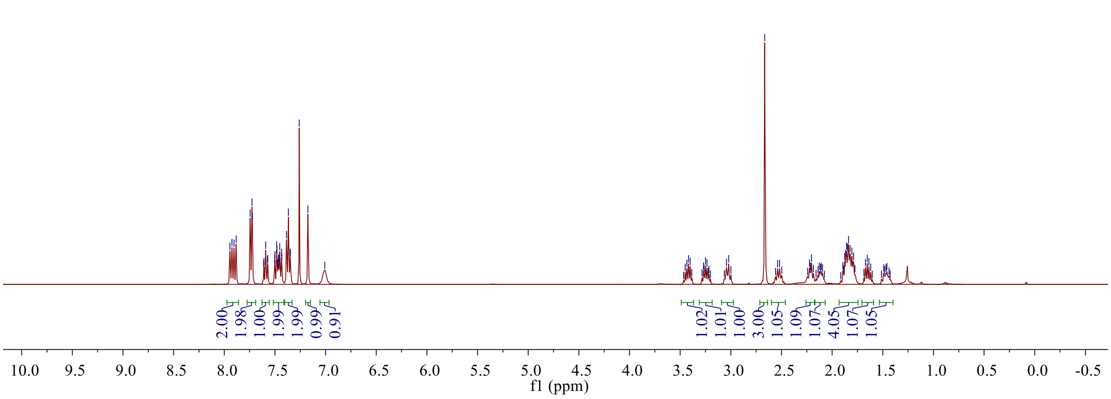

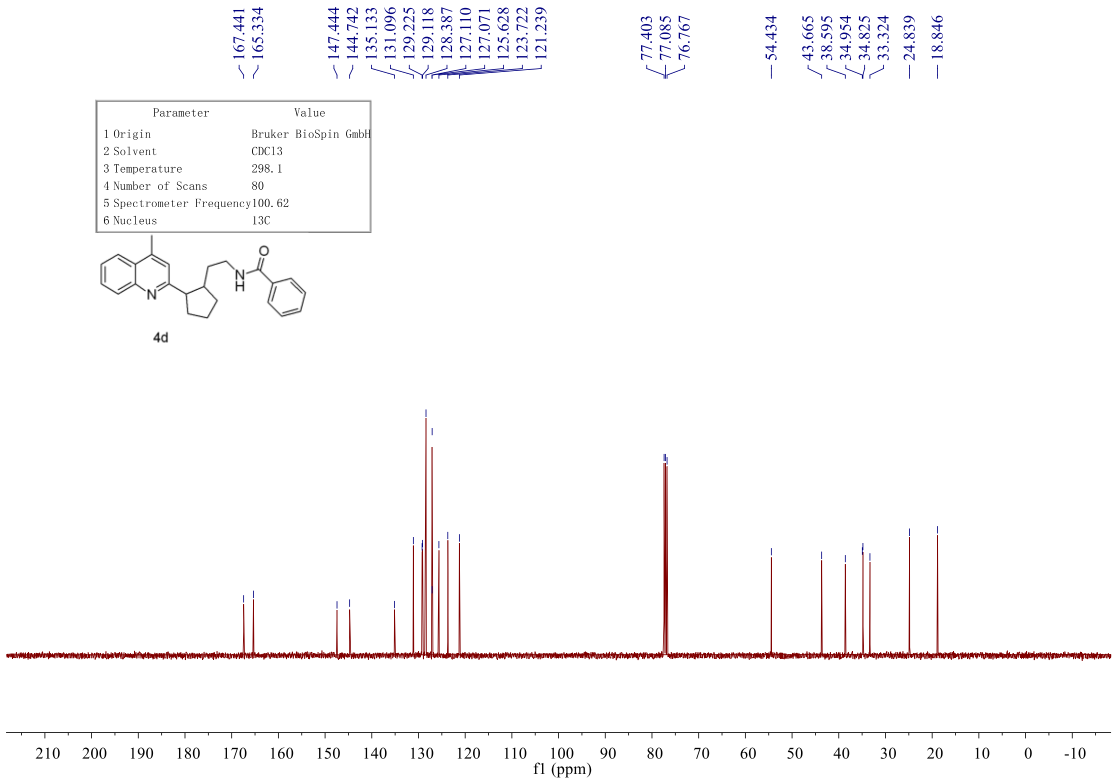
167.441
165.334
147.444
144.742
135.133
131.096
129.225
129.118
128.387
127.110
127.071
125.628
123.722
121.239
77.403
77.085
76.767
54.434
43.665
38.595
34.954
34.825
33.324
24.839
18.846
Parameter Value
1 Origin Bruker BioSpin GmbH
2 Solvent CDCl3
3 Temperature 298.1
4 Number of Scans 80
5 Spectrometer Frequency 100.62
6 Nucleus 13C
N
H
O
4d
210 200 190 180 170 160 150 140 130 120 110 100 90 80 70 60 50 40 30 20 10 0 -10
f1 (ppm)

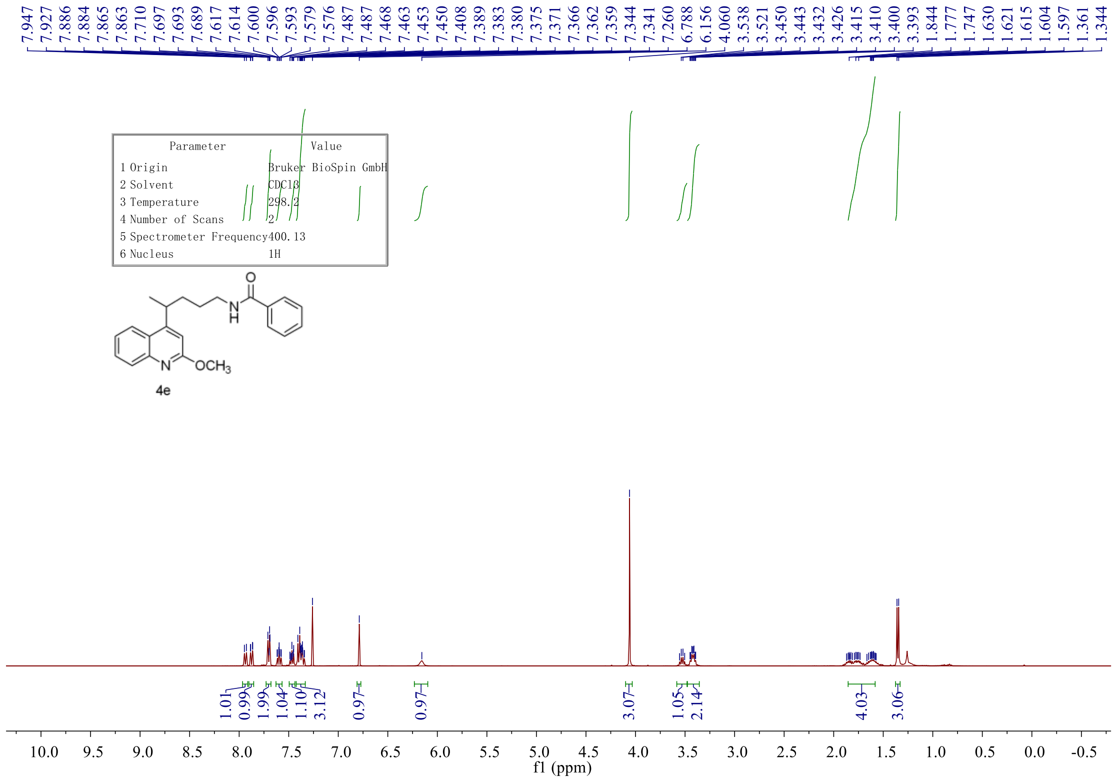
7.947
7.927
7.886
7.884
7.865
7.863
7.710
7.697
7.693
7.689
7.617
7.614
7.600
7.596
7.593
7.579
7.576
7.487
7.487
7.468
7.463
7.453
7.450
7.408
7.389
7.383
7.380
7.375
7.371
7.366
7.362
7.359
7.344
7.341
7.260
6.788
6.156
4.060
3.538
3.521
3.450
3.443
3.432
3.426
3.415
3.410
3.400
3.393
1.844
1.777
1.747
1.630
1.621
1.615
1.604
1.597
1.361
1.344
Parameter Value
1 Origin Bruker BioSpin GmbH
2 Solvent CDCl3
3 Temperature 298.2
4 Number of Scans 2
5 Spectrometer Frequency 400.13
6 Nucleus 1H
N
H
O
OCH3
4e
1.01
0.99
1.99
1.04
1.10
3.12
0.97
0.97
3.07
1.05
2.14
4.03
3.06
10.0 9.5 9.0 8.5 8.0 7.5 7.0 6.5 6.0 5.5 5.0 4.5 4.0 3.5 3.0 2.5 2.0 1.5 1.0 0.5 0.0 -0.5
f1 (ppm)

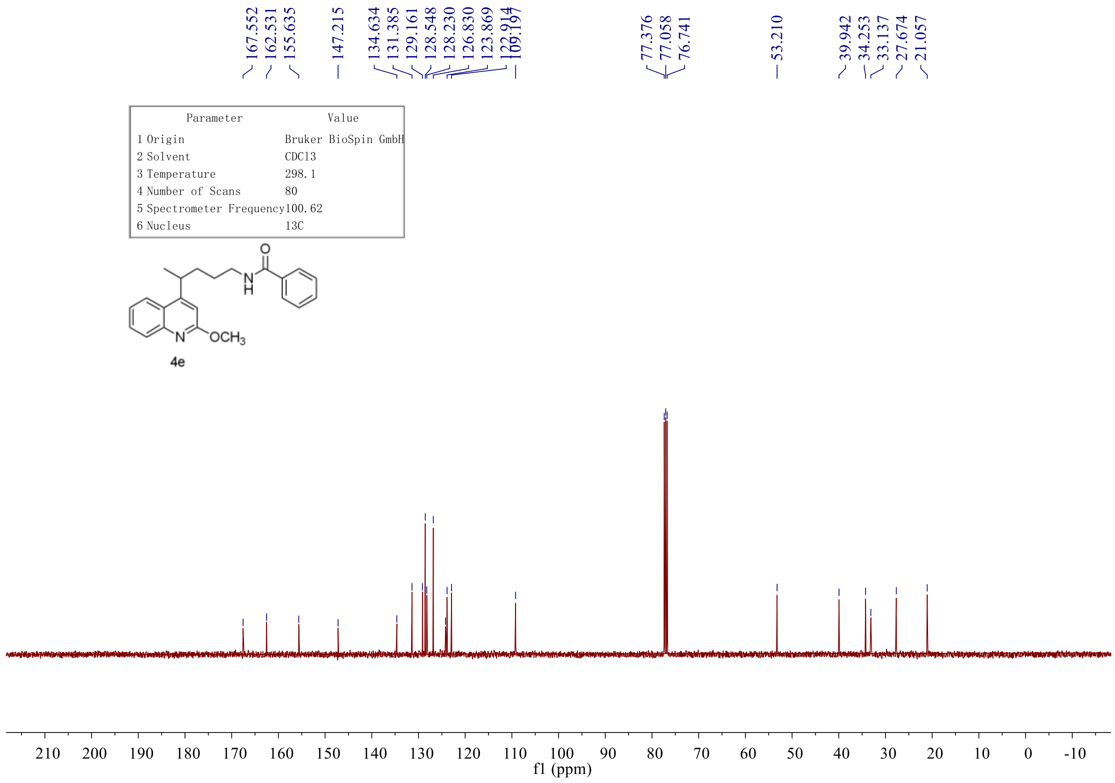
167.552
162.531
155.635
147.215
134.634
131.385
129.161
128.548
128.230
126.830
123.869
122.914
109.197
77.376
77.058
76.741
53.210
39.942
34.253
33.137
27.674
21.057
Parameter Value
1 Origin Bruker BioSpin GmbH
2 Solvent CDCl3
3 Temperature 298.1
4 Number of Scans 80
5 Spectrometer Frequency 100.62
6 Nucleus 13C
O
N
H
N
OCH3
4e
f1 (ppm)

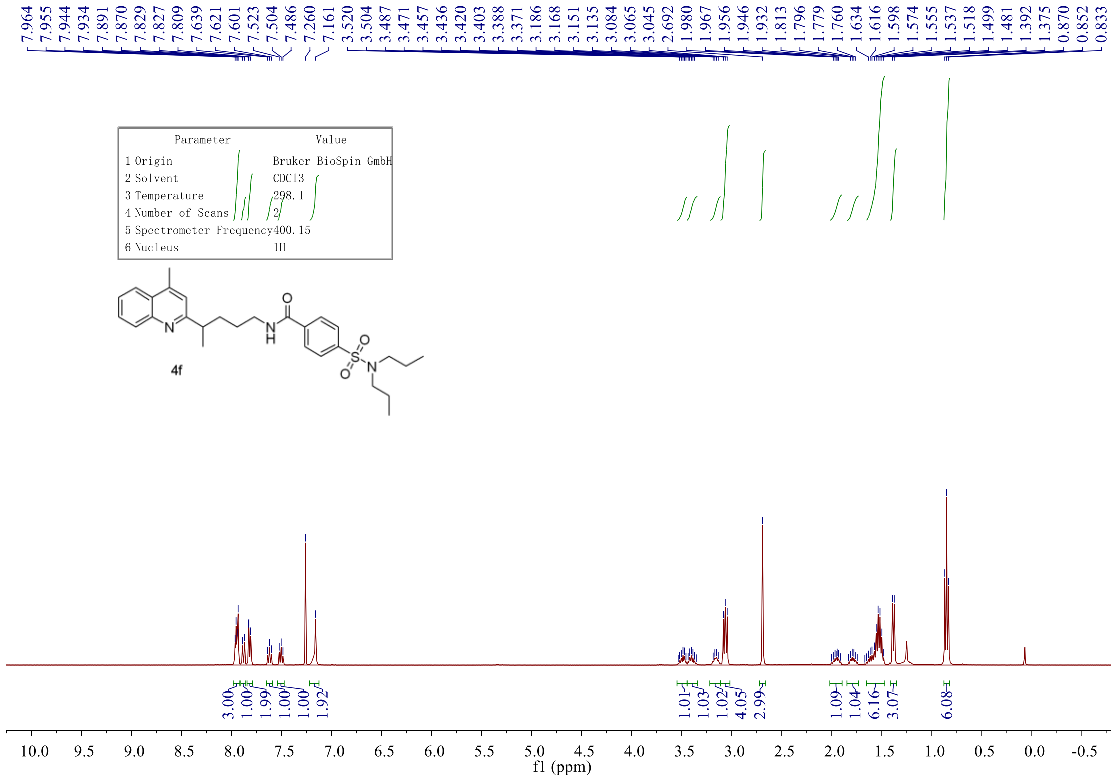
7.964
7.955
7.944
7.934
7.891
7.870
7.829
7.827
7.809
7.639
7.621
7.601
7.523
7.504
7.486
7.260
7.161
3.520
3.504
3.487
3.471
3.457
3.436
3.420
3.403
3.388
3.371
3.186
3.168
3.151
3.135
3.084
3.065
3.045
2.692
1.980
1.967
1.956
1.946
1.932
1.813
1.796
1.779
1.760
1.634
1.616
1.598
1.574
1.555
1.537
1.518
1.499
1.481
1.392
1.375
0.870
0.852
0.833
Parameter Value
1 Origin Bruker BioSpin GmbH
2 Solvent CDCl3
3 Temperature 298.1
4 Number of Scans 2
5 Spectrometer Frequency 400.15
6 Nucleus 1H
N
O
N
H
O
S
O
N
4f
3.00
1.00
1.99
1.00
1.00
1.92
1.01
1.03
1.02
4.05
2.99
1.09
1.04
6.16
3.07
6.08
f1 (ppm)

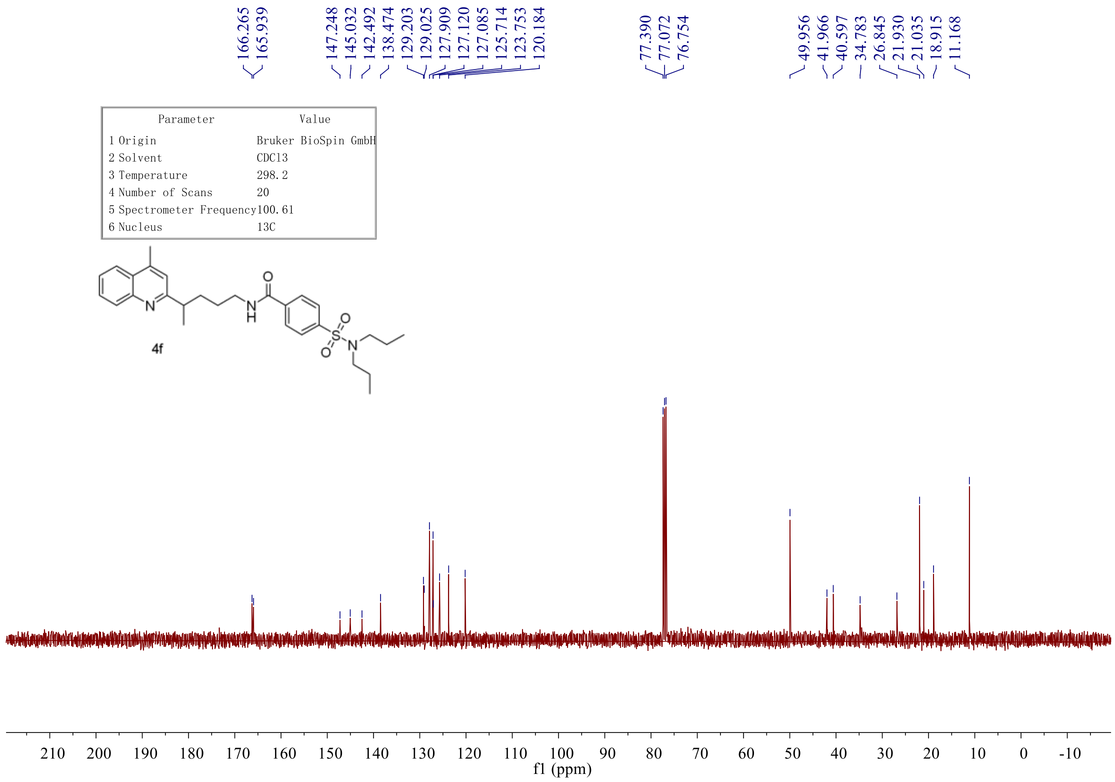

166.265
165.939
147.248
145.032
142.492
138.474
129.203
129.025
127.909
127.120
127.085
125.714
123.753
120.184
77.390
77.072
76.754
49.956
41.966
40.597
34.783
26.845
21.930
21.035
18.915
11.168
Parameter Value
1 Origin Bruker BioSpin GmbH
2 Solvent CDCl3
3 Temperature 298.2
4 Number of Scans 20
5 Spectrometer Frequency 100.61
6 Nucleus 13C
4f
210 200 190 180 170 160 150 140 130 120 110 100 90 80 70 60 50 40 30 20 10 0 -10
f1 (ppm)

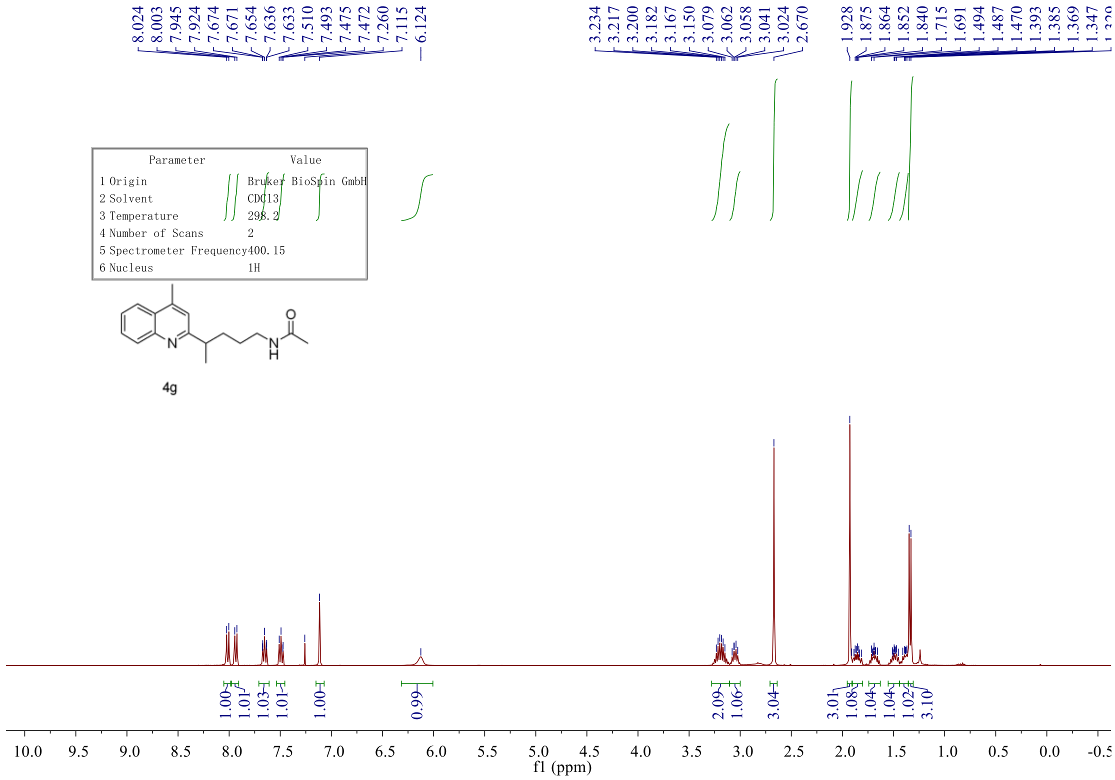

8.024
8.003
7.945
7.924
7.674
7.671
7.654
7.636
7.633
7.510
7.493
7.475
7.472
7.260
7.115
6.124
3.234
3.217
3.200
3.182
3.167
3.150
3.079
3.062
3.058
3.041
3.024
2.670
1.928
1.875
1.864
1.852
1.840
1.715
1.691
1.494
1.487
1.470
1.393
1.385
1.369
1.347
Parameter Value
1 Origin Bruker BioSpin GmbH
2 Solvent CDCl3
3 Temperature 298.2
4 Number of Scans 2
5 Spectrometer Frequency 400.15
6 Nucleus 1H
4g
1.00
1.01
1.03
1.01
1.00
0.99
2.09
1.06
3.04
3.01
1.08
1.04
1.04
1.02
3.10
10.0 9.5 9.0 8.5 8.0 7.5 7.0 6.5 6.0 5.5 5.0 4.5 4.0 3.5 3.0 2.5 2.0 1.5 1.0 0.5 0.0
f1 (ppm)

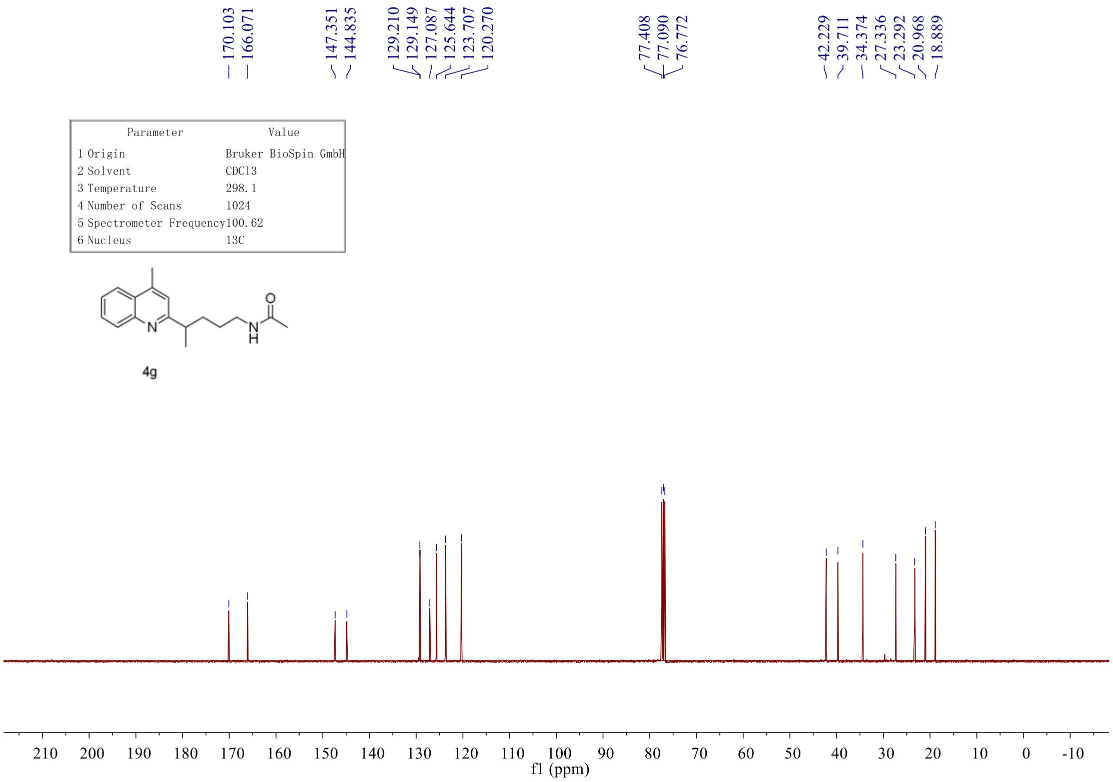

Parameter	Value
1 Origin	Bruker BioSpin GmbH
2 Solvent	CDCl3
3 Temperature	298.1
4 Number of Scans	1024
5 Spectrometer Frequency	100.62
6 Nucleus	13C

4g

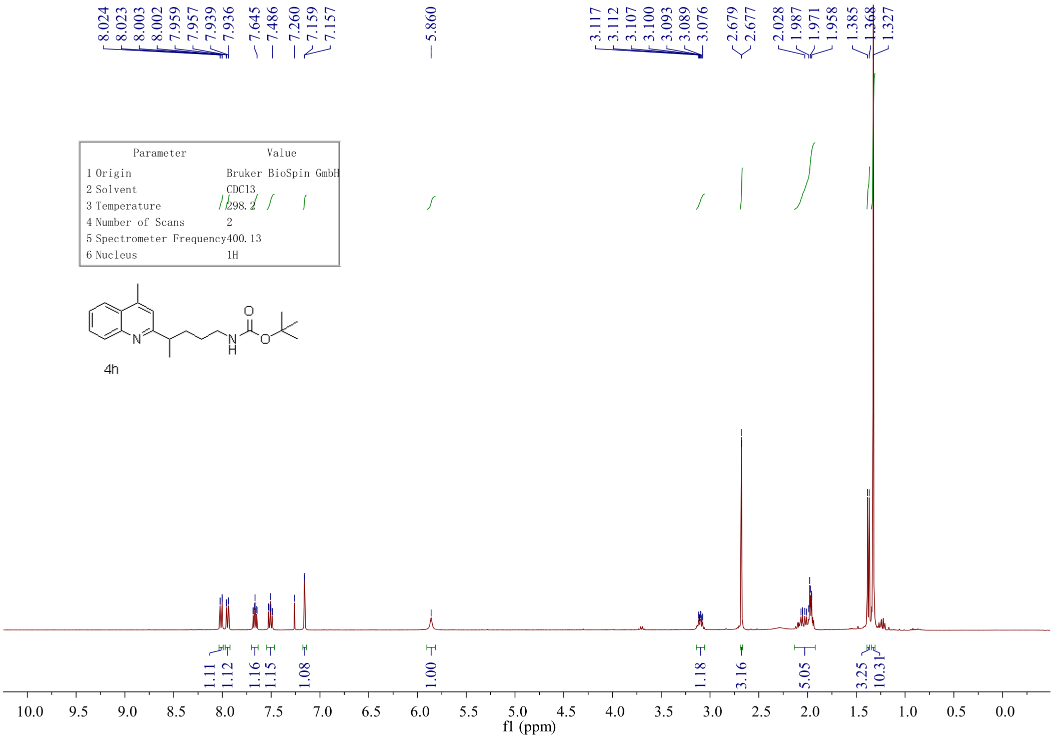

Parameter	Value
1 Origin	Bruker BioSpin GmbH
2 Solvent	CDCl3
3 Temperature	298.2
4 Number of Scans	2
5 Spectrometer Frequency	400.13
6 Nucleus	1H

4h

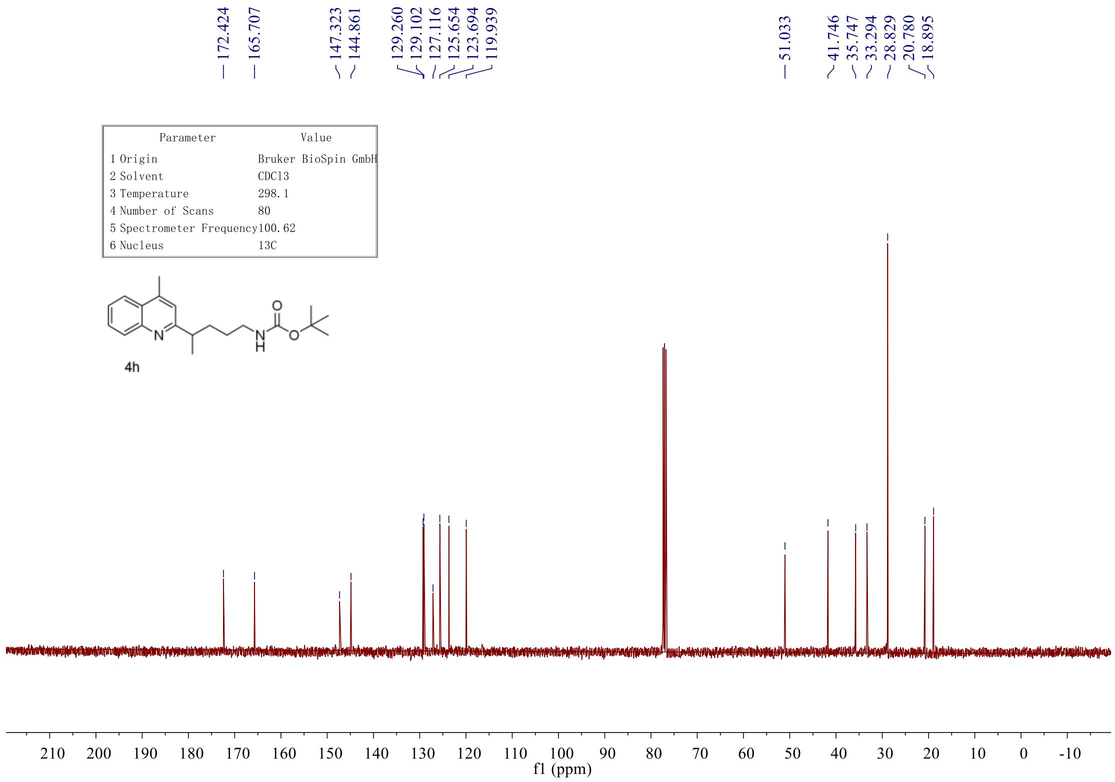
172.424
165.707
147.323
144.861
129.260
129.102
127.116
125.654
123.694
119.939
51.033
41.746
35.747
33.294
28.829
20.780
18.895
Parameter Value
1 Origin Bruker BioSpin GmbH
2 Solvent CDCl3
3 Temperature 298.1
4 Number of Scans 80
5 Spectrometer Frequency 100.62
6 Nucleus 13C
4h
f1 (ppm)

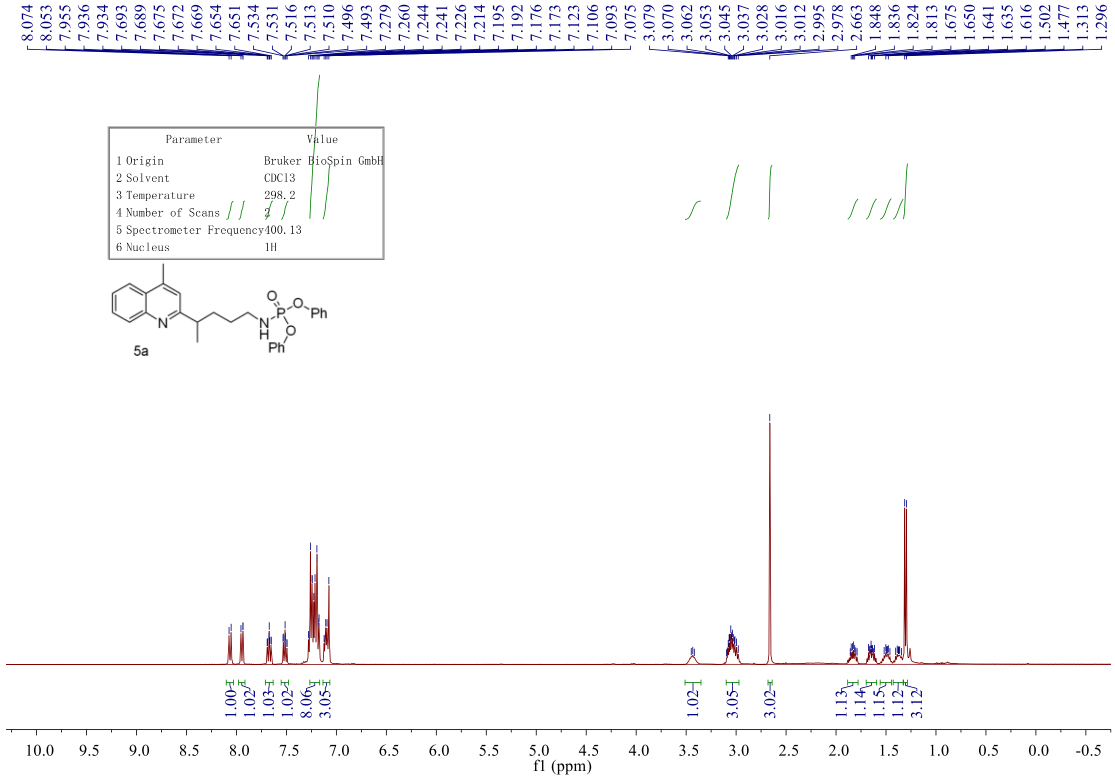
8.074
8.053
7.955
7.936
7.934
7.693
7.689
7.675
7.672
7.669
7.654
7.651
7.534
7.531
7.516
7.513
7.510
7.496
7.493
7.279
7.260
7.244
7.241
7.226
7.214
7.195
7.192
7.176
7.173
7.123
7.106
7.093
7.075
3.079
3.070
3.062
3.053
3.045
3.037
3.028
3.016
3.012
2.995
2.978
2.663
1.848
1.836
1.824
1.813
1.675
1.650
1.641
1.635
1.616
1.502
1.477
1.313
1.296
Parameter Value
1 Origin Bruker BioSpin GmbH
2 Solvent CDCl3
3 Temperature 298.2
4 Number of Scans 2
5 Spectrometer Frequency 400.13
6 Nucleus 1H
5a
1.00
1.02
1.03
1.02
8.06
3.05
1.02
3.05
3.02
1.13
1.14
1.15
1.12
3.12
f1 (ppm)

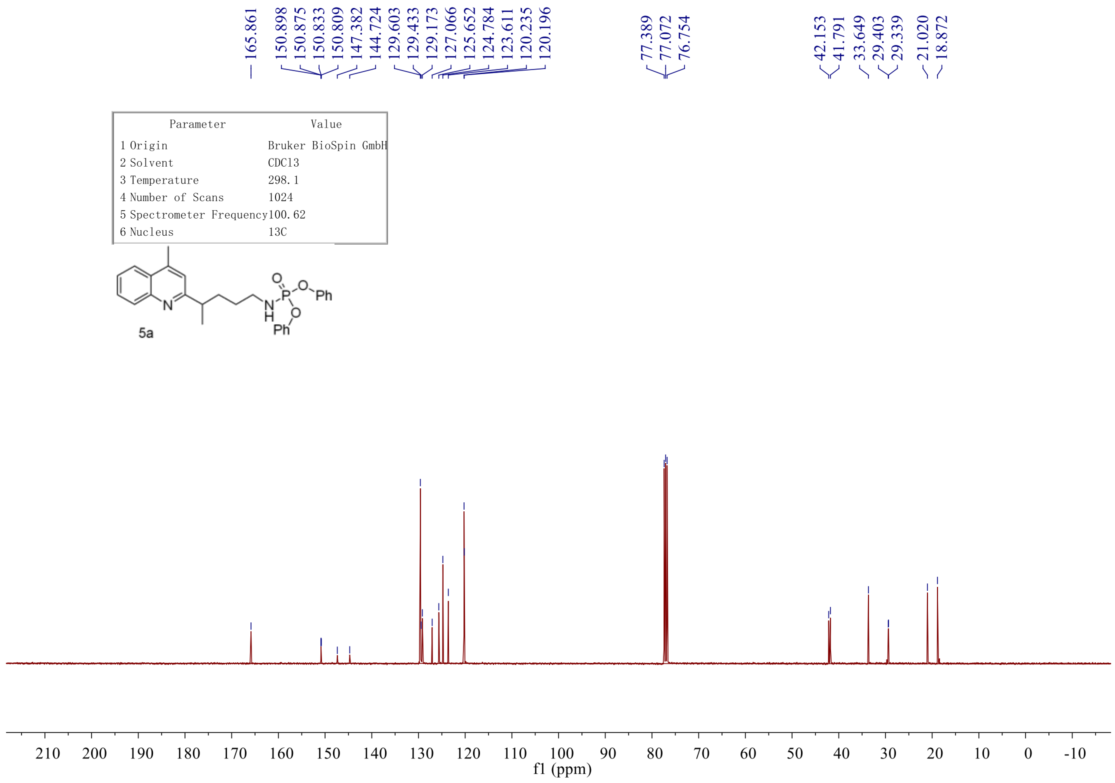
165.861
150.898
150.875
150.833
150.809
147.382
144.724
129.603
129.433
129.173
127.066
125.652
124.784
123.611
120.235
120.196
77.389
77.072
76.754
42.153
41.791
33.649
29.403
29.339
21.020
18.872
Parameter Value
1 Origin Bruker BioSpin GmbH
2 Solvent CDCl3
3 Temperature 298.1
4 Number of Scans 1024
5 Spectrometer Frequency 100.62
6 Nucleus 13C
N
O
P
O
Ph
N
H
O
Ph
5a
210 200 190 180 170 160 150 140 130 120 110 100 90 80 70 60 50 40 30 20 10 0 -10
f1 (ppm)

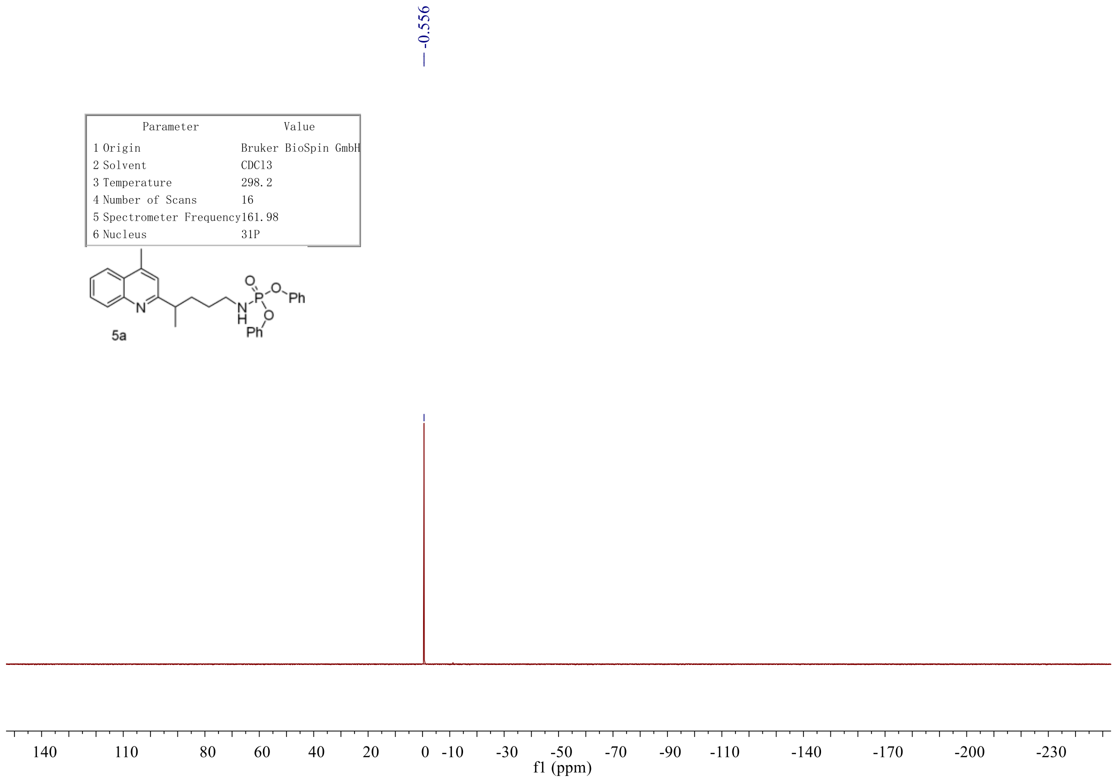
-0.556
Parameter Value
1 Origin Bruker BioSpin GmbH
2 Solvent CDCl3
3 Temperature 298.2
4 Number of Scans 16
5 Spectrometer Frequency 161.98
6 Nucleus 31P
N
O
P
O
Ph
N
H
O
Ph
5a
140 110 80 60 40 20 0 -10 -30 -50 -70 -90 -110 -140 -170 -200 -230
f1 (ppm)

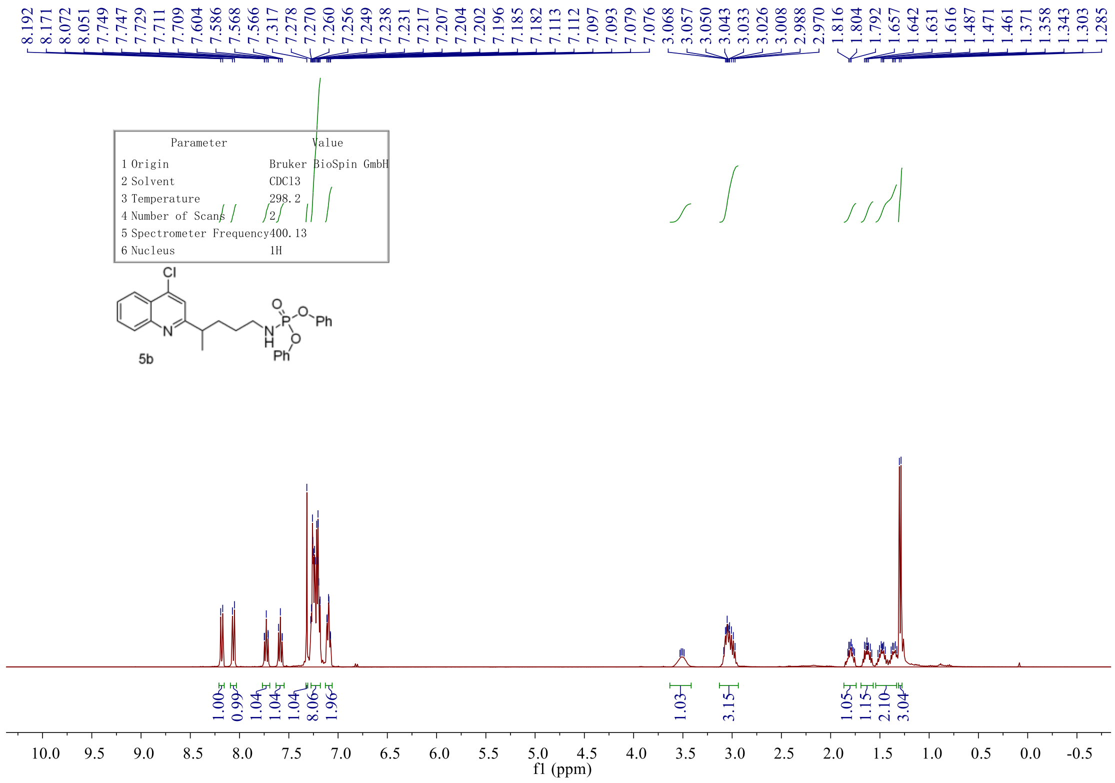

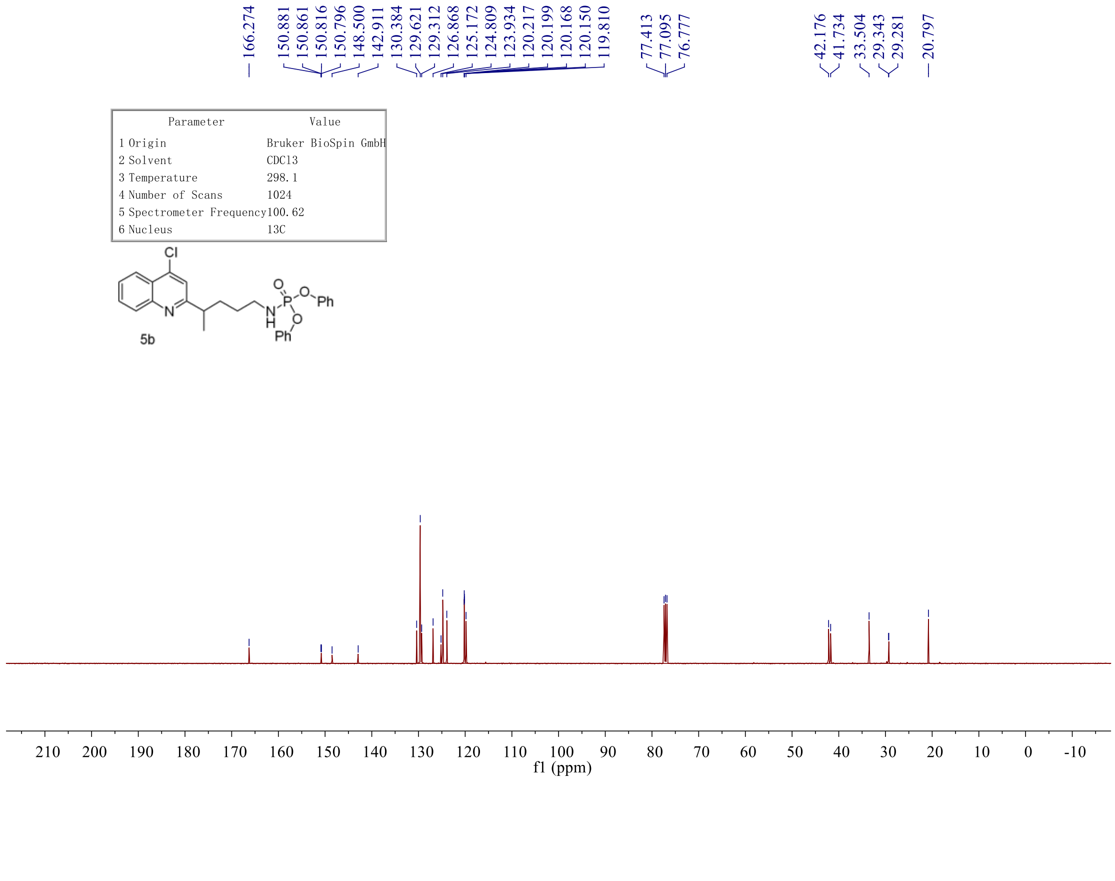

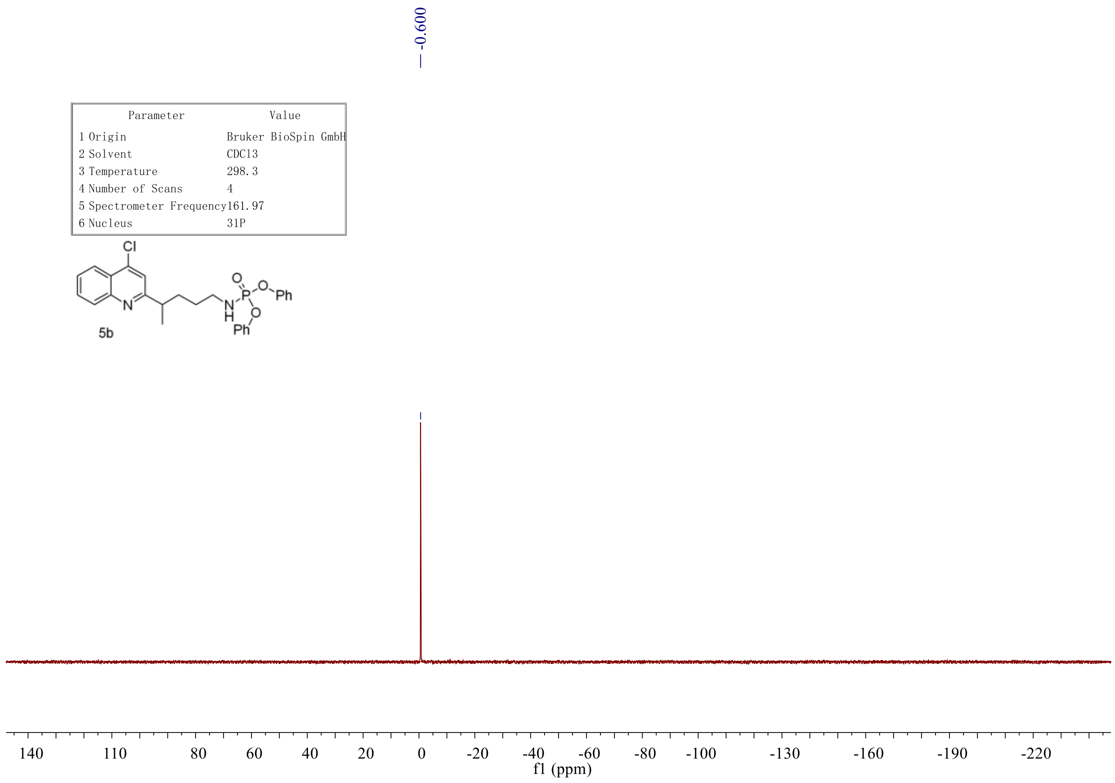

Parameter	Value
1 Origin	Bruker BioSpin GmbH
2 Solvent	CDCl3
3 Temperature	298.3
4 Number of Scans	4
5 Spectrometer Frequency	161.97
6 Nucleus	31P

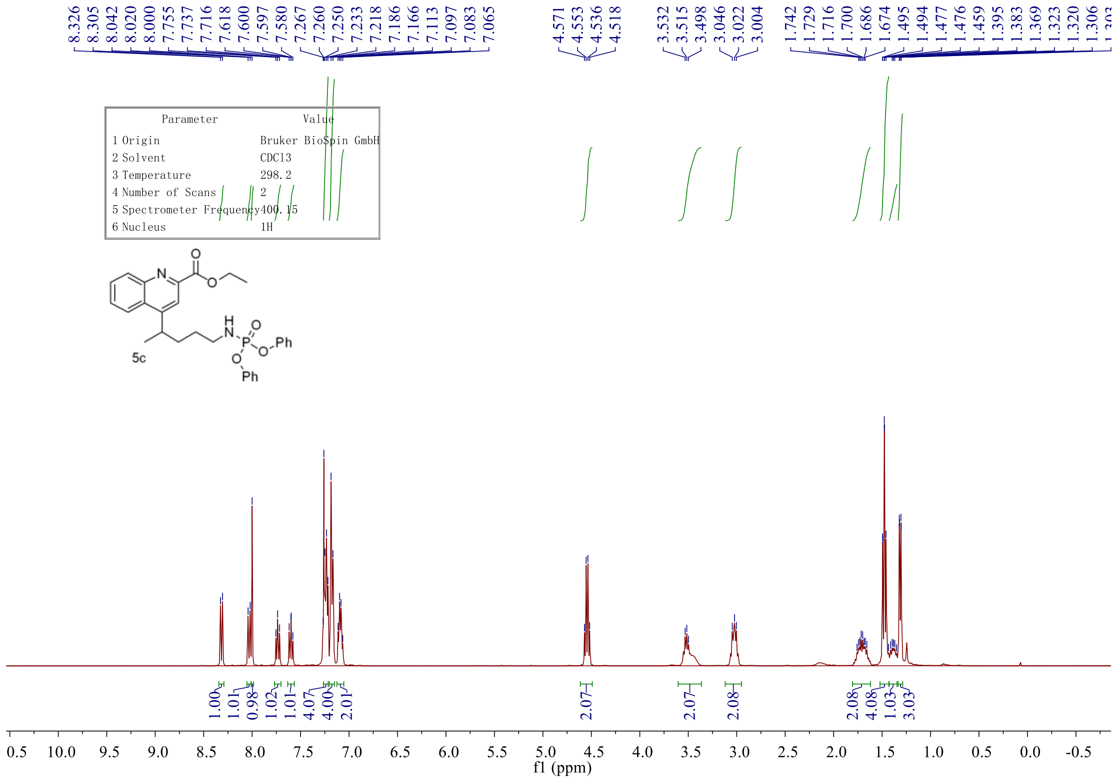

Parameter	Value
1 Origin	Bruker BioSpin GmbH
2 Solvent	CDCl3
3 Temperature	298.2
4 Number of Scans	2
5 Spectrometer Frequency	400.15
6 Nucleus	1H

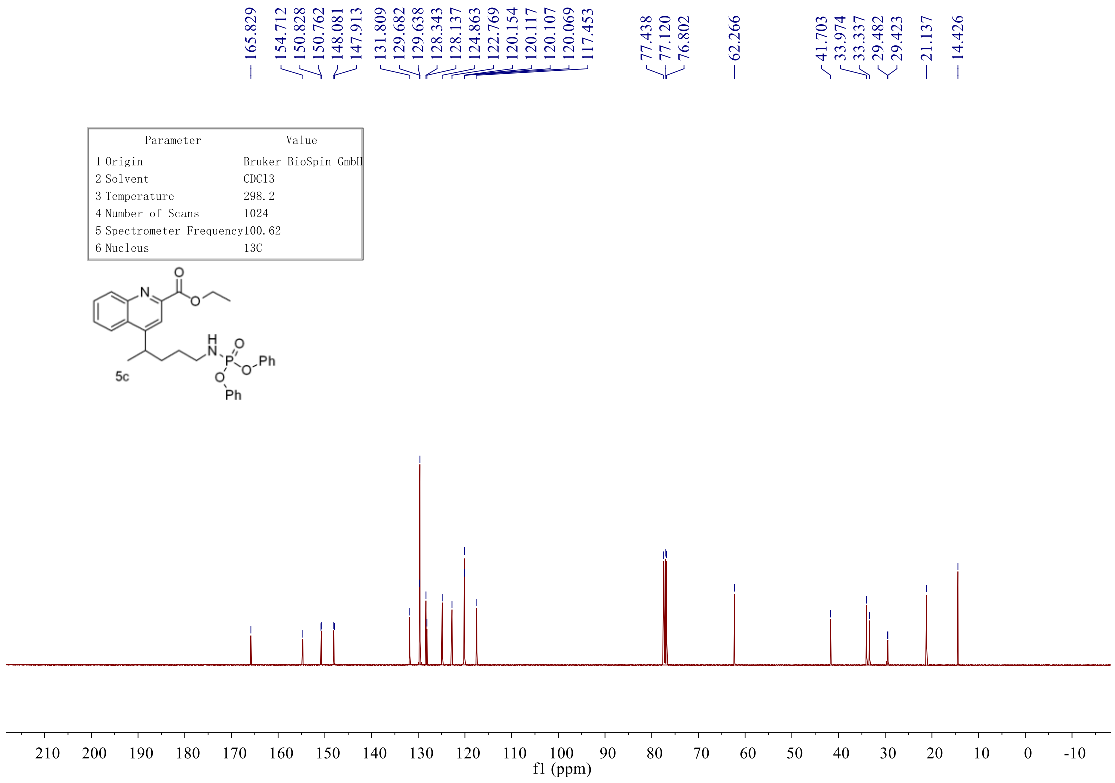
165.829
154.712
150.828
150.762
148.081
147.913
131.809
129.682
129.638
128.343
128.137
124.863
122.769
120.154
120.117
120.107
120.069
117.453
77.438
77.120
76.802
62.266
41.703
33.974
33.337
29.482
29.423
21.137
14.426
Parameter Value
1 Origin Bruker BioSpin GmbH
2 Solvent CDCl3
3 Temperature 298.2
4 Number of Scans 1024
5 Spectrometer Frequency 100.62
6 Nucleus 13C
5c
f1 (ppm)

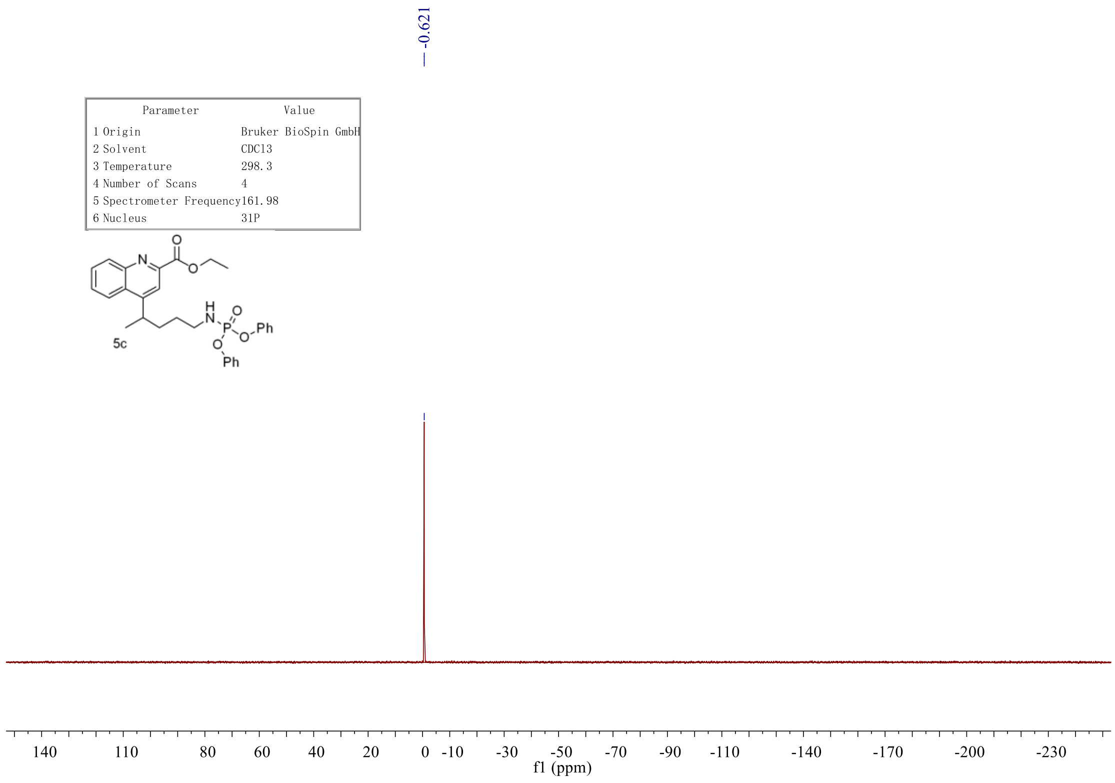
-0.621
Parameter Value
1 Origin Bruker BioSpin GmbH
2 Solvent CDCl3
3 Temperature 298.3
4 Number of Scans 4
5 Spectrometer Frequency 161.98
6 Nucleus 31P
5c
f1 (ppm)

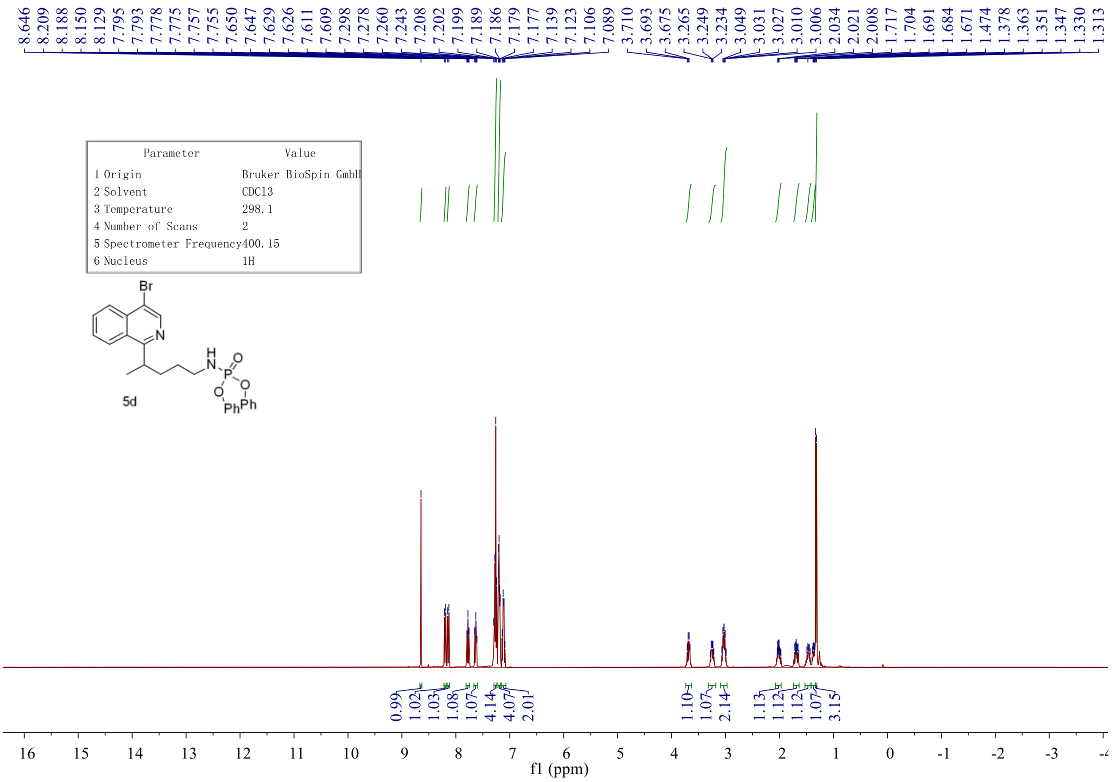

Parameter	Value
1 Origin	Bruker BioSpin GmbH
2 Solvent	CDCl3
3 Temperature	298.1
4 Number of Scans	2
5 Spectrometer Frequency	400.15
6 Nucleus	1H

5d

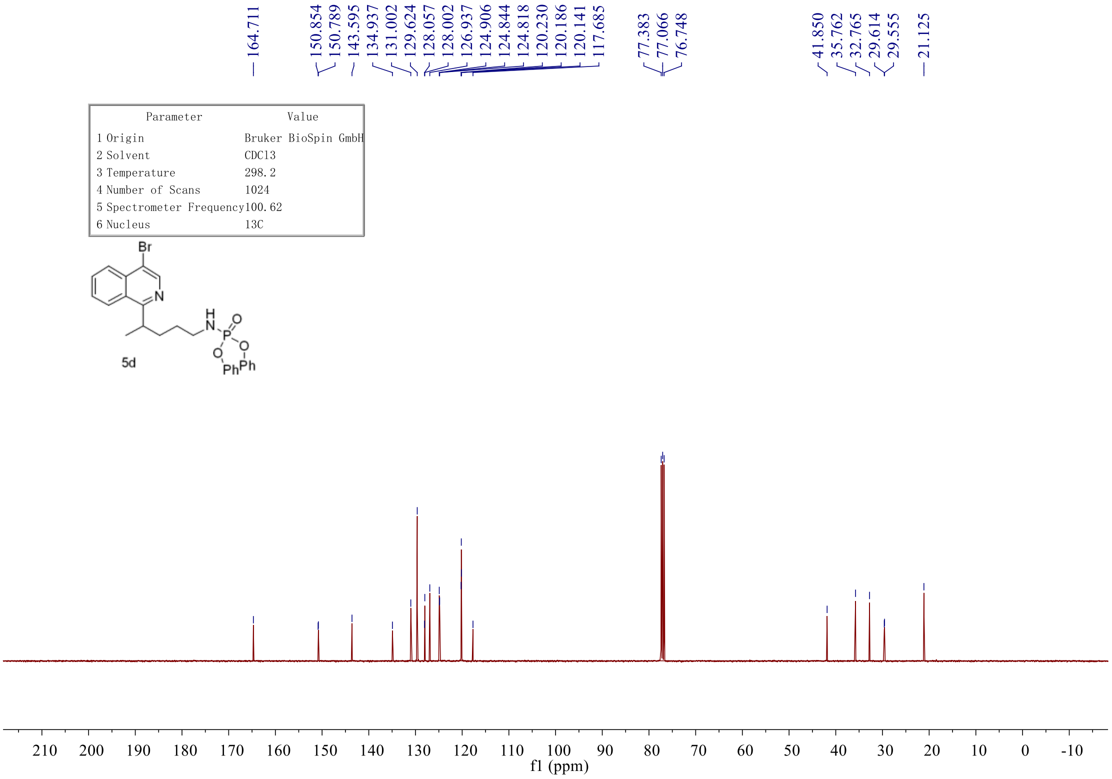

Parameter	Value
1 Origin	Bruker BioSpin GmbH
2 Solvent	CDCl3
3 Temperature	298.2
4 Number of Scans	1024
5 Spectrometer Frequency	100.62
6 Nucleus	13C

5d

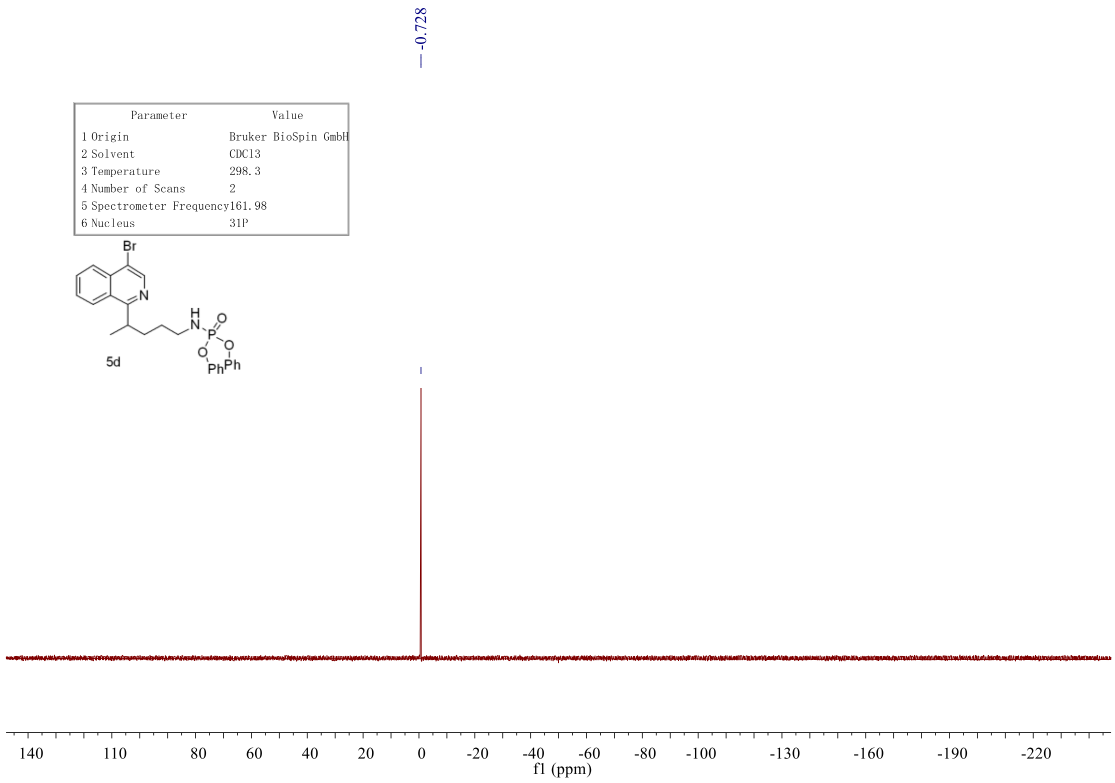
-0.728
Parameter Value
1 Origin Bruker BioSpin GmbH
2 Solvent CDCl3
3 Temperature 298.3
4 Number of Scans 2
5 Spectrometer Frequency 161.98
6 Nucleus 31P
Br
N
H
N
P
O
O
O
Ph
Ph
5d
140 110 80 60 40 20 0 -20 -40 -60 -80 -100 -130 -160 -190 -220
f1 (ppm)

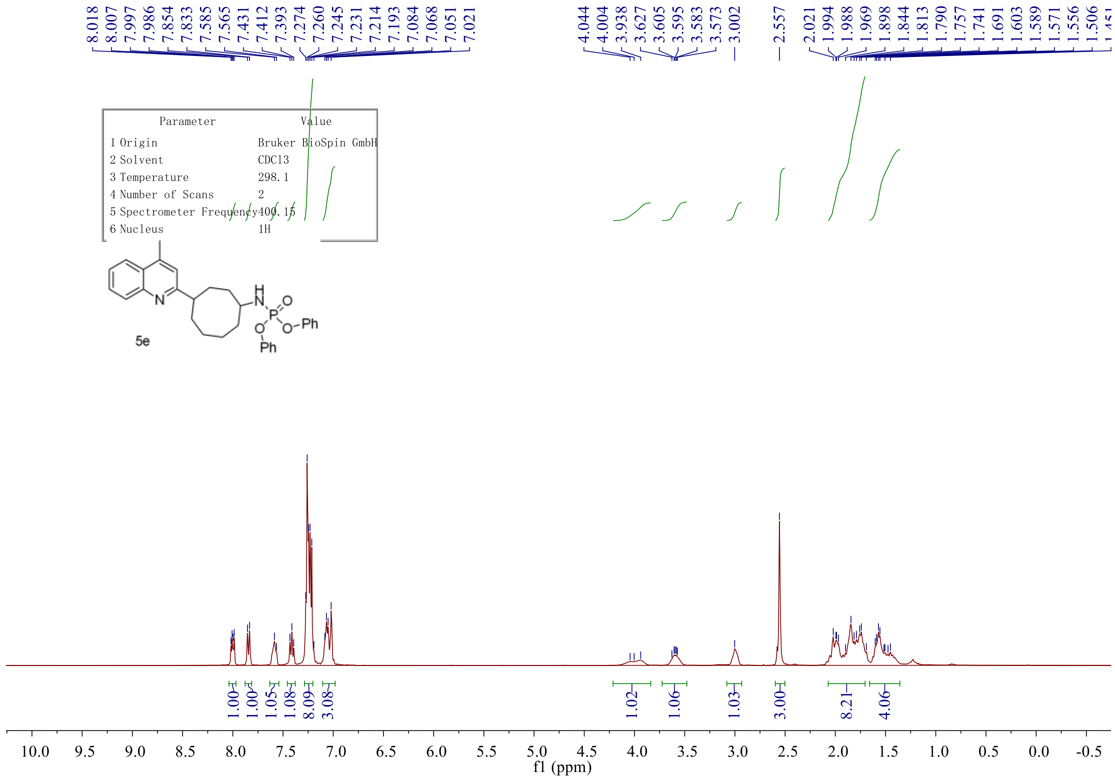
8.018 8.007 7.997 7.986 7.854 7.833 7.585 7.565 7.431 7.412 7.393 7.274 7.260 7.245 7.231 7.214 7.193 7.084 7.068 7.051 7.021
4.044 4.004 3.938 3.627 3.605 3.595 3.583 3.573 3.002 2.557 2.021 1.994 1.988 1.969 1.898 1.844 1.813 1.790 1.757 1.741 1.691 1.603 1.589 1.571 1.556 1.506
Parameter Value
1 Origin Bruker BioSpin GmbH
2 Solvent CDCl3
3 Temperature 298.1
4 Number of Scans 2
5 Spectrometer Frequency 400.15
6 Nucleus 1H
N
H
N
P
O
O
O
Ph
Ph
5e
1.00 1.00 1.05 1.08 8.09 3.08 1.02 1.06 1.03 3.00 8.21 4.06
10.0 9.5 9.0 8.5 8.0 7.5 7.0 6.5 6.0 5.5 5.0 4.5 4.0 3.5 3.0 2.5 2.0 1.5 1.0 0.5 0.0 -0.5
f1 (ppm)

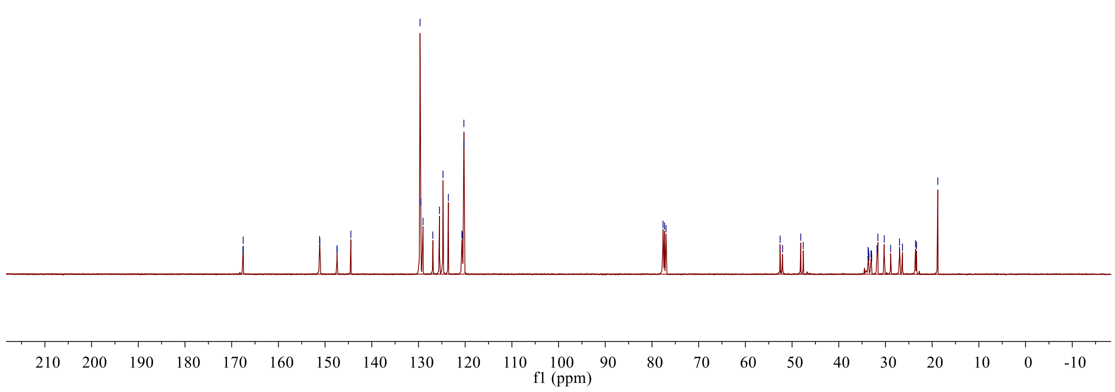

Parameter	Value
1 Origin	Bruker BioSpin GmbH
2 Solvent	CDCl3
3 Temperature	298.2
4 Number of Scans	1024
5 Spectrometer Frequency	100.62
6 Nucleus	13C

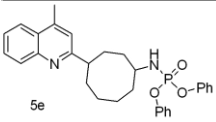

5e

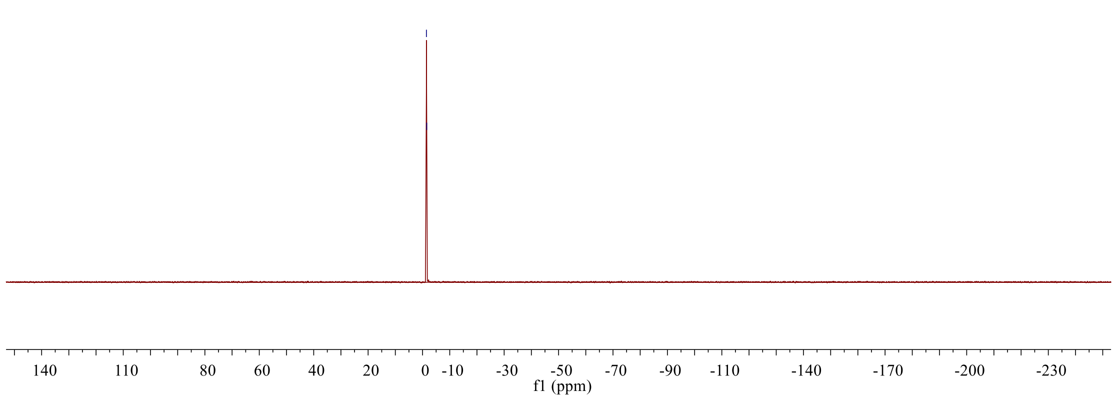

Parameter	Value
1 Origin	Bruker BioSpin GmbH
2 Solvent	CDCl3
3 Temperature	298.3
4 Number of Scans	2
5 Spectrometer Frequency	161.98
6 Nucleus	31P

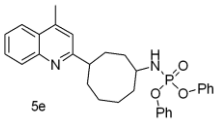

5e

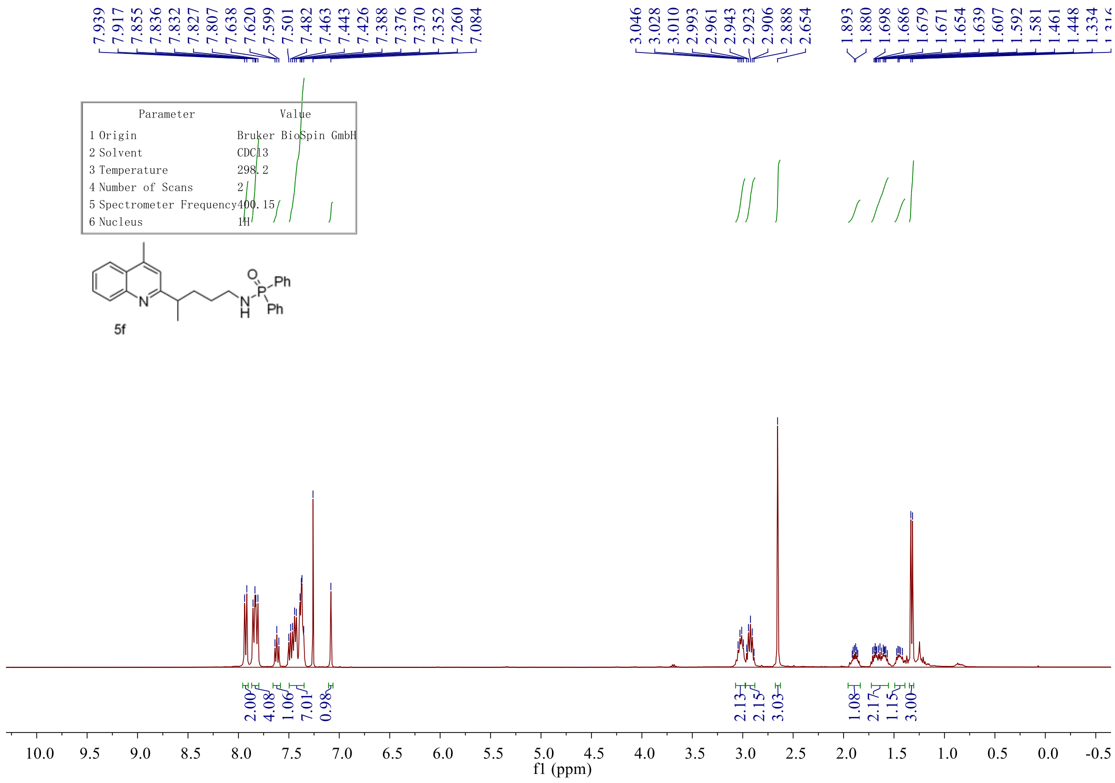
7.939 7.917 7.855 7.836 7.832 7.827 7.807 7.638 7.620 7.599 7.501 7.482 7.463 7.443 7.426 7.388 7.376 7.370 7.352 7.260 7.084
3.046 3.028 3.010 2.993 2.961 2.943 2.923 2.906 2.888 2.654
1.893 1.880 1.698 1.686 1.679 1.671 1.654 1.639 1.607 1.592 1.581 1.461 1.448 1.334
Parameter Value
1 Origin Bruker BioSpin GmbH
2 Solvent CDCl3
3 Temperature 298.2
4 Number of Scans 2
5 Spectrometer Frequency 400.15
6 Nucleus 1H
O
P
Ph
Ph
N
H
N
5f
2.00 4.08 1.06 7.01 0.98
2.13 2.15 3.03
1.08 2.17 1.15 3.00
10.0 9.5 9.0 8.5 8.0 7.5 7.0 6.5 6.0 5.5 5.0 4.5 4.0 3.5 3.0 2.5 2.0 1.5 1.0 0.5 0.0 -0.5
f1 (ppm)

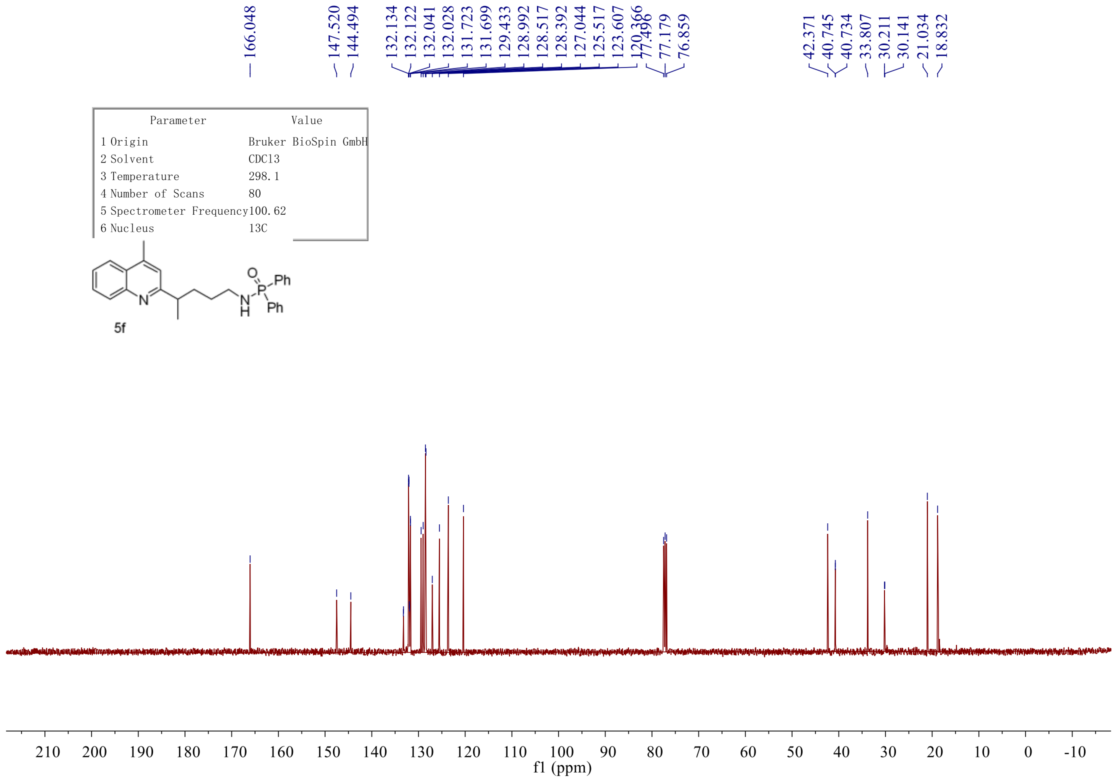
166.048
147.520 144.494
132.134 132.122 132.041 132.028 131.723 131.699 129.433 128.992 128.517 128.392 127.044 125.517 123.607 120.366 77.496 77.179 76.859
42.371 40.745 40.734 33.807 30.211 30.141 21.034 18.832
Parameter Value
1 Origin Bruker BioSpin GmbH
2 Solvent CDCl3
3 Temperature 298.1
4 Number of Scans 80
5 Spectrometer Frequency 100.62
6 Nucleus 13C
O
P
Ph
Ph
N
H
N
5f
210 200 190 180 170 160 150 140 130 120 110 100 90 80 70 60 50 40 30 20 10 0 -10
f1 (ppm)

Parameter	Value
1 Origin	Bruker BioSpin GmbH
2 Solvent	CDCl3
3 Temperature	298.3
4 Number of Scans	4
5 Spectrometer Frequency	161.97
6 Nucleus	31P

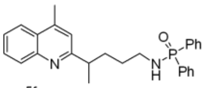

5f

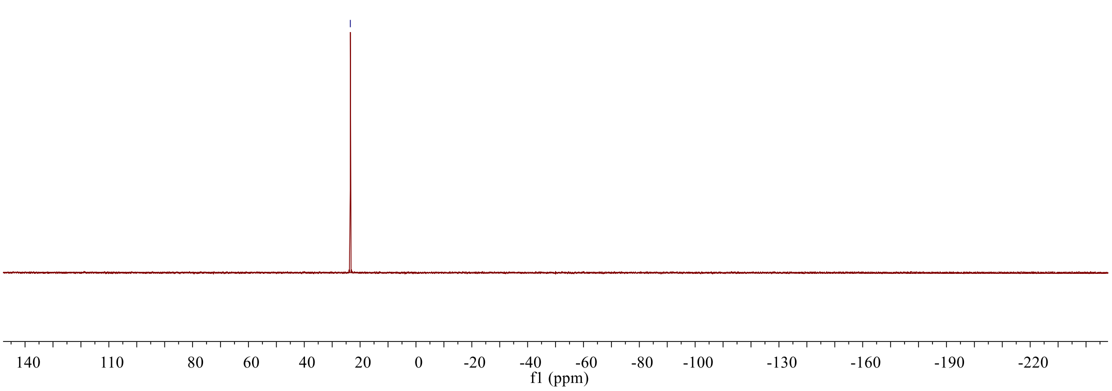

8. Absorption and emission studies

Solutions of different complexes were introduced to a 1 cm path length quartz cuvette equipped with a Teflon® septum and analyzed using an Agilent Cary 5000 spectrophotometer.

For the solutions of benzenesulfonamide **1a** and PIFA in DCE: **1a** (0.3 mmol) and PIFA (0.23 mmol) were dissolved in DCE (2 mL). The mixtures were stirred for 5 min, then transformed to 1 cm path length quartz cuvettes, sealed with Teflon® septa and degassed with a stream of argon for 10 minutes.

For the solutions of PIFA in DCE: PIFA (0.23 mmol) were dissolved in DCE (2 mL). The mixtures were stirred for 5 min, then transformed to 1 cm path length quartz cuvettes, sealed with

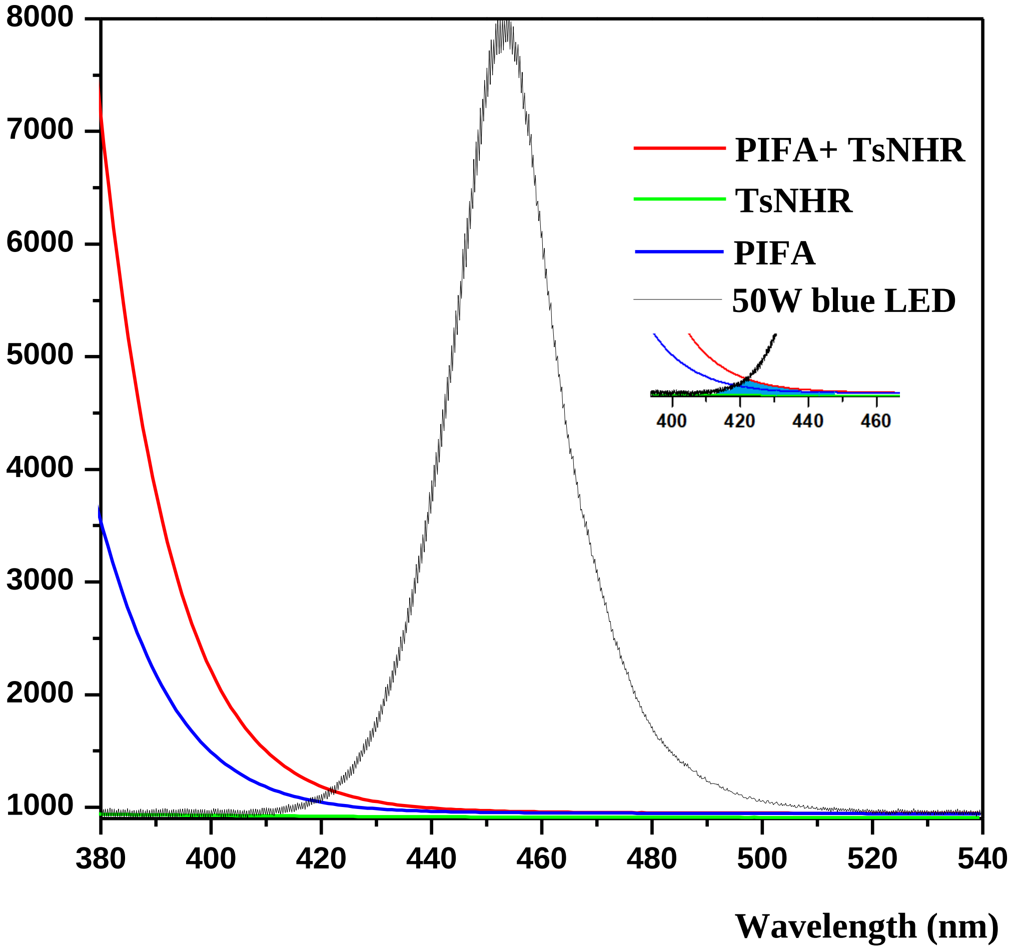

Teflon® septa and degassed with a stream of argon for 10 minutes.

Fig. 1 Absorption spectra of **1a**, PIFA, and the mixture of **1a** and PIFA, and emission spectrum of blue LEDs.

9. Determination of quantum yield

The quantum yield (Φ) was determined by the known ferrioxalate actinometry method, according to the reported procedures: W. Xu, J. Ma, X. Yuan, J. Dai, J. Xie, C. Zhu, *Angew. Chem. Int. Ed.*, **2018**, *57*, 10357-10361.

A ferrioxalate actinometry solution was prepared by following the Hammond variation of the Hatchard and Parker procedure outlined in Handbook of Photochemistry. The actinometry solutions (1mL) were irradiated with 50 W blue LEDs (400±5 nm) for specified time intervals (0 sec, 20 sec, 40 sec, 60 sec, 80 sec and 100 sec). The UV-Vis spectra was shown in **Fig 2a**.

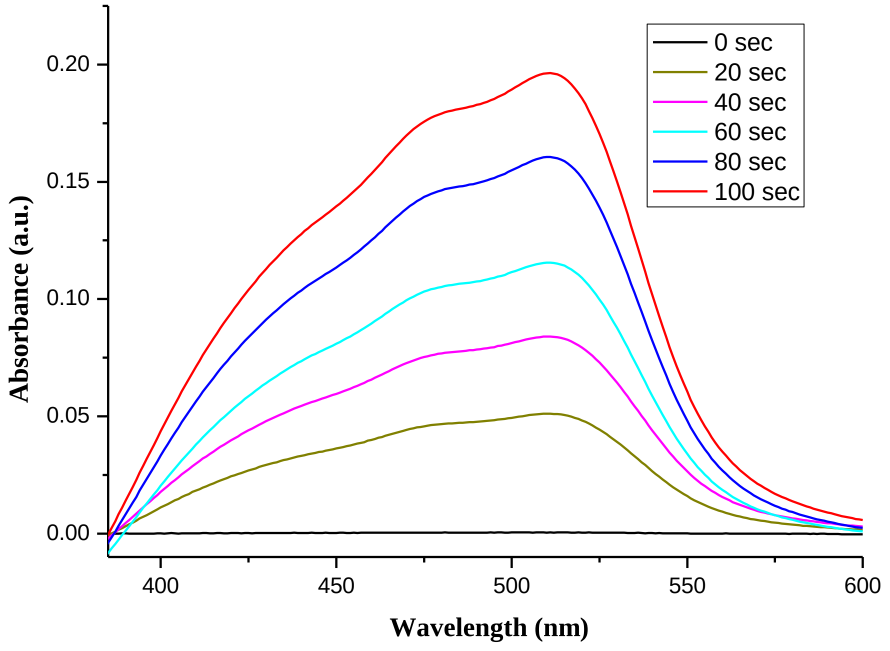

Fig 2a.

Based on the data, we got the graph (**Fig 2b**) between the number of moles of products (y axis) and time (x axis). Then, photon flux was estimated to 3.78 x 10^{-8} einstein S^{-1} by using $K_3[Fe(C_2O_4)_3]$ as an actinometer.

Photon flux may be determined by:

$$\text{Photon flux} = \frac{\text{moles } Fe^{2+}}{\Phi_{510nm} * t * F}$$

A plot of moles Fe^{2+} as a function of time yields a linear equation with an intercept at zero. Division of the slope by the documented quantum yield of the actinometer (Φ = 1.14 at 405 nm) and the mean fraction of light absorbed by the ferrioxalate solution (F ~ 1 at 402 nm) provides the photon flux in einsteins s^{-1}.

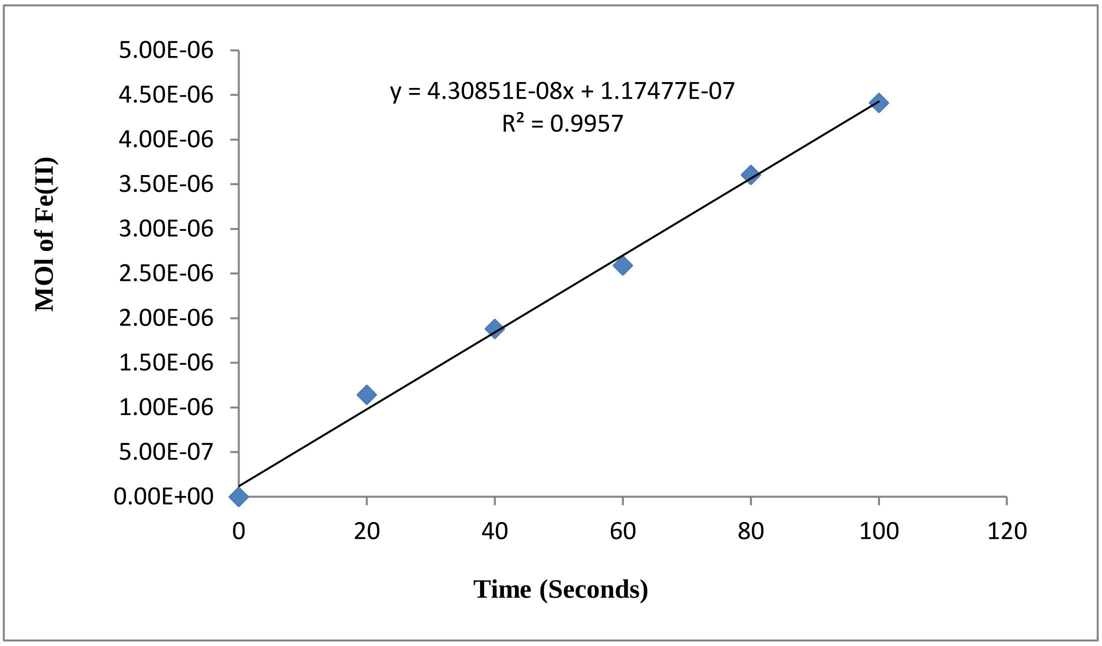

Fig 2b

$$\text{Photon flux} = \frac{\text{moles Fe}^{2+}}{\Phi_{510nm} * t * F} = 3.78 * 10^{-8} \text{ einsteins s}^{-1}$$

The slope collected is 4.31 * 10^{-8}; division by the known quantum yield Φ = 1.14 yields a photon flux of 3.78 * 10^{-8} einsteins s^{-1}.

The quantum yield is defined as:

$$\Phi = \frac{\text{Moles of Product Formed}}{\text{Photons Absorbed by Sample}}$$

If the transmittance of photons at the blue LEDs wavelength (400±5 nm) is sufficiently small, it can be assumed that all of the photons which pass through the cell are absorbed. The above equation may be then written as:

$$\Phi = \frac{\text{Moles of Product Formed}}{\text{Photon Flux} * \text{Time}} = \frac{\text{Moles of Product Formed}}{\text{Photons}}$$

According to this relationship, the experiment procedure is as follows:

For six clean tubes, according to the general procedure, the 0.1 mmol scale model reaction solution was irradiated with 50 W blue LEDs (400±5 nm) for specified time intervals (0 h, 1 h, 2 h, 3 h, 4 h and 5 h). The moles of products formed were determined by H-NMR yield with 1,3,5-trimethoxybenzene as reference standard.

Mol/ products	Mol of photons (Photon Flux*Time)	Time（s）
0.0000E+00	0.0000E+00	0
4.0000E-06	1.3606E-04	3600
1.1000E-05	2.7212E-04	7200
2.0000E-05	4.0817E-04	10800
2.4000E-05	5.4423E-04	14400
3.2000E-05	6.8029E-04	18000

Based on the data, we got the graph (**Fig.2c)**. The number of moles of products (y axis) per unit time is related to the number of photons (x axis, calculated from Photon Flux*Time). The slope gave the quantum yield (Φ) of the photoreaction, 0.048 (4.8%).

$$\Phi = \frac{\text{Moles of Product Formed}}{\text{Photon Flux} * \text{Time}} = \frac{\text{Moles of Product Formed}}{\text{Photons}} = \frac{y}{x} = 0.048$$

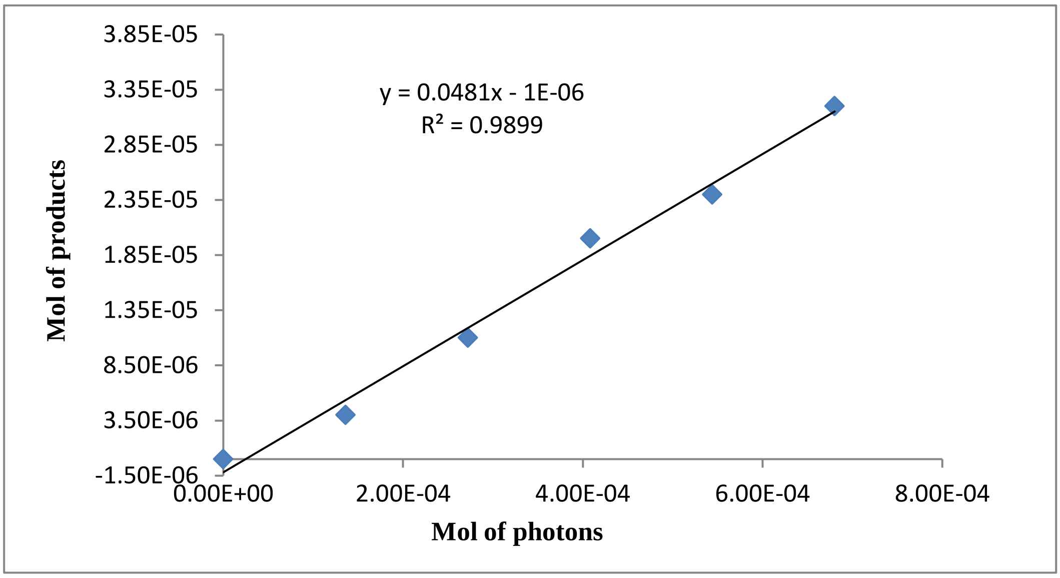

Fig 2c
